# Supplementary material for: Synthesis, structural analysis, and properties of highly twisted alkenes 13,13’-bis(dibenzo[a,i]fluorenylidene) and its derivatives
Source: Nat Commun. 2023 Aug 28;14:5248. doi: 10.1038/s41467-023-40990-8 (PMC10462764; doi:10.1038/s41467-023-40990-8)
Supplement: Supplementary file 1 — Supplementary Information [file 41467_2023_40990_MOESM1_ESM.pdf]

Supplementary Information

for

Synthesis, Structural Analysis and Properties of Highly Twisted Alkenes 13,13'-Bis(dibenzo[*a*,*i*]fluorenylidene) and Its Derivatives

Hao-Wen Kang[a], Yu-Chiao Liu[b], Wei-Kai Shao[a], Yu-Chen Wei[c], Chi-Tien Hsieh[a], Bo-Han Chen[d], Chih-Hsuan Lu[d], Shang-Da Yang[d], Mu-Jeng Cheng[a], Pi-Tai Chou[c,*], Ming-Hsi Chiang[b,e,*], Yao-Ting Wu[a,*]

[a]Department of Chemistry, National Cheng Kung University, 70101 Tainan, Taiwan.

[b]Institute of Chemistry, Academia Sinica, 11529 Taipei, Taiwan.

[c]Department of Chemistry, National Taiwan University, 10617 Taipei, Taiwan.

[d]Department of Electrical Engineering, National Tsing Hua University, 30013 Hsinchu, Taiwan.

[e]Department of Medical and Applied Chemistry, Kaohsiung Medical University, 80708 Kaohsiung, Taiwan.

Contents

**A**	Preparations	
**A1**	13,13'-Bis(dibenzo[*a*,*i*]fluorenylidene) **1a**	SI-1
**A2**	5,8-Disubstituted 13*H*-dibenzo[*a,i*]fluorenes **4b**–**4d**	SI-2
**A3**	13,13'-Bis(5,8-bis(4-tolylethyl)dibenzo[*a*,*i*]fluorenylidene) **1b**	SI-7
**A4**	13,13'-Bis(5,8-dimethoxydibenzo[*a*,*i*]fluorenylidene) **1c**	SI-7
**A5**	13,13'-Bis(5-methoxy-8-(4-tolylethyl)dibenzo[*a*,*i*]fluorenylidene) **1d**	SI-8
**A6**	15,15'-Bis(tribenzo[*a*,*c*,*i*]fluorenylidene) **2**	SI-9
**A7**	17,17'-Bis(tetrabenzo[*a,c,g,i*]fluorenylidene) **3**	SI-11
**B**	Structural Analysis	SI-13
**C**	Photophysical Properties	SI-24
**D**	Electrochemical Properties	SI-26
**E**	Magnetic properties	SI-28
**F**	Computational details	SI-33
**G**	NMR spectra	SI-35
**H**	Reference	SI-64

A. Preparations

A1. 13,13'-Bis(dibenzo[*a,i*]fluorenylidene) 1a

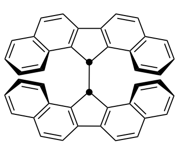

13,13'-Bis(13*H*-dibenzo[*a,i*]fluorenyl) (**7a**). To a solution of 13*H*-dibenzo[*a,i*]-fluorene (**4a**)[S1] (266.3 mg, 1.00 mmol) in anhydrous THF (10 mL) at −78 ºC, a solution of ^{n}BuLi (0.44 mL, 2.5 M in *n*-hexane, 1.10 mmol) was dropwise added. After being stirred at the same temperature for 1 h, the solution was treated with a solution of $TiCl_4$ (1.0 mL, 1.0 M in toluene, 1.00 mmol). The reaction mixture was slowly warmed to room temperature and stirred overnight. The suspensions, which were collected by filtration, rinsed with water (20 mL) and dried in vacuo, were identified as **7a** (185.7 mg, 70%). The filtrate was diluted with CH_2Cl_2 (20 mL), washed with water (2×20 mL) and dried over anhydrous $MgSO_4$. The solvents of the filtrate were removed under reduced pressure, and the residue was subjected to chromatography on silica gel. Eluting with hexane/CH_2Cl_2 (5:1) gave the title compound (53.1 mg, 20%) as a white solid, mp: >300 ºC. The combined yield for the synthesis of **7a** was 90%. ^{1}H NMR (500 MHz, $C_2D_2Cl_4$, 393 K): δ 7.88 (d, *J* = 7.7 Hz, 4H), 7.74 (d, *J* = 7.7 Hz, 4H), 7.64–7.35 (m, 12H), 7.34–7.20 (m, 4H), 5.99 (s, 2H). The ^{1}H NMR spectrum is identical to that reported in the literature.[S2] HR MS (FAB) *m*/*z*: $[M]^+$ calcd for $C_{42}H_{26}$: 530.2029; found: 530.2037. In the variable-temperature (VT) ^{1}H NMR spectra, resonances of **7a** at high temperature became very simple, but the manners of signal coalescence cannot be exactly tracked.

DFT computations on the dynamic behaviors of **7a** indicated that rocking of two DBF moieties around the central C–C bond with a barrier ($\Delta H^{\ddagger}$) of 11.6 kcal/mol (Supplementary Fig. 1) is energetically favored than the rotation via the strained eclipsed conformation as the transition state ($\Delta H^{\ddagger}$ = 19.4 kcal/mol), similar to 9,9'-bifluorenyl.[S3]

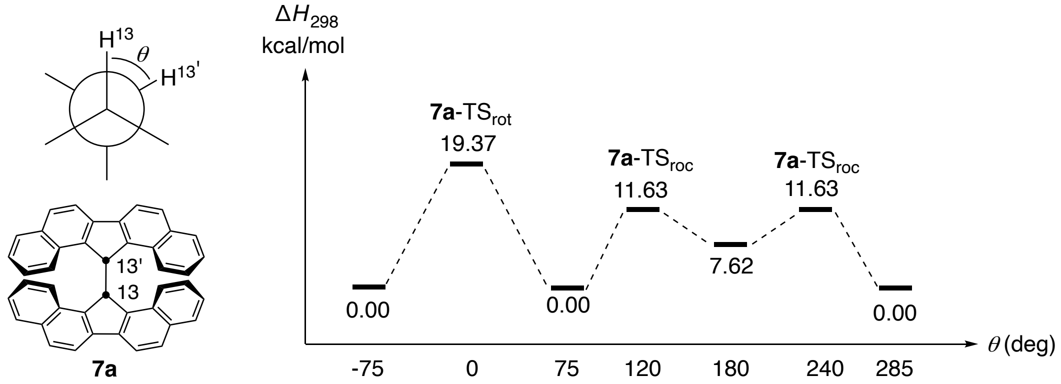

Supplementary Fig. 1: Molecular dynamics of **7a**.

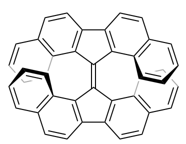

13,13'-Bis(dibenzo[*a,i*]fluorenylidene) (**1a**). A solution of **7a** (53.1 mg, 0.10 mmol) in DMF (4 mL) at room temperature was purged with nitrogen for 10 min, heated to 80 ºC under an atmosphere of N_2, treated with ^{t}BuOK (112.2 mg, 1.00 mmol) and stirred for 4 h. After being cooled to room temperature, the reaction mixture was poured into a hydrochloric acid solution (2 N, 4 mL). The solution was diluted with CH_2Cl_2 (20 mL), washed with water (2×20 mL), and dried over anhydrous $MgSO_4$. The solvents of the filtrate were removed under reduced pressure, and the residue was subjected to chromatography on spherical silica gel. Eluting with hexane/diethyl ether (5:1) gave the title compound (41.2 mg, 78%) as a green solid, mp: >300 ºC. ^{1}H NMR (400 MHz, $CDCl_3$): δ 8.02 (d, J = 8.4 Hz, 4H), 7.92 (d, J = 8.4 Hz, 4H), 7.83 (d, J = 8.4 Hz, 4H), 7.63 (d, J = 8.4 Hz, 4H), 7.00 (dd, J = 8.4, 7.6 Hz, 4H), 6.78 (dd, J = 8.4, 7.6 Hz, 4H). ^{13}C NMR (125 MHz, $CDCl_3$): 147.9, 140.6, 134.8, 134.7, 132.0, 129.5, 129.1, 127.9, 126.9, 124.5, 118.6. HRMS-MALDI, (*m*/*z*): $[M]^+$ calcd. for $C_{42}H_{24}$, 528.1873; found, 528.1875.

A2. 5,8-Disubstituted 13*H*-dibenzo[*a,i*]fluorenes 4b–4d

The reaction condition, which was utilized for synthesis of **4a**, **5** and **6**, did not efficiently convert bis(4-bromonaphthalene-1-yl)methanol and bis(4-methoxynaphthalene-1-yl)methanol (**S3**) to 5,8-dibromo-13*H*-dibenzo[*a,i*]fluorene and 5,8-dimethoxy-13*H*-dibenzo[*a,i*]fluorene (**4c**), respectively. 8-Methoxy-13*H*-dibenzo[*a,i*]fluoren-5-ol (**S5**) should be a suitable precursor for synthesis of **4b**–**4d**. As presented in Supplementary Fig. 2, the reaction of 1-lithio-4-methoxyphenanthrene with 4-methoxy-1-naphthaldehyde (**S2**) yielded bis(4-methoxynaphthalene-1-yl)methanol (**S3**), which was converted to 8-methoxy-6,6a-dihydro-13*H*-dibenzo[*a,i*]fluoren-5-one (**S4**) upon treatment of trifluoromethane-sulfonic acid (TfOH). Under basic conditions, **S4** was transferred to **S5**. Notably, **S4** was not generated from **4c** because the latter under the same condition did not yield the former. Therefore, the intermediate D in Supplementary Fig. 2 undergoes the enol ether hydrolysis, rather than the hydrogen shift(s).

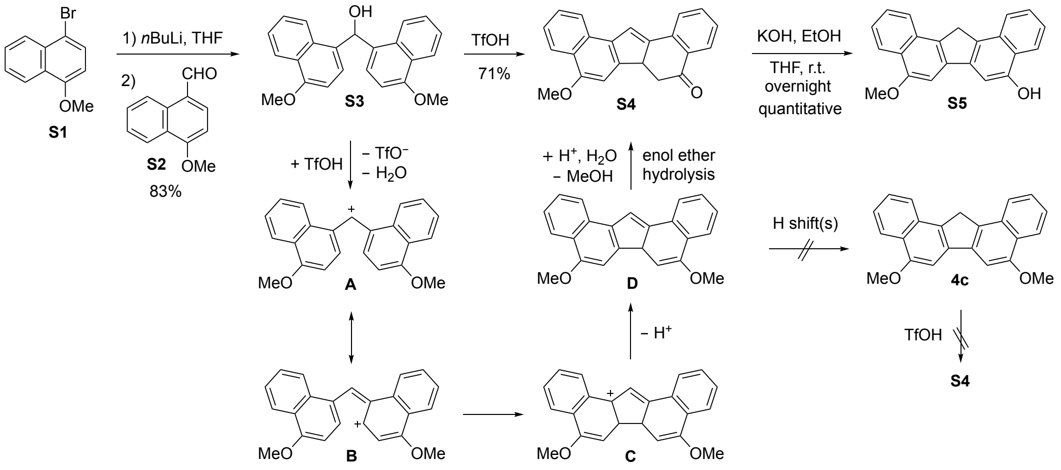

Supplementary Fig. 2: Synthesis of **S5** and a plausible formation mechanism of **S4**.

Compounds **4b**–**4d** were prepared from **S5** (Supplementary Fig. 3). **S5** was converted to dibenzo[*a,i*]fluorene-5,8-diyl bis(trifluoromethanesulfonate) **S6** by the boron tribromide-mediated demethylation and the subsequent triflations. The Sonogashira reaction of **S6** with 4-tolylethyne yielded 5,8-bis(4-tolylethynyl)-13*H*-dibenzo[*a,i*]fluorene (**4b**). The methylation of **S5** generated **4c**. 8-Methoxy-13*H*-dibenzo[*a,i*]fluoren-5-yl trifluoromethanesulfonate (**4d**) was obtained by the triflation of **S5** and the subsequent Sonogashira reaction with 4-tolylethyne.

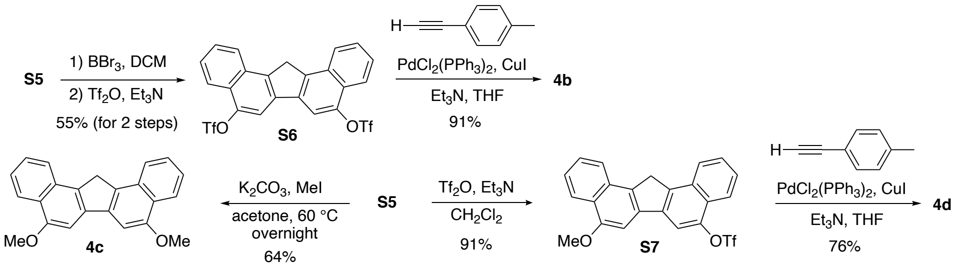

Supplementary Fig. 3: Synthesis of 5,8-disubstituted 13*H*-dibenzo[*a,i*]fluorenes **4b**, **4c** and **4d**.

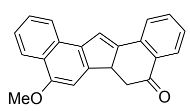

8-Methoxy-6,6a-dihydro-13*H*-dibenzo[*a,i*]fluoren-5-one (**S4**):

1) Preparation of bis(4-methoxynaphthalene-1-yl)methanol (**S3**). To a solution of 1-bromo-4-methoxyphenanthrene (1.90 mL, 12.0 mmol) in anhydrous THF (40 mL) at −78 ºC, a solution of ^{n}BuLi (4.80 mL, 2.5 M in *n*-hexane, 12.0 mmol) was dropwise added. After being stirred at the same temperature for 1 hour, the solution was treated with a solution of 4-methoxy-1-naphthaldehyde (**S2**)[S4] (1.86 g, 10.0 mmol) in anhydrous THF (20 mL). The reaction mixture was kept at the same temperature for additional 2 hours, warmed up to room temperature, and poured into a hydrochloric acid solution (2N, 20 mL). The aqueous layer was separated and extracted with ethyl acetate (2×40 mL). The combined organic layers were washed with water (2×40 mL), and dried over anhydrous $MgSO_4$. The solvents of the filtrate were removed under reduced pressure, and the residue

was subjected to chromatography on silica gel. Eluting with ethyl acetate/hexane (1:5) gave the title compound (2.87 g, 83%) as a white solid.

2) TfOH-mediated cycloisomerization. **S3** (0.69 g, 2.00 mmol) was dissolved in TfOH (4 mL) at room temperature, and the solution was stirred for 1 h. The reaction mixture was poured into crushed ice, and diluted with CH_2Cl_2 (50 mL). The organic layer was separated and washed with water (2×50 mL), and dried over anhydrous $MgSO_4$. The solvent of the filtrate was removed under reduced pressure, and the residue was subjected to chromatography on silica gel. Eluting with CH_2Cl_2 gave **S4** (0.45 g, 71%) as a bright yellow solid, mp: 185–186 ºC. ^{1}H NMR (400 MHz, $CDCl_3$): δ 8.36 (d, J = 8.4 Hz, 1H), 8.18 (d, J = 8.4 Hz, 1H), 8.09 (d, J = 8.4 Hz, 1H), 7.86 (d, J = 7.6 Hz, 1H), 7.74 (s, 1H), 7.67–7.61 (m, 2H), 7.54 (t, J = 8.4 Hz, 1H), 7.39 (t, J = 8.4 Hz, 1H), 7.08 (s, 1H), 4.20 (dd, J = 14.4, 5.6 Hz, 1H), 4.10 (s, 3H), 3.57 (dd, J = 15.2, 5.6 Hz, 1H), 2.44 (dd, J = 15.2, 14.4 Hz, 1H). ^{13}C NMR (125 MHz, $CDCl_3$): δ 197.6, 155.0, 145.8, 142.3, 137.7, 134.1, 133.8, 129.6, 128.7, 127.7, 127.5, 126.9, 125.1, 125.0, 124.4, 123.5, 123.2, 123.0, 100.5, 55.8, 49.7, 43.0. HRMS-EI (*m*/*z*): $[M]^+$ cacld. for $C_{22}H_{16}O_2$, 312.1145; found, 312.1143.

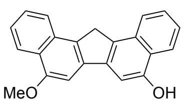

8-Methoxy-13*H*-dibenzo[*a,i*]fluoren-5-ol (**S5**). To a solution of **S4** (0.31 g, 1.00 mmol) in anhydrous THF (25 mL) at room temperature, a saturated solution of KOH (1.5 mL) in EtOH was added. After being stirred for 1 h, the reaction mixture was treated with an saturation aqueous solution of NH_4Cl (25 mL) and diluted with ethyl acetate (50 mL). The organic layer was separated, washed with water (2×50 mL) and dried over anhydrous $MgSO_4$. The solvents of the filtrate were removed under reduced pressure, quantitatively yielding the title compound as a white solid, mp: 176–177 ºC (dec.). ^{1}H NMR (400 MHz, $CDCl_3$): δ 8.32 (d, J = 8.4 Hz, 1H), 8.24 (d, J = 8.4 Hz, 1H), 8.05 (d, J = 8.4 Hz, 2H), 7.58 (t, ^3J = 7.2 Hz, 2H), 7.49–7.43 (m, 2H), 7.33 (s, 1H), 7.23 (s, 1H), 5.39 (s, 1H), 4.33 (s, 2H), 4.13 (s, 3H). Due to the gradual decomposition in $CDCl_3$, the ^{13}C NMR spectrum of **S5** was not recorded. HRMS-FAB (*m*/*z*): $[M]^+$ cacld. for $C_{22}H_{16}O_2$, 312.1145; found, 312.1147.

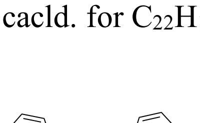

13*H*-Dibenzo[*a,i*]fluorene-5,8-diyl bis(trifluoromethanesulfonate) (**S6**):

1) Demethylation of **S5**. To a solution of **S5** (156.2 mg, 0.50 mmol) in anhydrous CH_2Cl_2 (40 mL) under nitrogen atmosphere at room temperature was added BBr_3 (2 mL, 1.0 M in CH_2Cl_2, 2.00 mmol). After being stirred overnight, the solution at 0 ºC was treated with water (20 mL), and diluted with ethyl acetate (20 mL). The organic layer was separated, washed with water (2×20 mL) and dried over anhydrous $MgSO_4$. The solvents of the filtrate were removed under reduced pressure, giving 5,8-dihydroxy-13*H*-dibenzo[*a,i*]fluorene, which was directly used in the next step.

2) Triflations of 5,8-dihydroxy-13*H*-dibenzo[*a,i*]fluorene. To a solution of 5,8-dihydroxy-13*H*-dibenzo[*a,i*]fluorene in anhydrous CH_2Cl_2 (40 mL) at room temperature, Et_3N (0.25 mL, 1.50 mmol) and Tf_2O (0.56 mL, 2.00 mmol) were added. After being stirred overnight, the reaction mixture was treated with water (20 mL) and diluted with CH_2Cl_2 (20 mL). The organic layer was separated, washed with water (2×20 mL) and dried over anhydrous $MgSO_4$. The solvents of the filtrate were removed under reduced pressure, and the residue was subjected to chromatography on silica gel. Eluting with hexane/CH_2Cl_2 (2:1) gave the title compound (155.6 mg, 55% for two steps) as a white solid, mp: 238–239 ºC. ^{1}H NMR (500 MHz, $CDCl_3$): δ 8.17–8.14 (m, 4H), 7.87 (s, 2H), 7.71 (ddd, *J* = 8.0, 7.0, 1.5 Hz, 2H), 7.66 (ddd, *J* = 8.0, 7.0, 1.5 Hz, 2H), 4.51 (s, 2H). ^{13}C NMR (125 MHz, $CDCl_3$): δ 145.8, 140.2, 138.2, 131.4, 128.4, 127.6, 125.6, 124.3, 122.3, 118.8 (q, *J* = 318.6 Hz), 111.0, 34.6. HRMS-FAB (*m*/*z*): [M]$^+$ calcd for $C_{23}H_{12}O_6F_6S_2$, 561.9980; found, 561.9979.

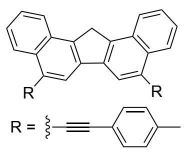

5,8-Bis(4-tolylethynyl)-13*H*-dibenzo[*a,i*]fluorene (**4b**): A mixture of **S6** (112.5 mg, 0.20 mmol), 4-ethynyltoluene (76.1 μL, 0.60 mmol), $PdCl_2(PPh_3)_2$ (14.0 mg, 0.02 mmol), CuI (3.8 mg, 0.02 mmol), Et_3N (2 mL) and THF (2 mL) in a thick-walled Pyrex tube was purged with nitrogen for 5 min. The sealed tube was kept in an oil bath at 40 ºC for 16 h. After being cooled to room temperature, the precipitates were collected by filtration and rinsed with MeOH, giving the title compound (90.3 mg, 91%) as an off-white solid, mp: 245–246 ºC. ^{1}H NMR (500 MHz, $CDCl_3$): δ 8.52 (d, *J* = 7.5 Hz, 2H), 8.23 (s, 2H), 8.10 (d, *J* = 7.5 Hz, 2H), 7.63–7.56 (m, 8H), 7.24 (d, *J* = 8.0 Hz, 4H), 4.49 (s, 2H), 2.42 (s, 6H). ^{13}C NMR (125 MHz, $CDCl_3$): δ 140.5, 138.9, 138.5, 132.7, 131.6, 130.5, 129.2, 127.5, 127.1, 126.1, 124.3, 122.9, 121.0, 120.4, 94.4, 87.4, 35.0, 21.6. HRMS-FAB (*m*/*z*): [M]$^+$ calcd. for $C_{39}H_{26}$, 494.2034; found, 494.2033.

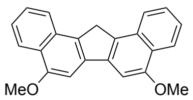

5,8-Dimethoxy-13*H*-dibenzo[*a,i*]fluorene (**4c**): A mixture of **S5** (374.9 mg, 1.20 mmol), K_2CO_3 (663.4 mg, 4.80 mmol), MeI (0.37 mL, 6.00 mmol) and acetone (12 mL) in a thick-walled Pyrex tube was purged with nitrogen for 5 min. The sealed tube was kept in an oil bath at 60 ºC for 16 h. After being cooled to room temperature, the solution was poured in water (10 mL) and diluted with CH_2Cl_2 (20 mL). The organic layer was separated, washed with water (2×10 mL) and dried over anhydrous $MgSO_4$. The solvents of the filtrate were removed under reduced pressure, and the residue was subjected to chromatography on silica gel. Eluting with hexane/CH_2Cl_2 (2:1) gave the title compound (251.2 mg, 64%) as a white solid, mp: 209–210 ºC. ^{1}H NMR (400 MHz, $CDCl_3$): δ 8.33 (d, *J* = 8.0 Hz, 2H), 8.04 (d, *J* = 8.0 Hz, 2H), 7.57 (t, *J* = 8.0 Hz, 2H), 7.45 (t, *J* = 8.0 Hz, 2H), 7.28 (s, 2H), 4.31 (s, 2H), 4.16 (s, 6H). ^{13}C NMR (125 MHz, $CDCl_3$): δ 155.8, 140.0, 132.2, 131.3, 127.0, 124.9, 124.4, 123.8, 123.3, 97.0, 55.8, 33.6. HRMS-FAB (*m*/*z*): [M]$^+$ calcd. for $C_{23}H_{18}O_2$, 326.1306; found, 326.1303.

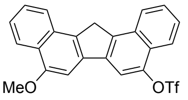

8-Methoxy-13*H*-dibenzo[*a,i*]fluoren-5-yl trifluoromethanesulfonate (**S7**). To a solution of **S5** (156.2 mg, 0.50 mmol) in anhydrous DCM (40 mL) at room temperature, Et_3N (0.56 mL, 2.00 mmol) and Tf_2O (0.21 mL, 1.25 mmol) were added. After being stirred overnight, the solution was treated with water (20 mL) and diluted with CH_2Cl_2 (20 mL). The organic layer was separated, washed with water (2×20 mL) and dried over anhydrous $MgSO_4$. The solvent of the filtrate was removed under reduced pressure. The residue was subjected to chromatography on silica gel, eluting with hexane/CH_2Cl_2 (2:1) to give the title compound (201.8 mg, 91%) as a white solid, m.p. 172–173 ºC. ^{1}H NMR (500 MHz, $CDCl_3$): δ 8.35 (d, *J* = 8.0 Hz, 1H), 8.15 (d, *J* = 8.0 Hz, 1H), 8.13 (d, *J* = 8.0 Hz, 1H), 8.04 (d, *J* = 8.0 Hz, 1H), 7.91 (s, 1H), 7.67 (t, *J* = 8.0 Hz, 1H), 7.61 (t, *J* = 8.0 Hz, 2H), 7.50 (t, *J* = 8.0 Hz, 1H), 7.24 (s, 1H), 4.42 (s, 2H), 4.16 (s, 3H). ^{13}C NMR (125 MHz, $CDCl_3$): δ 156.2, 145.6, 140.5, 139.9, 138.4, 131.9, 131.5, 131.1, 127.9, 127.4, 126.8, 125.4, 125.1, 124.9, 124.4, 123.8, 123.5, 122.0, 110.9, 96.7, 55.9, 34.1. The signal of CF_3 was not observed. HRMS-FAB (*m*/*z*): $[M]^+$ calcd. for $C_{23}H_{15}O_4F_3S$, 444.0643; found, 444.0642.

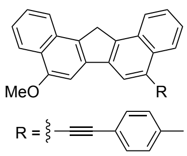

5-Methoxy-8-(4-tolylethynyl)-13*H*-dibenzo[*a,i*]fluorene (**4d**). A mixture of **S7** (177.6 mg, 0.40 mmol), 4-ethynyltoluene (76.1 μL, 0.60 mmol), $PdCl_2(PPh_3)_2$ (28.1 mg, 0.04 mmol), CuI (7.6 mg, 0.04 mmol), Et_3N (4 mL) and THF (4 mL) in a thick-walled Pyrex tube was purged with nitrogen for 5 min. The sealed tube was kept in an oil bath at 40 ºC for 16 h. After cooling to room temperature, the reaction mixture was poured in water (10 mL) and diluted with CH_2Cl_2 (20 mL). The organic layer was separated, washed with water (2×10 mL) and dried over anhydrous $MgSO_4$. The solvents of the filtrate were removed under reduced pressure, and the residue was subjected to chromatography on silica gel. Eluting with hexane/CH_2Cl_2 (2:1) gave the title compound (124.5 mg, 76%) as a yellow solid, mp: 168–169 ºC. ^{1}H NMR (500 MHz, $CDCl_3$): δ 8.53 (d, *J* = 8.0 Hz, 1H), 8.34 (d, *J* = 8.0 Hz, 1H), 8.24 (s, 1H), 8.13 (d, *J* = 8.0 Hz, 1H), 8.04 (d, *J* = 8.0 Hz, 1H), 7.62–7.55 (m, 5H), 7.47 (t, *J* = 8.0 Hz, 1H), 7.33 (s, 1H), 7.23 (d, *J* = 8.0 Hz, 2H), 4.43 (s, 2H), 4.15 (s, 3H), 2.42 (s, 3H). ^{13}C NMR (125 MHz, $CDCl_3$): δ 156.0, 141.2, 139.8, 139.2, 138.5, 131.6, 131.2, 132.5, 130.6, 129.3, 127.5, 127.2, 127.0, 125.9, 125.1, 124.7, 124.4, 123.8, 123.4, 122.7, 120.6, 120.5, 97.0, 94.2, 87.5, 55.8, 34.3, 21.6. Due to overlapping signals, one C_{quat} was not observed. HRMS-FAB (*m*/*z*): $[M]^+$ calcd. for $C_{31}H_{22}O$, 410.1671; found, 410.1669.

A3. 13,13'-Bis(5,8-bis(4-tolylethyl)dibenzo[*a*,*i*]fluorenylidene) 1b

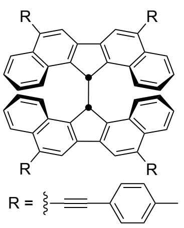

13,13'-Bis[5,8-bis(4-tolylethynyl)-13*H*-dibenzo[*a,i*]fluorenyl] (**7b**). To a solution of **4b** (122.3 mg, 0.25 mmol) in anhydrous THF (5 mL) at −78 ºC, a solution of ^{n}BuLi (0.11 mL, 2.5 M in *n*-hexane, 0.28 mmol) was dropwise added. After being stirred at the same temperature for 1 h, the solution was treated with a solution of $TiCl_4$ (0.25 mL, 1.0 M in toluene, 0.25 mmol). The reaction mixture was slowly warmed to room temperature and stirred overnight. The precipitates were collected by filtration and rinsed with MeOH, giving the title compound (98.6 mg, 80%) as a white solid, mp: >300 ºC. HRMS-FAB (*m*/*z*): $[M]^+$ calcd. for $C_{78}H_{50}$: 986.3913; found: 986.3911.

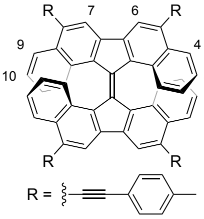

13,13'-Bis[5,8-bis(4-tolylethynyl)dibenzo[*a,i*]fluorenylidene] (**1b**): In a thick-walled Pyrex tube, a solution of **7b** (10 mg, 0.01 mmol) in DMSO (1.0 mL) at room temperature was purged with nitrogen for 10 min. After being treated with ^{t}BuOK (4.5 mg, 0.04 mmol), the sealed tube was kept at the same temperature for 1 h. The reaction mixture was poured into water (1 mL), treated with a hydrochloric acid solution (2N, 1 mL), and diluted with CH_2Cl_2 (5 mL). The solution was washed with water (2×5 mL), and dried over anhydrous $MgSO_4$. The solvents of the filtrate were removed under reduced pressure, and the residue was subjected to chromatography on spherical silica gel. Eluting with hexane/CH_2Cl_2 (5:1) gave the title compound (3.5 mg, 35%) as a black solid, mp: >300 ºC. One additional compound (5.4 mg) was collected, and its FAB MS spectrum showed a peak at *m*/*z* = 1984.5. ^{1}H NMR (500 MHz, $CDCl_3$): δ 8.30 (d, *J* = 8.0 Hz, 4H, 1,12-H), 8.23 (s, *J* = 8.0 Hz, 4H, 6,7-H), 8.06 (d, *J* = 7.5 Hz, 4H, 4,9-H), 7.60 (d, *J* = 8.0 Hz, 8H, tolyl), 7.25 (d, *J* = 8.0 Hz, 8H, tolyl), 7.14 (t, *J* = 7.5 Hz, 4H, 2,11-H), 6.88 (br, 4H, 3,10-H), 2.43 (s, 12H). ^{13}C NMR (125 MHz, $CDCl_3$): δ 139.0, 131.7, 129.3, 128.7, 127.5, 126.9, 125.4, 120.2, 21.6. Some carbon signals are missing due to overlapping signals and/or line broadening. HRMS-FAB (*m*/*z*): $[M+H]^+$ calcd. for $C_{78}H_{49}$, 985.3829; found, 985.3830.

A4. 13,13'-Bis(5,8-dimethoxydibenzo[*a*,*i*]fluorenylidene) 1c

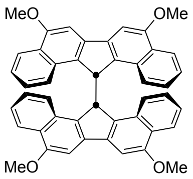

13,13'-Bis(5,8-dimethoxy-13*H*-dibenzo[*a,i*]fluorenyl) (**7c**): To a solution of **4c** (160.7 mg, 0.50 mmol) in anhydrous THF (5 mL) at −78 ºC, a solution of ^{n}BuLi (0.22 mL, 2.5 M in *n*-hexane, 0.55 mmol) was dropwise added. After being stirred at the same temperature for 1 h, the solution was treated with a solution of $TiCl_4$ (0.5 mL, 1.0 M in toluene, 0.50 mmol). The reaction mixture was slowly warmed to room temperature and stirred overnight. The precipitates were collected by filtration, and rinsed with MeOH, giving the

title compound (131.7 mg, 82%) as a white solid, mp: >300 ºC. HR MS (FAB) *m*/*z*: $[M]^+$ calcd for $C_{46}H_{34}O_4$: 650.2452; found: 650.2461.

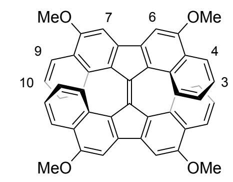

13,13'-Bis(5,8-dimethoxydibenzo[*a,i*]fluorenylidene) (**1c**): A solution of **7c** (23 mg, 0.04 mmol) in DMF (1.6 mL) at room temperature was purged with nitrogen for 10 min, heated to 80 ºC under an atmosphere of N_2, treated with ^{t}BuOK (39.7 mg, 0.35 mmol) and stirred overnight. After being cooled to room temperature, the solution was poured into water (2 mL) and acidified with a hydrochloric acid solution (2N, 2 mL). The solution was diluted with CH_2Cl_2 (5 mL), washed with water (2×5 mL) and dried over anhydrous $MgSO_4$. The solvents of the filtrate were removed under reduced pressure, and the residue was subjected to chromatography on spherical silica gel. Eluting with cyclohexane/diethyl ether (5:1) gave the title compound (13.8 mg, 60%) as a dark green solid, mp: >300 ºC. ^{1}H NMR (500 MHz, $CDCl_3$): δ 8.11 (d, *J* = 8.5 Hz, 4H, 1,12-H), 8.08 (d, *J* = 8.5 Hz, 4H, 4,9-H), 7.28 (s, 4H, 6,7-H), 7.00 (t, *J* = 8.5 Hz, 4H, 3,10-H), 6.80 (t, *J* = 8.5 Hz, 4H, 2,11-H), 4.24 (s, 12H). ^{13}C NMR (125 MHz, $CDCl_3$): δ 157.7, 140.4, 130.5, 128.7, 127.9, 126.9, 125.9, 123.7, 122.6, 97.4, 55.9. Due to overlapping signals, one C_{quat} was not observed. HRMS-FAB (*m*/*z*): $[M+H]^+$ calcd. for $C_{46}H_{33}O_4$, 649.2373; found, 649.2382.

A5. Bis(5-methoxy-8-*p*-tolylethynyldibenzo[*a,i*]fluorenylidene) 1d

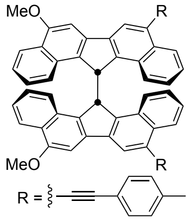

13,13'-Bis[5-methoxy-8-(4-tolylethynyl)-13*H*-dibenzo[*a,i*]fluorenyl] (**7d**). To a solution of **4d** (123.2 mg, 0.30 mmol) in anhydrous THF (4 mL) at −78 ºC was dropwise added a solution of ^{n}BuLi (0.13 mL, 2.5 M in *n*-hexane, 0.33 mmol). After being stirred at the same temperature for 1 h, the solution was treated with a solution of $TiCl_4$ (0.30 mL, 1.0 M in toluene, 0.30 mmol). The reaction mixture was slowly warmed to room temperature and stirred overnight. The precipitates were collected by filtration, and rinsed with MeOH, giving the title compound (96.1 mg, 78%) as a white solid, m. p. >300 ºC. HR MS (FAB) *m*/*z*: calcd $[M]^+$ for $C_{62}H_{42}O_2$: 818.3185; found: 818.3185.

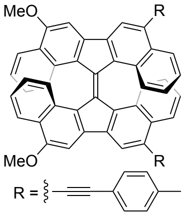

13,13'-Bis[5-methoxy-8-(4-tolylethynyl)-13*H*-dibenzo[*a,i*]fluorenylidene] (**1d**): In a thick-walled Pyrex tube, a solution of **7d** (25.7 mg, 0.03 mmol) in DMSO (3.0 mL) at room temperature was purged with nitrogen for 10 min. After being treated with ^{t}BuOK (14.1 mg, 0.13 mmol), the sealed tube was kept at the same temperature for 1 h. The reaction mixture was poured into water (3 mL), treated with a hydrochloric acid solution

(2N, 3 mL), and diluted with CH_2Cl_2 (10 mL). The solution was washed with water (2×5 mL), and dried over anhydrous $MgSO_4$. The solvents of the filtrate were removed under reduced pressure, and the residue was subjected to chromatography on spherical silica gel. Eluting with hexane/CH_2Cl_2 (4:1) gave the title compound (11.0 mg, 43%) as a dark green solid, mp: >300 ºC. One additional compound (10.3 mg) was collected, and its MALDI MS spectrum showed a peak at *m*/*z* = 1635.5. ^{1}H NMR (500 MHz, $CDCl_3$): δ 8.27 (d, *J* = 7.5 Hz, 2H), 8.19 (s, 2H), 8.11–8.07 (m, 6H), 7.59 (d, *J* = 8.0 Hz, 4H), 7.35 (s, 2H), 7.24 (d, *J* = 8.0 Hz, 4H), 7.10 (t, *J* = 7.5 Hz, 2H), 7.04 (t, *J* = 7.5 Hz, 2H), 6.86–6.81 (m, 4H), 4.24 (s, 6H), 2.42 (s, 6H). ^{13}C NMR (125 MHz, $CDCl_3$): δ 138.9, 135.7, 131.7, 129.6, 129.4, 128.7, 128.1, 127.6, 127.3, 126.5, 125.1, 124.3, 123.7, 123.1, 120.5, 98.0, 97.1, 56.1, 21.8. Some carbon signals are missing due to overlapping signals and/or line broadening. HRMS-FAB (*m*/*z*): [M]$^+$ calcd. for $C_{62}H_{40}O_2$, 816.3023; found, 816.3026.

A6. 15,15'-Bis(tribenzo[*a*,*c*,*i*]fluorenylidene) 2

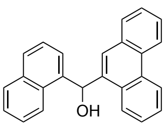

(1-Naphthyl)-(9-phenanthryl)methanol (**S8**). To a solution of 9-bromophenanthrene (3.09 g, 12.0 mmol) in anhydrous THF (40 mL) at −78 ºC was added dropwise a solution of ^{n}BuLi (4.80 mL, 2.5 M in *n*-hexane, 12.0 mmol). After being stirred for 1 h, the solution was treated with a solution of 1-naphthaldedehyde (1.36 mL, 10.00 mmol) in anhydrous THF (20 mL). The reaction mixture was kept at the same temperature for an additional 2 h, warmed up to room temperature, and poured into a hydrochloric acid solution (2N, 20 mL). The aqueous layer was extracted with ethyl acetate (2×40 mL). The combined organic layer was washed with water (2×40 mL), and dried over anhydrous $MgSO_4$. The solvents of the filtrate were removed under reduced pressure, and the residue was subjected to chromatography on silica gel. Eluting with hexane/ethyl acetate (5:1) gave the title compound (3.00 g, 90%) as a white solid, mp: 116–117 ºC. ^{1}H NMR (500 MHz, $CDCl_3$): δ 8.76 (d, *J* = 8.0 Hz, 1H), 8.69 (d, *J* = 8.0 Hz, 1H), 8.20–8.18 (m, 1H), 7.94–7.92 (m, 2H), 7.85 (s, 1H), 7.83 (d, *J* = 8.5 Hz, 1H), 7.80 (dd, *J* = 8.0, 1.5 Hz, 1H), 7.68–7.62 (m, 2H), 7.57 (ddd, *J* = 8.0, 7.0, 1.0 Hz, 1H), 7.54–7.47 (m, 3H), 7.41 (dd, *J* = 7.0, 1.5 Hz, 1H), 7.37–7.34 (m, 1H), 7.28 (s, 1H), 2.54 (br, 1H, OH). ^{13}C NMR (125 MHz, $CDCl_3$): δ 138.1, 136.6, 134.0, 131.4, 131.2, 130.9, 130.4, 130.0, 129.1, 128.9, 128.8, 126.92, 126.86, 126.8, 126.7, 126.4, 125.9, 125.8, 125.5, 125.3, 124.5, 123.5, 123.2, 122.5, 69.7. HRMS-FAB (*m*/*z*): [M]$^+$ calcd. for $C_{25}H_{18}O$, 334.1352; found, 334.1360.

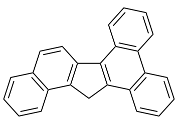

15*H*-tribenzo[*a,c,i*]fluorene (**5**). **S8** (3.00 g, 9.00 mmol) and polyphosphoric acid (14.4 mL) were stirred at 180 ºC for 6 h. After being cooled to room temperature, the reaction mixture was poured into crushed ice and diluted with CH_2Cl_2 (50 mL). The

organic layer was washed with water (2×50 mL) and dried over anhydrous $MgSO_4$. The solvents of the filtrate were removed under reduced pressure, and the residue was subjected to chromatography on silica gel. Eluting with hexane/CH_2Cl_2 (5:1) gave the title compound (1.79 g, 63%) as a white solid, mp: 227–228 ºC. ^{1}H NMR (500 MHz, $CDCl_3$): δ 8.97 (d, J = 8.0 Hz, 1H), 8.85 (d, J = 8.5 Hz, 1H), 8.76 (d, J = 8.0 Hz, 1H), 8.58 (d, J = 9.0 Hz, 1H), 8.23–8.21 (m, 1H), 8.18 (d, J = 8.5 Hz, 1H), 7.99 (d, J = 8.5 Hz, 1H), 7.95 (d, J = 8.0 Hz, 1H), 7.79 (ddd, J = 8.0, 7.0, 1.5 Hz, 1H), 7.74–7.64 (m, 3H), 7.59 (ddd, J = 8.0, 7.0, 1.5 Hz, 1H), 7.50 (ddd, J = 8.0, 7.0, 1.5 Hz, 1H), 4.56 (s, 2H). ^{13}C NMR (125 MHz, $CDCl_3$): δ 140.81, 140.78, 139.8, 135.6, 131.8, 131.0, 130.4, 129.7 (×2), 129.2, 128.7, 127.7, 127.1, 126.8, 126.5, 126.2, 125.8, 125.4, 124.6, 124.3, 124.0, 123.7, 123.4, 121.6, 35.2. HRMS-MALDI (m/z): $[M]^+$ calcd. for $C_{25}H_{16}$, 316.1247; found, 316.1253.

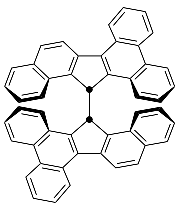

15,15'-Bis(15*H*-tribenzo[*a,c,i*]fluorenyl) (**8**). To a solution of **5** (316.4 mg, 1.00 mmol) in anhydrous THF (30 mL) at −78 ºC was dropwise added a solution of ^{n}BuLi (0.44 mL, 2.5 M in *n*-hexane, 1.10 mmol). After being stirred at the same temperature for 1 h, the solution was treated with a solution of $TiCl_4$ (1.00 mL, 1.0 M in toluene, 1.00 mmol). The reaction mixture was slowly warmed to room temperature and stirred overnight. The suspensions, which were collected by filtration, rinsed with water (20 mL) and dried in vacuo, was identified as **8** (157.7 mg, 50%). The filtrate was diluted with CH_2Cl_2 (20 mL), washed with water (2×20 mL) and dried over anhydrous $MgSO_4$. The solvents of the filtrate were removed under reduced pressure, and the residue was subjected to chromatography on silica gel. Eluting with hexane/CH_2Cl_2 (5:1) gave the title compound (63.1 mg, 20%) as a bright yellow solid, m. p. >300 ºC. The combined yield for the synthesis of **8** was 70%. ^{1}H NMR (500 MHz, CD_2Cl_2, 203 K): δ 9.18 (d, J = 7.5 Hz, 1H), 9.13 (d, J = 7.5 Hz, 1H), 9.08 (d, J = 8.0 Hz, 1H), 8.98 (d, J = 8.0 Hz, 1H), 8.46 (d, J = 8.5 Hz, 1H), 8.24 (d, J = 8.0 Hz, 1H), 8.21–8.15 (m, 3H), 8.10–8.06 (m, 2H), 8.03 (d, J = 8.5 Hz, 1H), 7.98–7.90 (m, 2H), 7.76 (t, J = 7.5 Hz, 1H), 7.72 (t, J = 7.5 Hz, 1H), 7.63 (d, J = 8.5 Hz, 1H), 7.54 (t, J = 7.5 Hz, 1H), 7.49 (t, J = 7.5 Hz, 1H), 7.42 (t, J = 7.5 Hz, 1H), 7.39 (d, J = 8.0 Hz, 1H), 7.16 (d, J = 8.5 Hz, 1H), 7.05 (t, J = 7.5 Hz, 1H), 6.79 (t, J = 7.5 Hz, 1H), 6.45 (t, J = 7.5 Hz, 1H), 5.96–5.90 (m, 3H), 5.88 (d, J = 7.5 Hz, 1H), 5.72 (d, J = 7.5 Hz, 1H). Signals of **8** in $CDCl_3$ were not observed in the ^{13}C NMR spectrum although the sample has been scanned 24000 times. HRMS-MALDI (m/z): $[M+H]^+$ calcd. for $C_{50}H_{31}$, 631.2420; found, 631.2428. HRMS-FAB (m/z): $[M]^+$ calcd for $C_{50}H_{30}$: 630.2342; found: 630.2346.

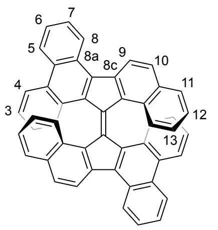

15,15'-Bis(tribenzo[*a,c,i*]fluorenylidene) (**2**). A solution of **8** (63.1 mg, 0.10 mmol) in DMSO (4 mL) at room temperature was purged with nitrogen for 10 min, heated to 80 ºC under an atmosphere of N_2, treated with ^{t}BuOK (112.2 mg, 1.00 mmol) and stirred overnight (around 18 h). After being cooled to room temperature, the reaction mixture was poured into a hydrochloric acid solution (2N, 4 mL). The solution was diluted with CH_2Cl_2 (20 mL), washed with water (2×20 mL), and dried over anhydrous $MgSO_4$. The solvents of the filtrate were removed under reduced pressure, and the residue was subjected to chromatography on spherical silica gel. Eluting with hexane/ether (5:1) gave the title compound (45.3 mg, 72%) as a blue solid, mp: 279–280 ºC. ^{1}H NMR (700 MHz, $CDCl_3$): δ 9.02 (d, *J* = 8.4 Hz, 2H, 8-H), 8.70 (d, *J* = 8.4 Hz, 2H, 5-H), 8.57 (d, *J* = 9.1 Hz, 2H, 9-H), 8.47 (d, *J* = 8.4 Hz, 2H, 4-H), 8.25 (d, *J* = 8.4 Hz, 2H, 1-H), 8.09 (d, *J* = 8.4 Hz, 2H, 14-H), 7.87 (br, 2H, 10-H), 7.78 (t, *J* = 7.7 Hz, 2H, 7-H), 7.71 (br, 2H, 6-H), 7.61 (d, *J* = 8.4 Hz, 2H, 11-H), 7.18 (t, *J* = 7.7 Hz, 2H, 3-H), 6.99 (t, *J* = 7.7 Hz, 2H, 12-H), 6.86 (t, *J* = 7.7 Hz, 2H, 2-H), 6.76 (t, *J* = 7.7 Hz, 2H, 13-H). ^{1}H NMR (500 MHz, $CDCl_3$, 223 K): δ 9.06 (d, *J* = 8.0 Hz, 2H), 8.74 (d, *J* = 8.0 Hz, 2H), 8.63 (d, *J* = 8.0 Hz, 2H), 8.54 (d, *J* = 8.0 Hz, 2H), 8.29 (d, *J* = 8.0 Hz, 2H), 8.14 (d, *J* = 9.0 Hz, 2H), 7.95 (d, *J* = 9.0 Hz, 2H), 7.78 (dd, *J* = 8.0, 7.5 Hz, 2H), 7.77 (dd, *J* = 8.0, 7.5 Hz, 2H), 7.68 (d, *J* = 8.0 Hz, 2H), 7.25 (dd, *J* = 8.0, 7.5 Hz, 2H), 7.05 (dd, *J* = 8.0, 7.5 Hz, 2H), 6.91 (dd, *J* = 8.0, 7.5 Hz, 2H), 6.82 (dd, *J* = 8.0, 7.5 Hz, 2H). ^{13}C NMR (175 MHz, $CDCl_3$): δ 141.3, 141.2, 135.1, 134.1, 133.8, 133.4, 131.9, 131.0, 129.4, 128.7, 128.4, 128.3, 128.1, 127.5, 127.3, 127.1, 125.2, 125.1, 124.6, 123.9, 122.9, 122.1. Some carbon signals are missing due to overlapping signals and/or line broadening. HRMS-MALDI (*m*/*z*): $[M]^+$ calcd. for $C_{50}H_{28}$, 628.2186; found, 628.2199.

A7. Bis(17,17'-tetrabenzo[*a,c,g,i*]fluorenylidene) 3

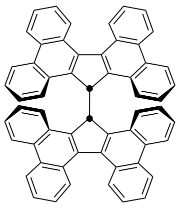

17,17'-Bis(17*H*-tetrabenzo[*a,c,g,i*]fluorenyl) (**9**). Method A: To a solution of 17*H*-tetrabenzo[*a,c,g,i*]fluorene (**6**)[S5] (366.5 mg, 1.00 mmol) in anhydrous THF (35.7 mL) at −78 ºC, a solution of ^{n}BuLi (0.44 mL, 2.5 M in *n*-hexane, 1.10 mmol) was dropwise added. After being stirred at the same temperature for 1 h, the solution was treated with a solution of $TiCl_4$ (1.0 mL, 1.0 M in toluene, 1.00 mmol). The reaction mixture was slowly warmed to room temperature and stirred overnight, the precipitated was isolated by filtration gave the title compound (222.9 mg, 61%) as a bright yellow solid, m. p. >300 ºC. The filtrate contained a mixture of **6** and **9** (around 10–20%), but they were difficult to be separated by chromatography. ^{1}H NMR (500 MHz, CD_2Cl_2, 253 K) δ 9.29 (d, *J* = 8.0 Hz, 2H), 9.11 (d, *J* = 8.0 Hz, 2H), 8.92 (d, *J* = 8.0 Hz, 2H), 8.20 (dd, *J* = 7.5, 7.0 Hz, 2H), 8.01 (d, *J* = 8.0 Hz, 2H), 7.99–7.97 (m, 4H), 7.75 (d, *J* = 7.5 Hz, 2H), 7.68 (dd, *J* = 8.0, 7.5 Hz, 2H), 7.39 (t, *J* = 7.5 Hz, 2H), 7.12–7.09 (m, 4H), 7.02 (t, *J* =

7.5 Hz, 4H), 6.38 (dd, J = 8.0, 7.0 Hz, 2H), 6.23 (d, J = 8.0 Hz, 2H), 6.15 (s, 2H). *Note:* The crystallographic structure of **9** indicated that two TBF fragments are arranged in a face-to-face manner with partial overlapping.[S5] ^{1}H NMR spectrum recorded at low temperature agreed with the structural pattern. ^{13}C NMR (175 MHz, CD_2Cl_2): δ 143.3, 139.7, 139.1, 134.2, 131.5, 130.9, 130.0, 129.5, 129.3, 128.1, 127.8, 127.5, 127.0 (×2), 126.3, 126.0, 125.6, 125.4, 125.3, 125.0, 124.8, 124.3, 124.03, 123.99, 123.4, 123.3, 122.6, 121.8, 52.2. HRMS-FAB (*m*/*z*): [M]$^+$ calcd. for $C_{58}H_{34}$, 730.2655; found, 730.2659.

Method B: Compound **9** (219.3 mg, 60%) was obtained from 8b*H*-tetrabenzo[*a,c,g,i*]fluorene[S5] (366.5 mg, 1.00 mmol).

17,17'-Bis(tetrabenzo[*a,c,g,i*]fluorenylidene) (**3**). A solution of **9** (73.1 mg, 0.10 mmol) in DMF (4 mL) was purged with nitrogen for 10 min, heated to 80 ºC under an atmosphere of N_2, treated with ^{t}BuOK (112.2 mg, 1.00 mmol) and stirred overnight. The reaction mixture was cooled to room temperature, and poured into a hydrochloric acid solution (2N, 4 mL). The aqueous layer was extracted with CH_2Cl_2 (2×20 mL) and the combined organic layer was washed with water (2×20 mL), dried over anhydrous $MgSO_4$. The solvents of the filtrate were removed under reduced pressure. The residue was triturated with diethyl ether (10 mL) and dried in vacuo, giving **3** (17.4 mg, 24%). The ether solution was evaporated to dryness. Chromatography on spherical silica gel (hexane/ether 5:1) yielded **9** (51.1 mg, 70%).

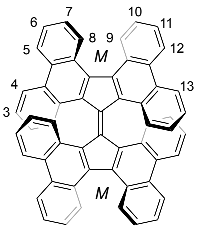

3: A purple solid, mp: >300 ºC. ^{1}H NMR (400 MHz, $CDCl_3$): δ 8.63 (d, J = 8.0 Hz, 4H, 5,12-H), 8.49 (d, J = 8.0 Hz, 4H, 8,9-H), 8.41 (d, J = 8.4 Hz, 4H, 4,13-H), 7.96 (d, J = 8.4 Hz, 4H, 1,16-H), 7.70 (br, 4H, 6,11-H), 7.63 (t, J = 7.6 Hz, 4H, 7,10-H), 7.11 (t, J = 7.6 Hz, 4H, 3,14-H), 6.71 (t, J = 7.6 Hz, 4H, 2,15-H). ^{1}H NMR (500 MHz, $C_2D_2Cl_4$): δ 8.68 (d, ^3J = 7.7 Hz, 4H), 8.53 (d, J = 7.7 Hz, 4H), 8.47 (d, J = 7.7 Hz, 4H), 8.01 (d, J = 7.7 Hz, 4H), 7.76 (br, 4H), 7.71 (t, J = 7.7 Hz, 4H), 7.12 (t, J = 7.7 Hz, 4H), 6.79 (t, J = 7.7 Hz, 4H). Signals of **3** in $CDCl_3$ were not observed in the ^{13}C NMR spectrum although the sample has been scanned 32000 times. HRMS-MALDI (*m*/*z*): [M]$^+$ calcd. for $C_{58}H_{32}$, 728.2499; found, 728.2490.

B. Structural Analysis

Supplementary Table 1. Crystallographic data and structure refinement details

	**1a**	**1b**	**1c**	(*M*,*M*)-**3**
CCDC	2116430	2204920	2204921	2116431
formula	$C_{42}H_{24}$	$C_{78}H_{48}$	$C_{46}H_{32}O_4$•CH_2Cl_2	$C_{58}H_{32}$
formula weight	528.61	985.16	733.64	728.83
temperature (K)	100(2)	100.00(10)	100.00(10)	140(2)
wavelength (Å)	0.71073	1.54184	1.54184	0.71073
crystal system	Monoclinic	Monoclinic	Triclinic	Orthorhombic
space group	C_2/c	$P2_1/n$	P-1	$Aba2$
a (Å)	36.522(4)	11.3142(6)	9.6918(3)	24.2582(8)
b (Å)	8.0589(9)	32.9673(14)	12.9896(3)	18.3254(6)
c (Å)	36.188(4)	14.9172(8)	15.3647(4)	8.0821(3)
α (deg)	90	90	77.234(2)	90
β (deg)	90.881(2)	106.775(5)	77.911(2)	90
γ (deg)	90	90	68.754(2)	90
Volume (Å^3)	10650(2)	5327.3(5)	1740.37(8)	3592.8(2)
Z	16	4	2	4
d_{calc} (g/cm^3)	1.319	1.228	1.400	1.347
F(000)	4416	2064.0	764.0	1520
crystal size (mm)	0.188×0.169×0.138	0.25×0.05×0.01	0.25×0.14×0.03	0.126×0.12×0.028
θ range (deg)	2.231 to 28.302	3.372 to 67.078	3.698 to 72.763	2.223 to 28.308
reflns collected	273551	33105	22904	26780
indep reflns/R_{int}	13223/0.1020	9471/0.0957	6534/0.0322,	4444/0.0407
parameters	757	707	483	263
GOF on F^2	1.030	1.005	1.101	1.066
R_1, wR_2 [I>2σ(I)]	0.0459, 0.0990	0.0845, 0.2176	0.0548, 0.1719	0.0528, 0.1347
R_1, wR_2 (all data)	0.0671, 0.1076	0.1434, 0.2549	0.0599, 0.1754	0.0602, 0.1395

Supplementary Table 2. Structural parameters of studied compounds.[a]

		θ_1 [deg]	θ_2 [deg]	*d* [Å]
**1a**	X-ray	49, 51	10–16	1.402(3), 1.400(2)
S_0-**1a**	DFT	51	13	1.403
**1a**-TS_{rot}		90	0	1.463
T_1-**1a**		90	0	1.462
**1b**	X-ray	58	1, 5	1.431(4)
S_0-**1b**	DFT	52	14	1.413
**1b**-TS_{rot}		90	0	1.466
T_1-**1b**		90	0	1.466
**1c**	X-ray	55	9, 15	1.404(3)
S_0-**1c**	DFT	50	14	1.405
**1c**-TS_{rot}		90	0	1.464
T_1-**1c**		90	0	1.465
S_0-*syn*-**1d**	DFT	51	14	1.409
S_0-*anti*-**1d**		51	14	1.409
**1d**-TS_{rot}		90	0	1.465
T_1-**1d**		90	0	1.466
S_0-*syn*-(*M*,*P*)-**2**	DFT	50	24, 18	1.403
(*M*,*P*)-**2**-TS_{rot}		90	7	1.463
T_1-(*M*,*P*)-**2**		90	7	1.464
S_0-*syn*-(*M*,*M*)-**2**		46	26	1.402
(*M*,*M*)-**2**-TS_{rot}		87	8	1.463
T_1-(*M*,*M*)-**2**		86	8	1.463
S_0-*anti*-(*M*,*P*)-**2**		50	24, 17	1.404
S_0-*anti*-(*M*,*M*)-**2**		46	26	1.402
(*M*,*M*)-**3**	X-ray	36	39	1.395(3)
S_0-(*M*,*M*)-**3**	DFT	37	37	1.397
(*M*,*M*)-**3**-TS_{rot}		82	19	1.462
T_1-(*M*,*M*)-**3**		74	22	1.462
S_0-(*M*,*P*)-**3**	DFT	52	31, 6	1.402
(*M*,*P*)-**3**-TS_{rot}		90	18	1.463
T_1-(*M*,*P*)-**3**		90	18	1.463

[a] Computed structures were obtained by calculating at the level (U)B3LYP-D3/6-31G** of theory.

Supplementary Table 3. Structural parameters obtained by wB97XD/6-31G**

	θ_1 [deg]	θ_2 [deg]	d [Å]
S_0-**1a**	47	16	1.383
**1a**-TS_{rot}	90	0	1.461
T_1-**1a**	90	0	1.462
CS-**1b**	47	17	1.388
OS-**1b** (≡ S_0-**1a**)	55	13	1.420
**1b**-TS_{rot}	90	0	1.463
T_1-**1b**	90	0	1.464
S_0-**1c**	47	16	1.384
**1c**-TS_{rot}	90	0	1.463
T_1-**1c**	90	0	1.463
S_0-(*M*,*M*)-**3**	34	38	1.377
(*M*,*M*)-**3**-TS_{rot}	87	18	1.460
T_1-(*M*,*M*)-**3**	75	22	1.461

At the wB97XD/6-311++G** level of theory, OS-**1b** (OS = open-shell singlet) is more stable than the closed-shell (CS) state ($\Delta E_{CS\text{–}OS} = E_{CS} - E_{OS}$) by 1.21 kcal/mol.

Supplementary Table 4. Structural parameters obtained by CAM-B3LYP/6-31G**

	θ_1 [deg]	θ_2 [deg]	d [Å]
CS-**1a**	50	13	1.386
OS-**1a** (≡ S_0-**1a**)	56	10	1.412
**1a**-TS_{rot}	90	0	1.466
T_1-**1a**	90	0	1.467
CS-**1b**	51	13	1.393
OS-**1b** (≡ S_0-**1b**)	66	7	1.477
**1b**-TS_{rot}	90	0	1.468
T_1-**1b**	90	0	1.469
CS-**1c**	49	13	1.388
OS-**1c** (≡ S_0-**1c**)	57	9	1.420
**1c**-TS_{rot}	90	0	1.467
T_1-**1c**	90	0	1.468
S_0-(*M*,*M*)-**3**	37	35	1.381
T_1-(*M*,*M*)-**3**	77	21	1.467

At the CAM-B3LYP/6-311++G** level of theory, OS-**1a**, OS-**1b**, and OS-**1c** were are more stable than the corresponding CS state by 0.82, 2.41 and 0.98 kcal/mol, respectively.

Supplementary Table 5. Computed properties obtained by DFT calculations.[a]

	method	**1a**	**1b**	**1c**	(*M,M*)-**3**
$\Delta H_{rot}^{\ddagger}$ (kcal/mol)	I	4.64	2.35	4.53	5.13
	II	1.74	0.42	1.34	2.39
	III	0.28	0.00	0.17	—
ΔH_{ST} (kcal/mol)	Exptl.	5.32	3.65	5.36	5.68
	I	5.52	3.19	5.41	5.79
	II	2.74	1.38	2.31	3.17
	III	1.28	0.29	1.14	0.44

[a] Method I = (U)B3LYP-D3/6-311++G**//6-31G**. II = (U)wB97XD/6-311++G**//6-31G**. III = (U)CAM-B3LYP/6-311++G**//6-31G**. At the wB97XD/6-311++G** level of theory, OS-**1b** (OS = open-shell singlet) was determined as the ground state (S_0), which is more stable than the closed-shell (CS) state ($\Delta E_{CS\text{–}OS} = E_{CS} - E_{OS}$) by 1.21 kcal/mol. At the CAM-B3LYP/6-311++G** level of theory, OS-**1a**, OS-**1b**, and OS-**1c** were theoretically determined as the ground states (S_0), and they are more stable than the corresponding CS states by 0.82, 2.41 and 0.98 kcal/mol, respectively.

Molecular Packing

Compound **1a** crystallized in the monoclinic C_2/c space group, and there were two independent molecules (blue and red) in a unit cell (Supplementary Fig. 4a). The molecules of the same color form one-dimensional supramolecular chain structures through the π–π overlapping between the yellow naphthyl moiety of a molecule and the green six-membered ring of the neighboring molecule (Supplementary Fig. 4b and 4c). The distances from the centroid of the green ring to the mean square plane of yellow moiety are 3.49 Å and 3.43 Å for blue and red molecules, respectively. There are short carbon···hydrogen contacts between blue and red molecules with distances of 2.77 Å and 2.79 Å (Supplementary Fig. 4d).

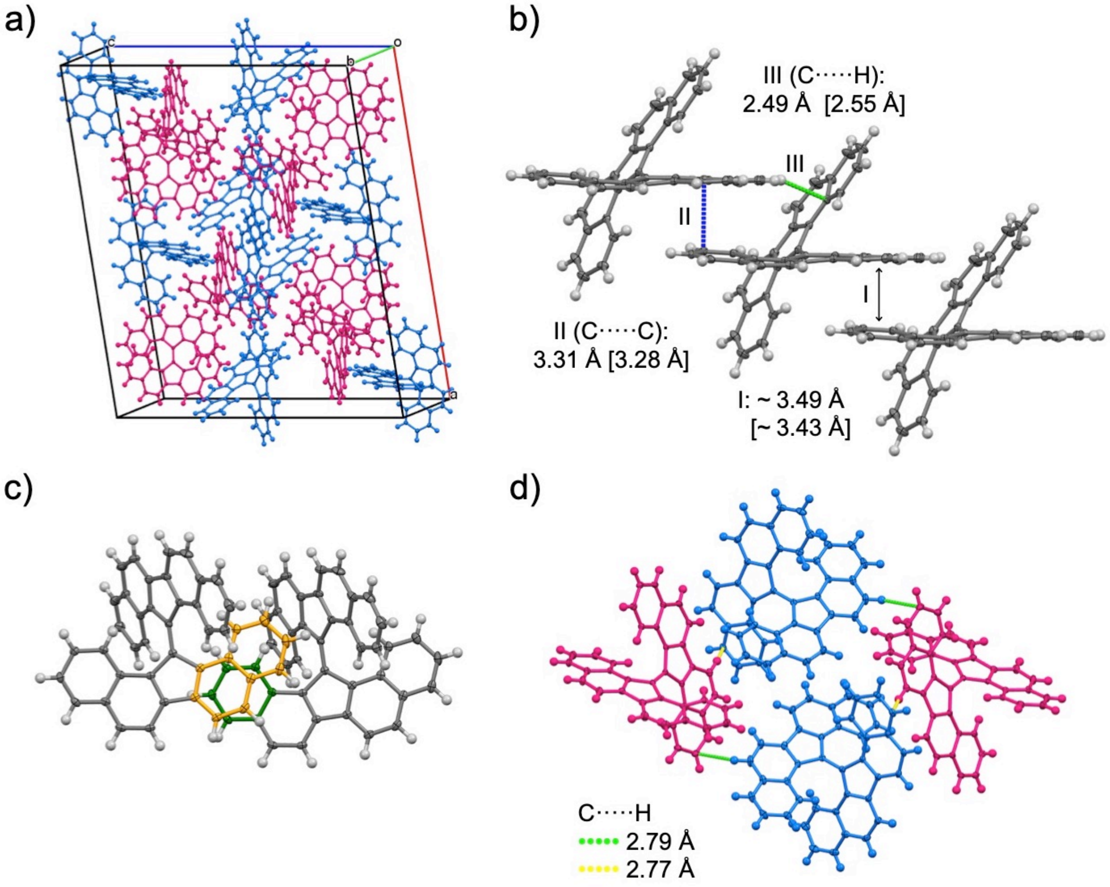

Supplementary Fig. 4: Molecular packing of **1a**. a) Unit cell. b,c) π–π Surface overlap. d) The intermolecular interactions between blue and red molecules. The structural data for red molecules are listed in square brackets.

Compound **1b** crystallized in the monoclinic $P2_1/n$ space group. Supplementary Fig. 5 presents the intermolecular interactions of the concerned molecule (red) with its two neighboring molecules (gray). There are numerous short carbon···carbon contacts [<3.390(6) Å] in these molecules. Effective π···π surface overlapping mainly exists between a indenyl moiety of a molecule and an alkynyl group of the neighbor, and vice versa. In each molecular half-fragment, there are two sets of such π···π surface overlapping with the distances of 3.36 Å and 3.48 Å, gauging by the separation of the centroid of an alkynyl group to the mean square plane of an indenyl moiety.

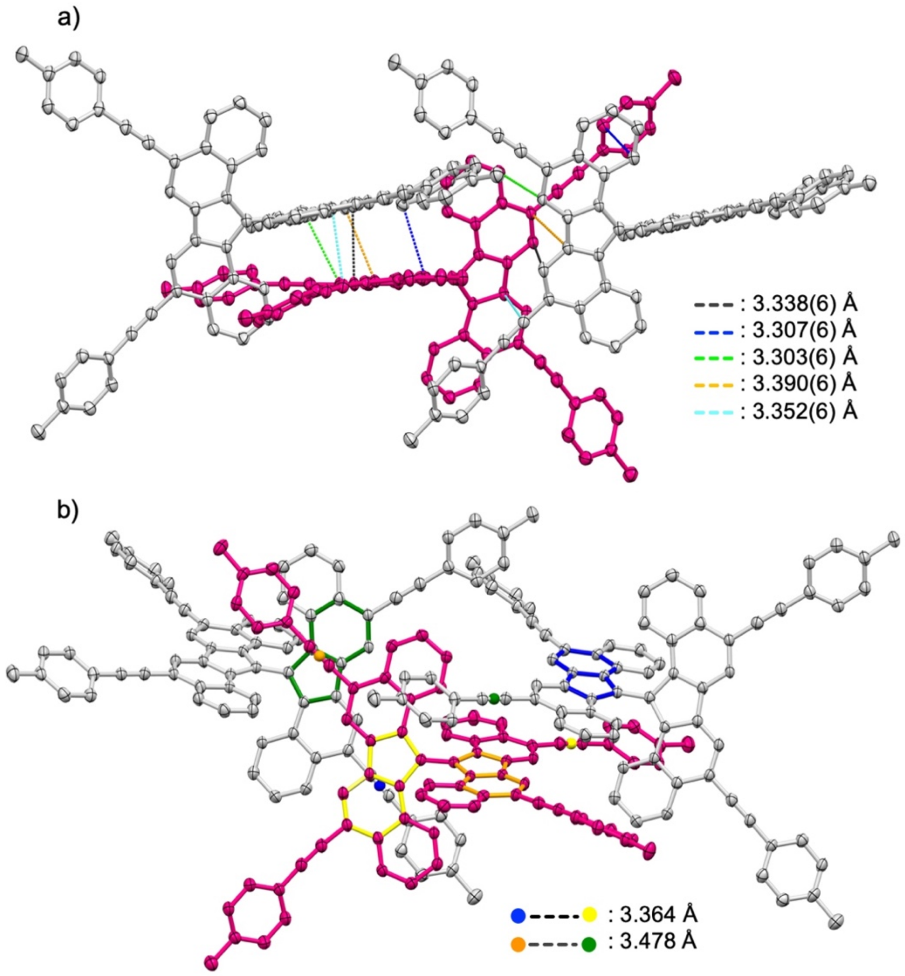

Supplementary Fig. 5: Molecular packing of **1b**. a) The intermolecular interactions of a molecule with its two neighboring molecules. b) The π···π surface overlapping between indenyl moieties and alkynyl groups. Hydrogen atoms are omitted for clarity.

Compound **1c** crystallized in the triclinic *P*-1 space group, and a unit cell contains two **1c** molecules and two dichloromethane molecules. Supplementary Fig. 6 presents the intermolecular interactions of the concerned molecule m^A with its three neighboring molecules m^B, m^C and m^D. Several short nonbonded carbon···carbon contacts [3.375(5) Å and 3.405(4) Å for m^A/m^C; 3.393(4) Å and 3.445(4) Å for m^A/m^D; 3.436(5) Å for m^A/m^B] were observed in these molecules. The π···π surface overlapping was observed for two naphthyl moieties of m^A and m^B (the blue and orange moieties in Supplementary Fig. 8c), and the interplanar distance is 3.47 Å.

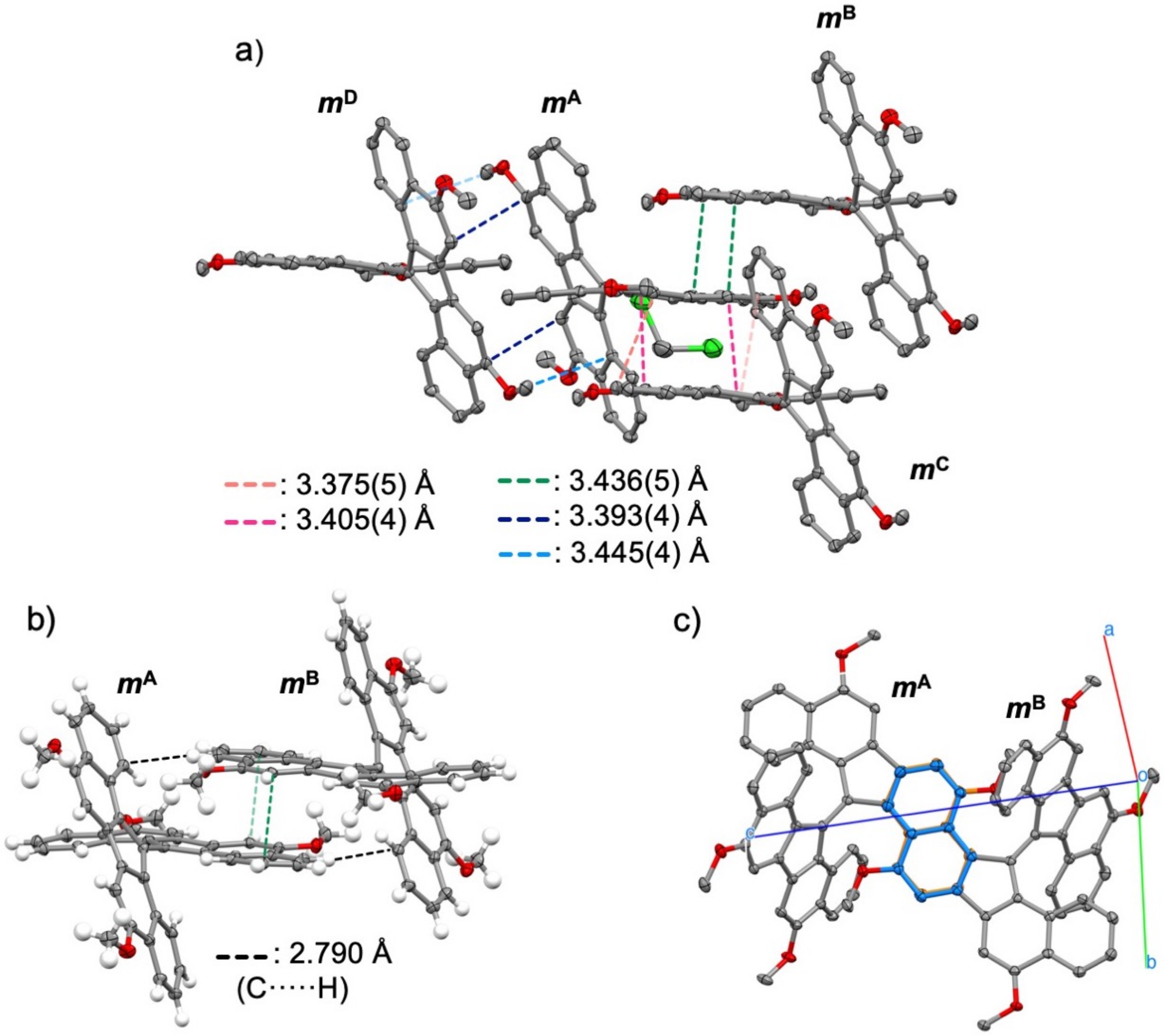

Supplementary Fig. 6: Molecular packing of **1c**. a) Intermolecular interactions of m^A with m^B, m^C and m^D. b, c) The π–π surface overlapping between m^A and m^B. Hydrogen atoms in a) and c) are omitted for clarity.

Compound **3** crystallized in the orthorhombic *Aba*2 space group. Homochiral molecules of **3** aggregate along the *b* and *c* axes (Supplementary Fig. 7). In these homochiral molecules, there are several carbon···carbon contacts (3.33 Å for the blue dashed lines) and carbon···hydrogen contacts (2.84 Å and 2.83 Å for the magenta and green dashed lines, respectively). The intermolecular interactions between heterochiral molecules are mainly the short carbon···hydrogen contacts with a distance of 2.71 Å (yellow dashed lines).

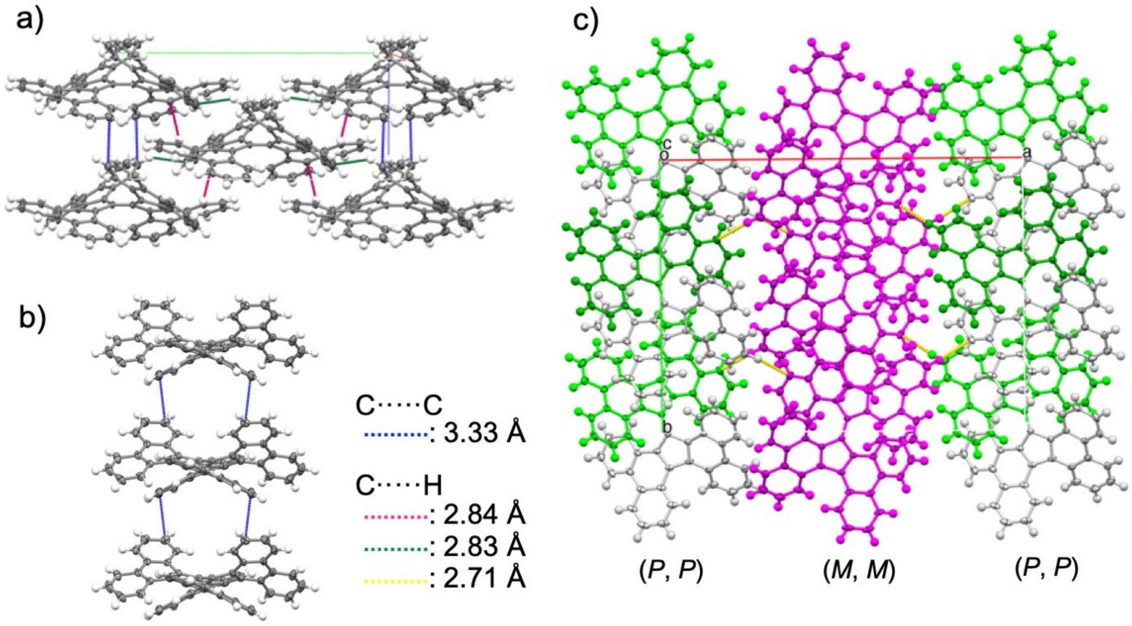

Supplementary Fig. 7: Molecular packing of **3**. a,b) The cluster of homochiral molecules. c) The intermolecular interactions between heterochiral molecules.

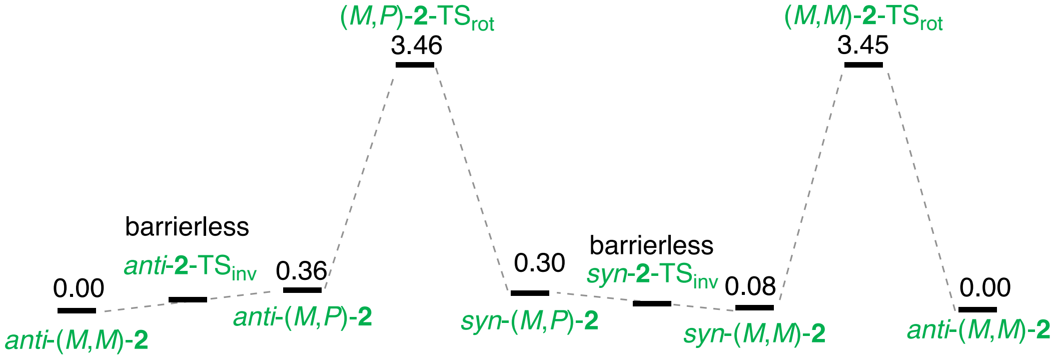

Supplementary Fig. 8: A plausible inversion process for compound **2** (in kcal/mol).

The C=C bond in computed S_0-(*M*,*M*)-**3** (d = 1.397 Å, θ_1 = 37º) is less twisted than that in S_0-(*M*,*P*)-**3** (d = 1.402 Å, θ_1 = 52º), and the (*M*)-TBF fragments (θ_2 = ~35º, θ_3 = 46º) in the two diastereomers were more distorted than the (*P*)-TBF moiety (θ_2 = 6º, θ_3 = 37º), indicating a quasi-planar DBF fragment (θ_2 < 10º) in the latter buttresses a highly twisted C=C bond. S_0-(*M*,*M*)-**3** is more stable than S_0-(*M*,*P*)-**3** by 2.76 kcal/mol, and the conversion from the former to the latter by inverting the helical scaffold required around 13.36 kcal/mol ($\Delta H^{\ddagger}$, see Supplementary Fig. 9). S_0-(*M*,*M*)-**3** is the representative diastereomer in this study, and its heights of $\Delta H^{\ddagger}_{rot}$ (5.13 kcal/mol) and ΔH_{ST} (5.79 kcal/mol) exceeded those of (*M*,*P*)-**3** (3.51 and 2.65 kcal/mol, respectively). This result indicates that the π-extended backbone may stabilize **3**-TS$_{rot}$, and T_1-**3**, but the overcrowded periphery decreases the strain of the ground state.

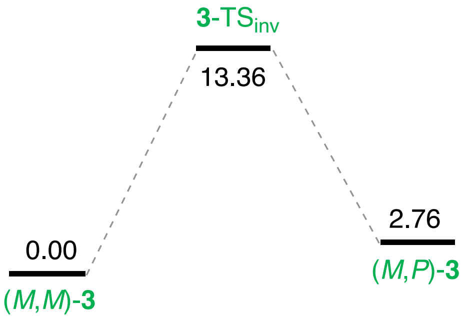

Supplementary Fig. 9: A plausible inversion process for compound **3** (in kcal/mol).

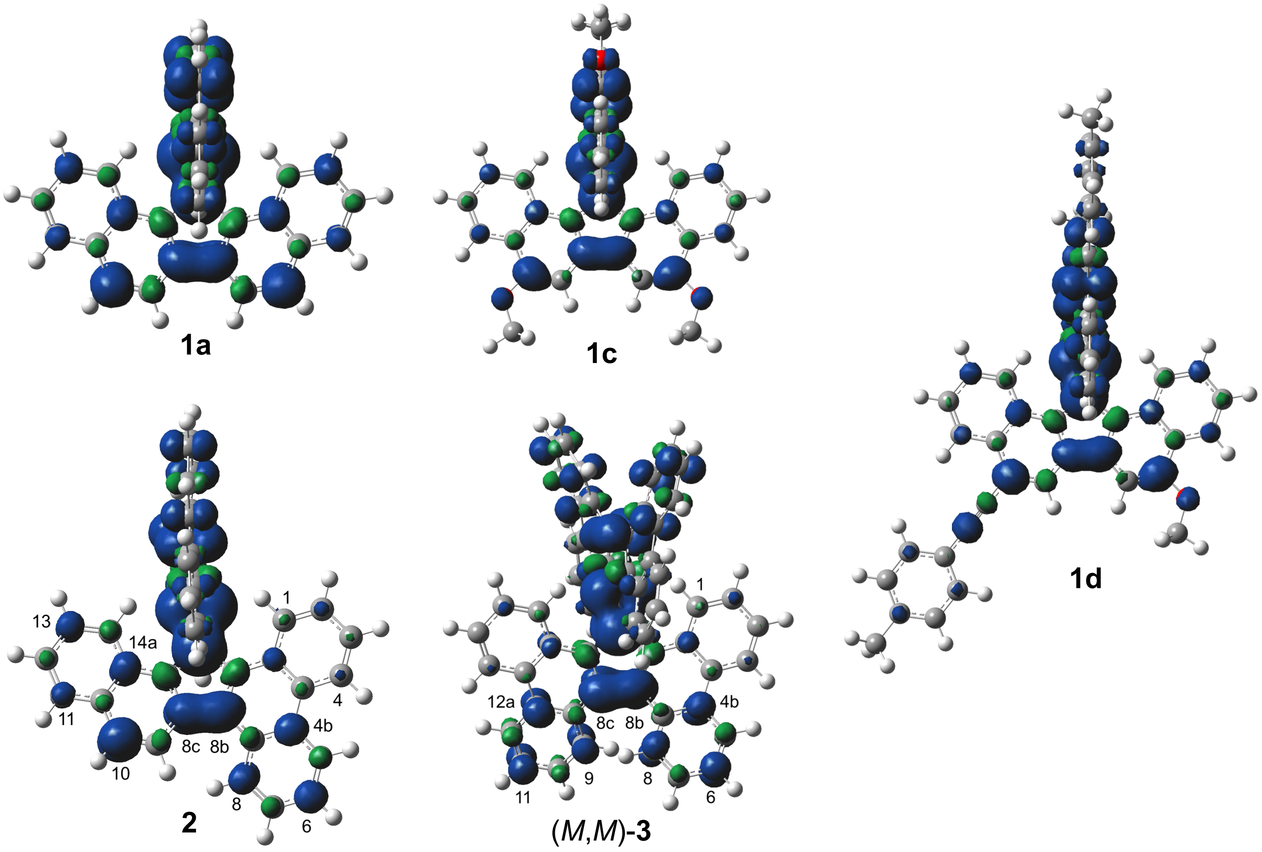

Supplementary Fig. 10: Spin density plots of the T_1 state (Isovalue = 0.02; density = 0.002).

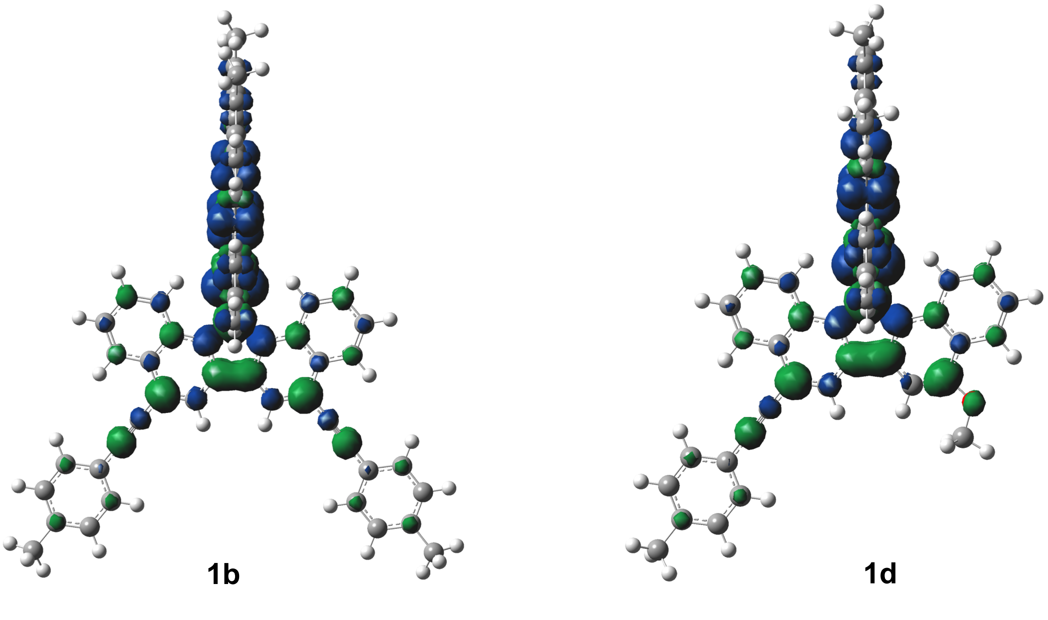

Supplementary Fig. 11: Spin density plots of the transition state of the rotation process (Isovalue = 0.02; density = 0.002).

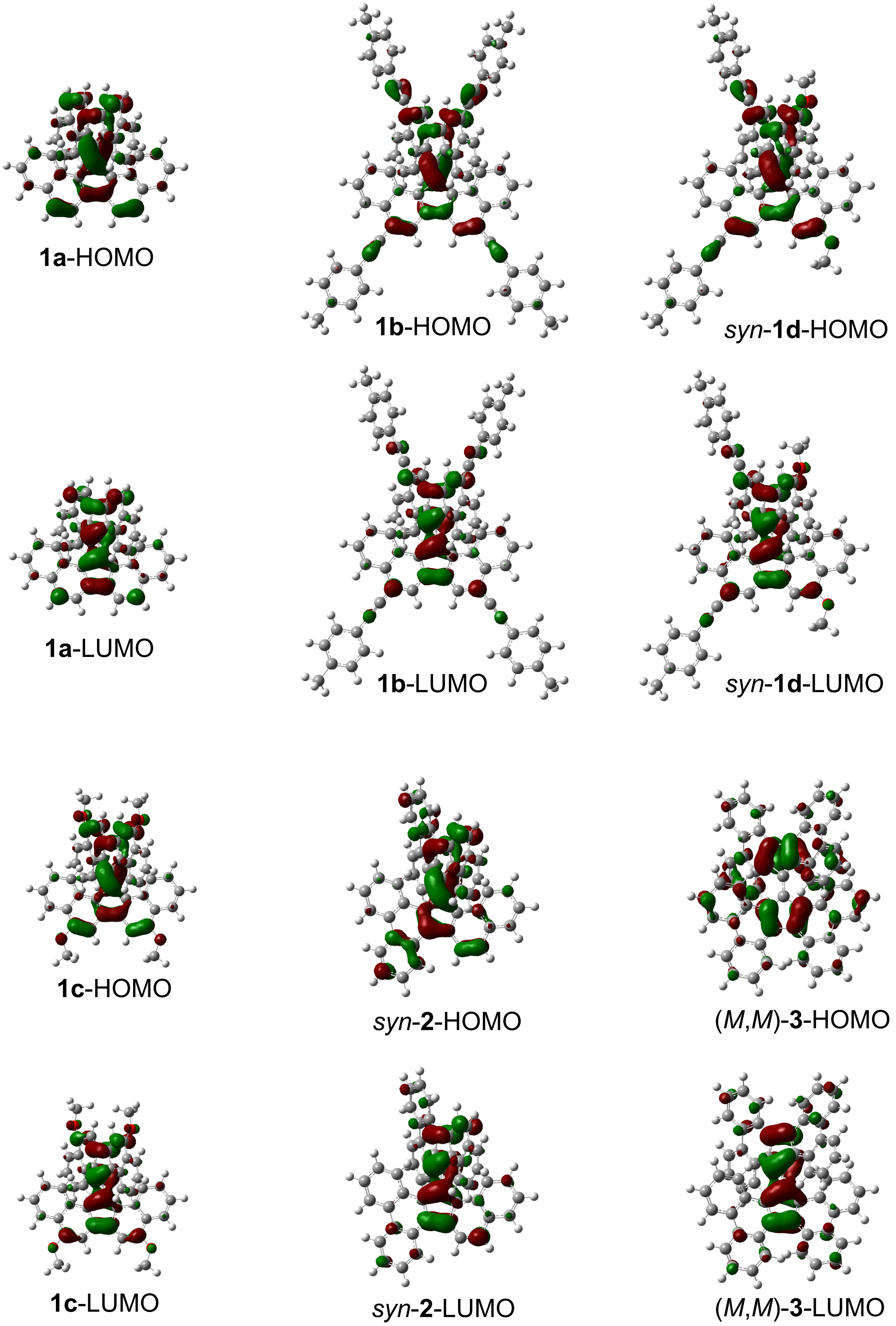

Supplementary Fig. 12: Frontier orbitals.

C. Photophysical Properties

Setup of light source and transient absorption spectroscopy. The detailed schematic setups of our light source and transient absorption spectroscopy are illustrated in our previous study[S6]. The details are summarized as follows. The measurements were performed using a commercial Yb:KGW laser system (Pharos, Light Conversion) with a central wavelength of 1030 nm, an average power of 2.5 W, a repetition rate of 3.125 kHz, a pulse energy of 800 μJ and a pulse duration of 190 fs. Two identical pulses were produced with a low-GDD 50/50 beam splitter and passed through our designed nonlinear compressor using a previously reported technique, namely, multiple plate compression (MPC)[S7,S8]. For this experiment, pulse compression was achieved with 8 chirped mirror bounces (Ultrafast Innovation), thus removing the material dispersion introduced by the optics before the sample. The pump pulse was modulated by a laser-triggered mechanical chopper modulating at half the laser repetition rate (1.5625 kHz). A broadband half-wave plate and wire-grid polarizer were used to precisely control the excitation power and ensure that no nonlinear effects were introduced. The delay time (relative to the pump pulse) of the probe pulse was adjusted by a linear translation stage (DL325, Newport) that supports a delay range up to ~ 2.2 ns. The pump and probe beams were both focused on the sample in a noncollinear manner with a cross-angle of 5 degrees. Different focusing conditions were chosen for pump and probe pulses to ensure that the focused pump spot size (~67.7 μm) was slightly larger than the focused probe spot size (~27.3 μm) and that the probed region was uniformly excited. After the pulses passed through the sample, the transmitted probe pulse was spatially separated and guided into our designed spectrometer, which includes a high-speed linear array camera (Glaz Linescan-I-Gen2, Synertronic with S12198-512Q CMOS, Hamamatsu) to ensure that each probe pulse is captured. Since the pump pulses are modulated at half of the repetition rate, the spectral difference between every two probe shots (one sees the pump, while the other does not) provides the $\Delta T/T$ signal.

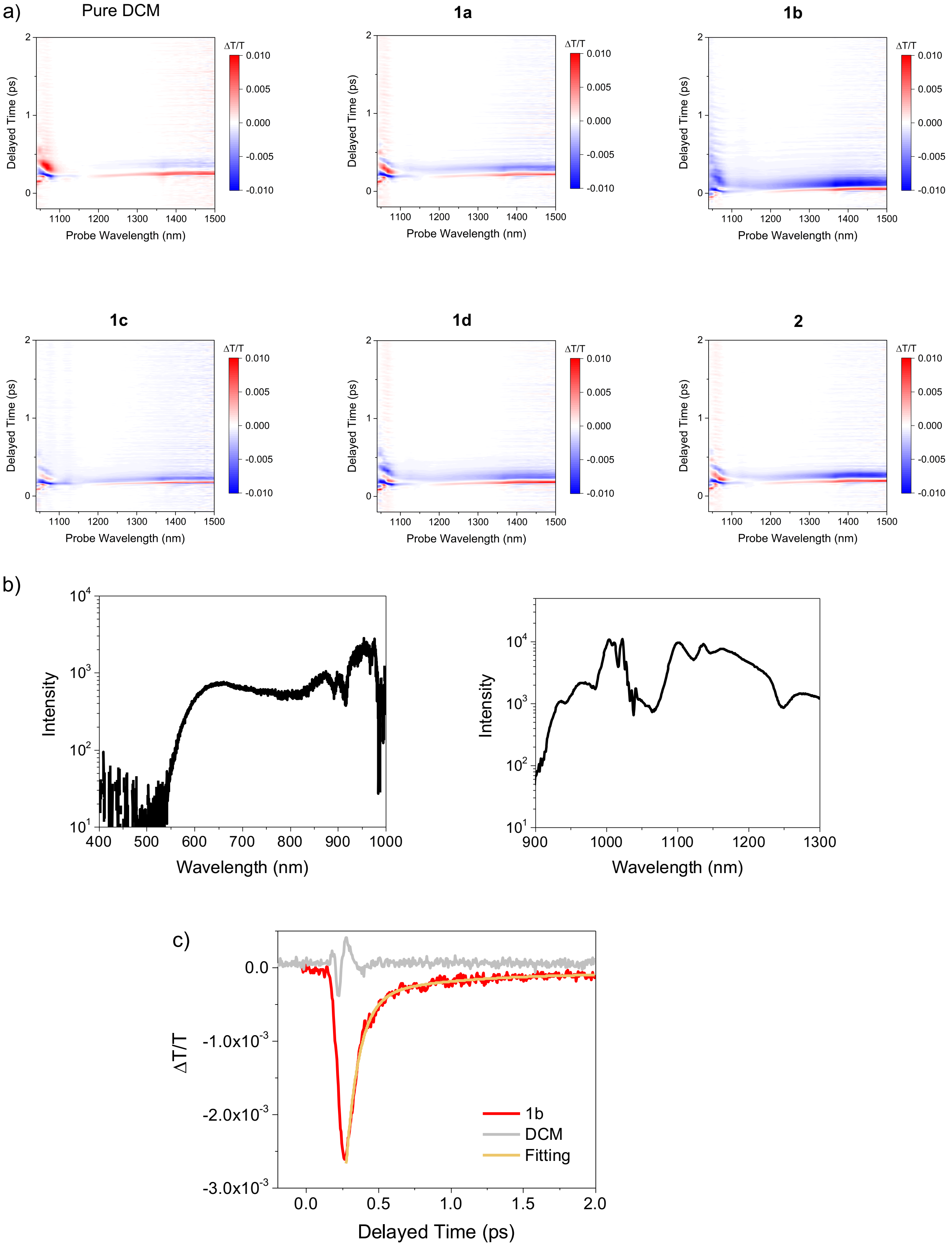

Supplementary Fig. 13: a) Visible-pump NIR-probe TA spectra at room temperature. b) **1b**'s spectra of the pump pulse (left) and probe pulse (right) applied in the Visible-pump NIR-probe TA spectroscopy. c) Time trace of the TA spectra of **1b**. The time traces are obtained by averaging the ones at 1100–1200 nm. The lifetimes of 90 fs and 618 fs are acquired by fitting the bi-exponential function.

D. Electrochemical Properties

Supplementary Table 6. Electrochemical properties

		$E_{½}^{ox}$ (V)	$E_{½}^{red}$ (V)	E_g^{EC} (eV)
**1a**	CV	0.53^{irr}	−1.06, −1.36	1.59
	DPV	0.63	−1.05, −1.35	1.68
**1b**	CV	0.45^{qr}, 0.61^{qr}	−0.81, −1.02	1.26
	DPV	0.53, 0.66	−0.79, −1.00	1.32
**1c**	CV	0.15, 0.26	−1.31, −1.54	1.46
	DPV	0.18, 0.25	−1.29, −1.52	1.47
**1d**	CV	0.33	−1.05, −1.26	1.38
	DPV	0.35	−1.03, −1.24	1.38
**2**	CV	0.56^{irr}	−0.93, −1.20	1.49
	DPV	0.65	−0.91, −1.18	1.56
**3**	CV	0.65^{irr}	−0.94, −1.23	1.59
	DPV	0.63	−0.93, −1.21	1.56

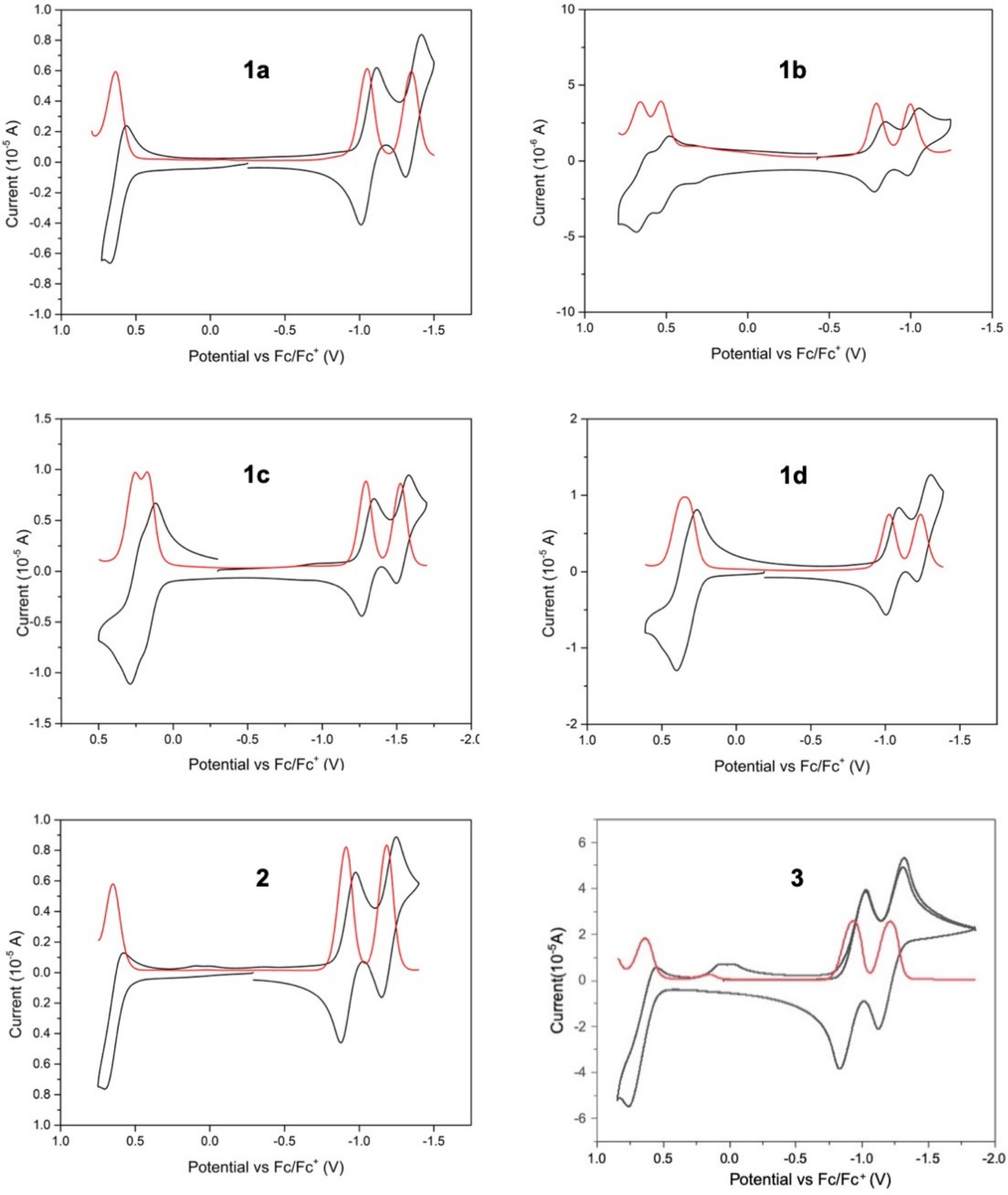

Supplementary Fig. 14: Cyclic voltammograms and differential pulse voltammogram of studied compounds.

E. Magnetic Properties
E1. SQUID

1a@5000G
diamagnetic susceptibility = –332.4×10$^{–6}$ cm^3 mol$^{–1}$
–2J = –1862 cm$^{–1}$ = 5.32 kcal/mol
TIP = 2.27×10$^{–3}$ cm^3/mol, R^2 = 0.9987

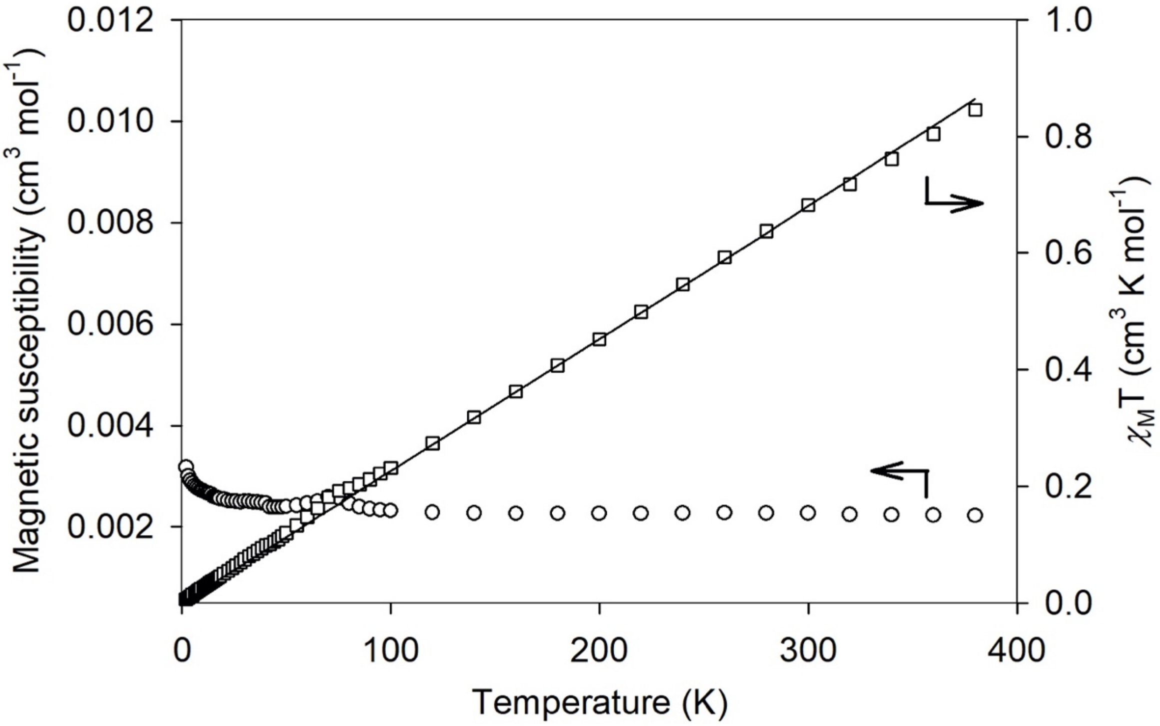

1b@ 5000G
diamagnetic susceptibility = –626.4×10$^{–6}$ cm^3 mol$^{–1}$
–2J = –1276 cm$^{–1}$ = 3.65 kcal/mol
TIP=2.11×10$^{–3}$ cm^3/mol, R^2= 0.9777

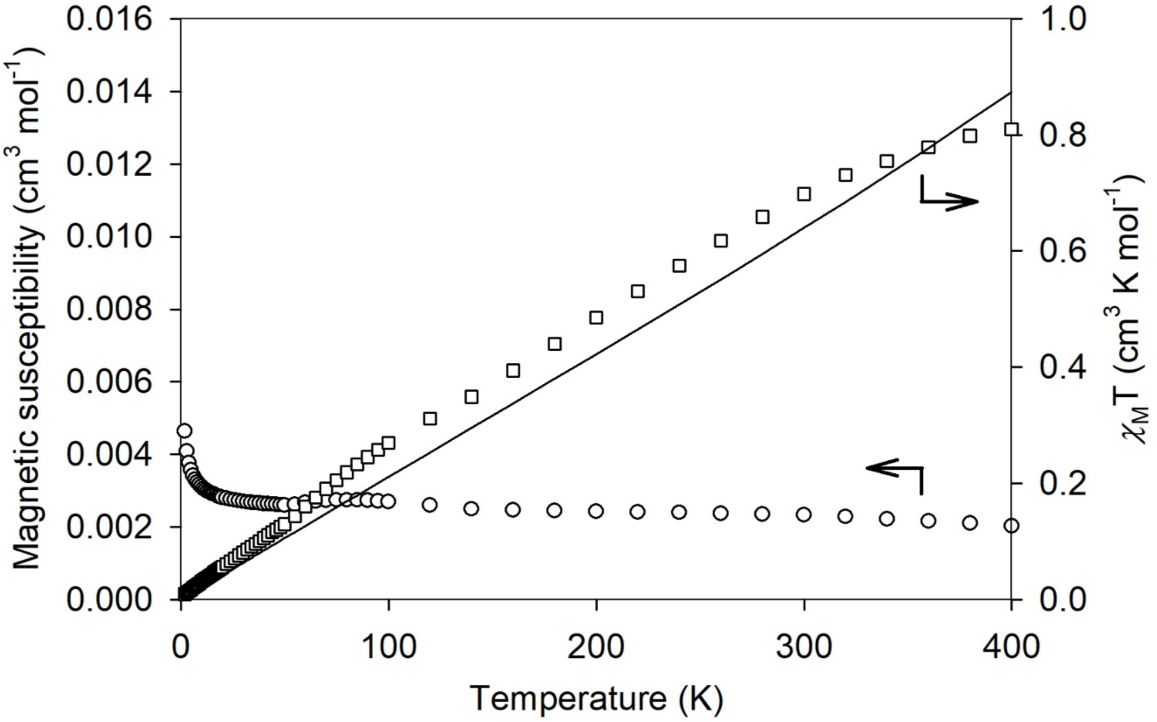

1c@ 5000G
diamagnetic susceptibility = –398.24 ×10^{-6} cm^3 mol^{-1}
–2J = –1876 cm^{-1} = 5.36 kcal/mol
TIP=5.52×10^{-4} cm^3/mol, R^2 = 0.9995

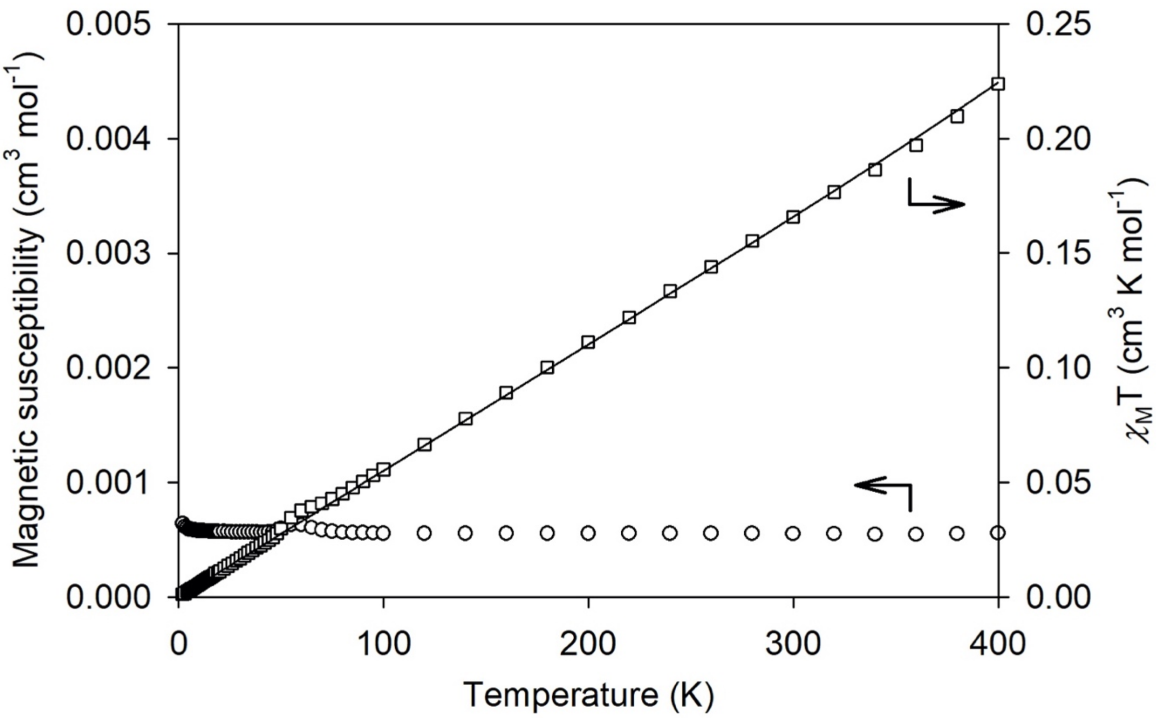

2@2000G
diamagnetic susceptibility = –394.04×10^{-6} cm^3 mol^{-1}
–2J = –1510 cm^{-1} = 4.32 kcal/mol
TIP = 2.53×10^{-3} cm^3/mol, R^2 = 0.9987

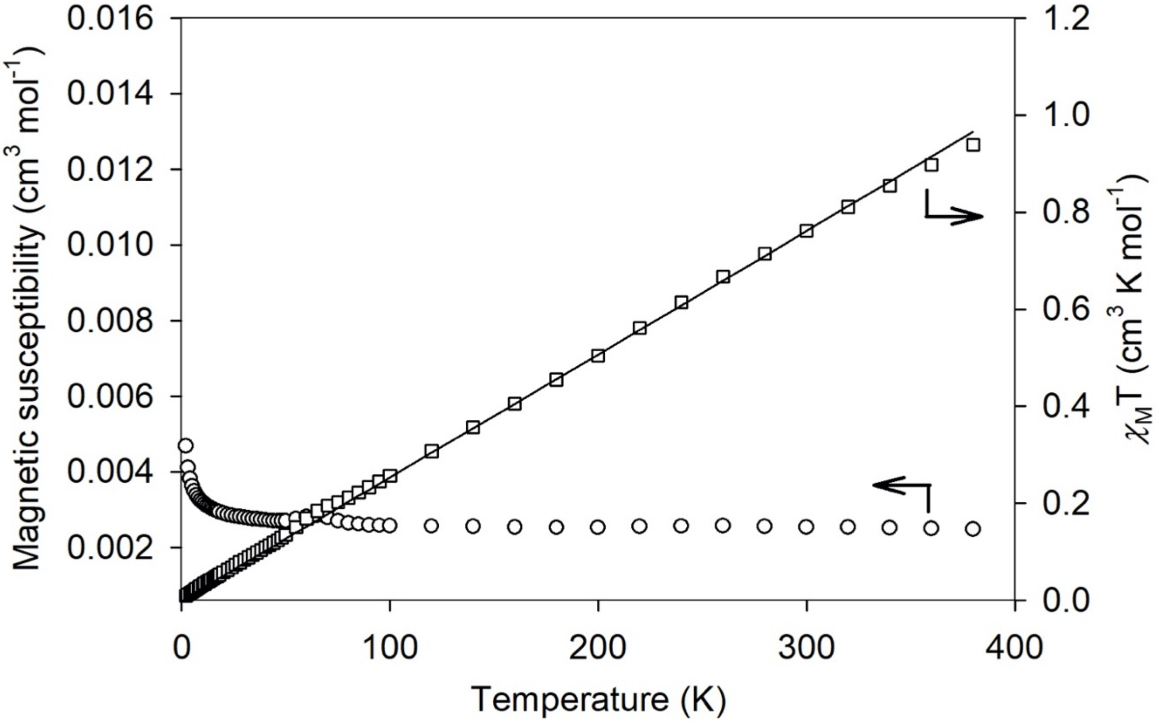

3@2000G
diamagnetic susceptibility = –455.68×10^{-6} cm^3 mol^{-1}
–2J = –1988 cm^{-1} = 5.68 kcal/mol
TIP = 2.26×10^{-3} cm^3/mol, R^2 = 0.9975

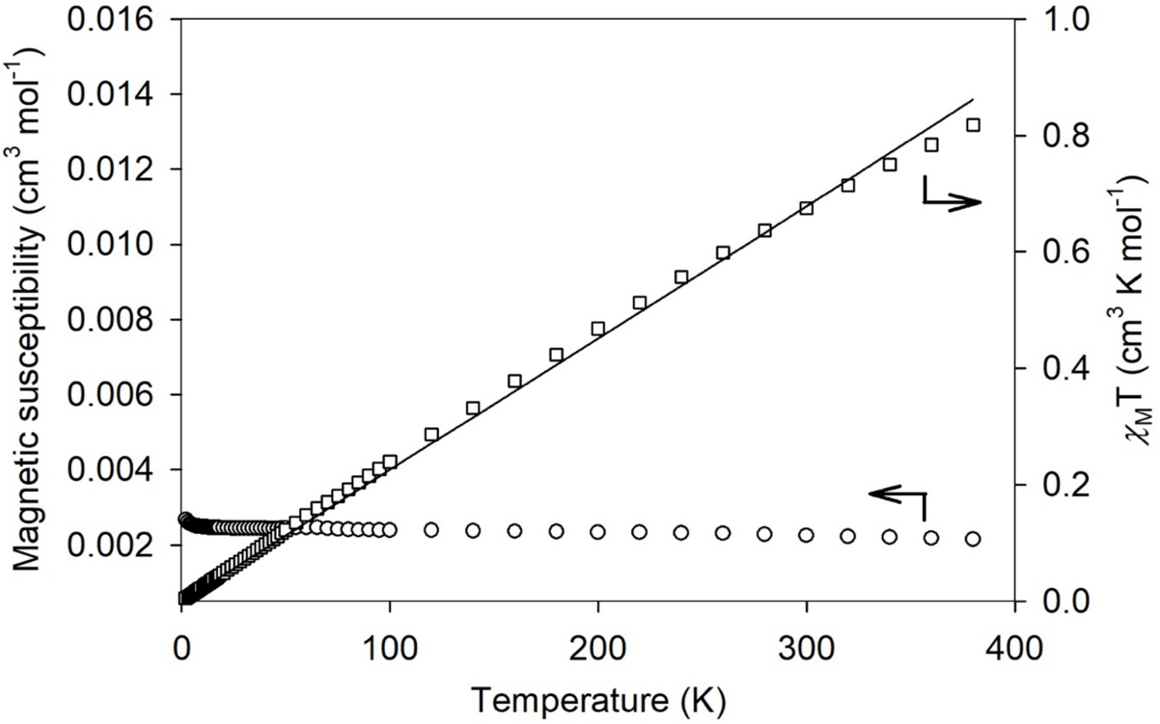

Supplementary Fig. 15: Temperature-dependent magnetic susceptibility and $\chi_M T$ plots. The solid lines are the satisfactory to the theoretical equation.

E2. ESR Spectra

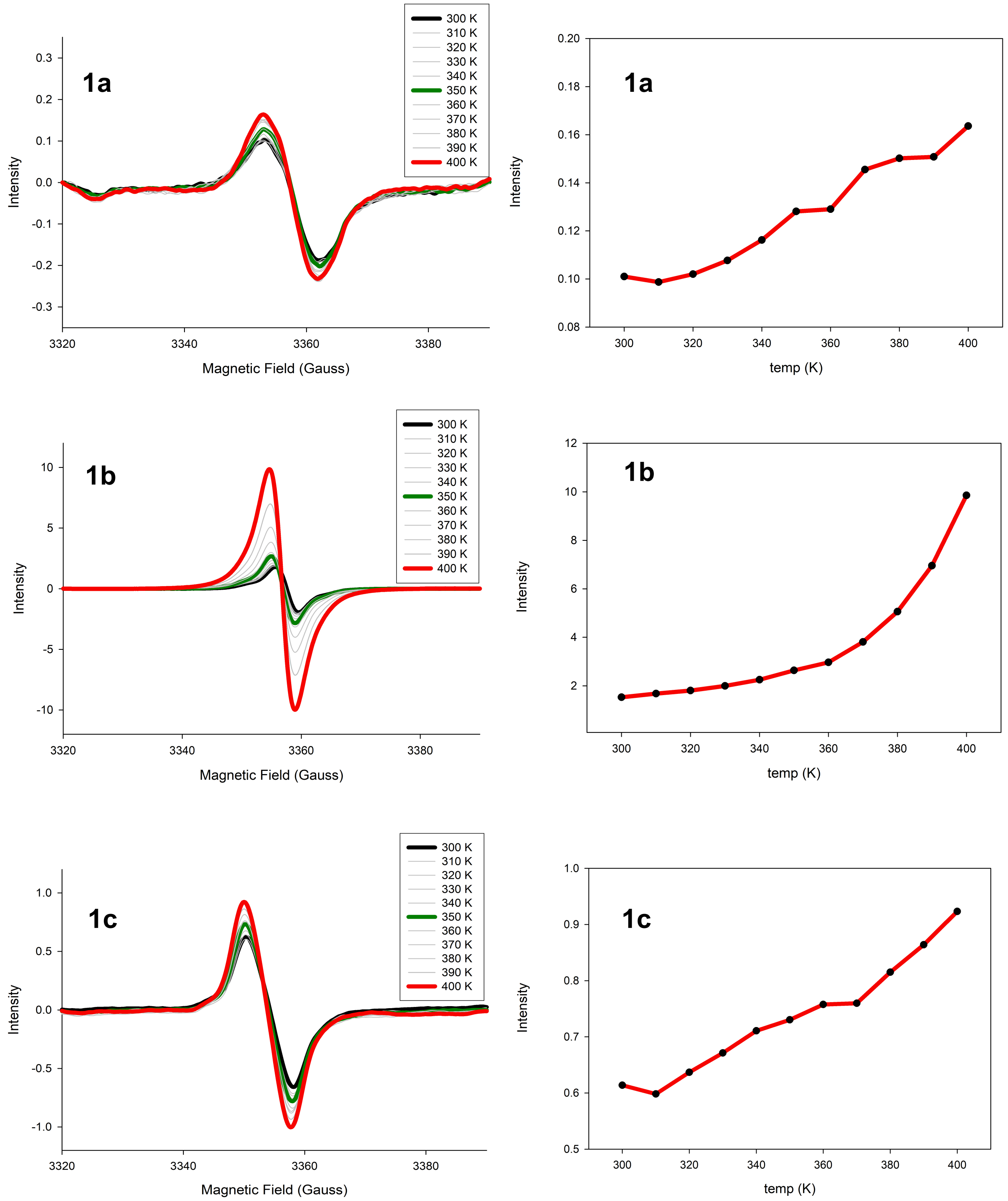

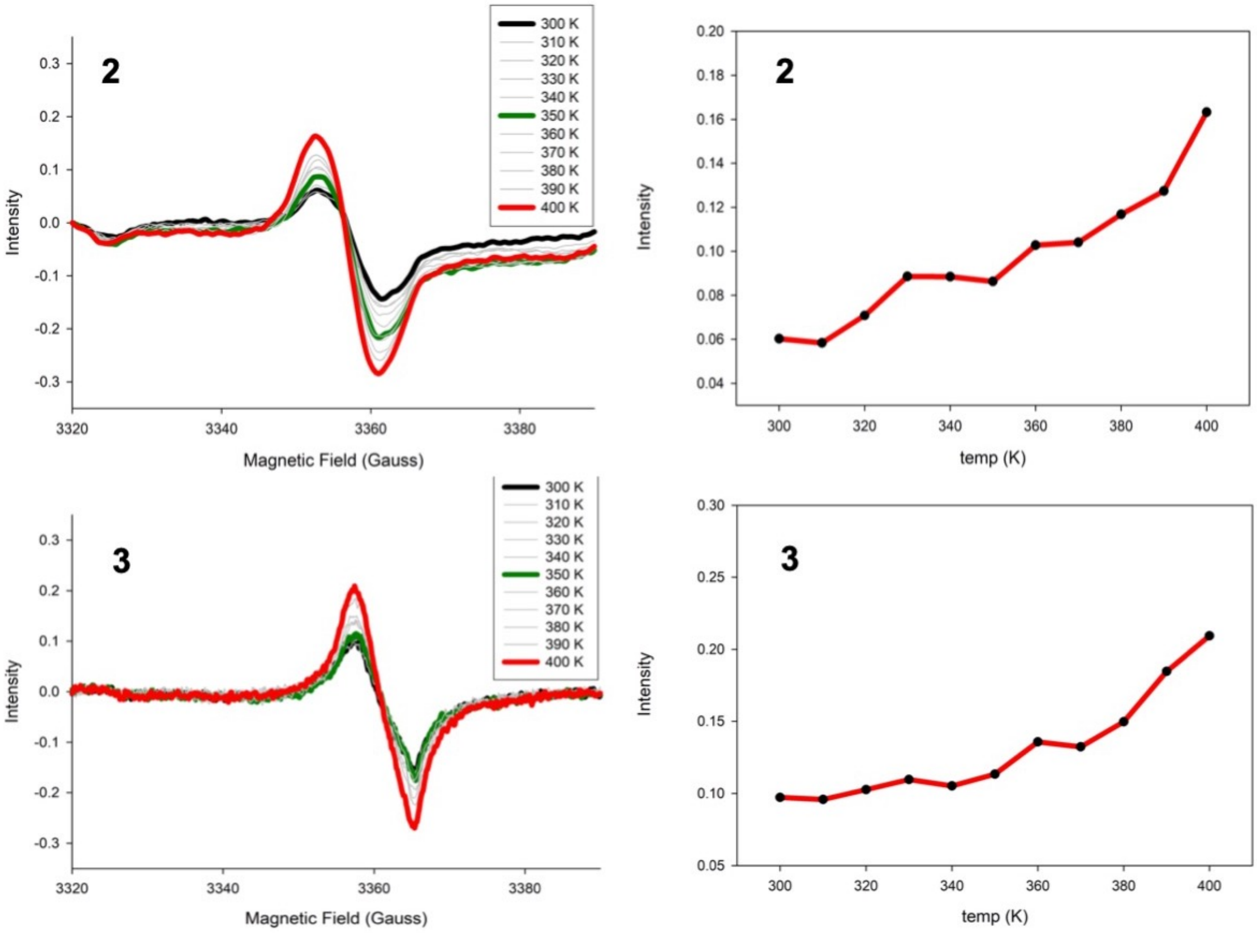

Supplementary Fig. 16: VT ESR spectra of the powdered samples and the plot of temperature-dependent signal intensity against temperature.

F. Computational Details

B3LYP-D3/6-311++G**//6-31G**	E (SCF)	H_{corr}	H_{total} (hartree)
S₀-**1a**	-1615.4141	0.5505	-1614.8636
T_1-**1a**	-1615.4035	0.5487	-1614.8548
**1a**-TS_{rot}	-1615.4039	0.5477	-1614.8562
S_0-**1b**	-3001.9318	1.0583	-3000.8735
T_1-**1b**	-3001.9249	1.0566	-3000.8683
**1b**-TS_{rot}	-3001.9252	1.0555	-3000.8697
S_0-**1c**	-2073.6625	0.6921	-2072.9704
T_1-**1c**	-2073.6523	0.6906	-2072.9617
**1c**-TS_{rot}	-2073.6526	0.6895	-2072.9631
S_0-*anti*-**1d**	-2537.7978	0.8753	-2536.9225
S_0-*syn*-**1d**	-2537.7979	0.8753	-2536.9226
T_1-**1d**	-2537.7894	0.8736	-2536.9158
**1d**-TS_{rot}	-2537.7898	0.8726	-2536.9172
S_0-*anti*-(*M,M*)-**2**	-1922.7728	0.6502	-1922.1226
S_0-*syn*-(*M,M*)-**2**	-1922.7726	0.6502	-1922.1224
T_1-(*M,M*)-**2**	-1922.7641	0.6484	-1922.1157
(*M,M*)-**2**-TS_{rot}	-1922.7644	0.6473	-1922.1171
S_0-*anti*-(*M,P*)-**2**	-1922.7721	0.6502	-1922.1219
S_0-*syn*-(*M,P*)-**2**	-1922.7722	0.6502	-1922.1220
T_1-(*M,P*)-**2**	-1922.7640	0.6484	-1922.1156
(*M,P*)-**2**-TS_{rot}	-1922.7644	0.6473	-1922.1171
anti-**2**-TS_{inv}	-1922.7718	0.6489	-1922.1229
syn-**2**-TS_{inv}	-1922.7720	0.6489	-1922.1231
S_0-(*M,M*)-**3**	-2230.1293	0.7493	-2229.3800
T_1-(*M,M*)-**3**	-2230.1181	0.7473	-2229.3708
(*M,M*)-**3**-TS_{rot}	-2230.1182	0.7463	-2229.3719
S_0-(*M,P*)-**3**	-2230.1248	0.7492	-2229.3756
T_1-(*M,P*)-**3**	-2230.1173	0.7473	-2229.3700
(*M,P*)-**3**-TS_{rot}	-2230.1177	0.7463	-2229.3714
**3**-TS_{inv}	-2230.1066	0.7478	-2229.3588
**7a** (θ = 75°)	-1616.6616	0.5749	-1616.0867
**7a** (θ = 180°)	-1616.6492	0.5746	-1616.0746
**7a**-TS_{rot} (θ = 0°)	-1616.6287	0.5728	-1616.0559
**7a**-TS_{roc} (θ = 120°)	-1616.6418	0.5736	-1616.0682

*ω*B97XD/6-311++G**//6-31G**			
	E (SCF)	H_{corr}	Htotal (hartree)
S_0-**1a**	-1614.7664	0.5564	-1614.2100
T_1-**1a**	-1614.7597	0.5540	-1614.2057
**1a**-TS_{rot}	-1614.7602	0.5530	-1614.2073
CS-**1b**	-3000.7105	1.0697	-2999.6408
OS-**1b** (≡ S_0-**1b**)	-3000.7107	1.0680	-2999.6427
T_1-**1b**	-3000.7071	1.0666	-2999.6405
**1b**-TS_{rot}	-3000.7076	1.0656	-2999.6420
S_0-**1c**	-2072.8550	0.6998	-2072.1552
T_1-**1c**	-2072.8490	0.6975	-2072.1515
**1c**-TS_{rot}	-2072.8495	0.6964	-2072.1531
S_0-(*M,M*)-**3**	-2229.2436	0.7581	-2228.4855
T_1-(*M,M*)-**3**	-2229.2357	0.7552	-2228.4804
(*M,M*)-**3**-TS_{rot}	-2229.2358	0.7541	-2228.4817

CAM-B3LYP/6-311++G**//6-31G**			
	E(SCF)	H_{corr}	H_{total} (hartree)
CS-**1a**	-1614.3903	0.5570	-1613.8333
OS-**1a** (≡ S_0-**1a**)	-1614.3903	0.5557	-1613.8346
T_1-**1a**	-1614.3871	0.5545	-1613.8326
**1a**-TS_{rot}	-1614.3876	0.5534	-1613.8342
CS-**1b**	-3000.0293	1.0709	-2998.9583
OS-**1b** (≡ S_0-**1b**)	-3000.0308	1.0686	-2998.9622
T_1-**1b**	-3000.0297	1.0680	-2998.9617
**1b**-TS_{rot}	-3000.0302	1.0670	-2998.9632
CS-**1c**	-2072.4418	0.7002	-2071.7416
OS-**1c** (≡ S_0-**1c**)	-2072.4421	0.6988	-2071.7432
T_1-**1c**	-2072.4393	0.6979	-2071.7414
**1c**-TS_{rot}	-2072.4398	0.6969	-2071.7429
S_0-(*M,M*)-**3**	-2228.7199	0.7585	-2227.9615
T_1-(*M,M*)-**3**	-2228.7164	0.7557	-2227.9608

G. NMR spectra

^{1}H (400 MHz) and ^{13}C (125 MHz) NMR spectrum of **1a** in $CDCl_3$

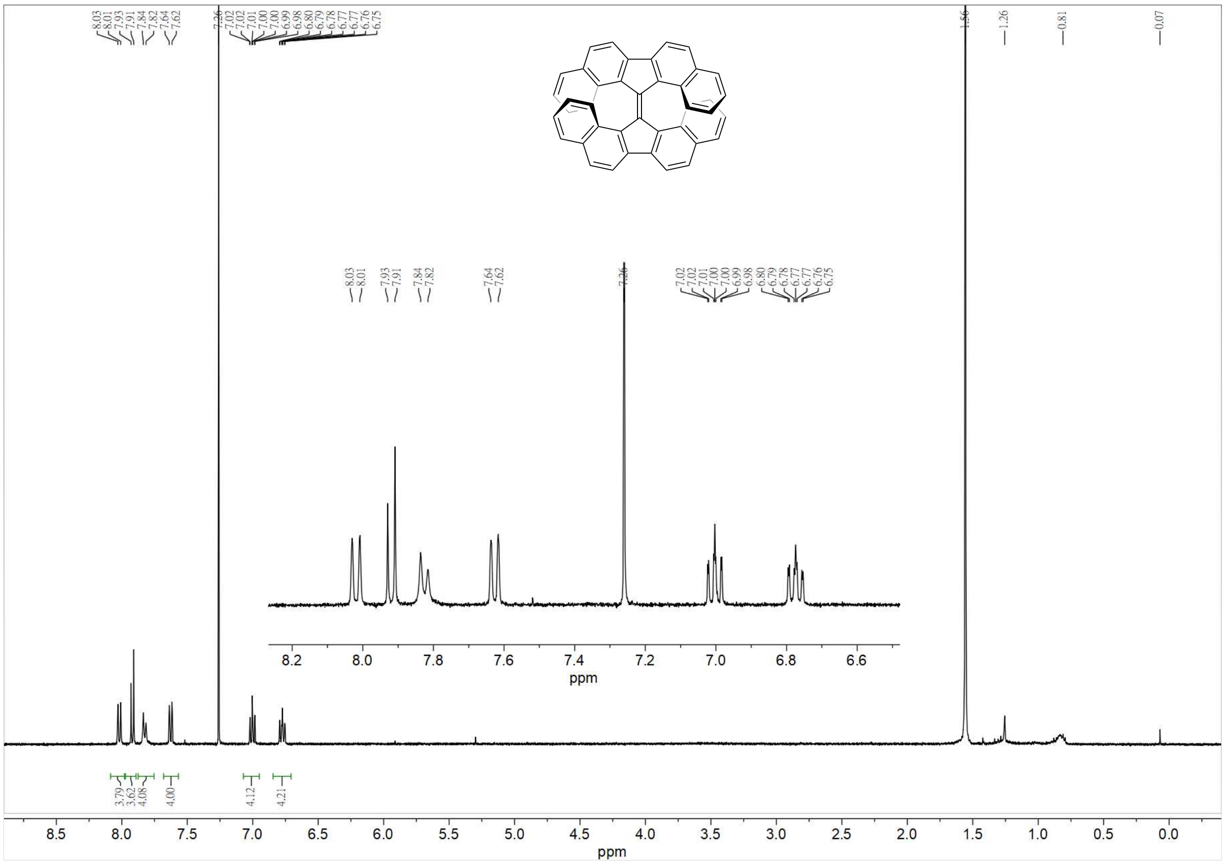

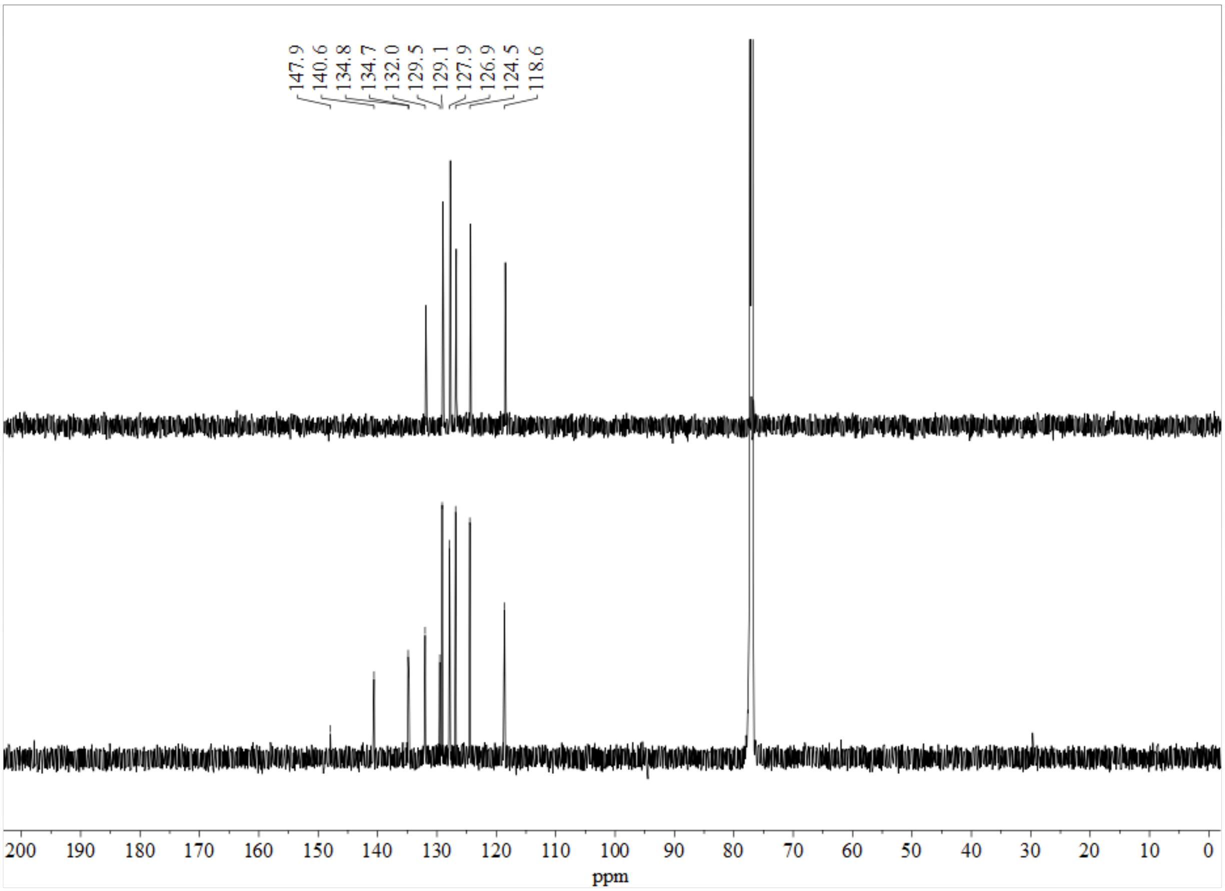

^{1}H (500 MHz) and ^{13}C (125 MHz) NMR spectra of **1b** in $CDCl_3$

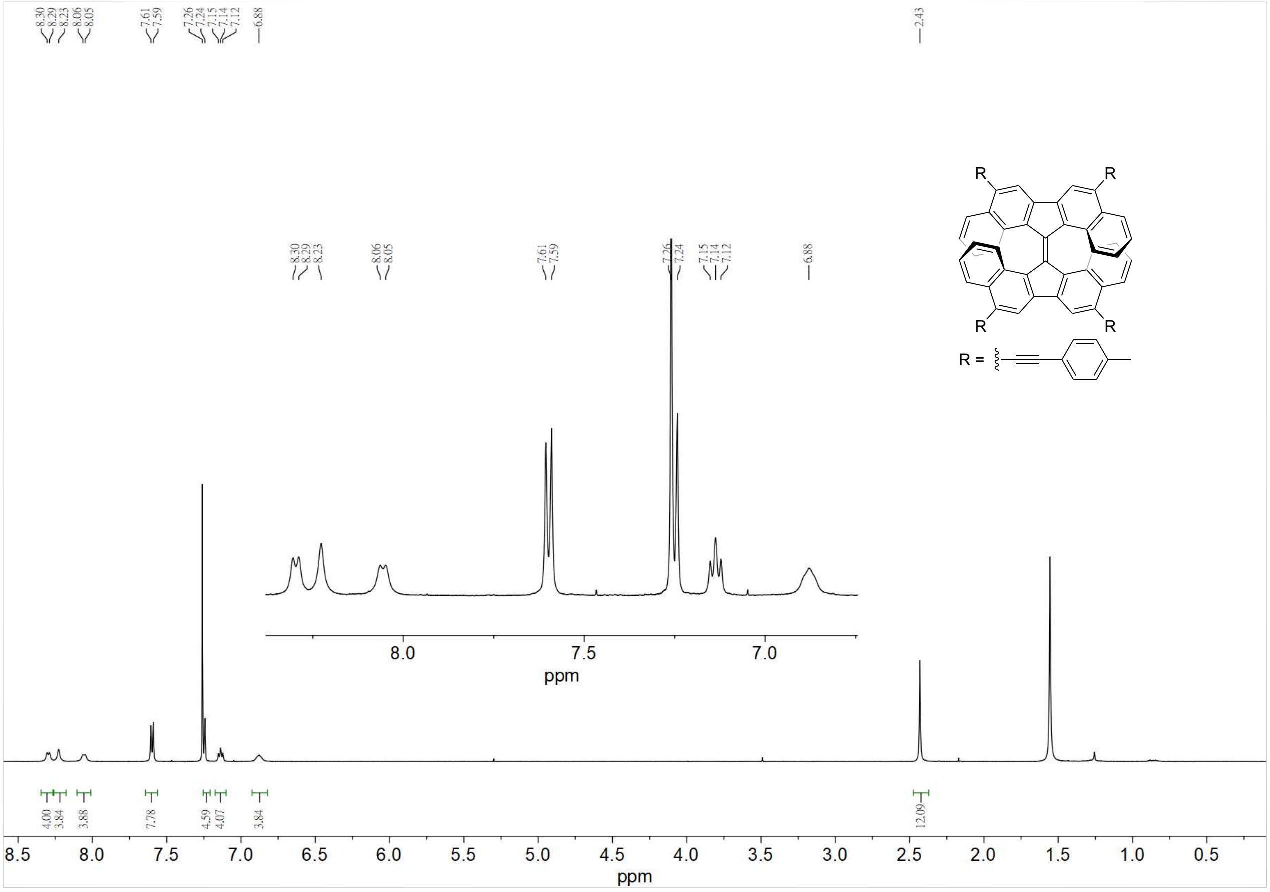

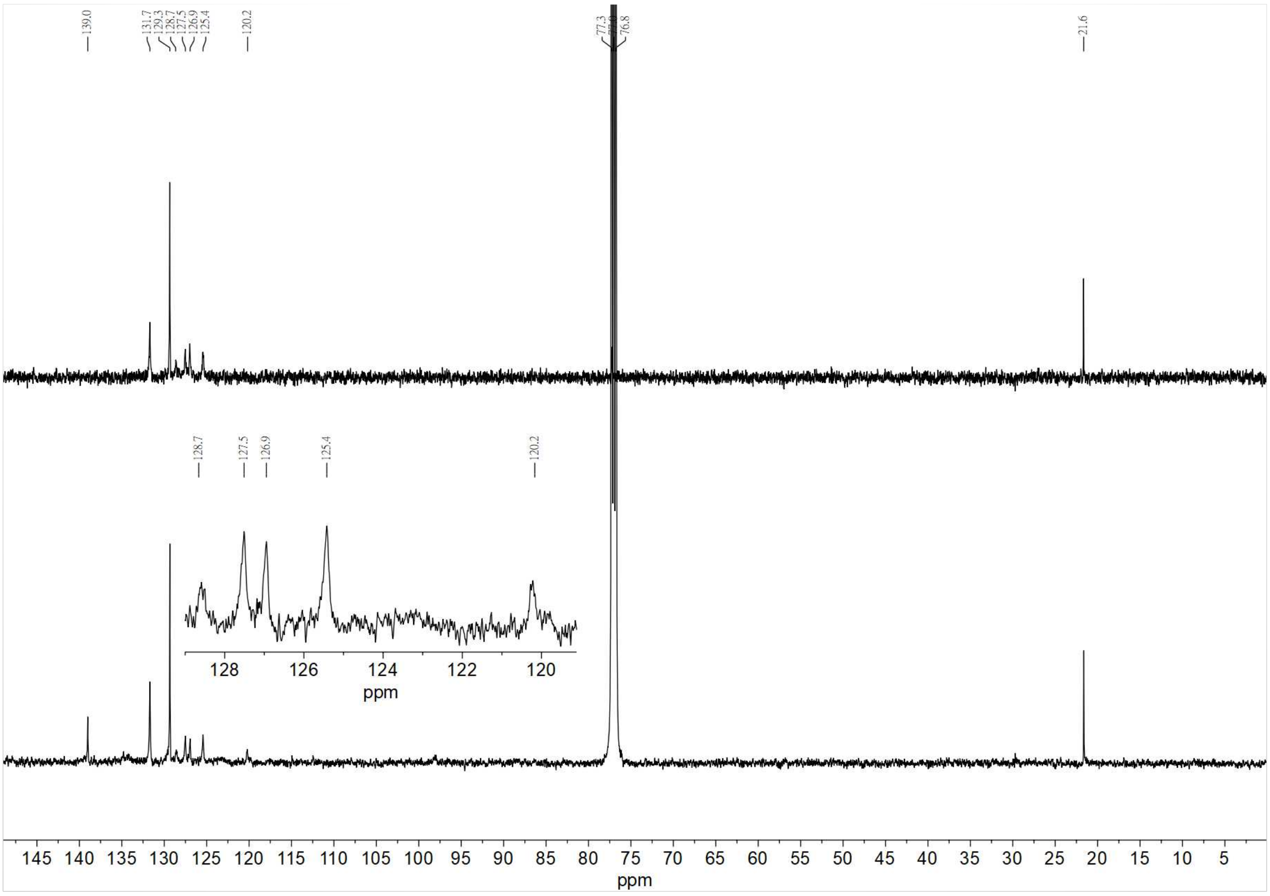

2D COSY NMR spectrum of **1b** in $CDCl_3$ (500 MHz)

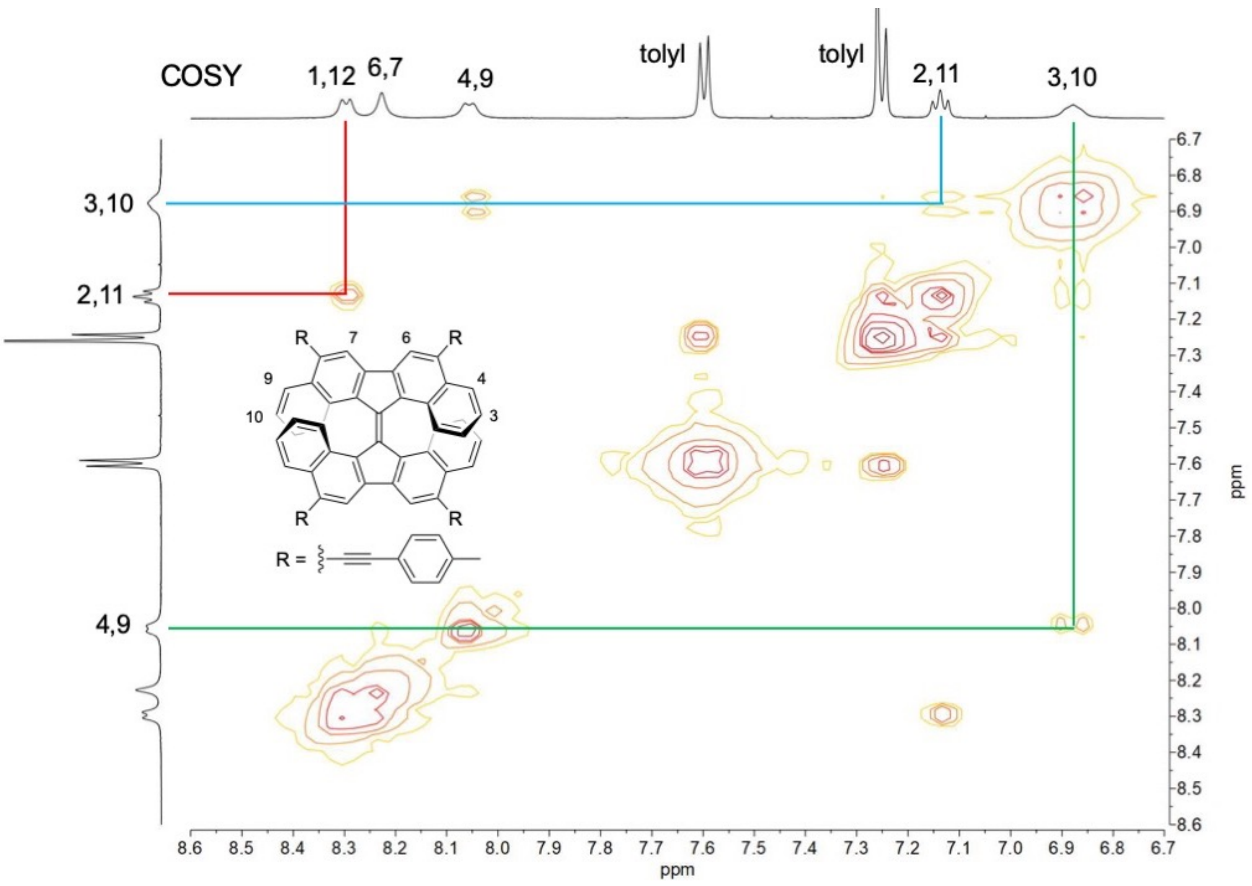

2D NOESY NMR spectrum of **1b** in $CDCl_3$ (500 MHz)

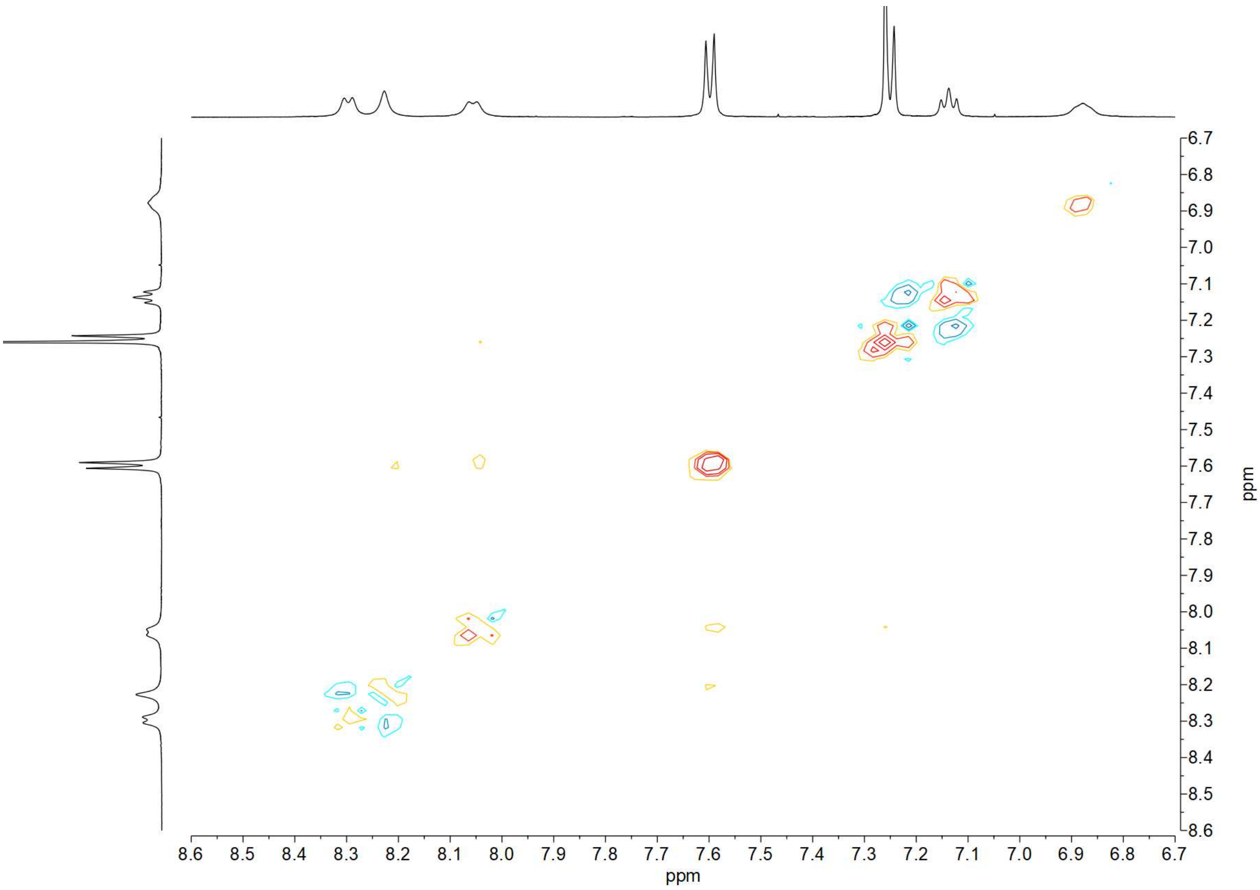

VT ^{1}H NMR spectra of **1b** (500 MHz, $CDCl_3$)

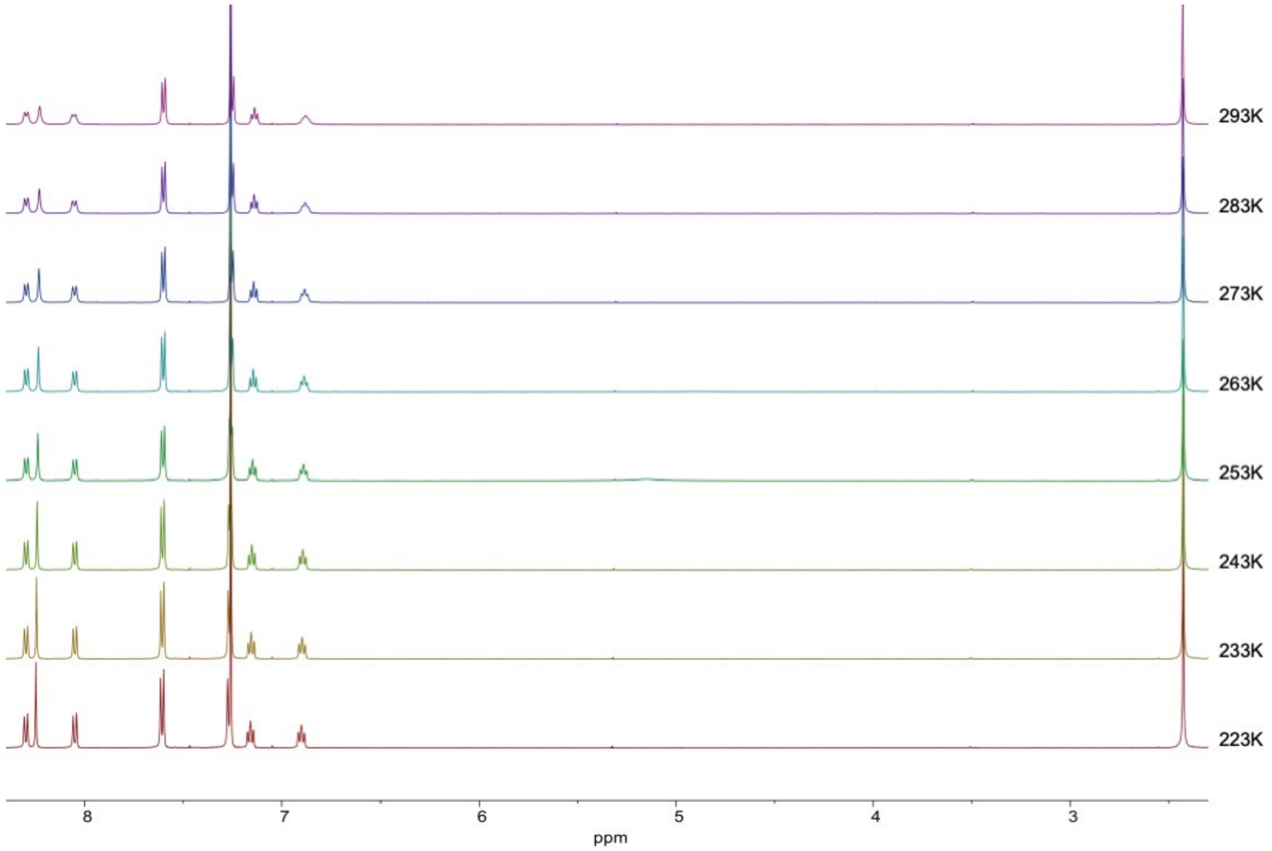

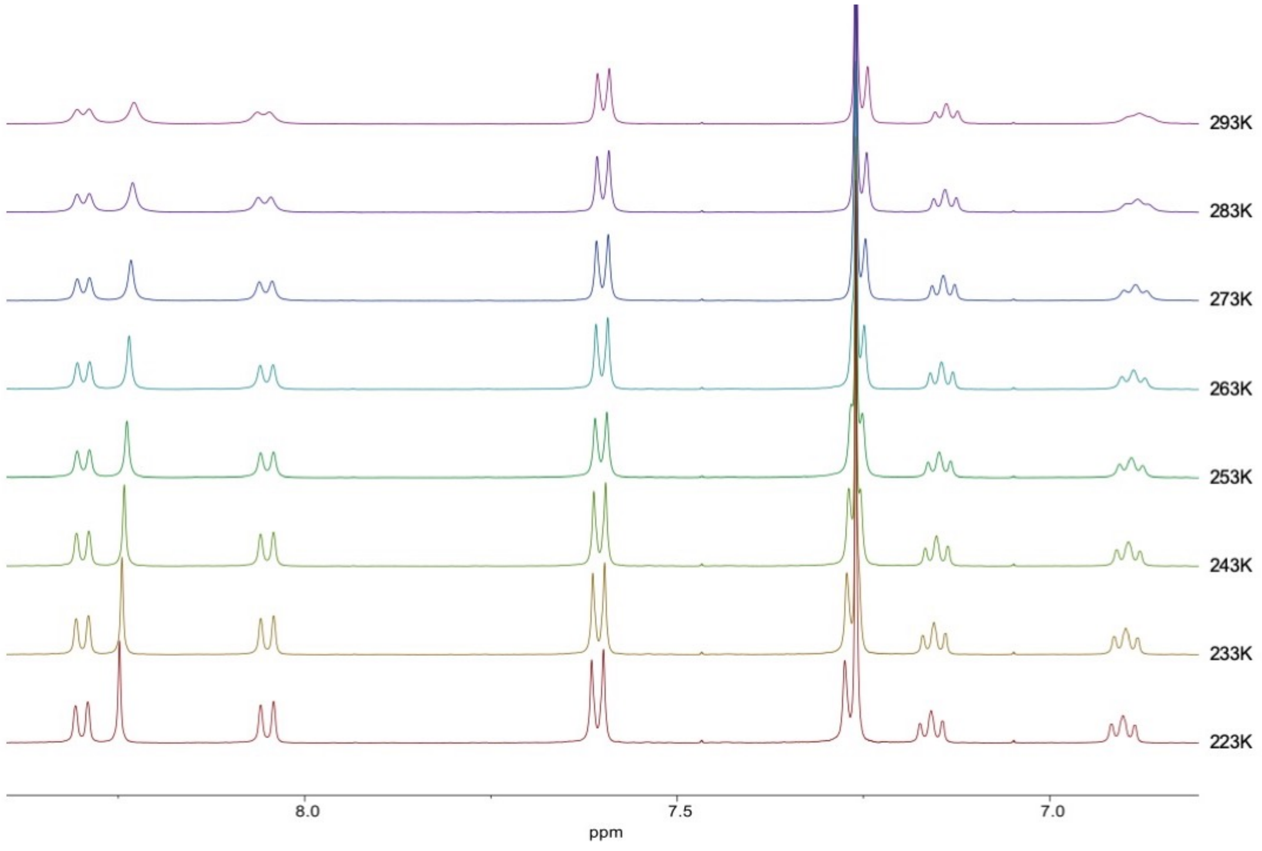

^{1}H (500 MHz) and ^{13}C (125 MHz) NMR spectra of **1c** in $CDCl_3$

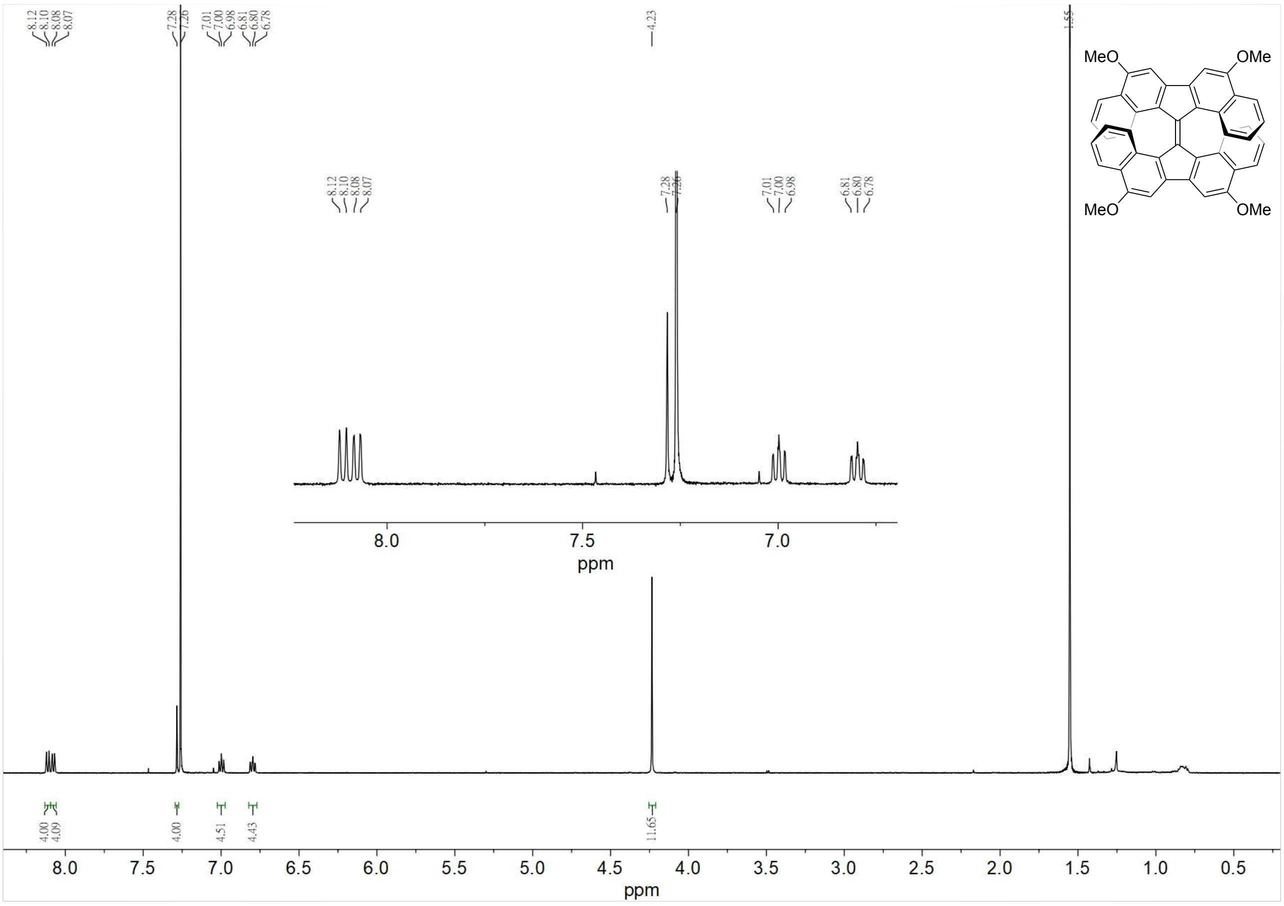

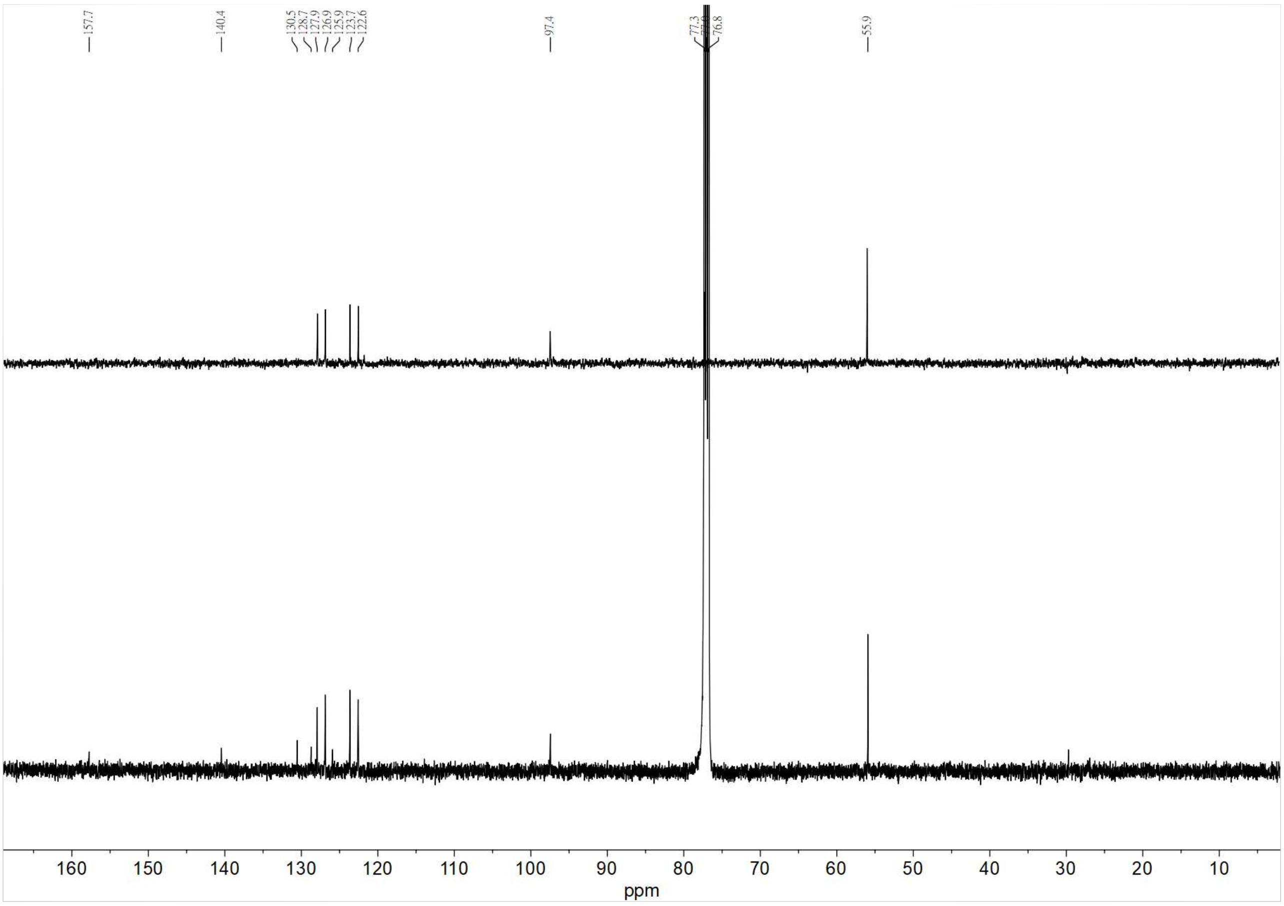

2D COSY NMR spectrum of **1c** in $CDCl_3$ (500 MHz)

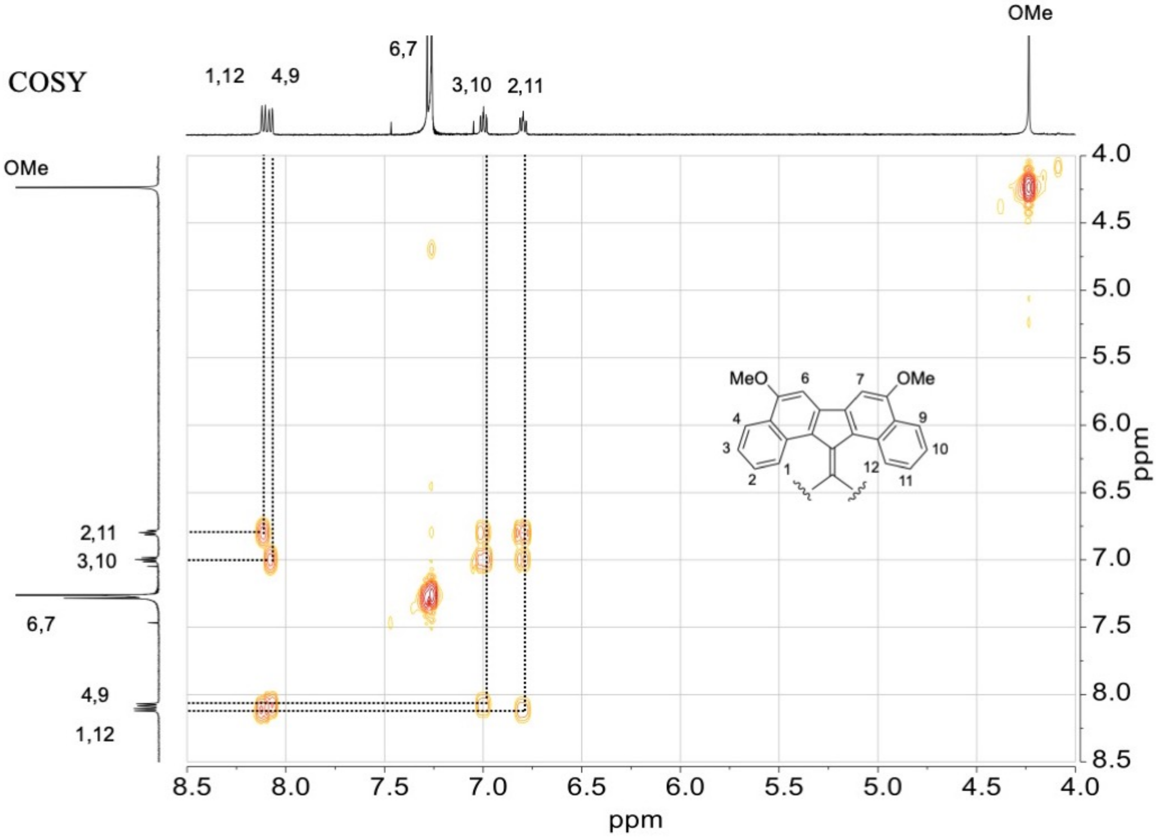

2D NOESY NMR spectrum of **1c** in $CDCl_3$ (500 MHz)

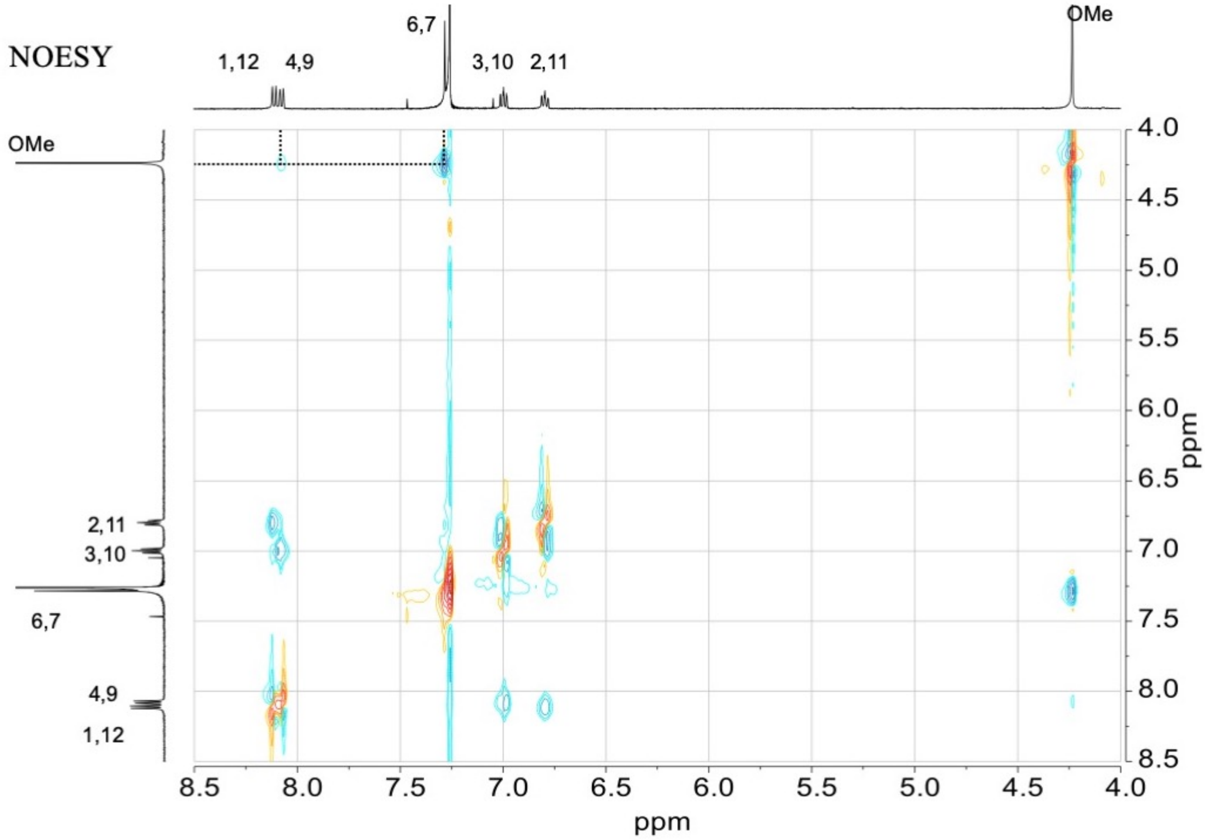

^{1}H (500 MHz) and ^{13}C (125 MHz) NMR spectra of **1d** in $CDCl_3$

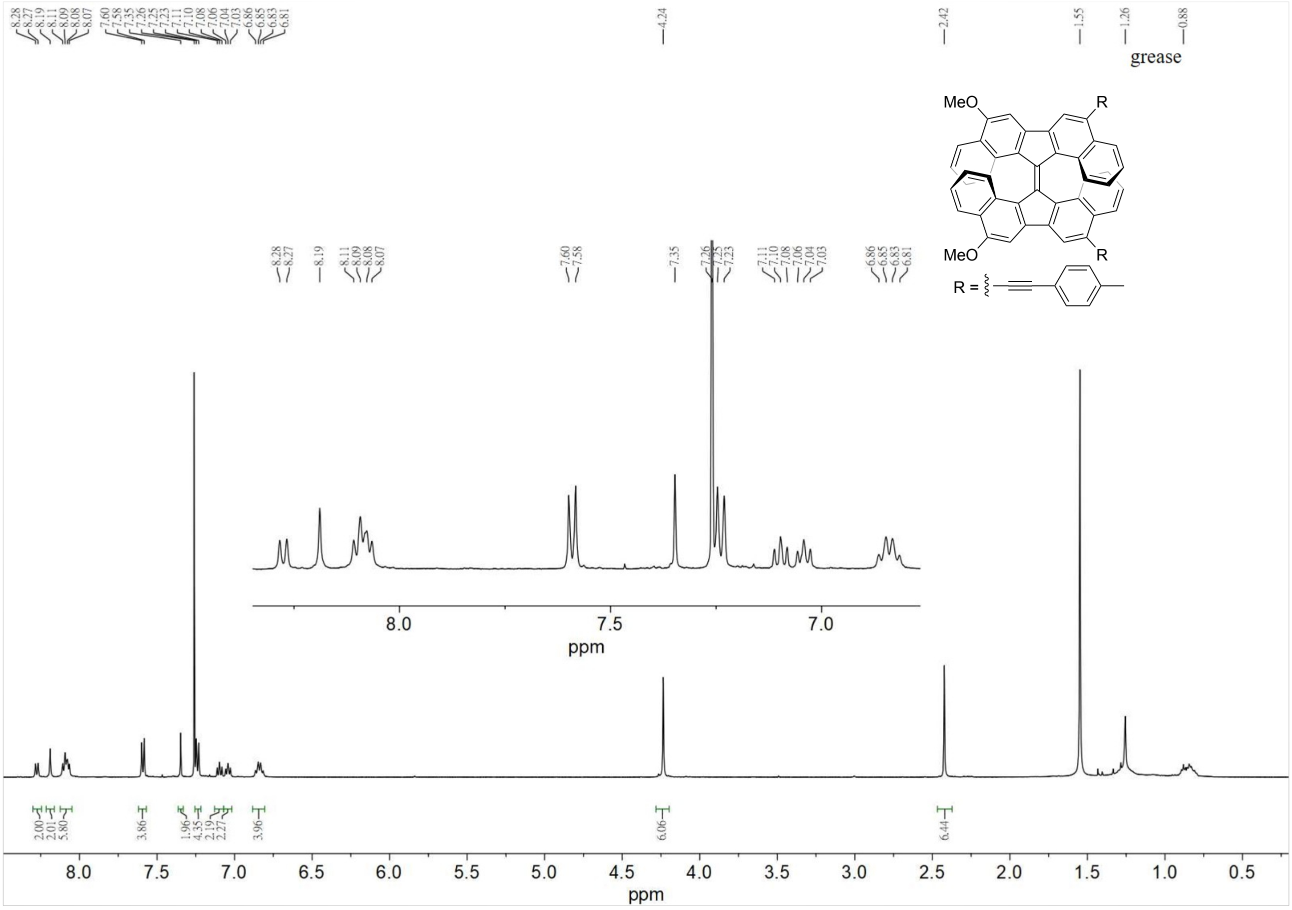

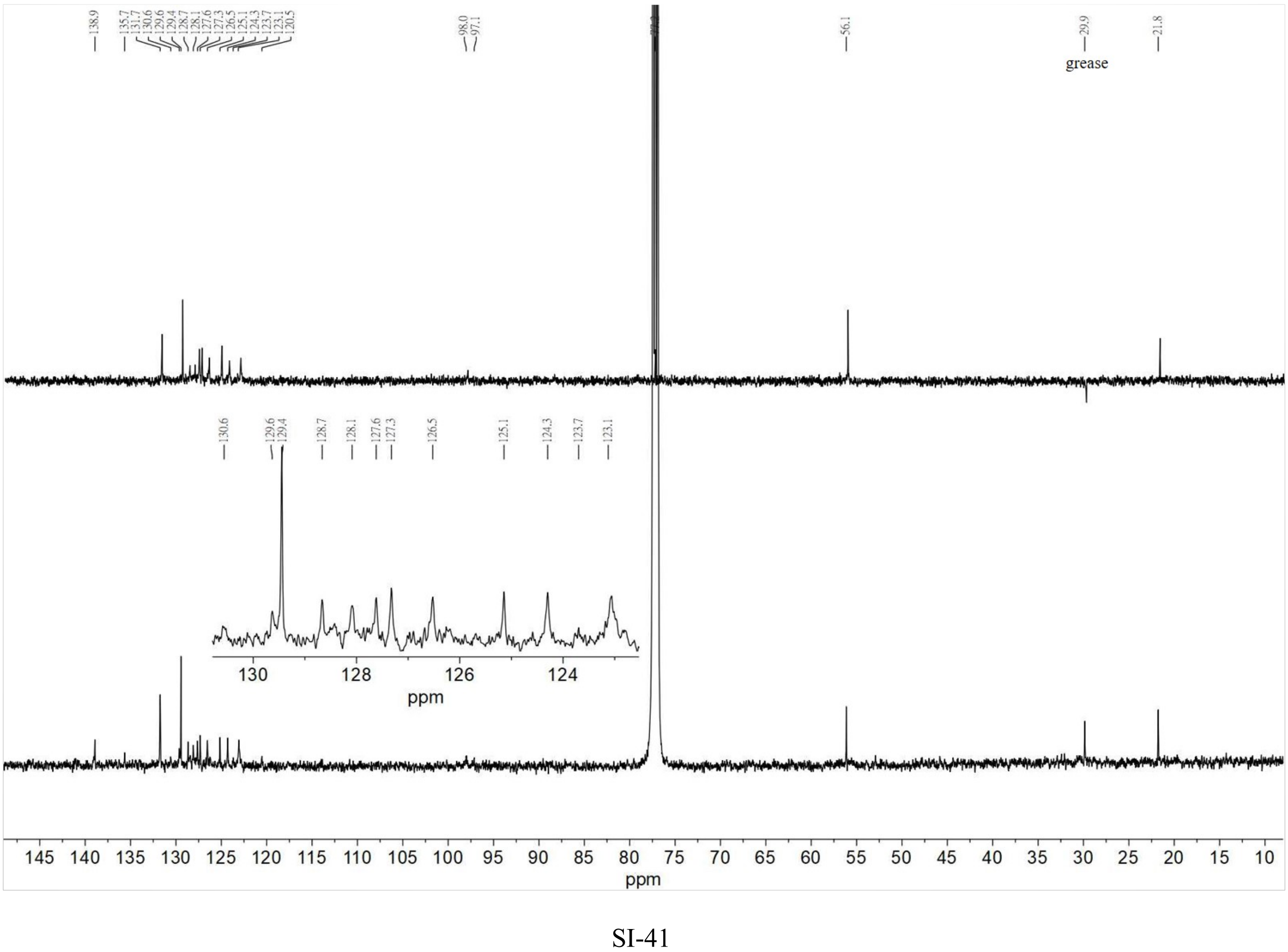

^{1}H NMR spectrum of **1d** in CD_2Cl_2 (500 MHz, 293 K)

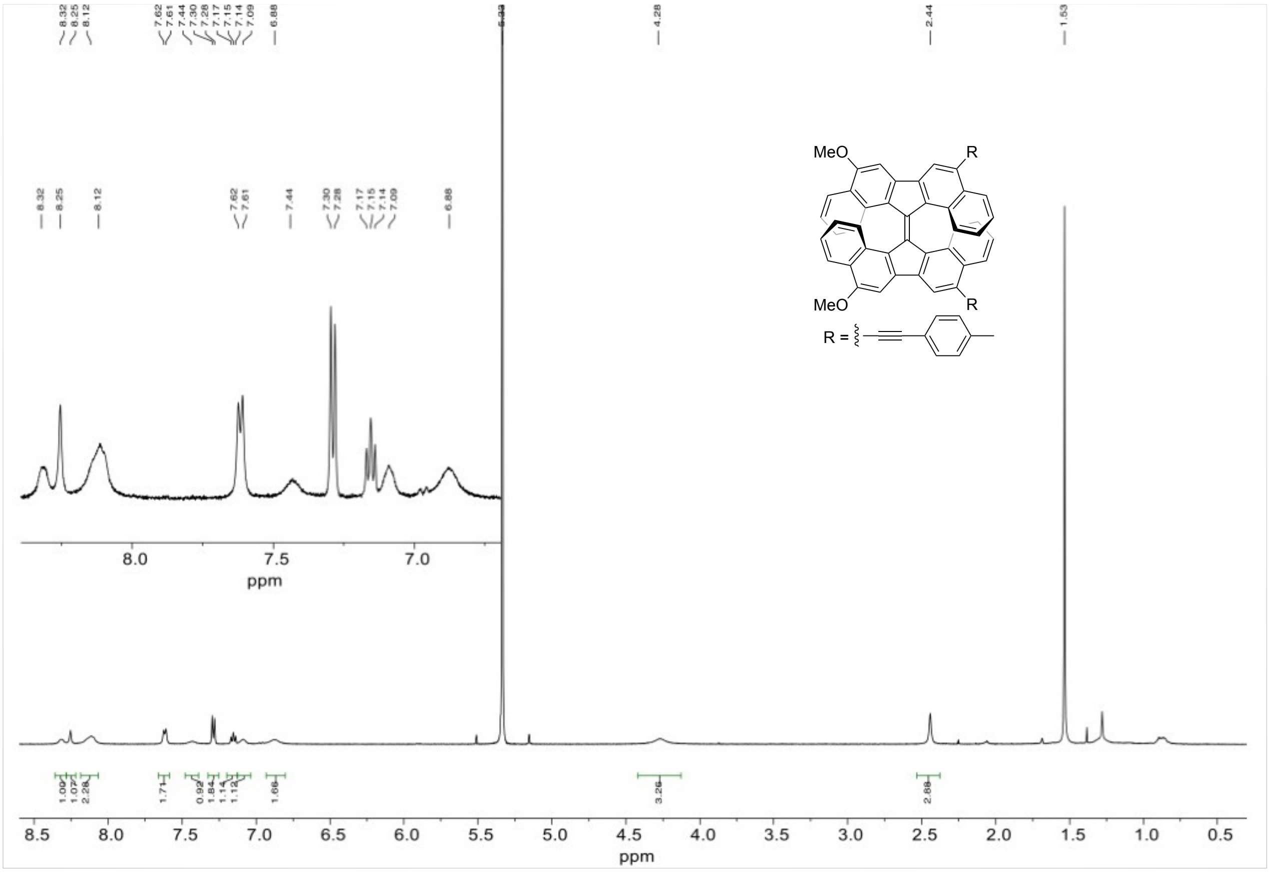

^{1}H NMR spectrum of **1d** in CD_2Cl_2 (500 MHz, 183 K)

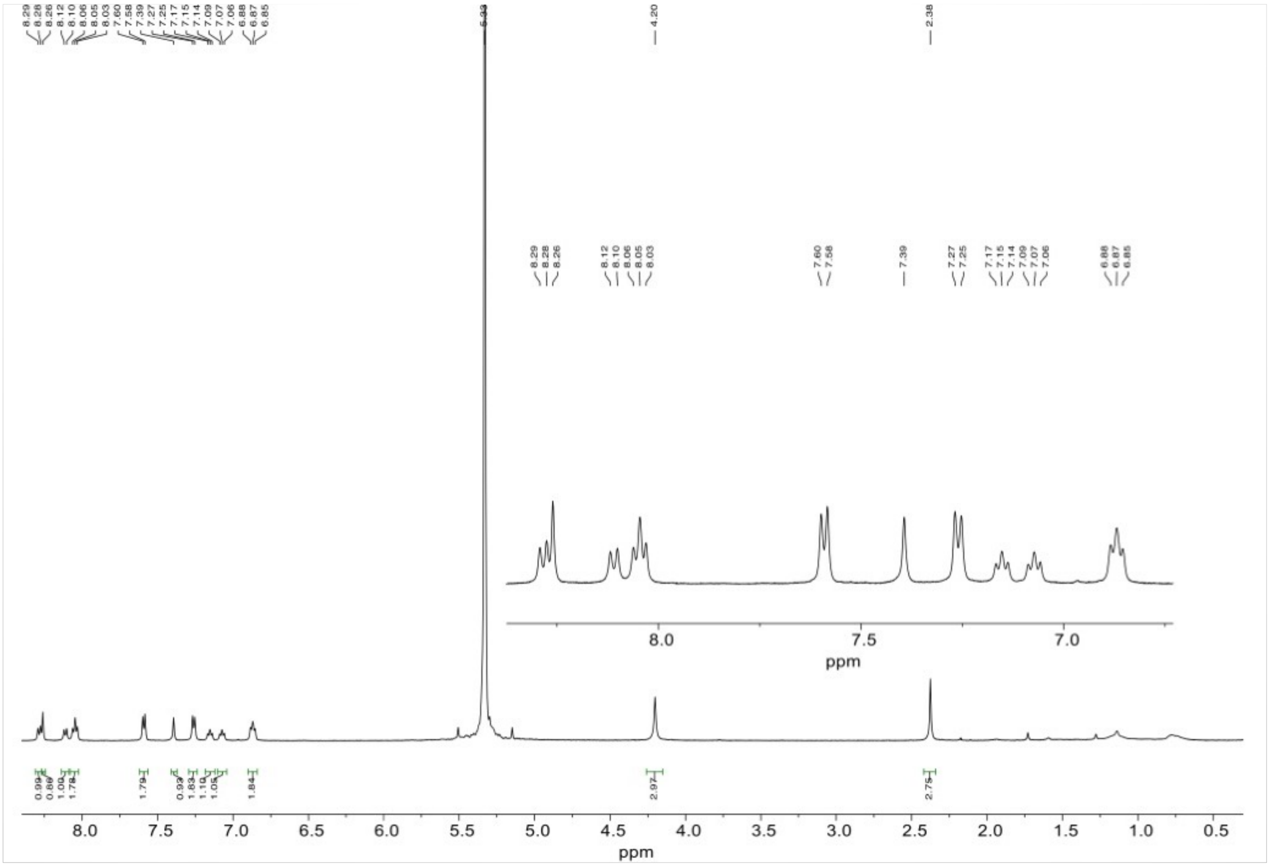

VT ^{1}H NMR spectrum of **1d** in CD_2Cl_2 (500 MHz)

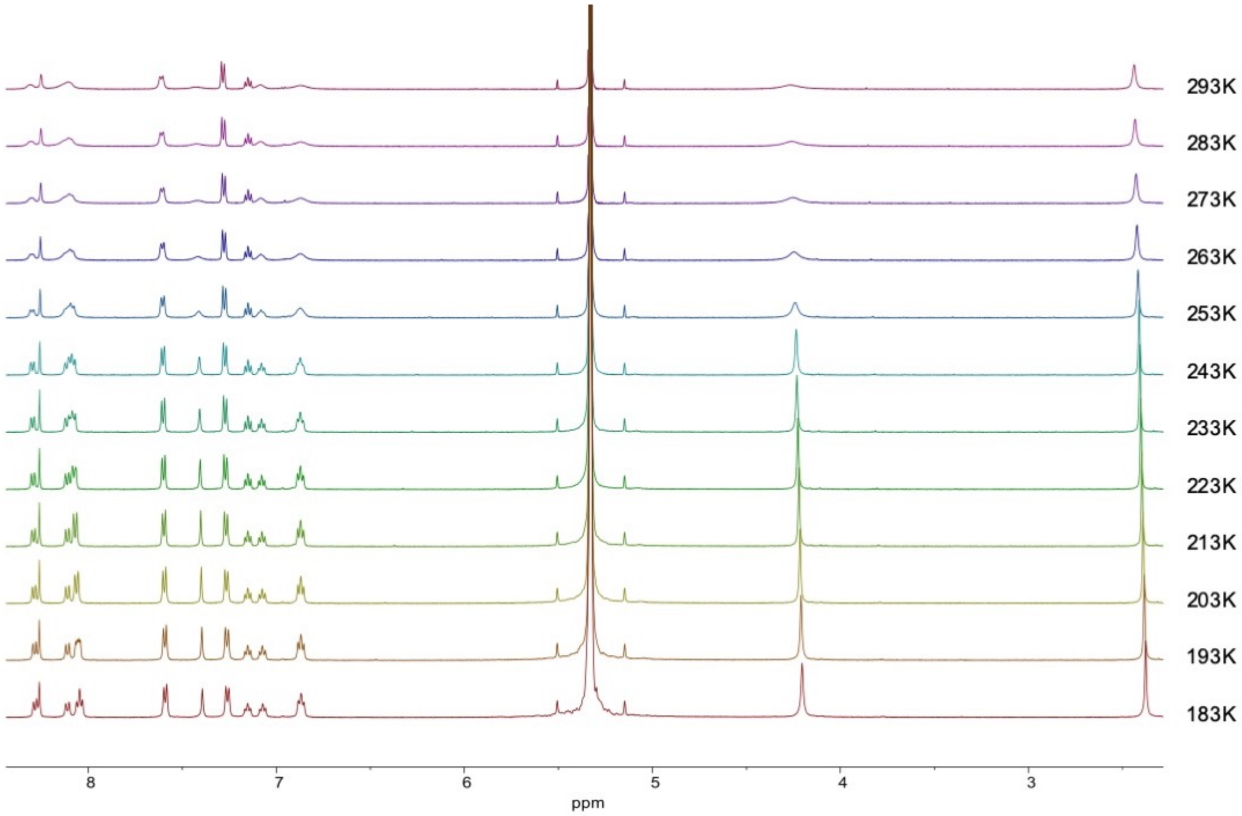

VT ^{1}H NMR spectrum of **1d** (6.5−9 ppm) in CD_2Cl_2 (500 MHz)

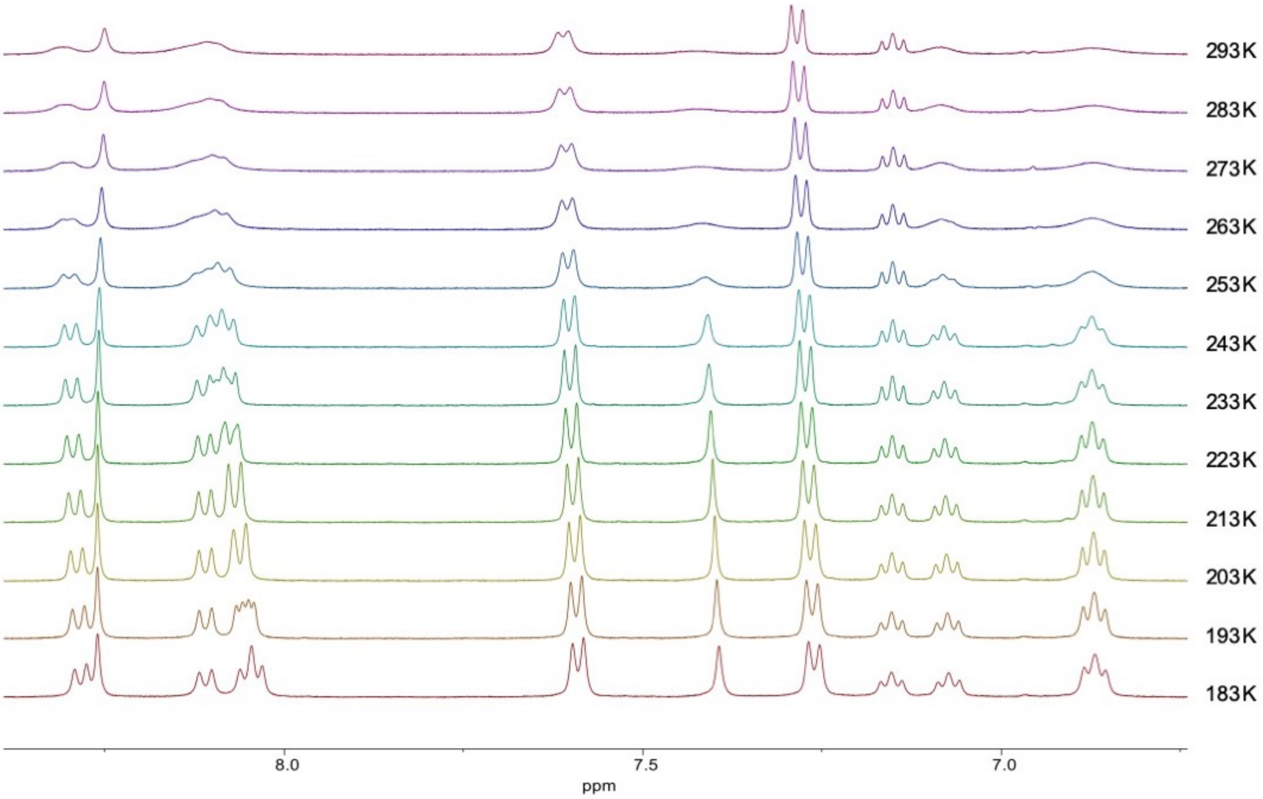

^{1}H (700 MHz) and ^{13}C (125 MHz) NMR spectra of **2** in $CDCl_3$

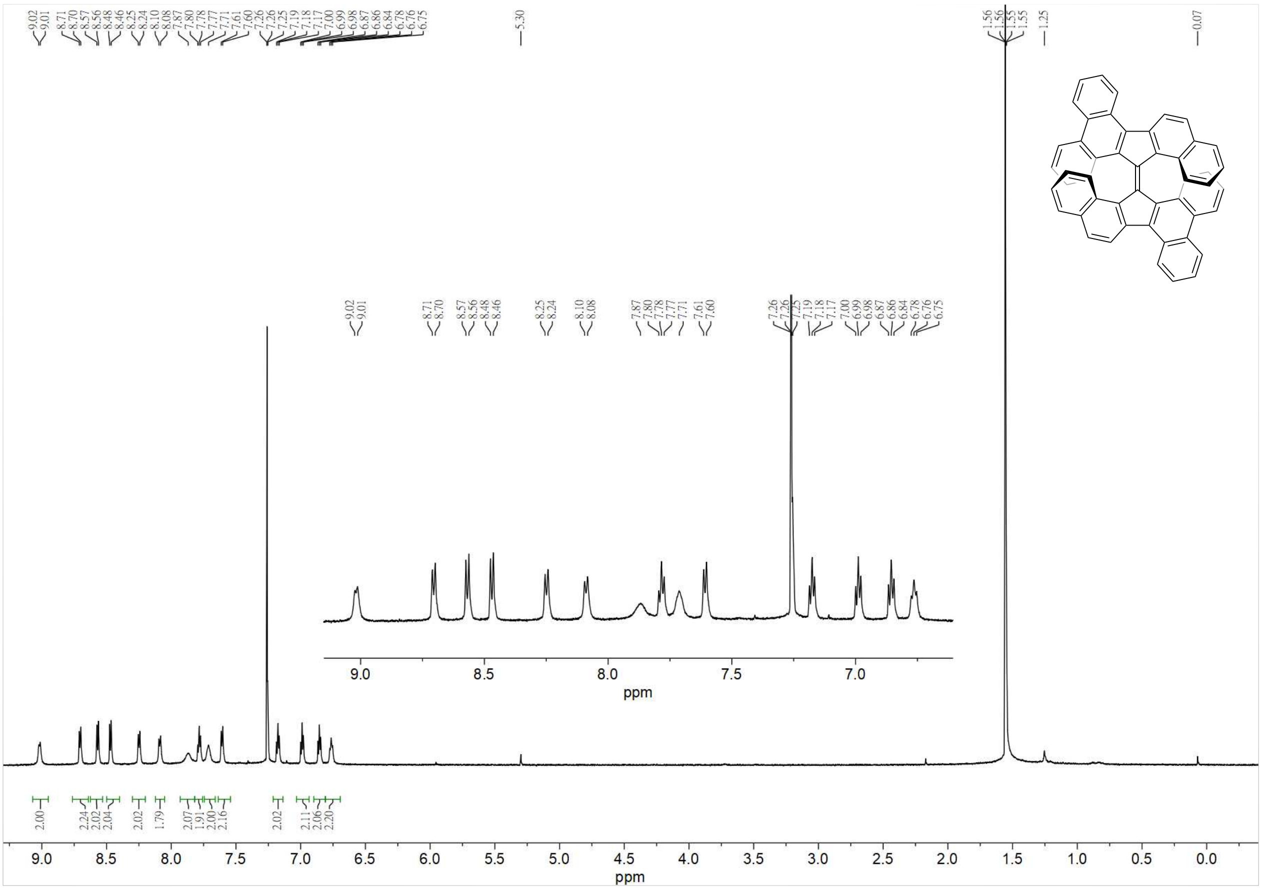

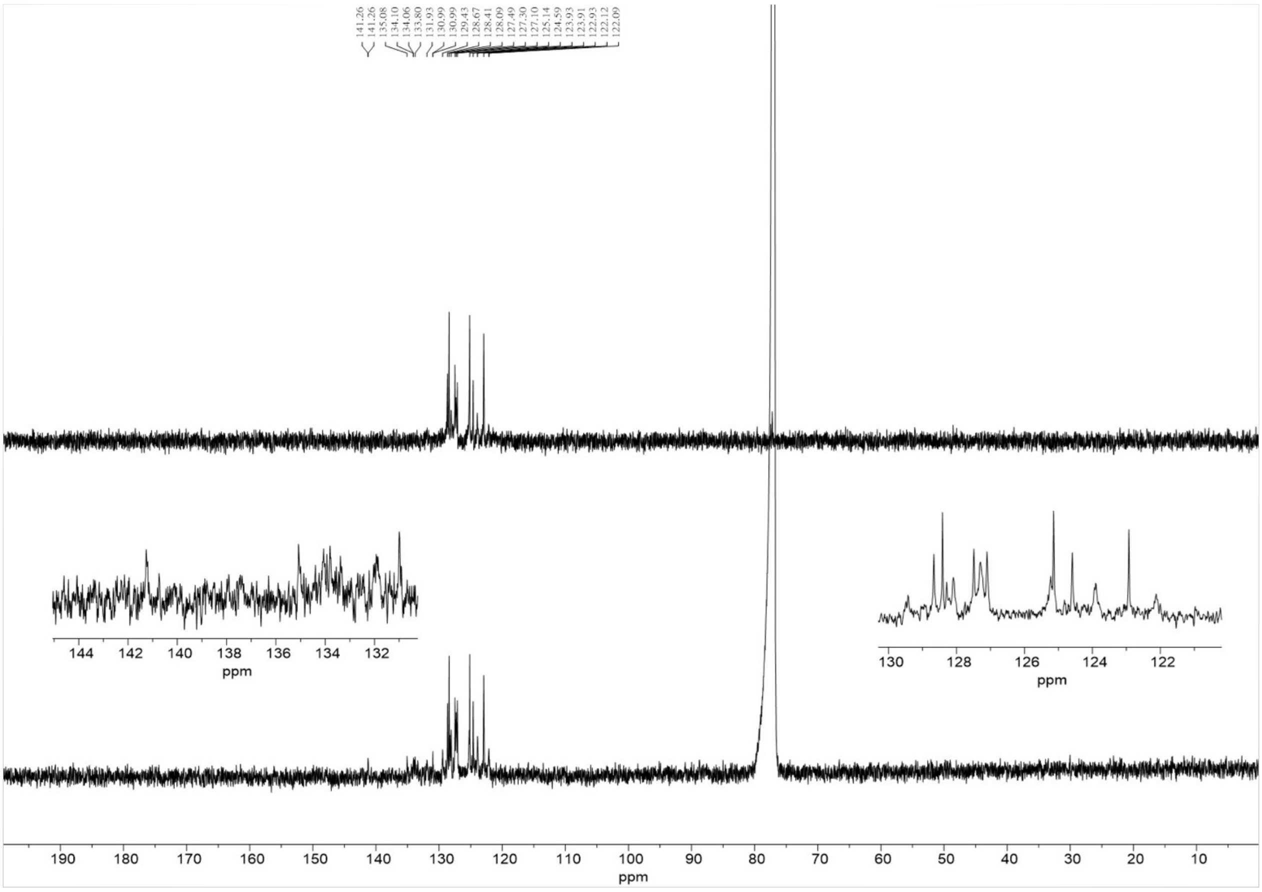

VT ^{1}H NMR spectrum of **2** in $C_2D_2Cl_4$ (500 MHz)

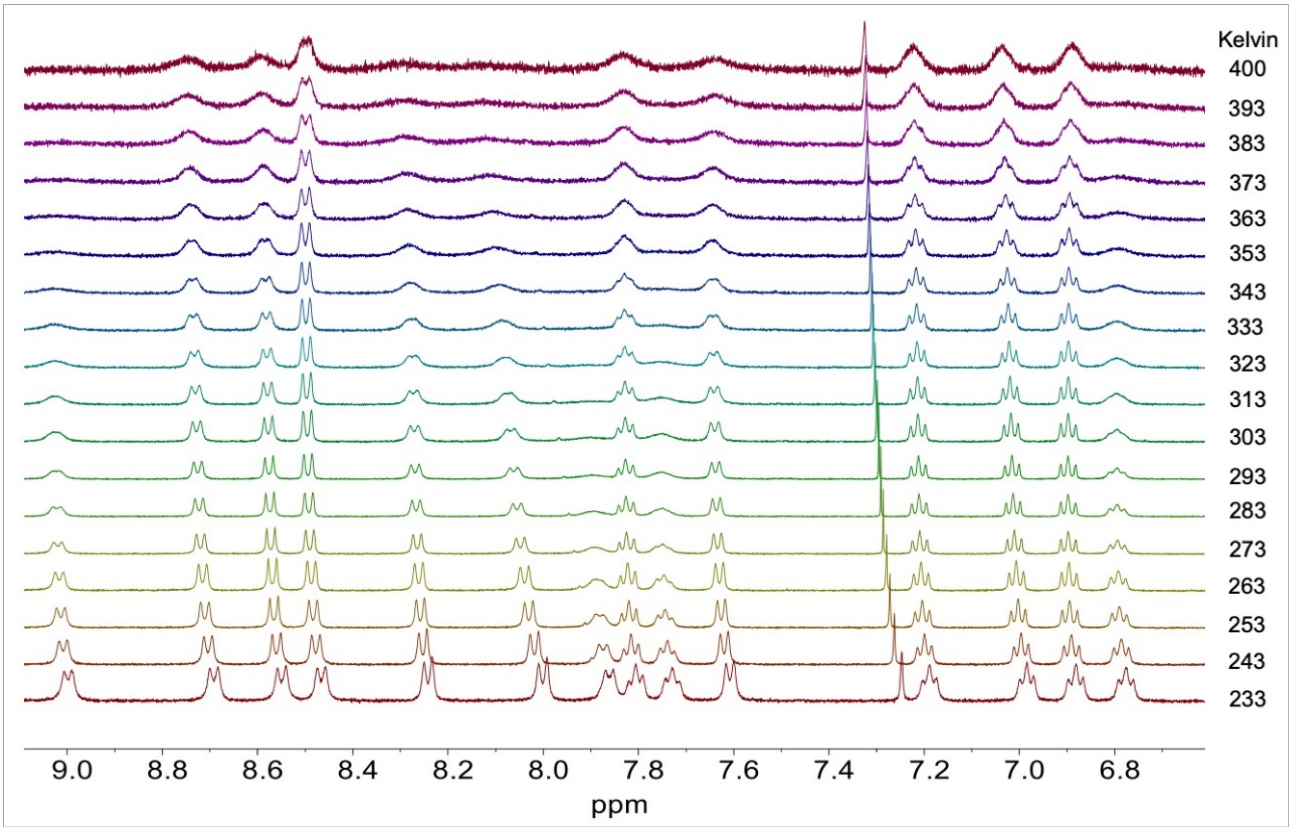

VT ^{1}H NMR spectrum of **2** in CD_2Cl_2 (500 MHz)

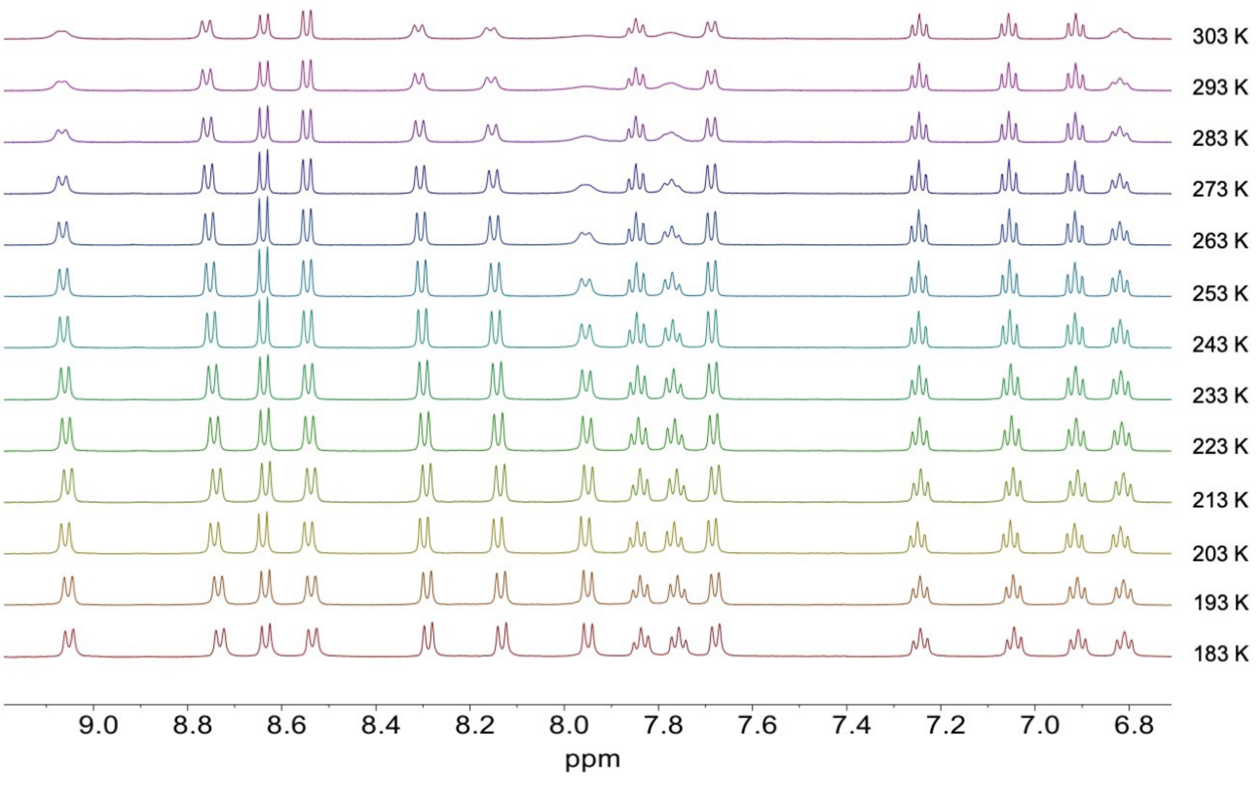

2D COSY NMR spectrum of **2** in $CDCl_3$ (700 MHz)

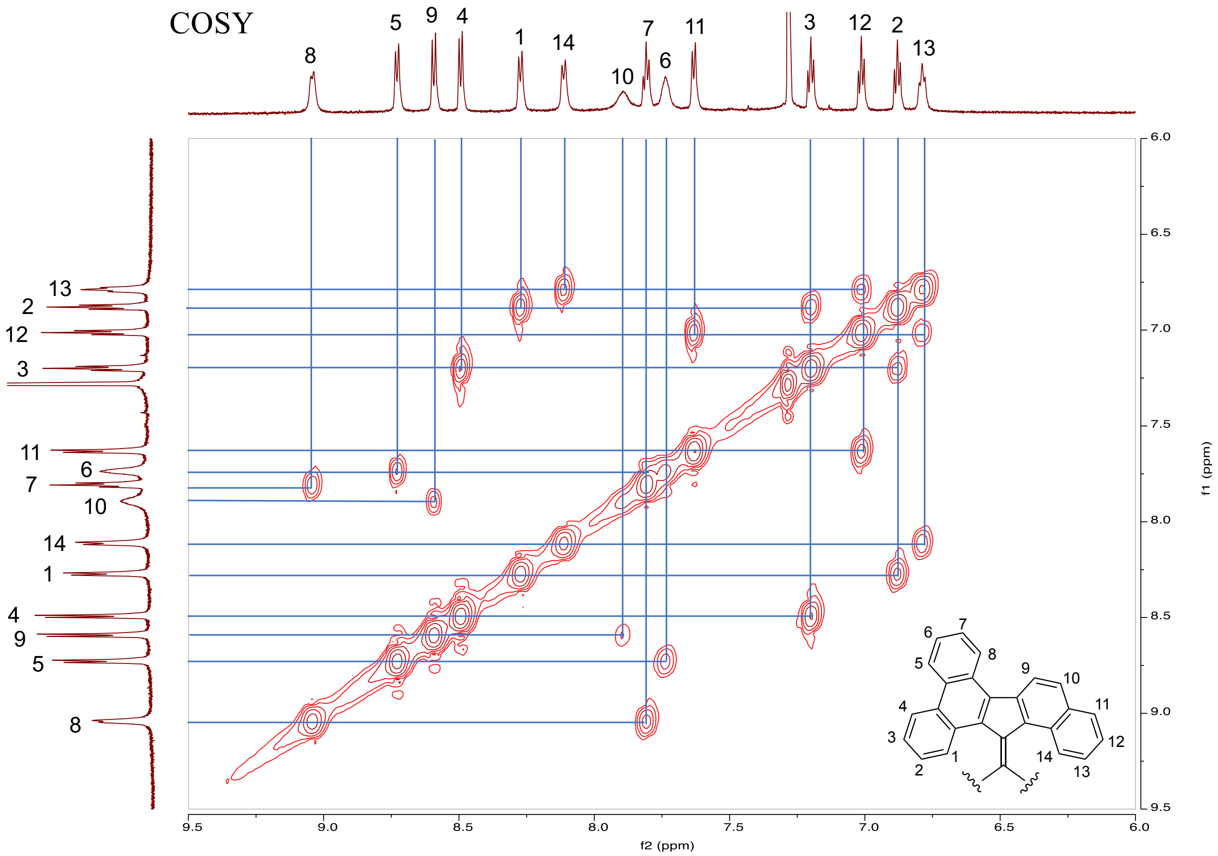

2D NOESY NMR spectrum of **2** in $CDCl_3$ (700 MHz)

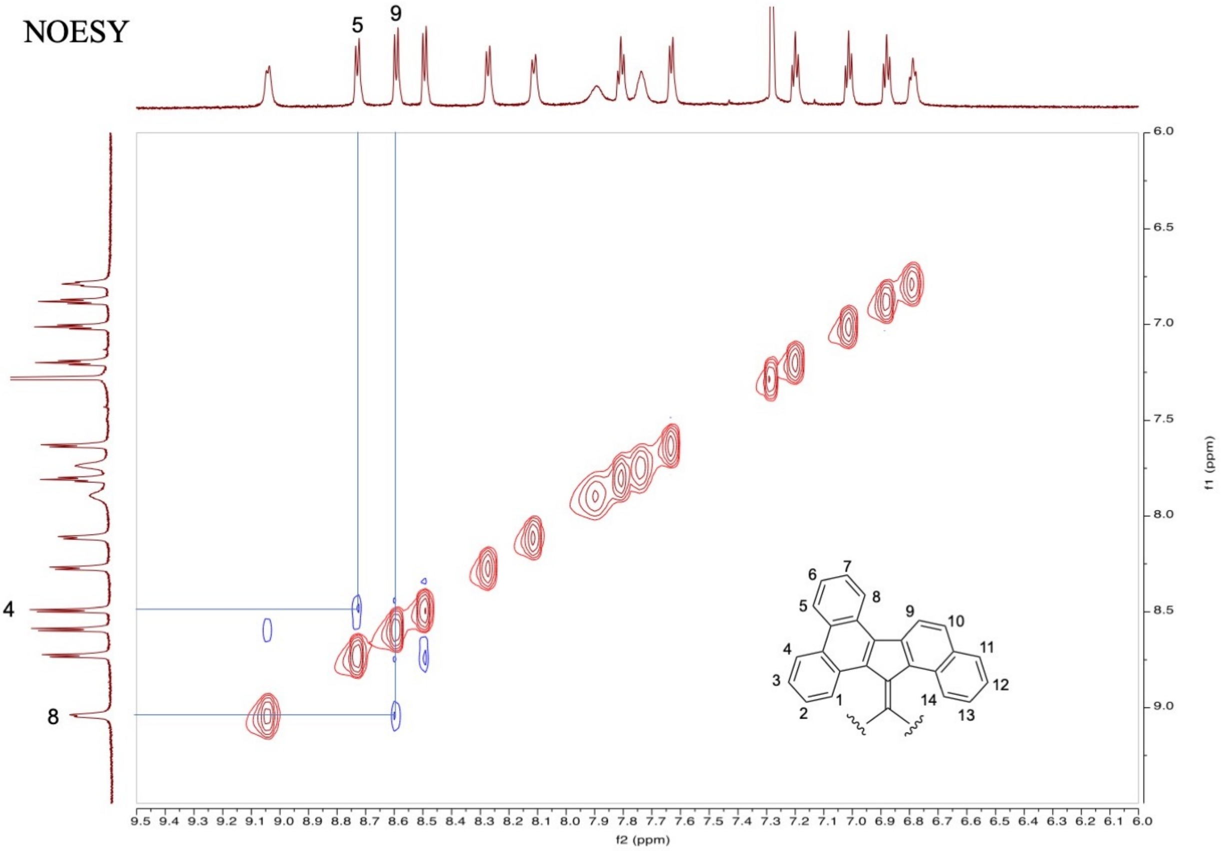

^{1}H NMR spectrum of **3** in $CDCl_3$ (400 MHz, 298 K)

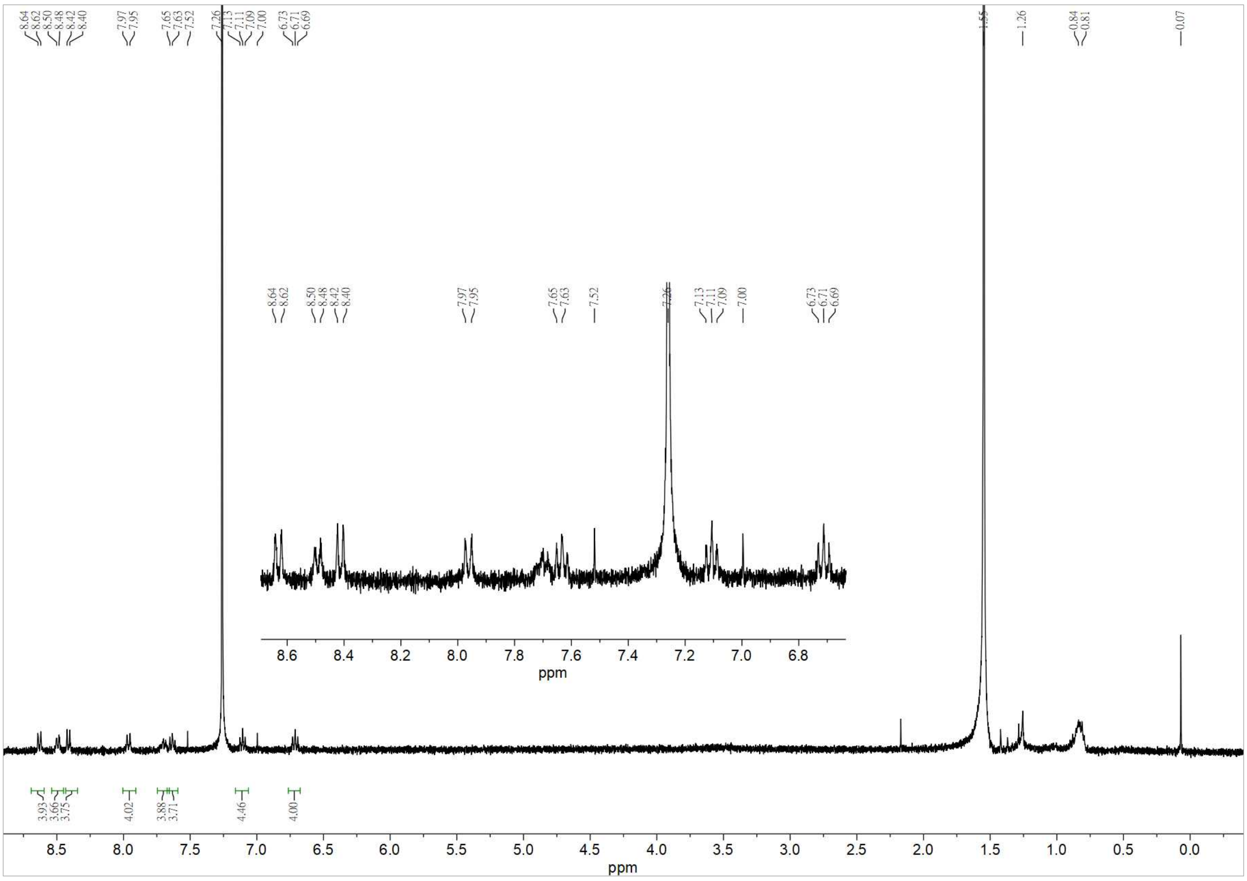

^{1}H NMR spectrum of **3** in $C_2D_2Cl_4$ (500 MHz, 293 K)

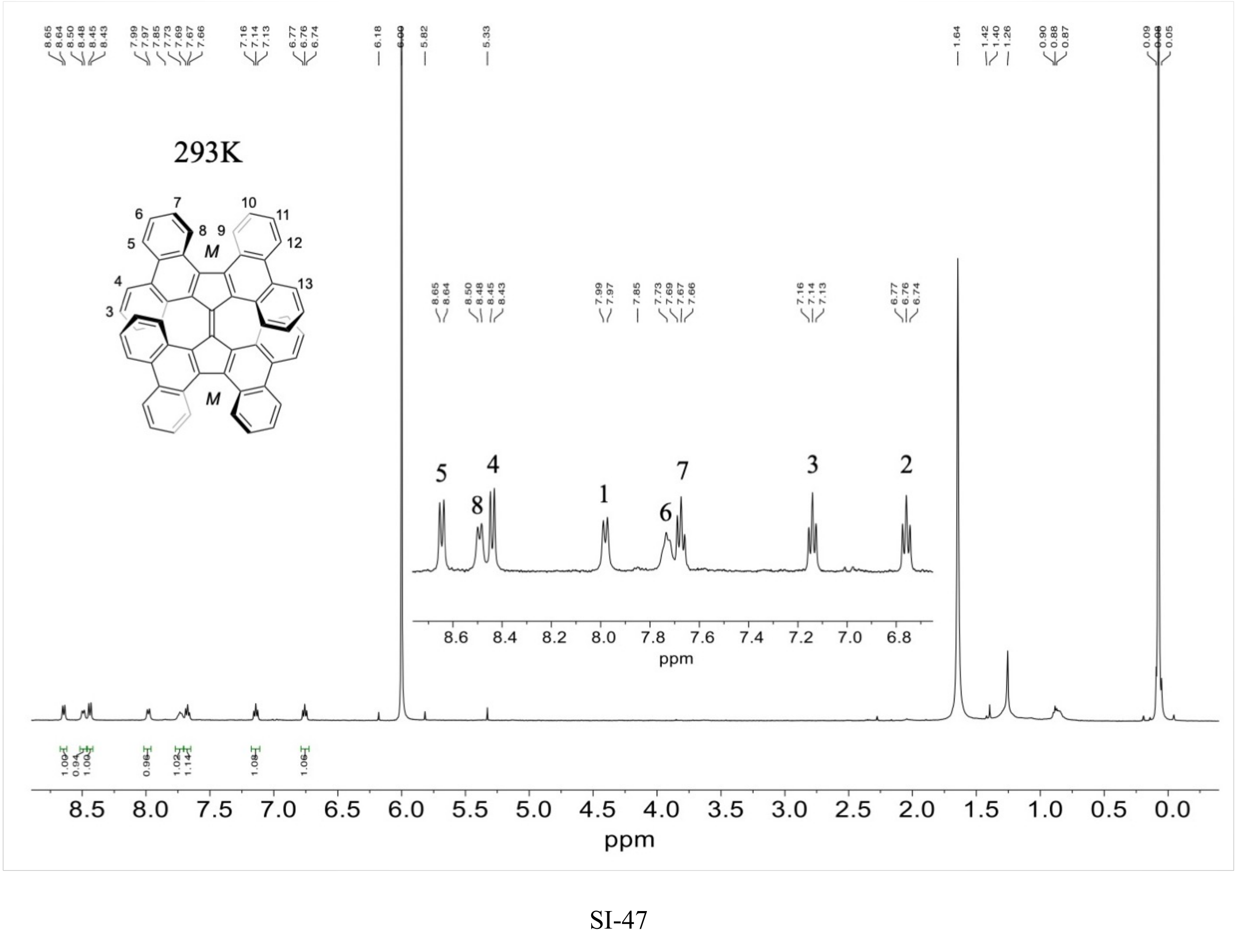

^{1}H NMR spectrum of **3** in $C_2D_2Cl_4$ (500 MHz, 233 K)

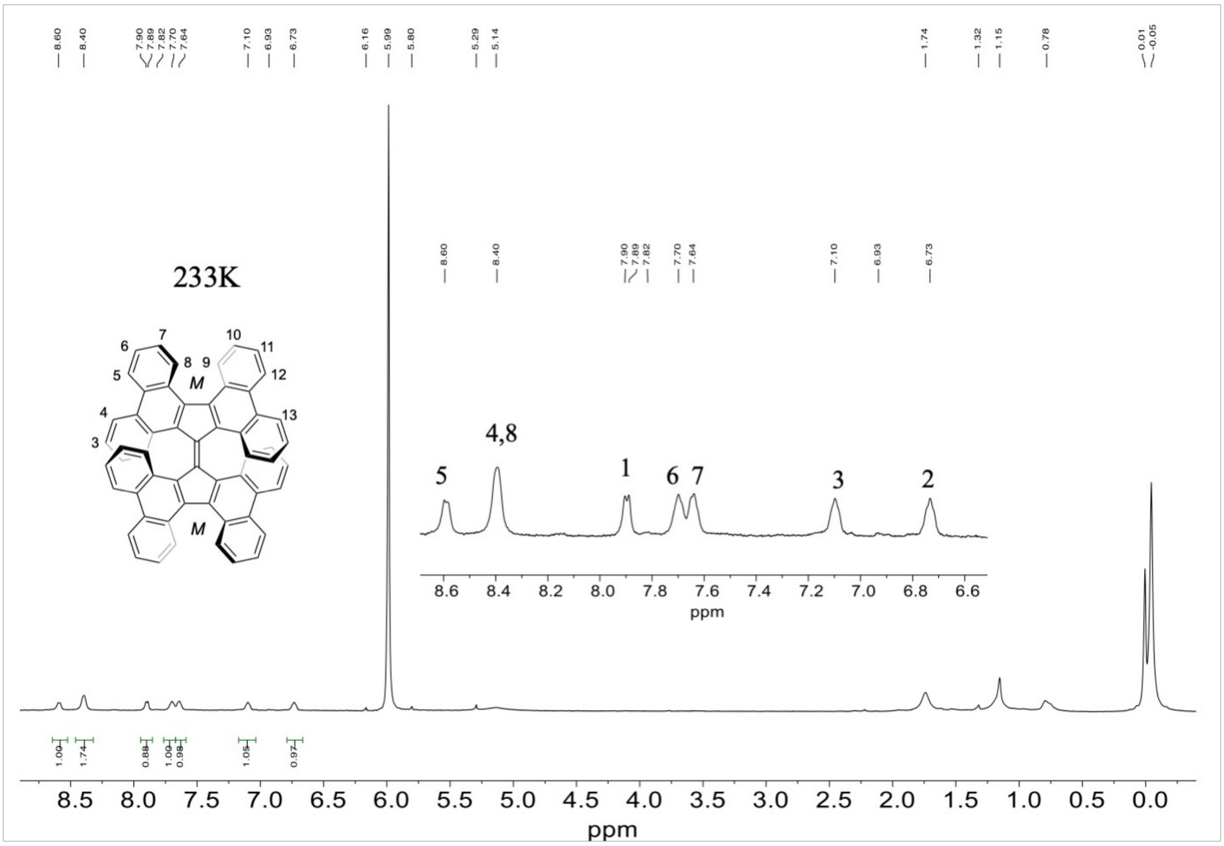

In VT ^{1}H NMR spectra of **3**, peaks with progressive line broadening were observed at temperatures higher than 303 K, and signals of 6/11-H and 8/9-H disappeared at temperatures above 353 K and 373 K, respectively. Below 293 K, resonances gradually became broad again, presumably owing to the low solubility.

VT ^{1}H NMR spectra of **3** in $C_2D_2Cl_4$ (500 MHz).

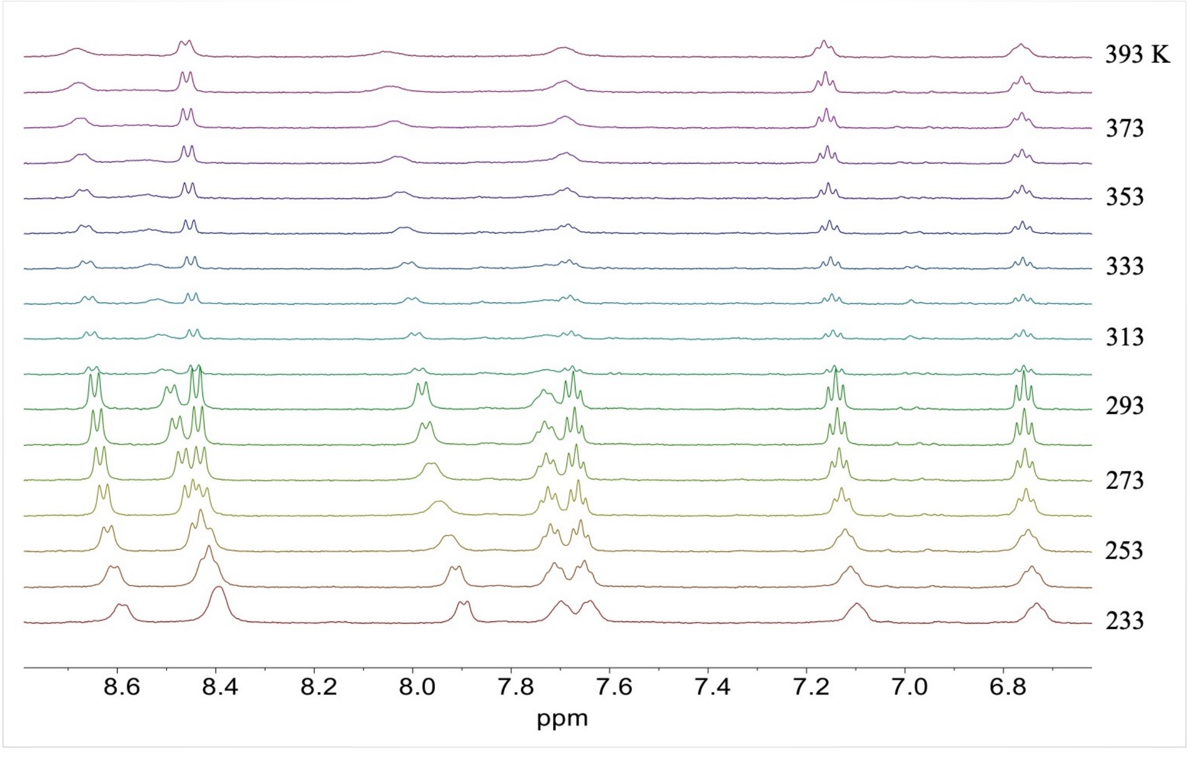

2D COSY NMR spectrum of **3** in $C_2D_2Cl_4$ (700 MHz)

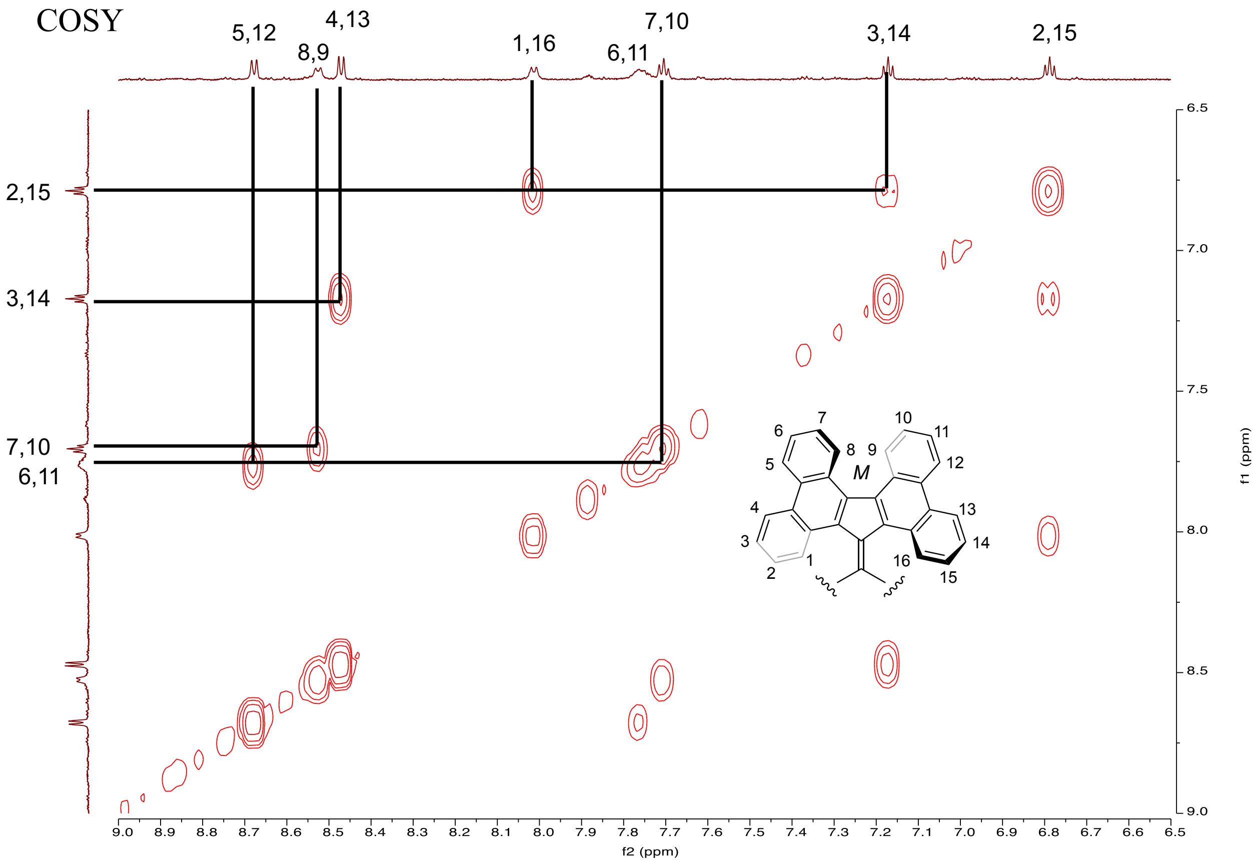

2D NOESY NMR spectrum of **3** in $C_2D_2Cl_4$ (700 MHz)

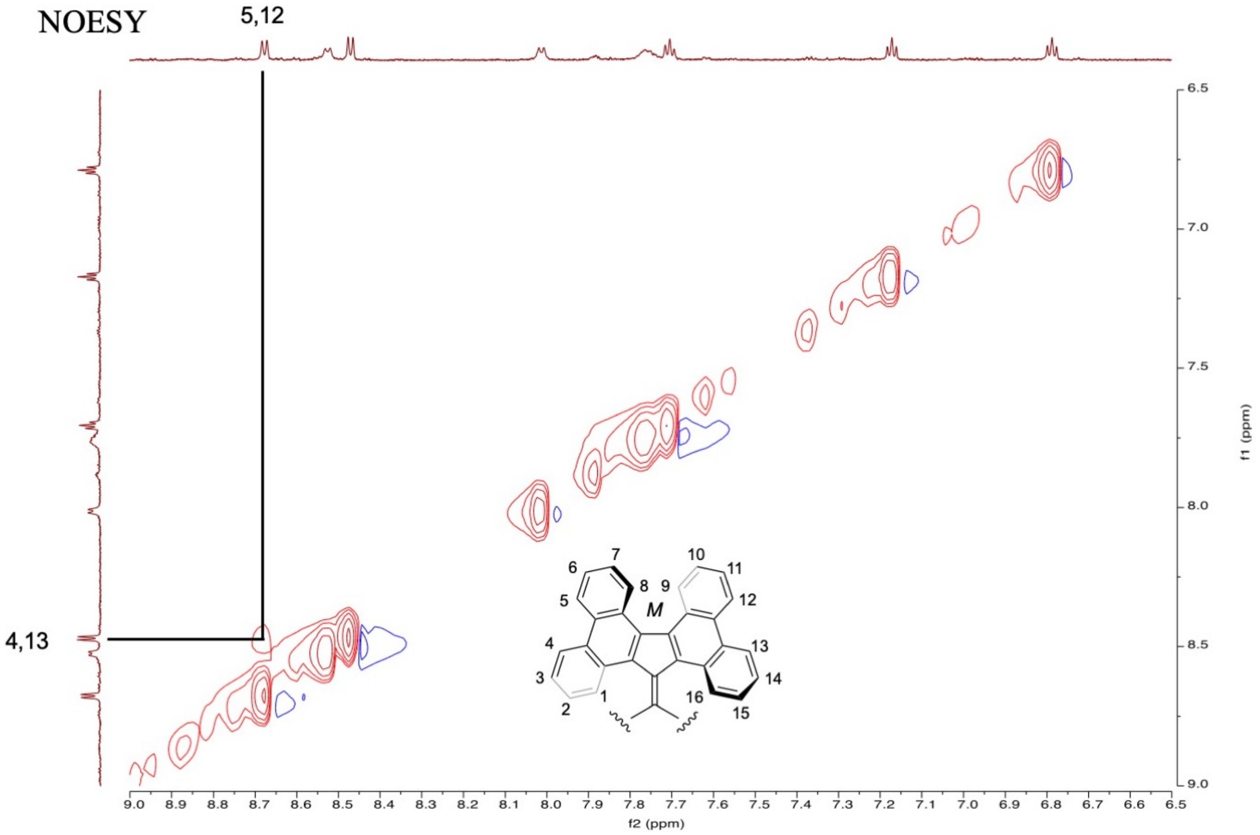

^1H (500 MHz) and ^{13}C (125 MHz) NMR spectra of **4b** in $CDCl_3$

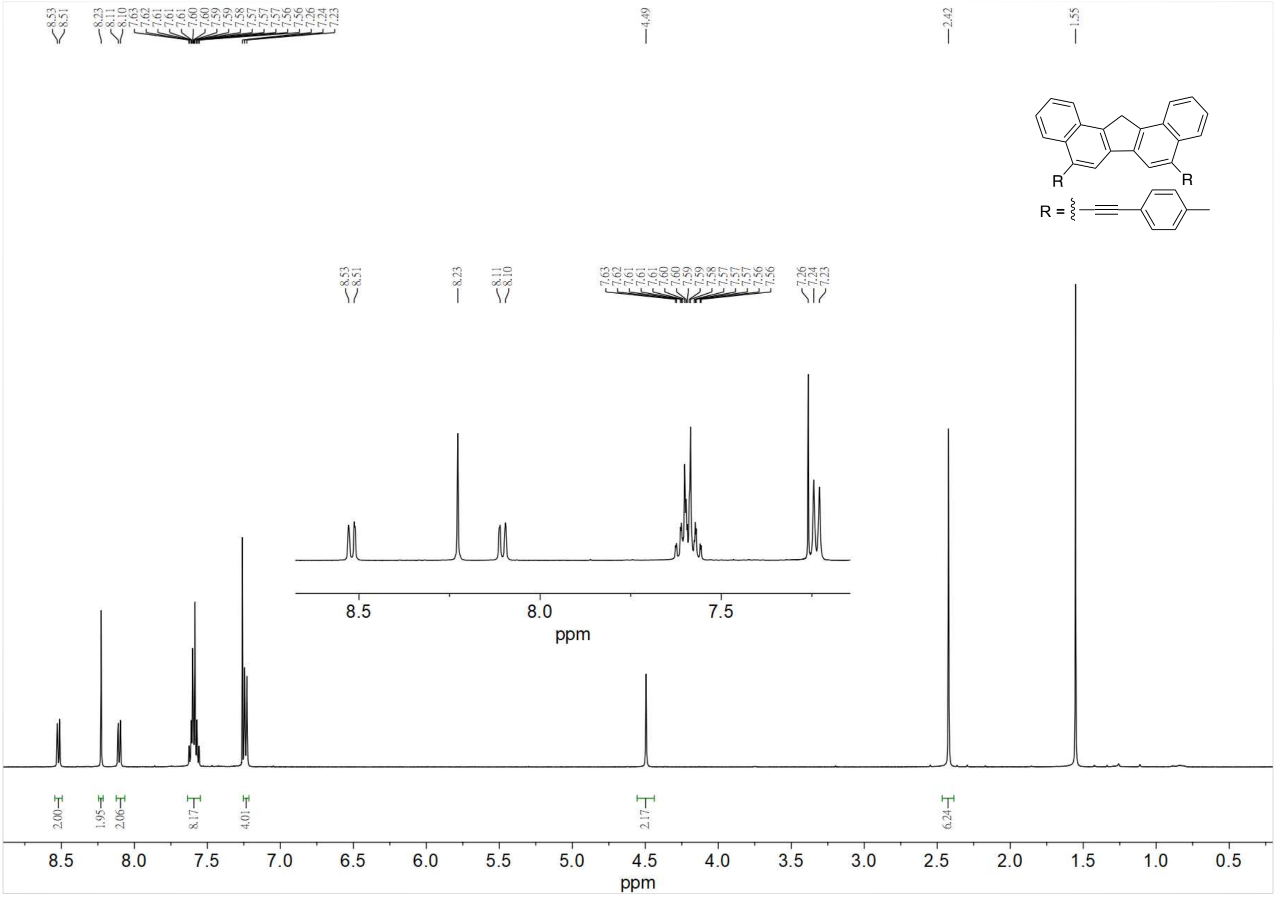

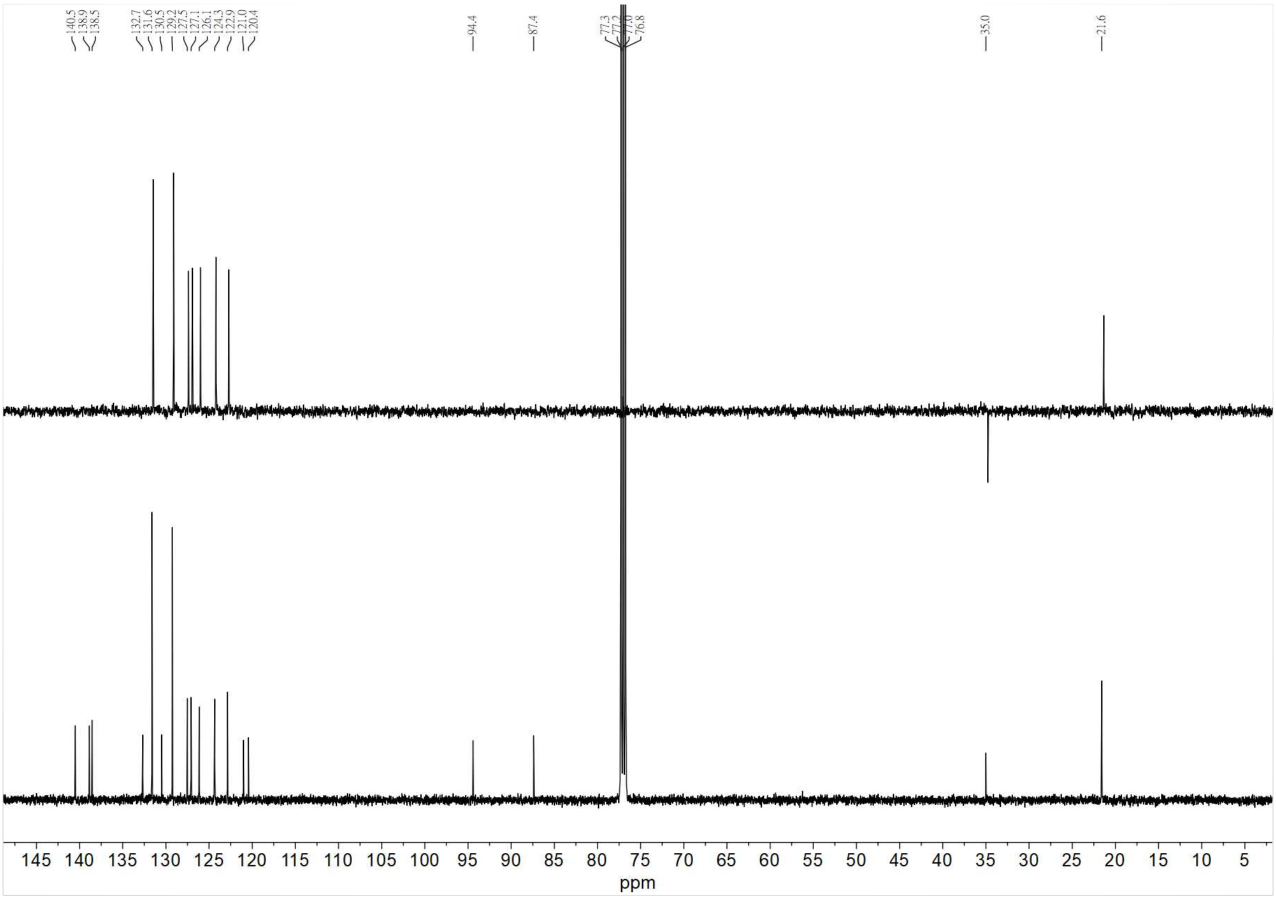

^{1}H (500 MHz) and ^{13}C (125 MHz) NMR spectra of **4c** in $CDCl_3$

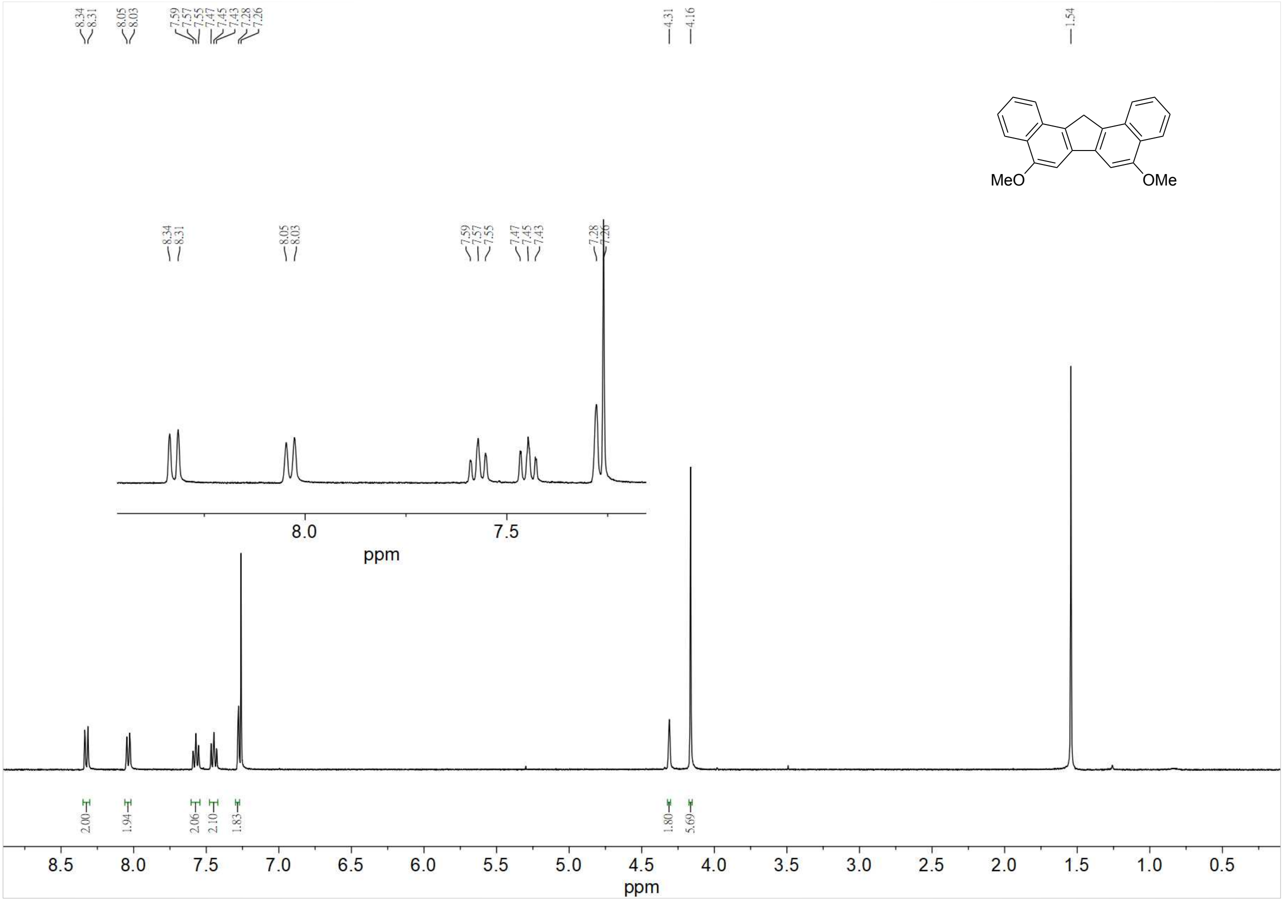

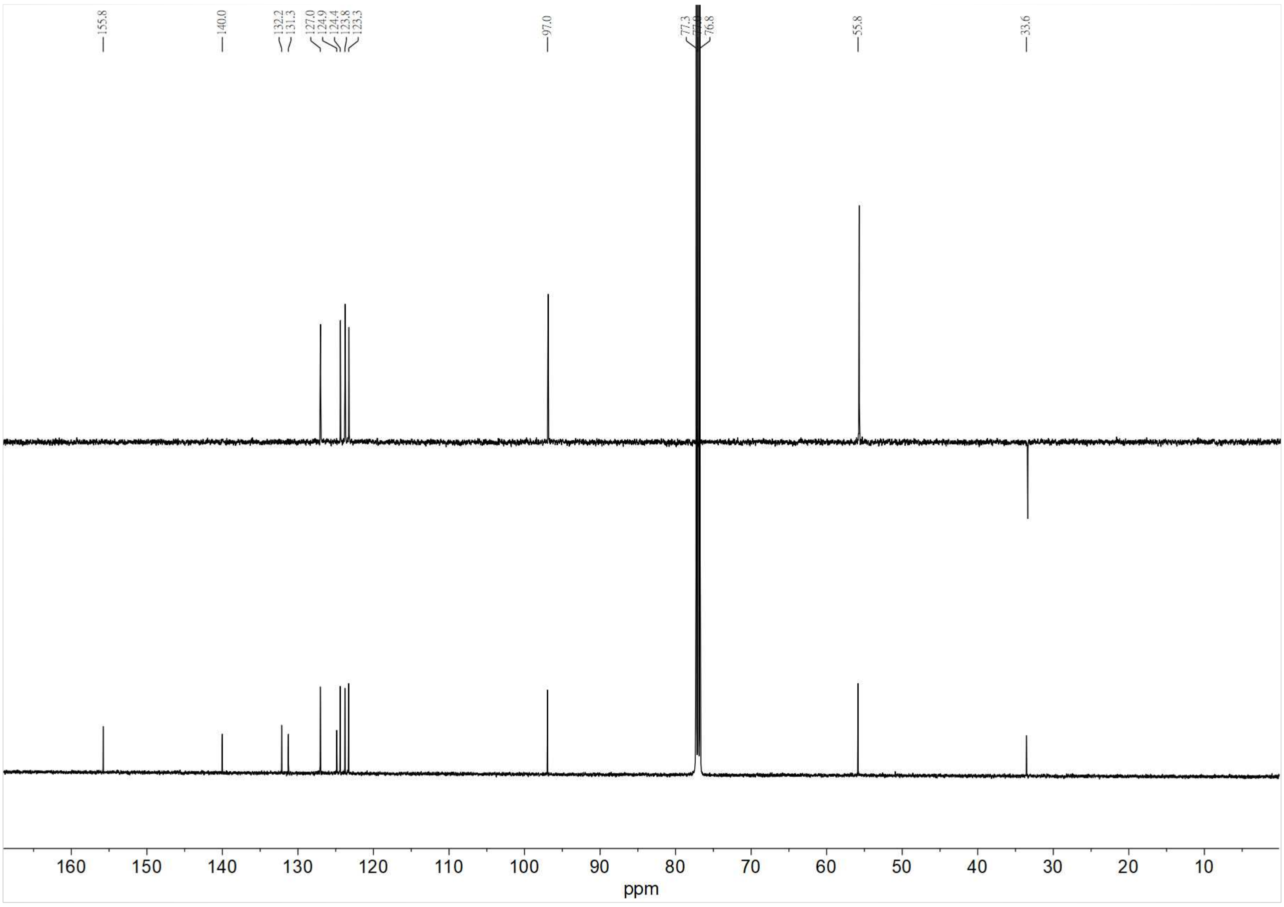

^{1}H (500 MHz) and ^{13}C (125 MHz) NMR spectra of **4d** in $CDCl_3$

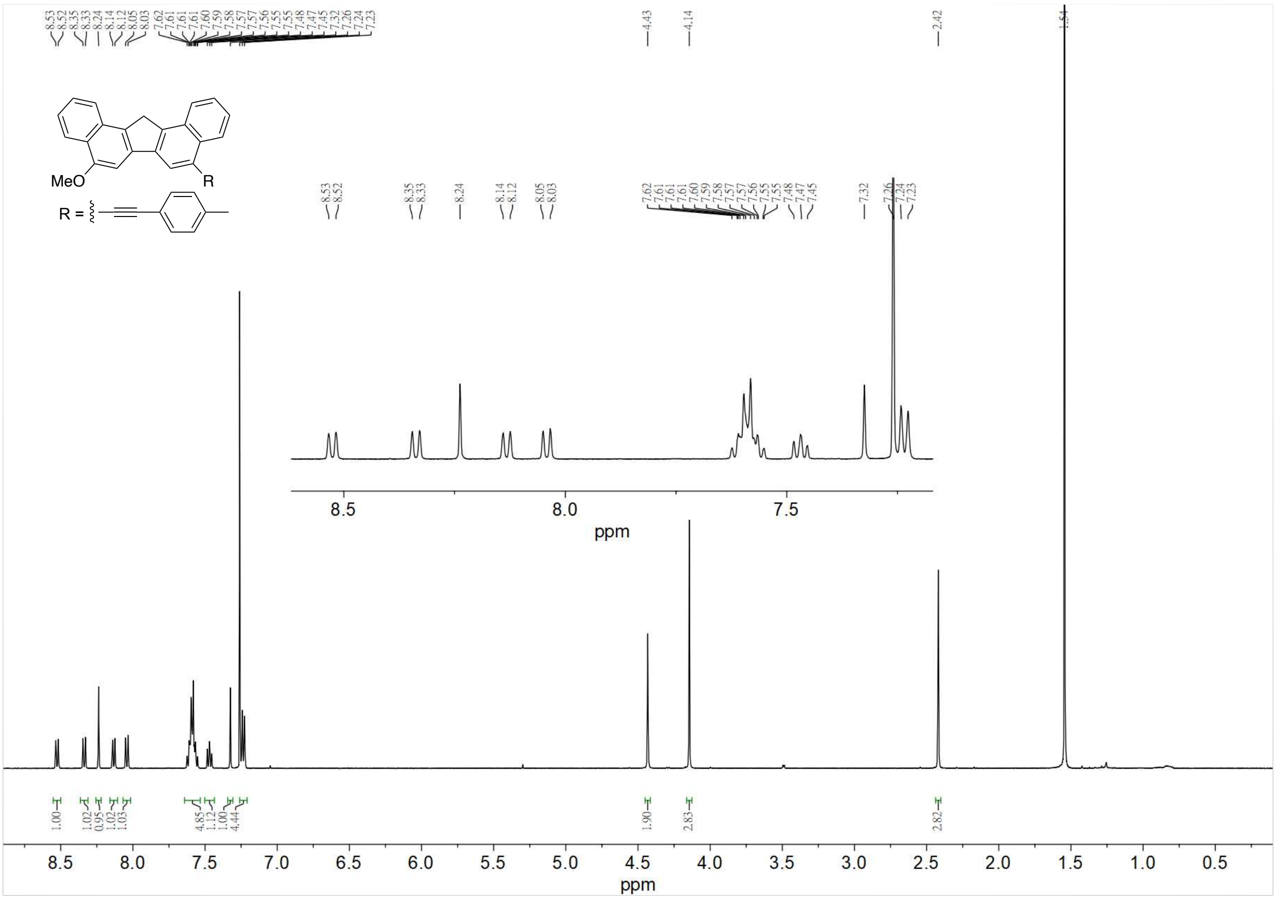

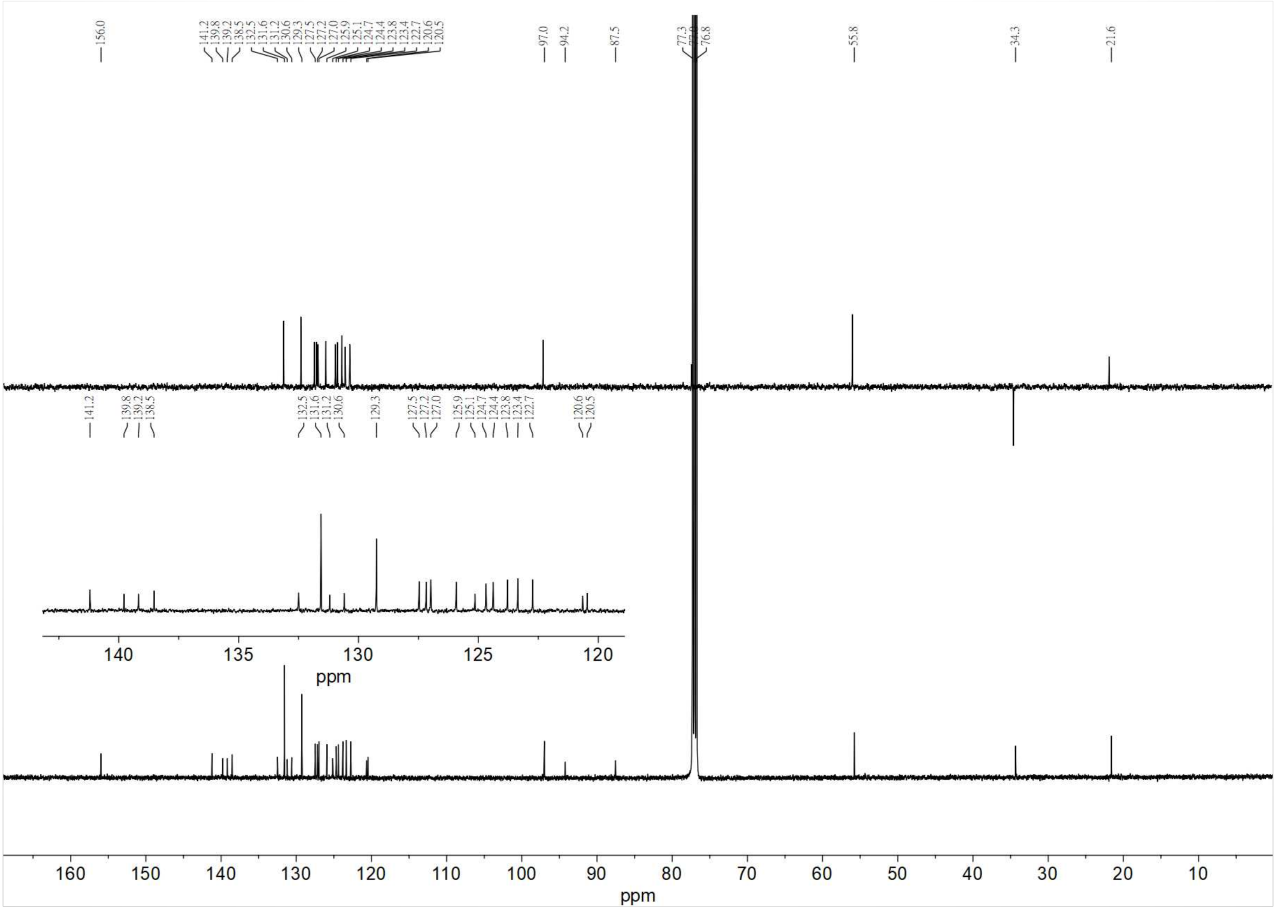

^{1}H (400 MHz) and ^{13}C (125 MHz) NMR spectra of **5** in $CDCl_3$

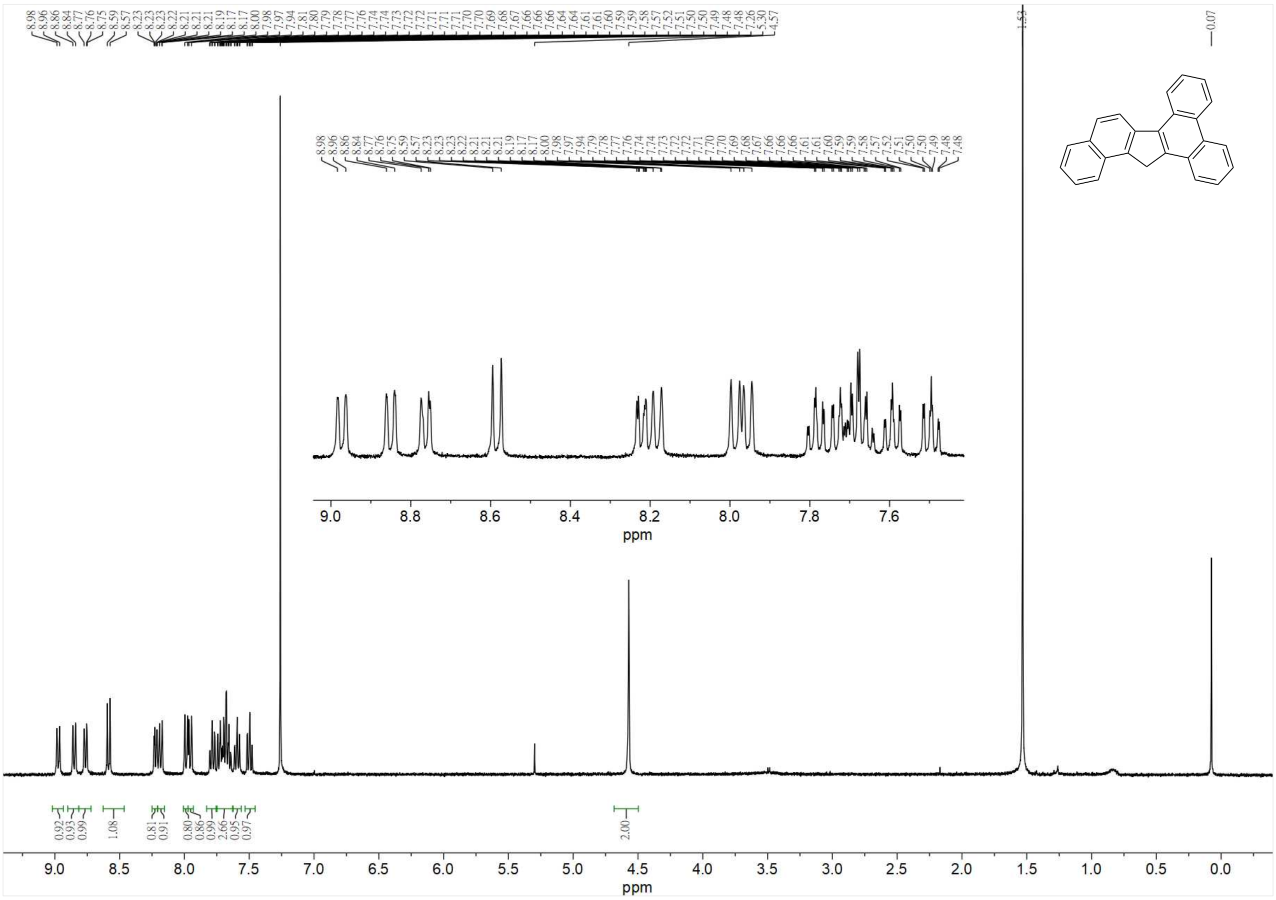

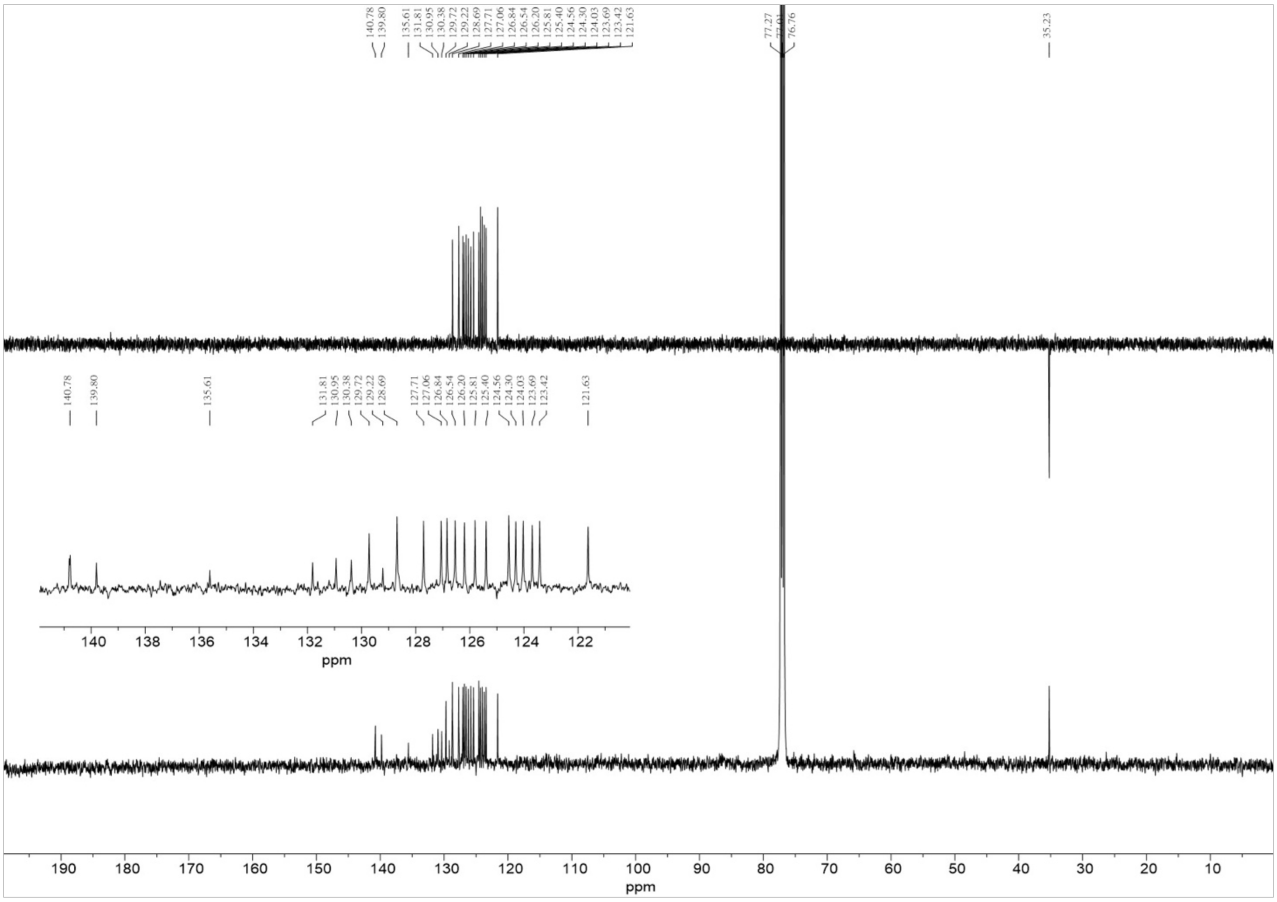

^{1}H NMR spectrum of **6** in $CDCl_3$ (400 MHz)

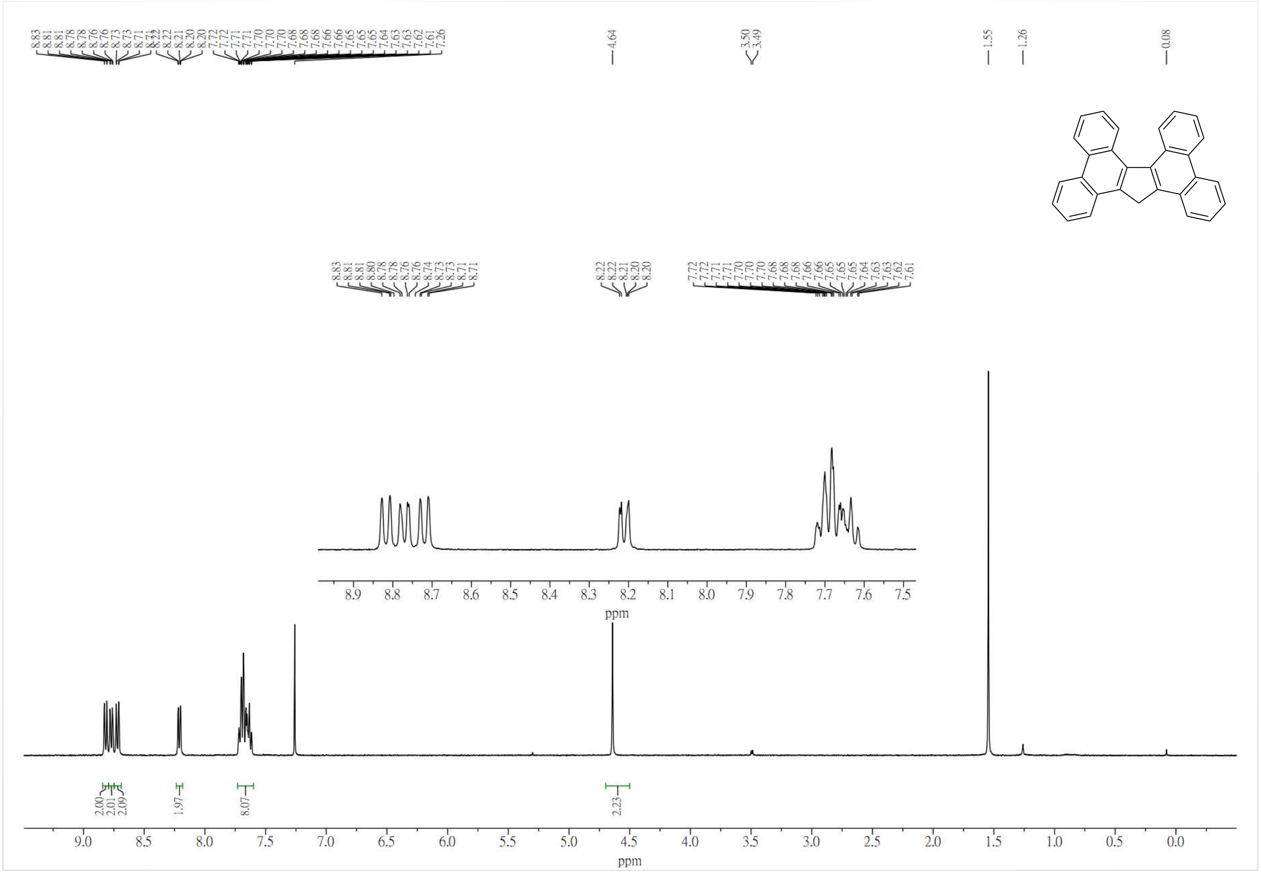

^{1}H NMR spectrum of **7a** in $C_2D_2Cl_4$ (500 MHz, 393 K)

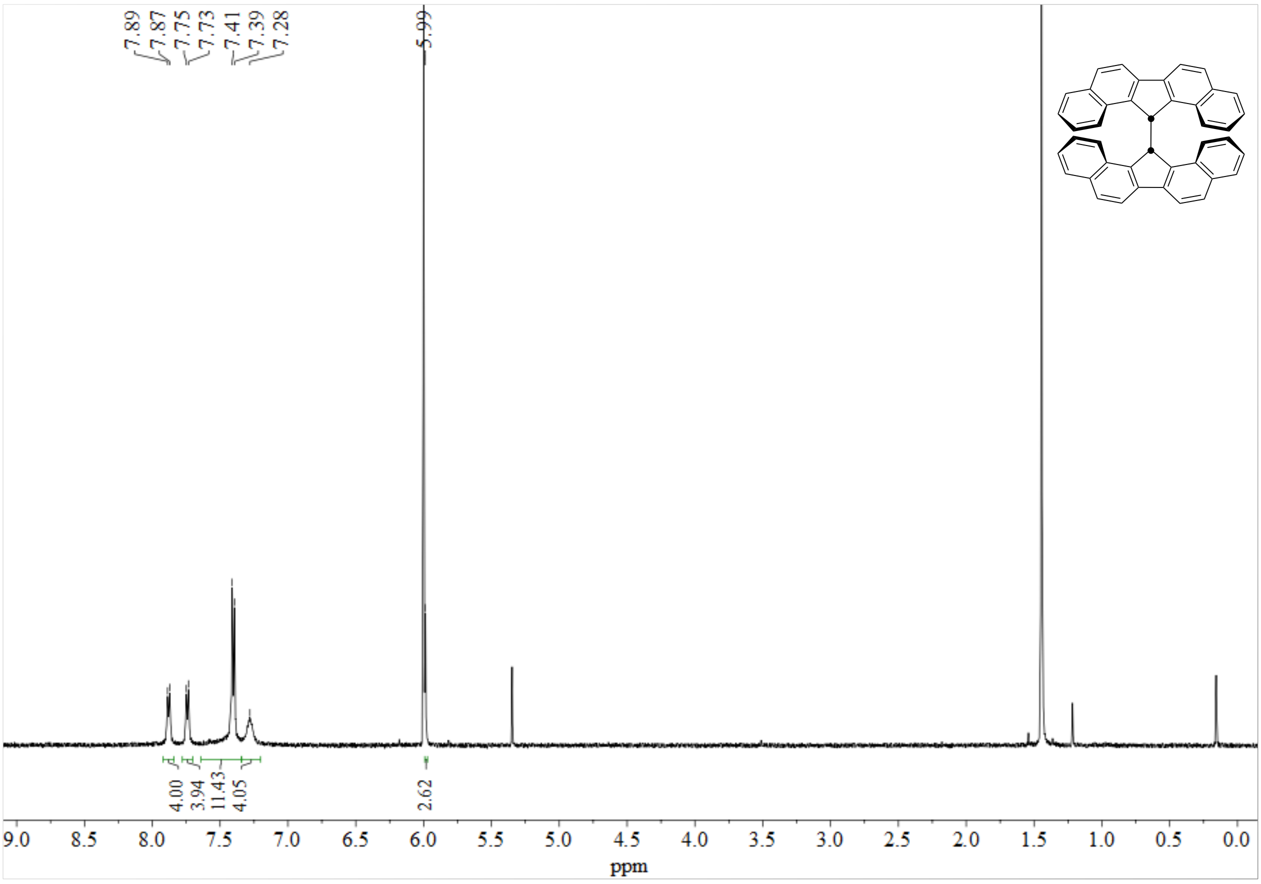

VT ^{1}H NMR spectrum of **7a** in CD_2Cl_2 (500 MHz)

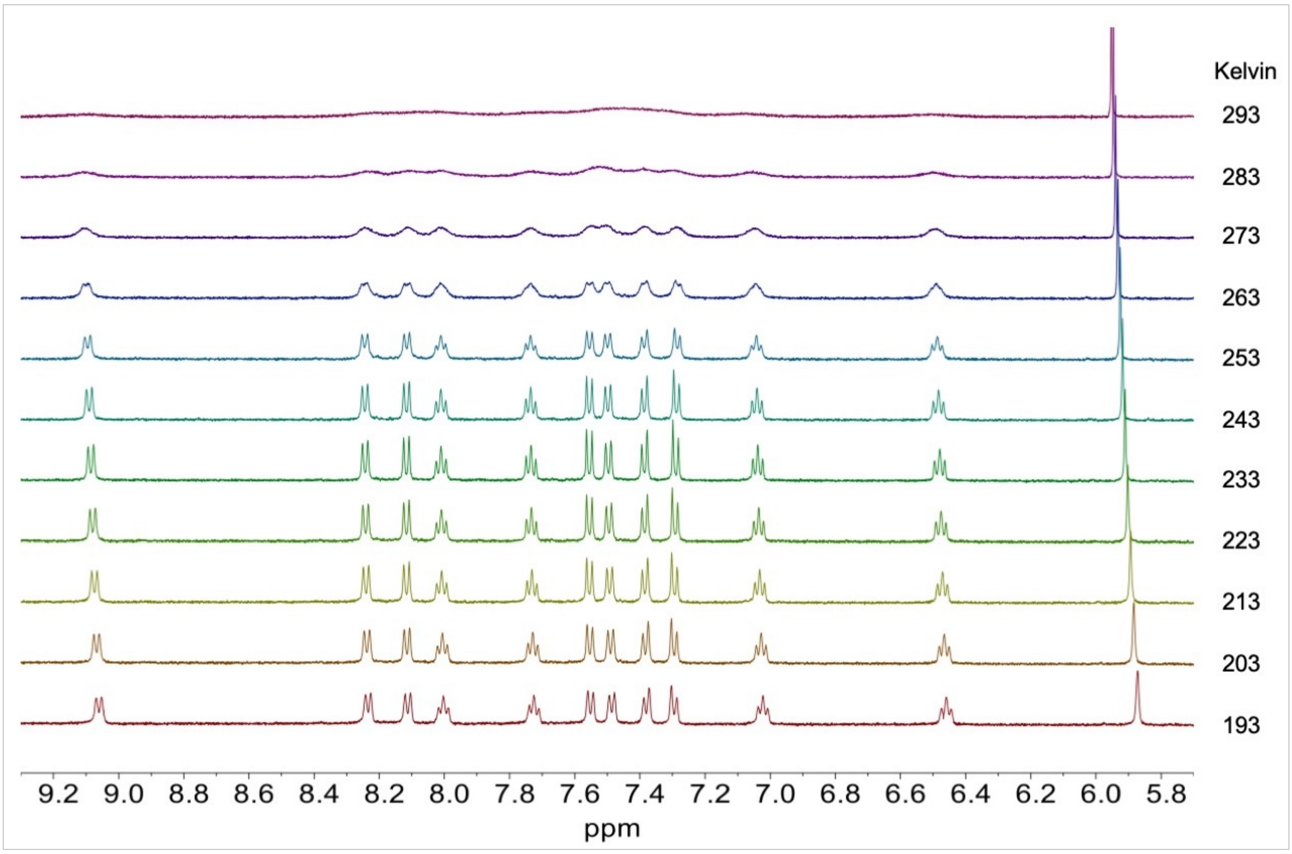

VT ^{1}H NMR spectrum of **7a** in $C_2D_2Cl_4$ (500 MHz)

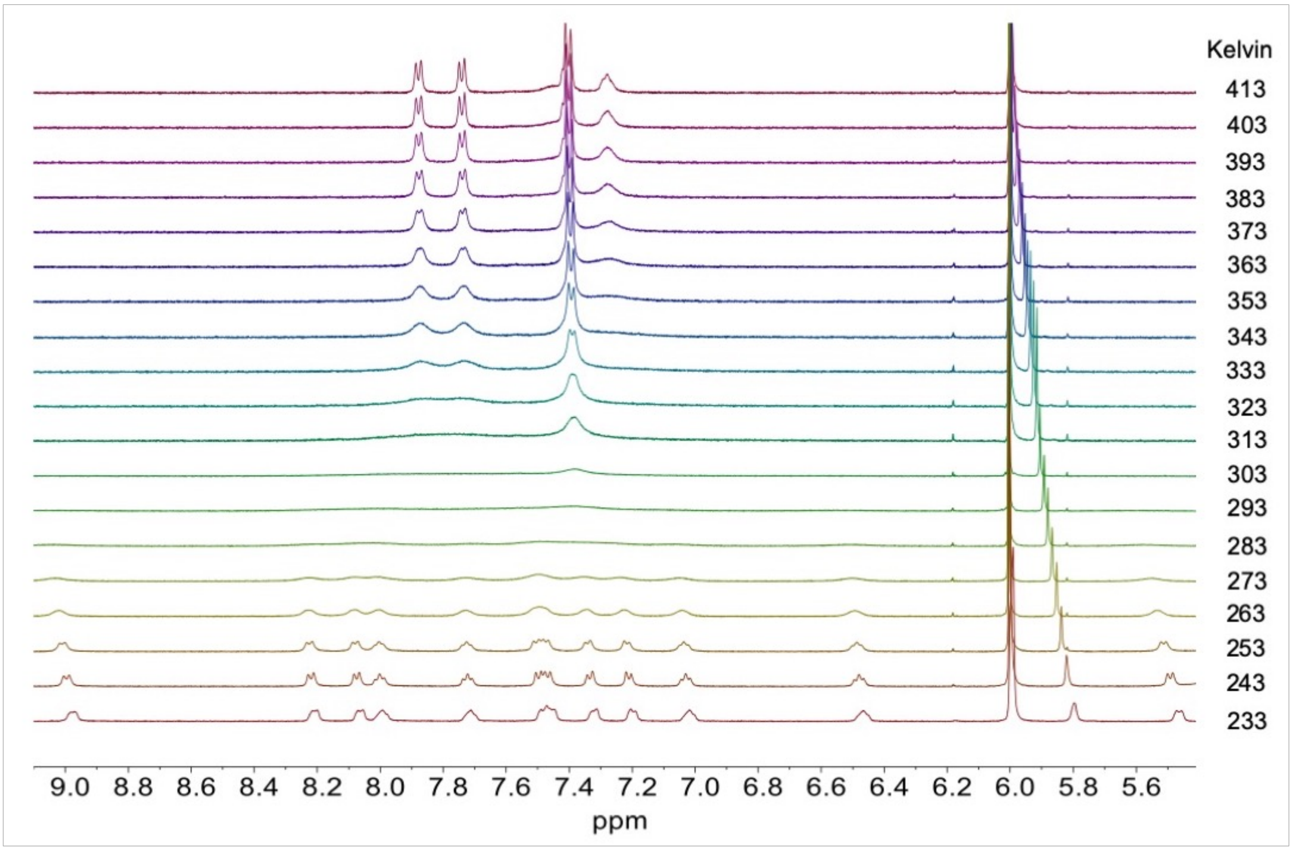

^1H NMR spectrum of **8** in CD_2Cl_2 (500 MHz, 203 K)

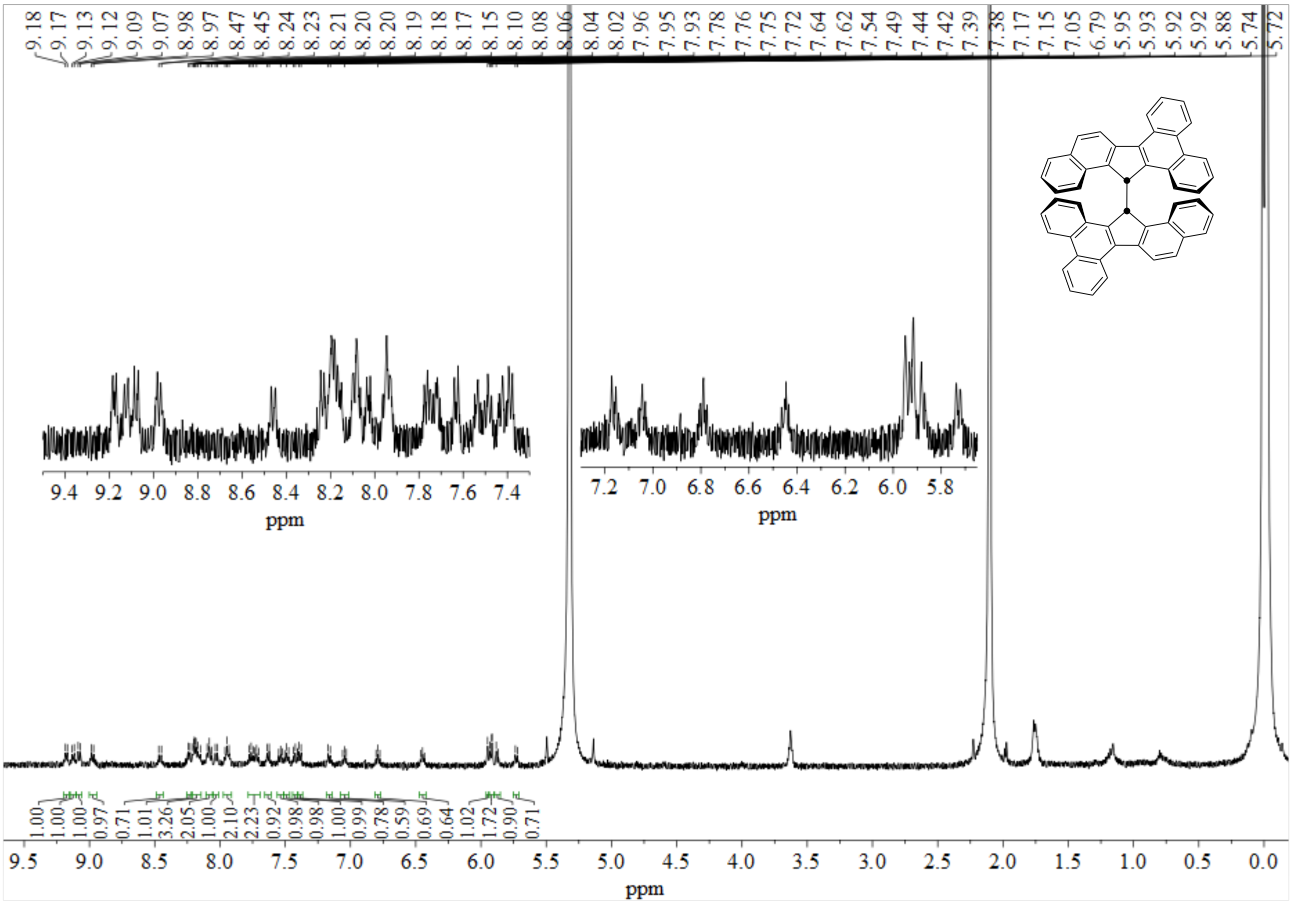

VT ^1H NMR spectrum of **8** in $CDCl_2$ (500 MHz)

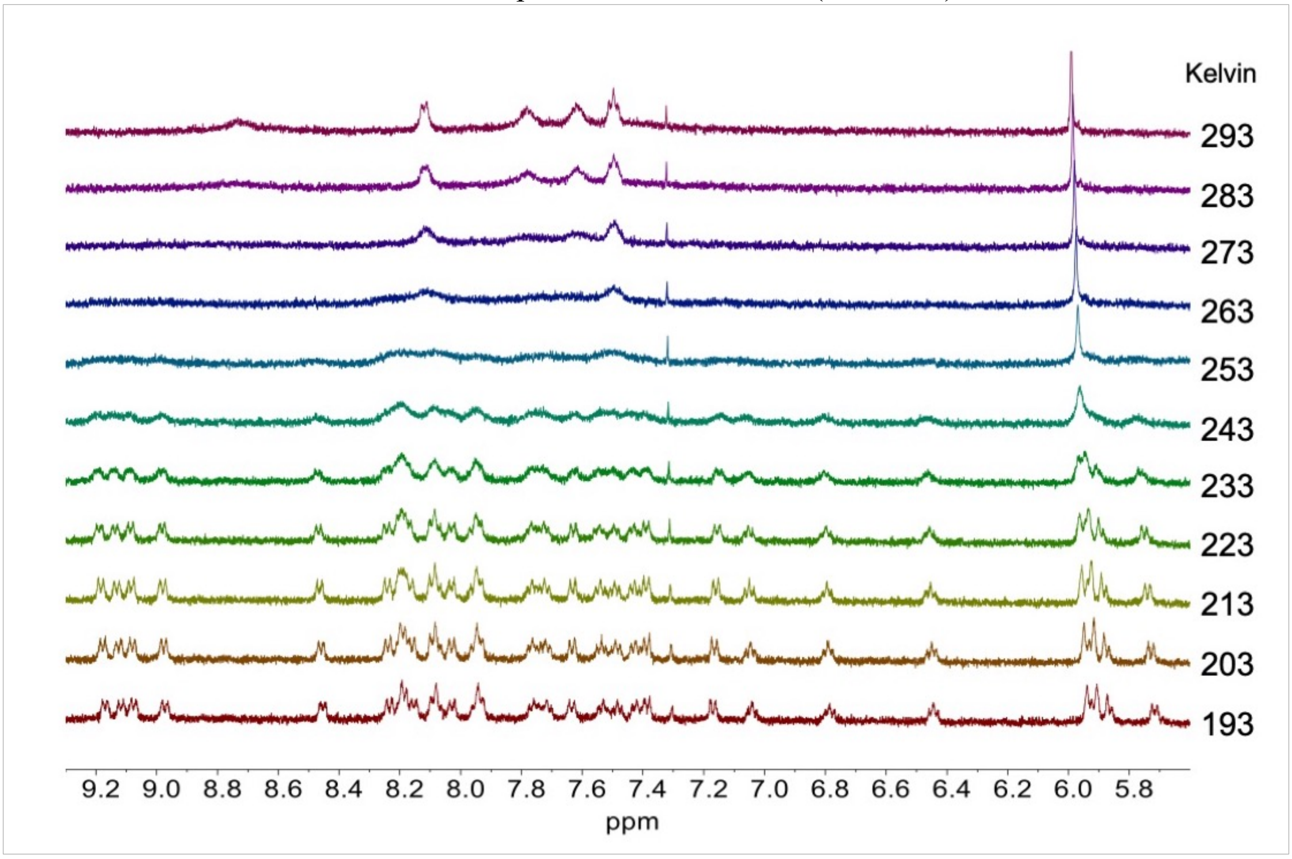

^{1}H (500 MHz, 253 K) and ^{13}C (175 MHz) NMR spectra of **9** in CD_2Cl_2

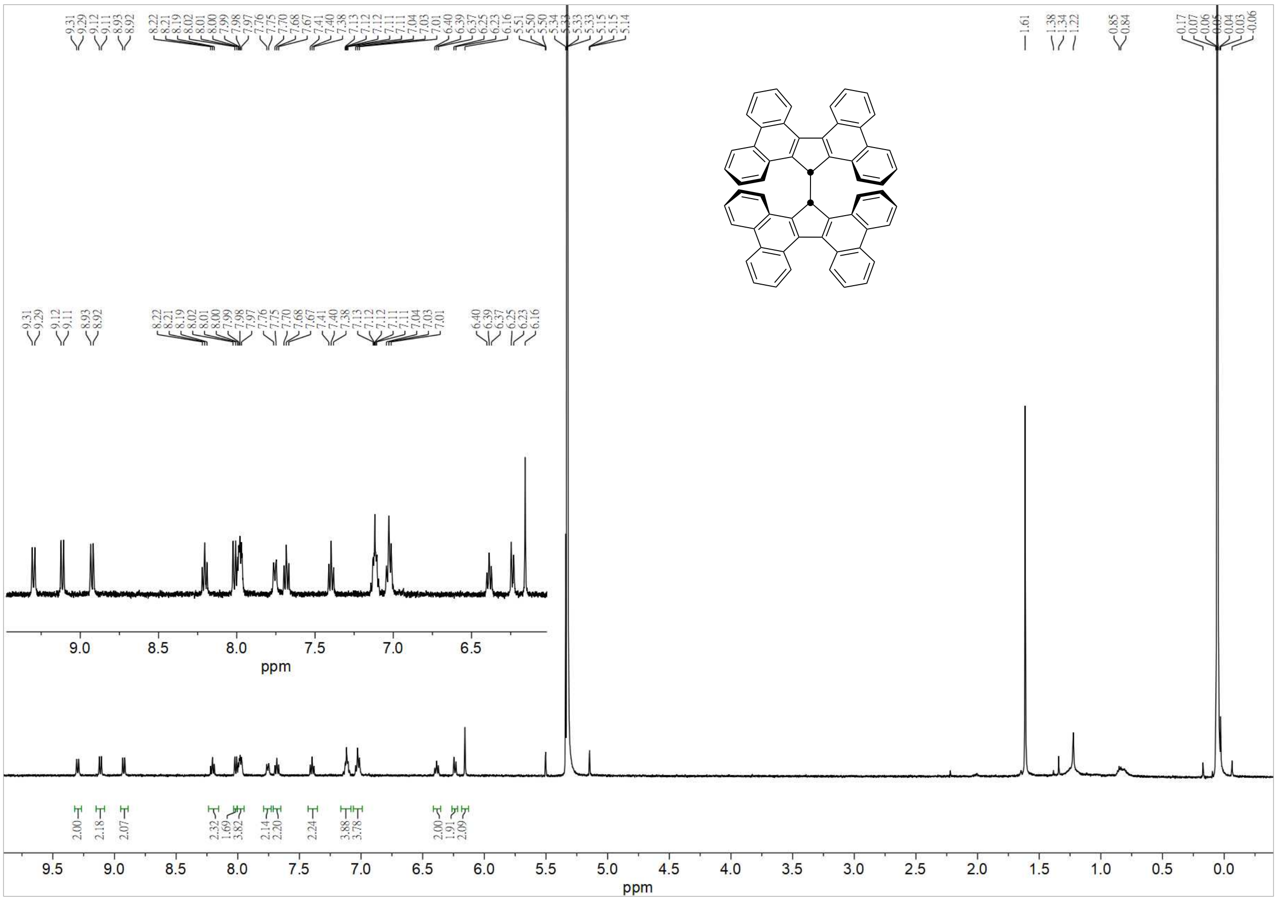

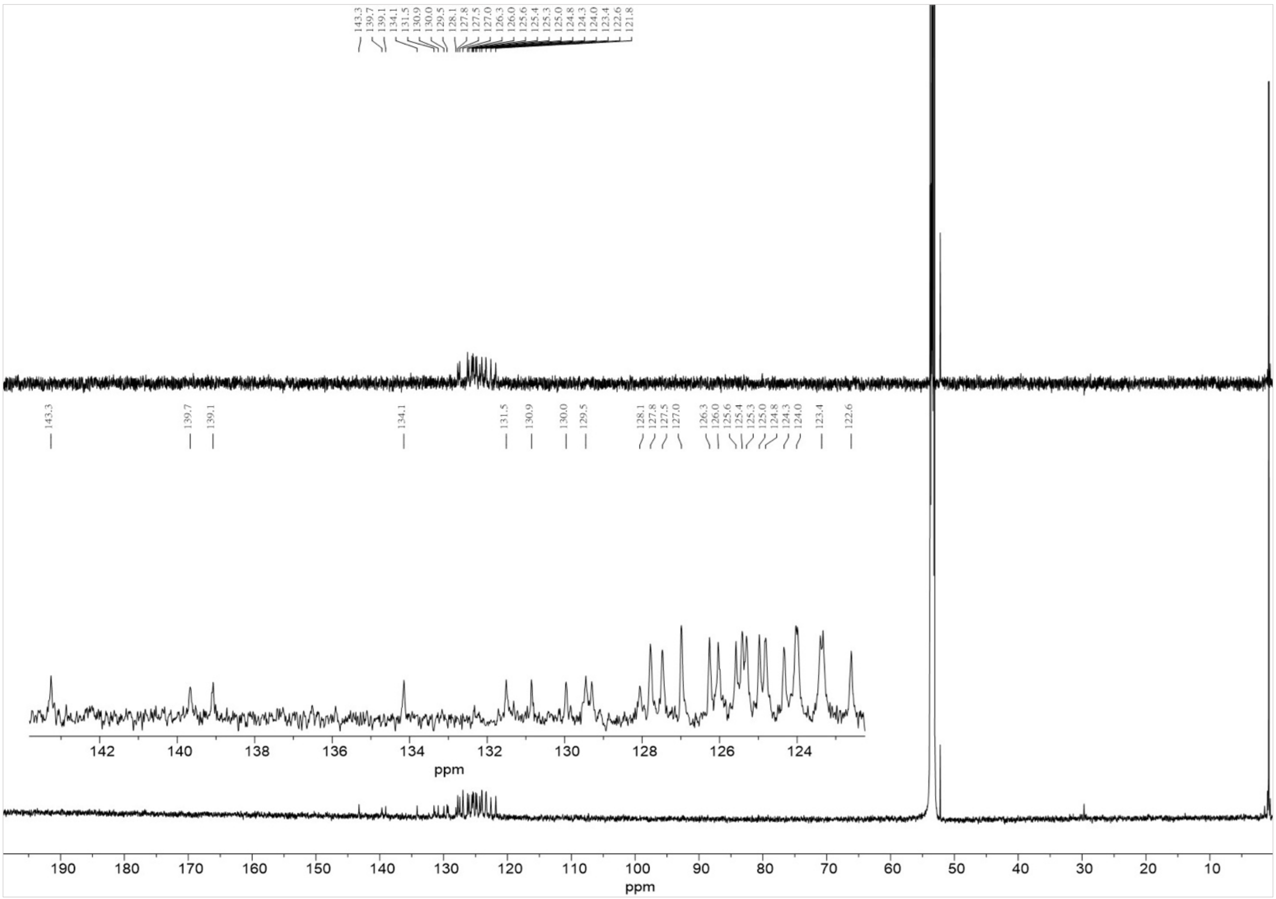

VT ^1H NMR spectrum of **9** in CD_2Cl_2 (500 MHz)

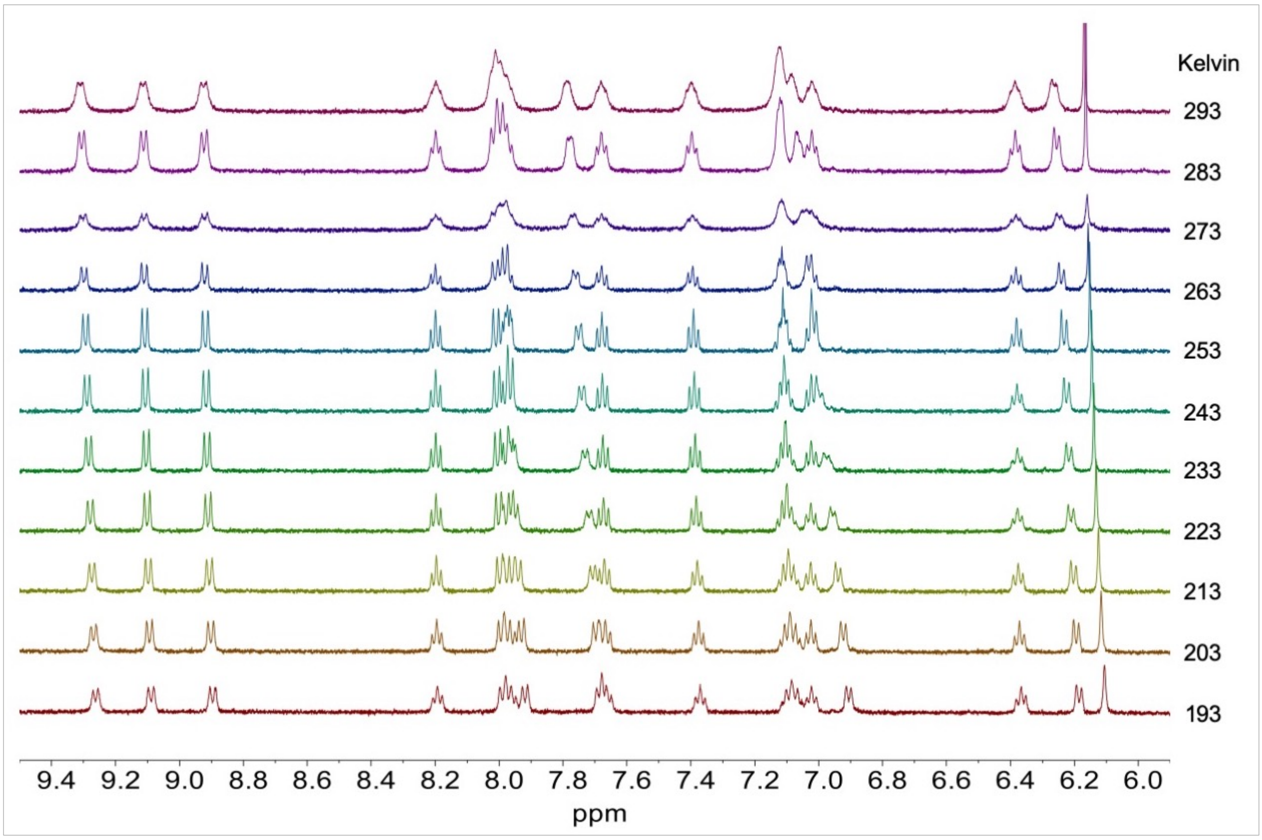

^1H NMR spectrum of **10** in $CDCl_3$ (400 MHz)

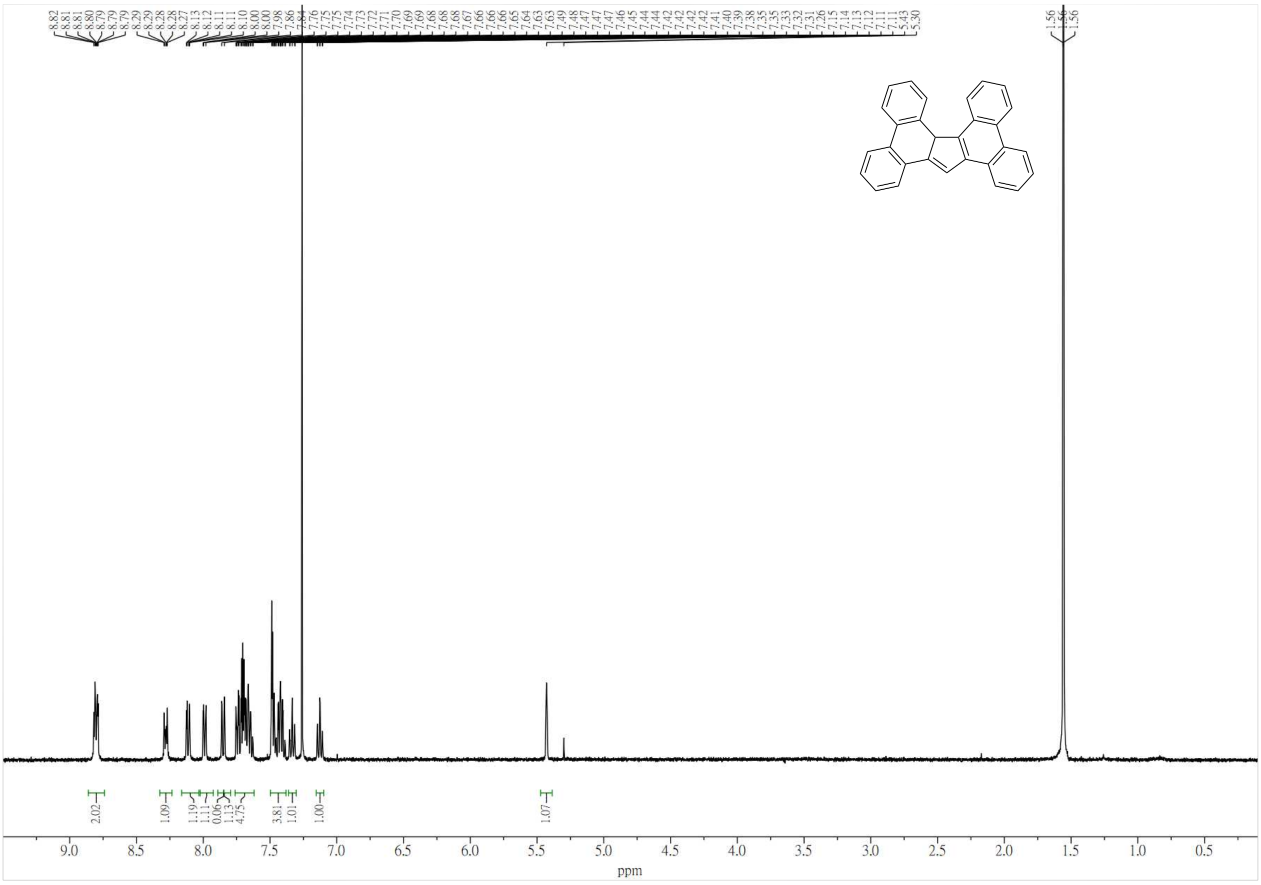

^{1}H (500 MHz) and ^{13}C (125 MHz) NMR spectra of **S4** in $CDCl_3$

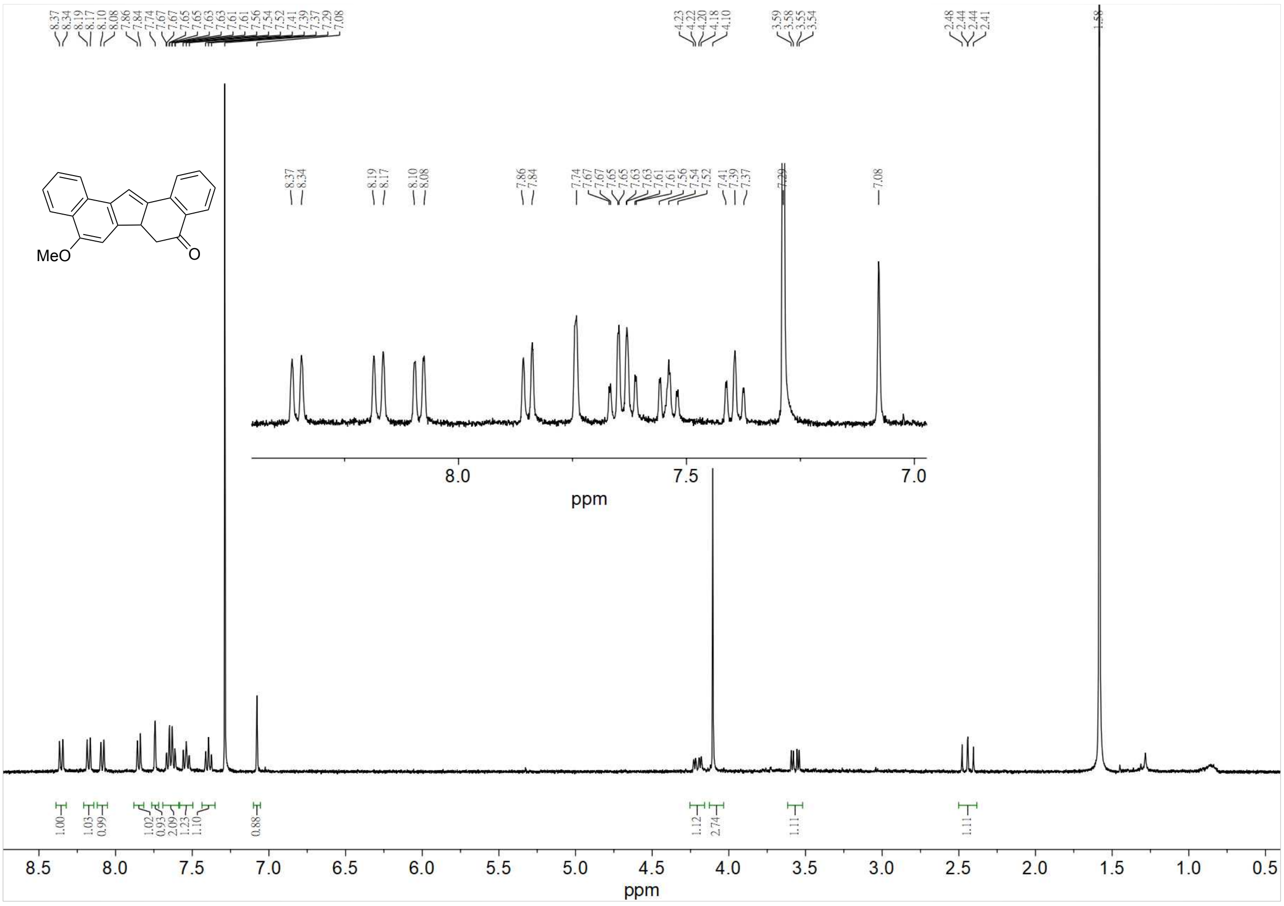

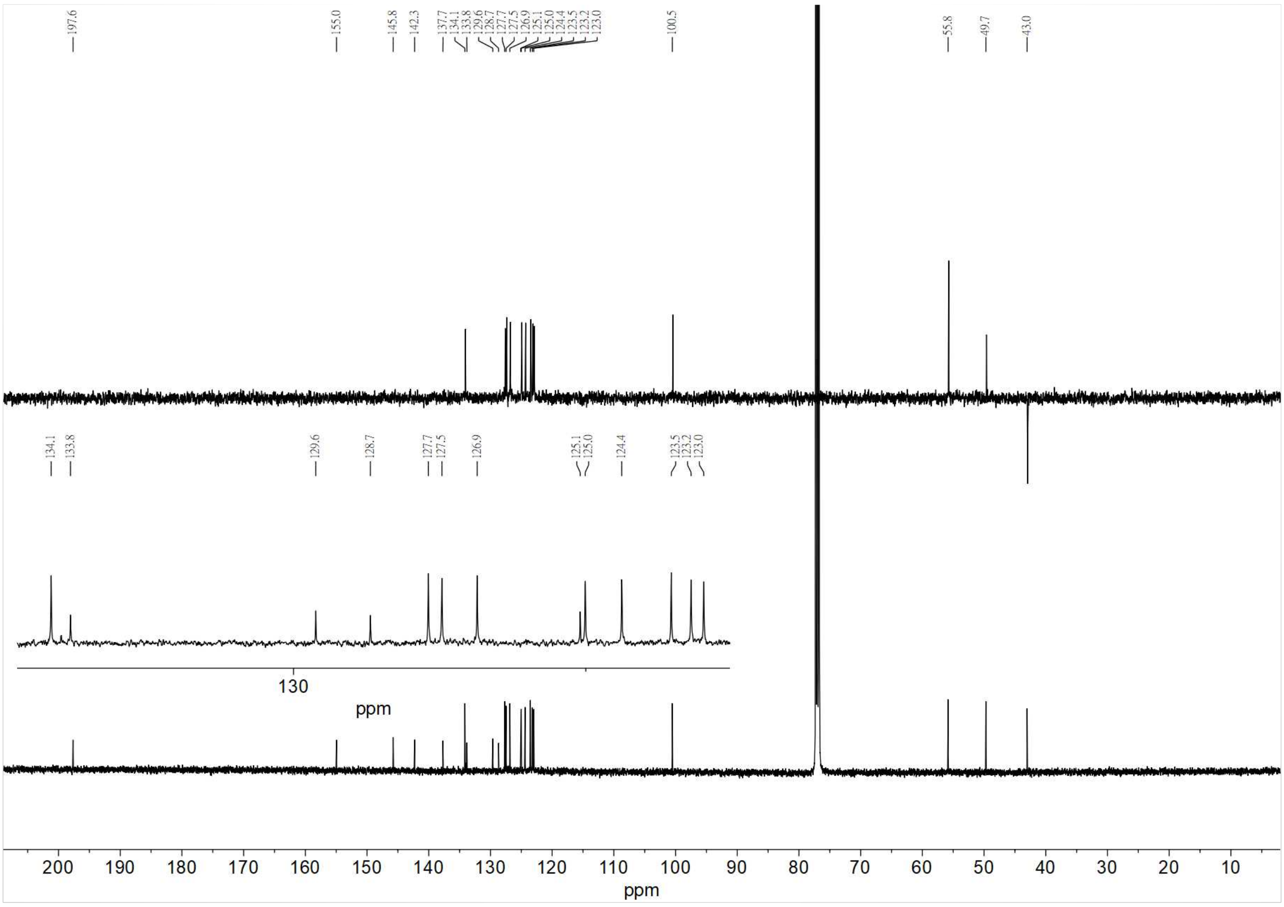

^{1}H NMR spectrum of **S5** (400 MHz, $CDCl_3$)

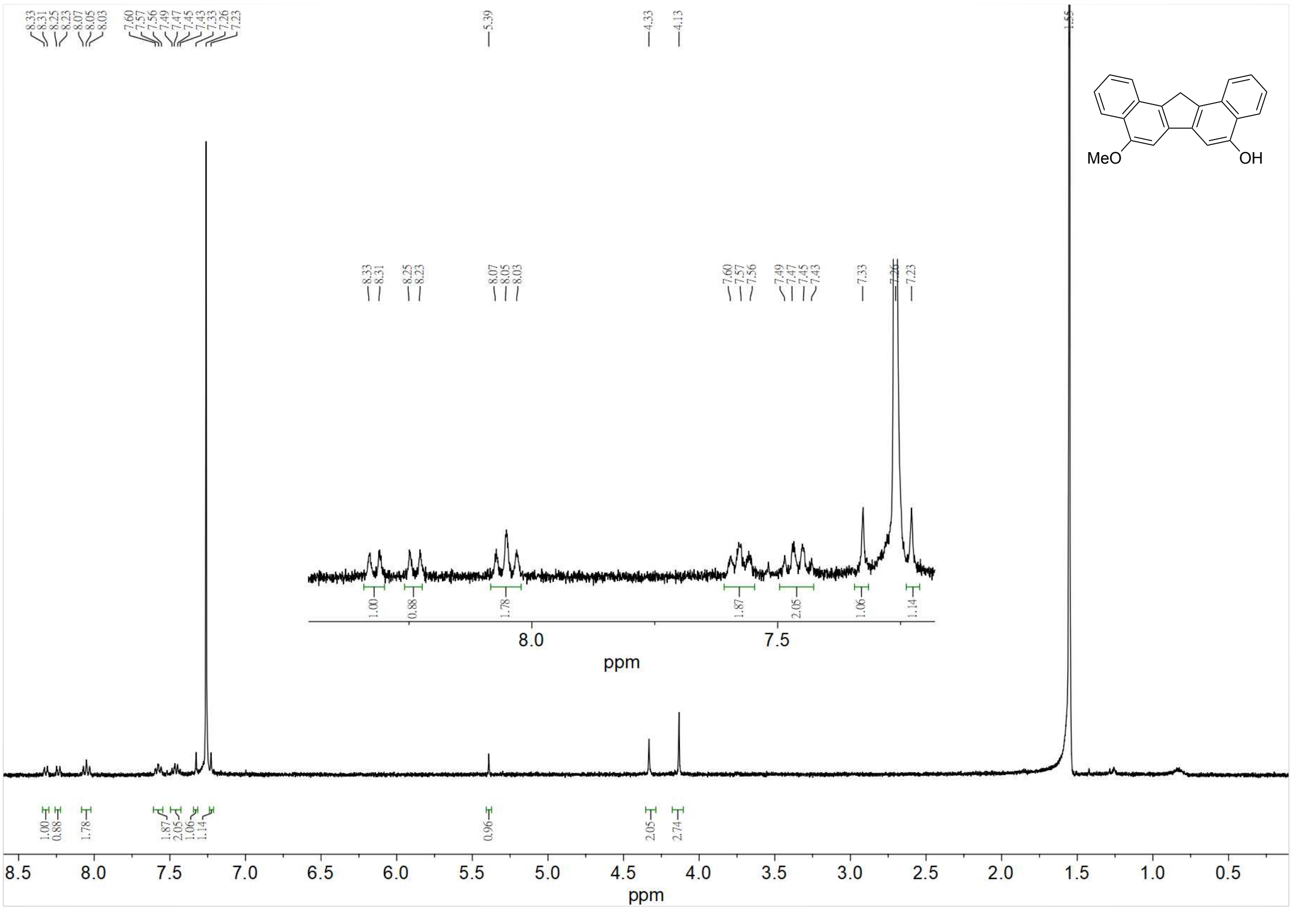

^{1}H (500 MHz) and ^{13}C (125 MHz) NMR spectra of **S6** in $CDCl_3$

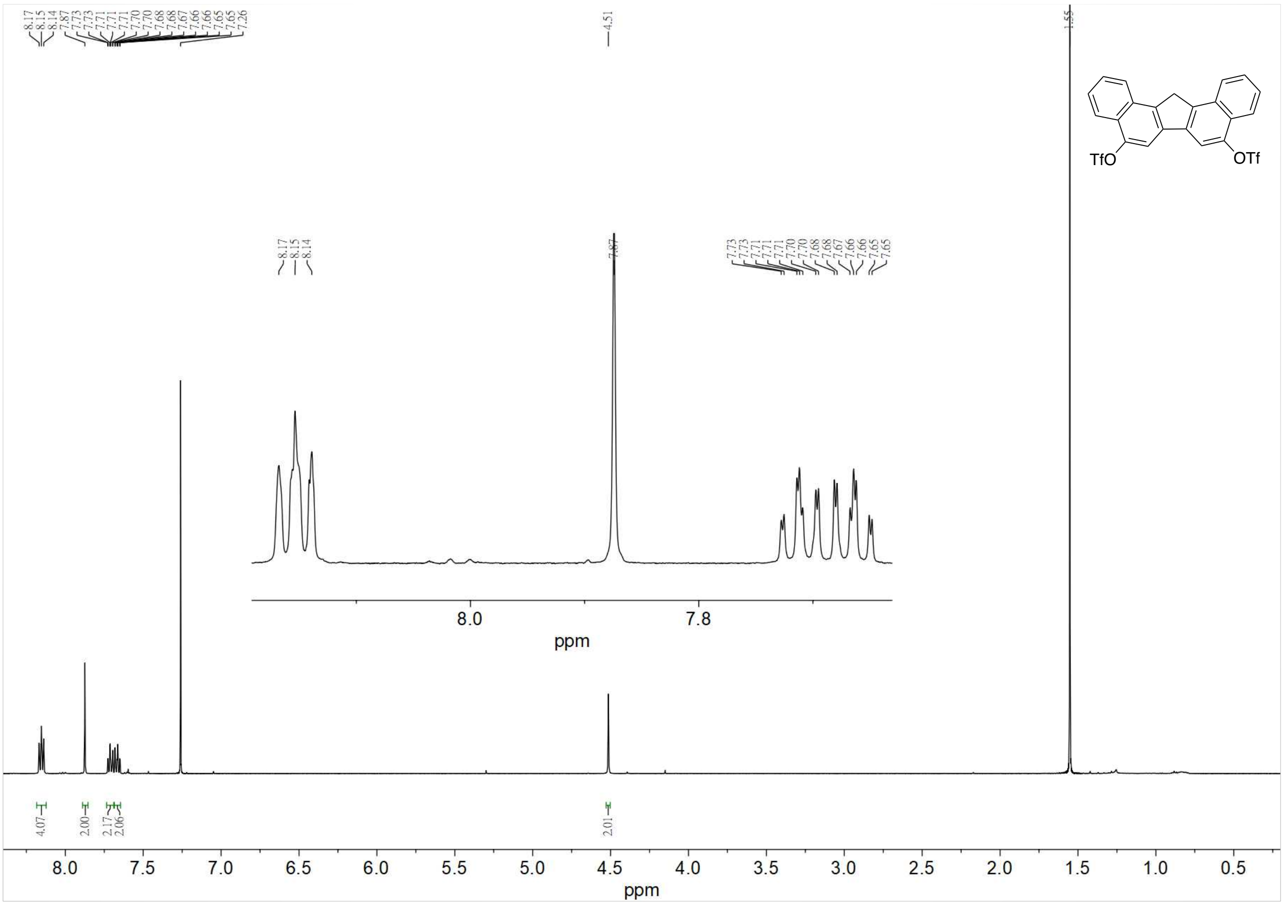

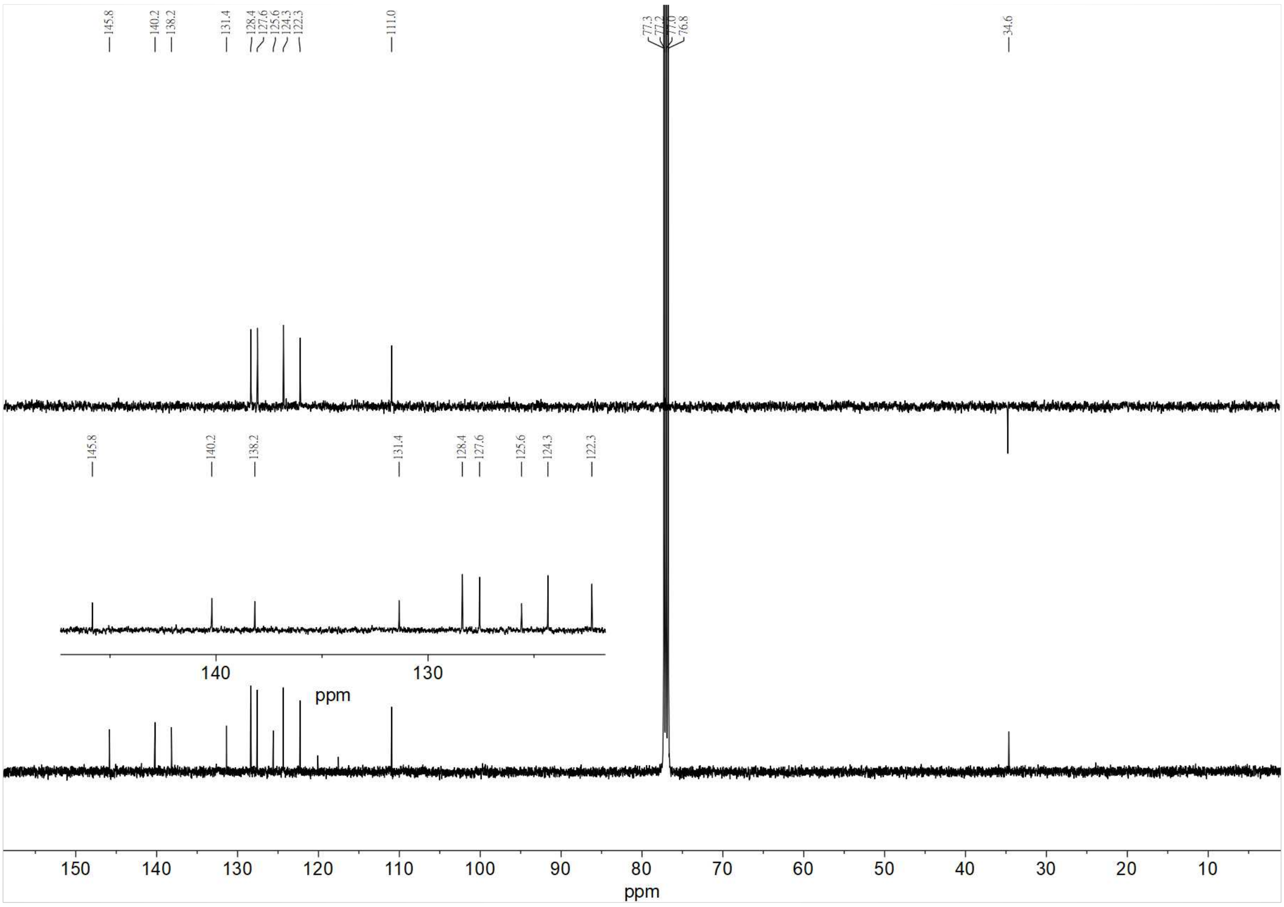

^{1}H (500 MHz) and ^{13}C (125 MHz) NMR spectra of **S7** in $CDCl_3$

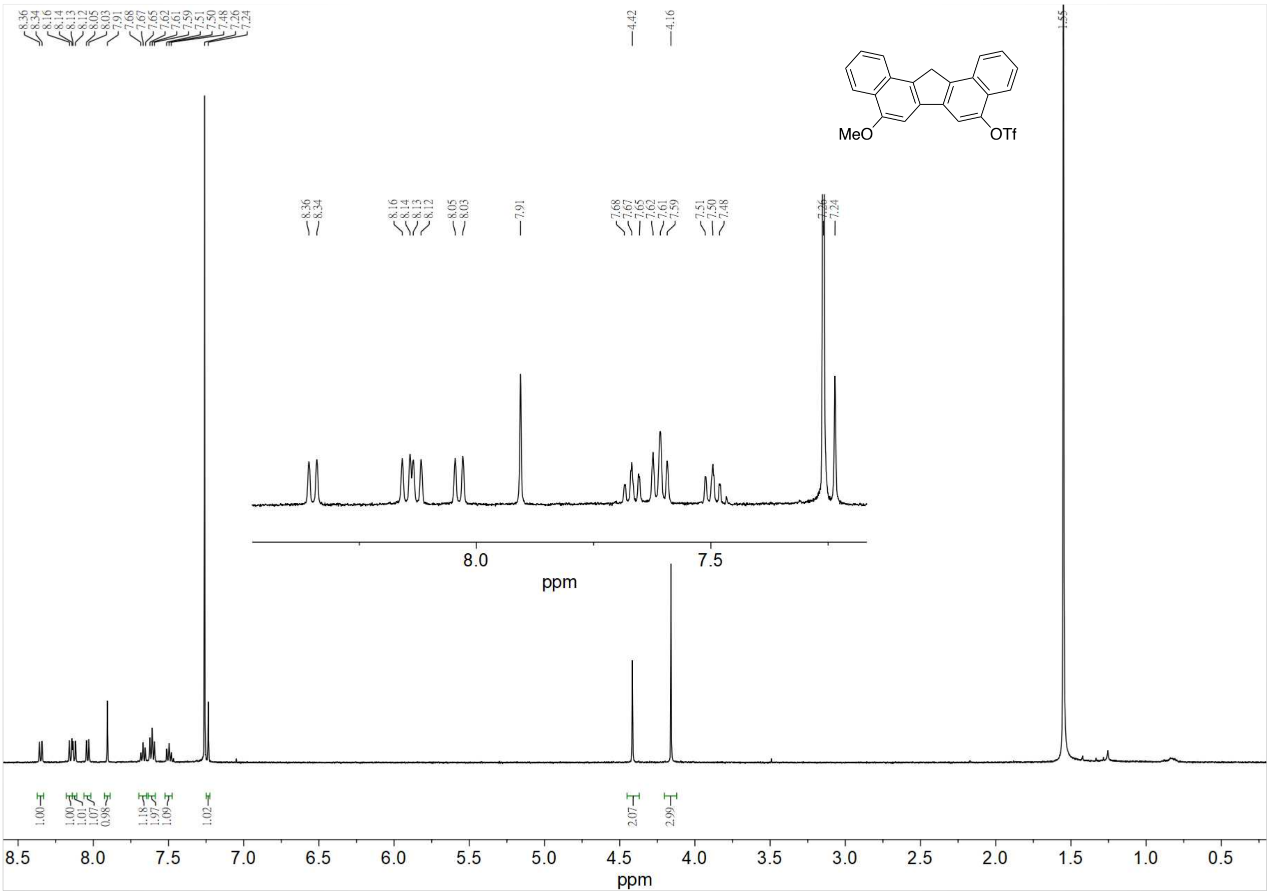

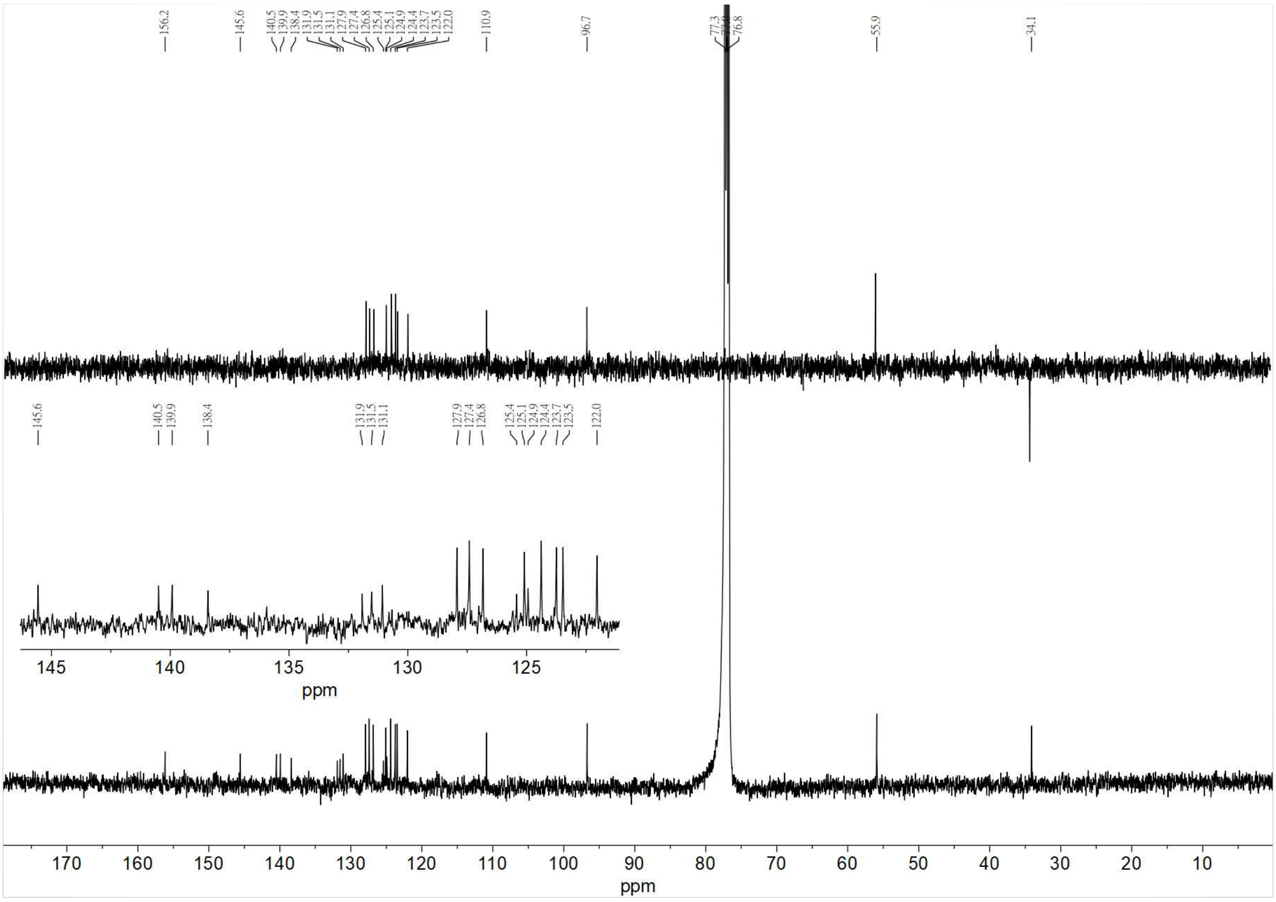

^1H (400 MHz) and ^{13}C (125 MHz) NMR spectra of **S8** in $CDCl_3$

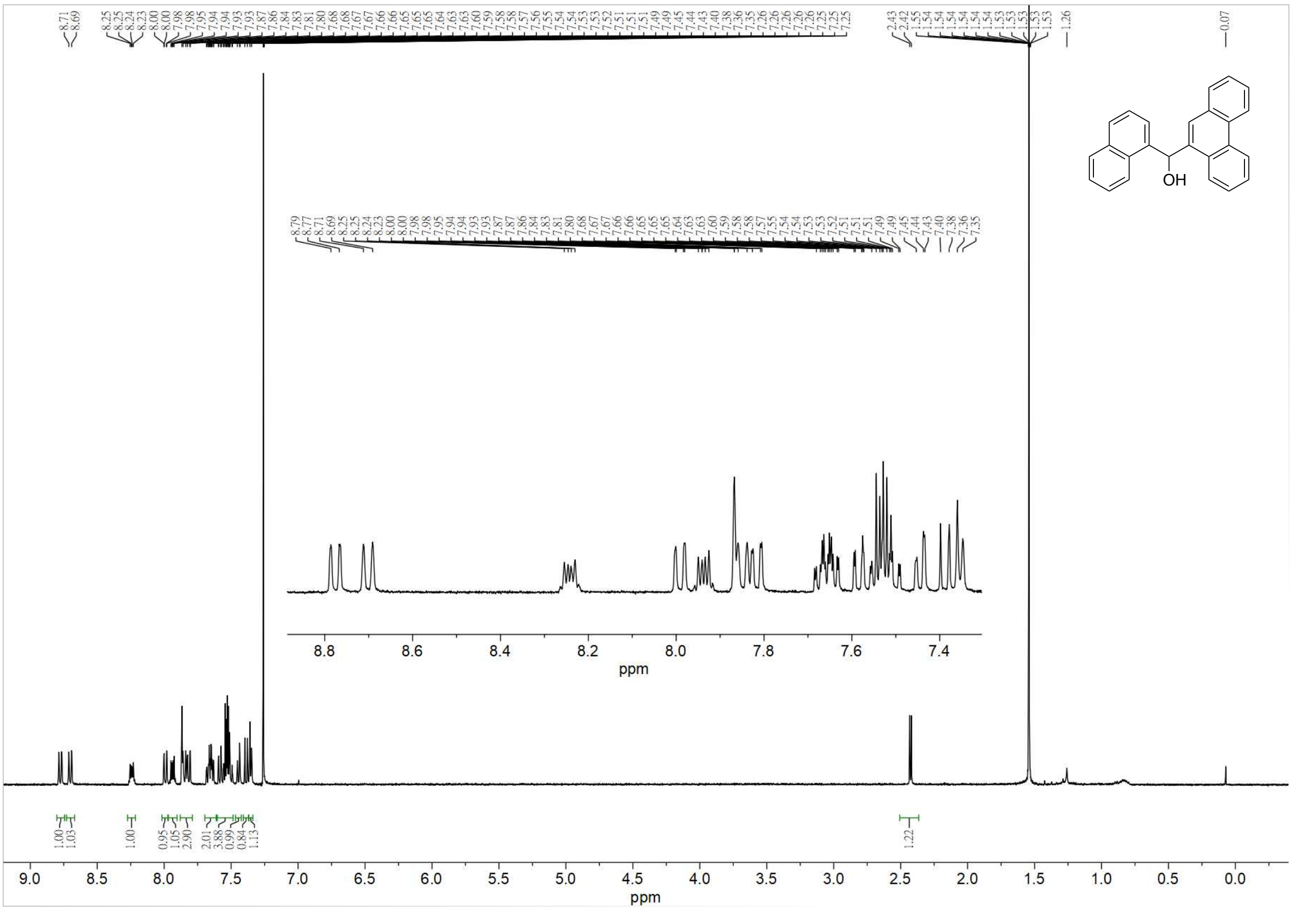

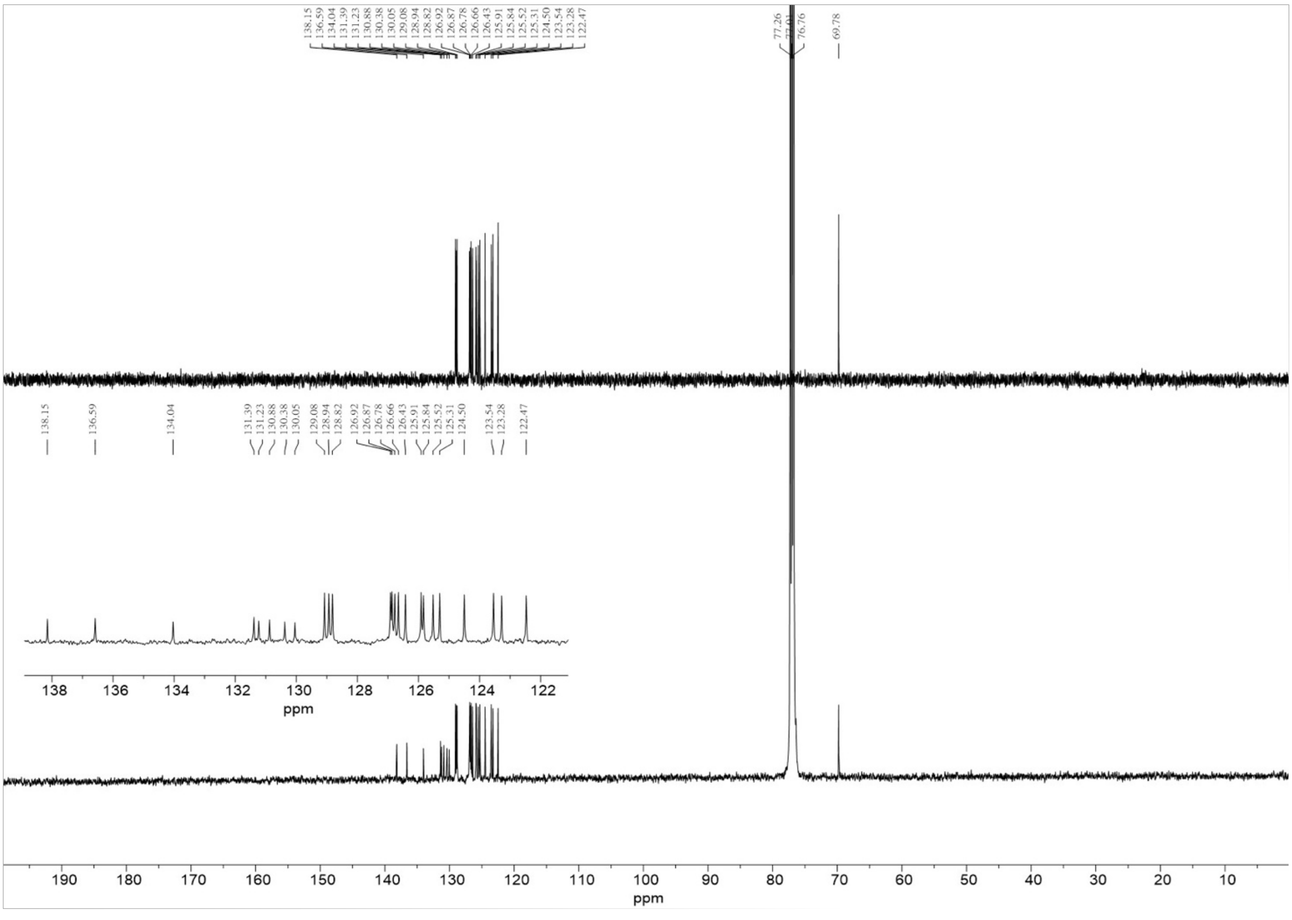

H. Reference

S1 Lin, F. *et al.* Nature of the entire range of rare earth metal-based cationic catalysts for highly active and syndioselective styrene polymerization. *ACS Catal.* **6**, 176−185 (2016).

S2 Wentrup, C.; Regimbald-Krnel, M. J.; Müller, D. & Comba, P. A thermally populated, perpendicularly twisted alkene triplet diradical. *Angew. Chem. Int. Ed.* **55**, 14600−14605 (2016).

S3 Olah, G. A. *et al.* Crowded hydrocarbons. 2. Conformational study of 9,9'-bifluorenyls by dynamic nuclear magnetic resonance spectroscopy. *J. Org. Chem.* **46**, 1761–1764 (1981).

S4 Cerchia, C. *et al.* Discovery of novel naphthylphenylketone and naphthylphenylamine derivatives as cell division cycle 25B (CDC25B) phosphatase inhibitors: Design, synthesis, inhibition mechanism, and in vitro efficacy against melanoma cell lines. *J. Med. Chem.* **62**, 7089−7110 (2019).

S5 Schröder, K. *et al.* Tetrabenzo[*a,c,g,i*]fluorenyllithium and η^5-tetrabenzo[*a,c,g,i*]fluorenyl-titanium complexes. *Organometallics* **25**, 3824–3836 (2006).

S6 Wei, Y.-C. *et al.* Excited-state THz vibrations in aggregates of Pt^{II} complexes contribute to the enhancement of near-infrared emission efficiencies. *Angew. Chem. Int. Ed.* **135**, e202300815 (2013)

S7 Lu, C.-H. *et al.* Generation of intense supercontinuum in condensed media. *Optica* **1**, 400−406. (2014).

S8 Lu, C.-H. *et al.* Greater than 50 times compression of 1030 nm Yb:KGW laser pulses to single-cycle duration. *Opt. Express* **27**, 15638−15648 (2019).
