# Supplementary material for: Synthesis, structural analysis, and properties of highly twisted alkenes 13,13’-bis(dibenzo[a,i]fluorenylidene) and its derivatives
Source: Nat Commun. 2023 Aug 28;14:5248. doi: 10.1038/s41467-023-40990-8 (PMC10462764; doi:10.1038/s41467-023-40990-8)
Supplement: Supplementary file 3 — Description of Additional Supplementary Files [file 41467_2023_40990_MOESM3_ESM.pdf]

Description of Additional Supplementary Files

File Name: Supplementary Data 1
Description: The Cartesian coordinates of optimized structures
