# Supplementary material for: Synthesis, structural analysis, and properties of highly twisted alkenes 13,13’-bis(dibenzo[a,i]fluorenylidene) and its derivatives
Source: Nat Commun. 2023 Aug 28;14:5248. doi: 10.1038/s41467-023-40990-8 (PMC10462764; doi:10.1038/s41467-023-40990-8)
Supplement: Supplementary file 4 — Supplementary Data 1 [file 41467_2023_40990_MOESM4_ESM.docx]

**Coordinates of optimized structures**

**B3LYP-D3/6-311++G**//6-31G****

*S_0_*-**1a**

0 1

C 21.428671 0.372742 12.423821

C 21.566306 0.421971 13.822858

C 21.928870 1.381361 11.583665

H 21.815473 1.304873 10.506468

C 22.570259 2.461221 12.149063

H 22.992360 3.239971 11.519601

C 22.656443 2.608814 13.559931

C 22.101650 1.604786 14.434026

C 23.258940 3.766729 14.121256

H 23.687789 4.503830 13.447184

C 23.282557 3.969497 15.481196

H 23.741567 4.861606 15.896452

C 22.674482 3.022276 16.335702

H 22.650728 3.199996 17.406817

C 22.100314 1.877810 15.826889

H 21.630278 1.180170 16.503934

C 20.696795 -0.836251 12.065634

C 20.902354 -0.768966 14.385817

C 20.354598 -1.313627 10.789541

C 20.366295 -1.531873 13.242708

C 20.800255 -1.109447 15.742835

C 19.678080 -2.508883 10.685431

H 20.615075 -0.744259 9.902391

C 19.791960 -2.843226 13.144950

C 19.570718 -1.480331 16.468610

C 21.901241 -1.166867 16.722966

H 19.375599 -2.885182 9.711909

C 19.400210 -3.298637 11.833830

C 19.607577 -3.738233 14.231039

C 18.184089 -1.427633 16.103027

C 19.919953 -1.739484 17.806323

C 21.357900 -1.555116 17.960993

C 23.326516 -1.089912 16.575433

C 18.765501 -4.562253 11.694976

C 19.007814 -4.966330 14.056685

H 19.953531 -3.457426 15.214654

C 17.684085 -0.971889 14.855265

C 17.221666 -1.841281 17.094276

C 18.967592 -2.093109 18.776543

C 22.150778 -1.733146 19.106864

C 24.013330 -0.921985 15.344708

C 24.124210 -1.225142 17.769289

H 18.461123 -4.880646 10.701206

C 18.558677 -5.378974 12.781854

H 18.887371 -5.626967 14.910202

C 16.336391 -0.993580 14.569612

H 18.371748 -0.589400 14.115775

C 15.841669 -1.870595 16.757242

C 17.643434 -2.181990 18.407978

H 19.274399 -2.298294 19.797734

H 21.695976 -2.021729 20.049697

C 23.509642 -1.527962 19.014423

H 23.450836 -0.887297 14.423612

C 25.386139 -0.815331 15.296263

C 25.535956 -1.088800 17.684235

H 18.077251 -6.344640 12.660586

C 15.402249 -1.468611 15.517714

H 15.989688 -0.632752 13.605745

H 15.132938 -2.202378 17.511707

H 16.891251 -2.482586 19.132343

H 24.140999 -1.630038 19.892949

H 25.877876 -0.691928 14.335844

C 26.159735 -0.875186 16.477427

H 26.118973 -1.175996 18.597519

H 14.344077 -1.490983 15.275314

H 27.239990 -0.778209 16.426135

*T_1_*-**1a**

0 3

C 21.204330 0.461829 12.417108

C 21.187871 0.552172 13.831494

C 21.454858 1.582305 11.615768

H 21.463759 1.495066 10.533078

C 21.691146 2.802478 12.223748

H 21.887520 3.682727 11.617596

C 21.685386 2.945208 13.639936

C 21.429479 1.804928 14.481733

C 21.930362 4.206980 14.243019

H 22.122484 5.061365 13.598982

C 21.926422 4.353843 15.612116

H 22.115421 5.325074 16.059363

C 21.675002 3.234459 16.436923

H 21.672020 3.351563 17.516616

C 21.432830 1.992535 15.886878

H 21.242398 1.148370 16.536731

C 20.928315 -0.924765 12.049839

C 20.905032 -0.761707 14.356512

C 20.834130 -1.530708 10.791129

C 20.746775 -1.666599 13.243845

C 20.796865 -1.115405 15.772386

C 20.557363 -2.884309 10.717308

H 20.976026 -0.946995 9.886112

C 20.460135 -3.068618 13.190895

C 19.589357 -1.087604 16.561730

C 21.878988 -1.553413 16.620403

H 20.480472 -3.371263 9.748911

C 20.367069 -3.672929 11.886856

C 20.264284 -3.891498 14.328498

C 18.242326 -0.734335 16.228074

C 19.918780 -1.502047 17.876518

C 21.349743 -1.793051 17.913202

C 23.271807 -1.756670 16.357069

C 20.084113 -5.060716 11.788040

C 19.990951 -5.237059 14.193104

H 20.330589 -3.456701 15.317283

C 17.818484 -0.309292 14.943804

C 17.259690 -0.820735 17.277511

C 18.948904 -1.579501 18.883342

C 22.161814 -2.232335 18.965797

C 23.890408 -1.543586 15.099482

C 24.090080 -2.208451 17.452813

H 20.015846 -5.507225 10.799283

C 19.899028 -5.830969 12.914297

H 19.845017 -5.845627 15.080690

C 16.499083 0.013881 14.702297

H 18.541467 -0.237598 14.141727

C 15.911693 -0.479779 16.990401

C 17.641145 -1.242740 18.582270

H 19.218943 -1.899246 19.885715

H 21.739501 -2.411186 19.950467

C 23.510494 -2.435153 18.732925

H 23.292839 -1.204085 14.263529

C 25.241618 -1.762445 14.926548

C 25.476756 -2.422615 17.235910

H 19.683210 -6.891250 12.823368

C 15.534145 -0.070574 15.731030

H 16.200133 0.336833 13.709426

H 15.177702 -0.548291 17.789296

H 16.875194 -1.296953 19.351167

H 24.155804 -2.775878 19.538100

H 25.691301 -1.591637 13.952860

C 26.045447 -2.205627 16.000789

H 26.085725 -2.763587 18.069316

H 14.499415 0.187030 15.526052

H 27.107514 -2.373596 15.849659

**1a**-TS_rot_ (OS, S^2^=1.057139)

0 1

C 3.792934 3.793090 0.829812

C 3.451132 3.451641 2.119060

C 2.464126 2.464724 2.379241

C 1.814789 1.815102 1.270566

C 2.191263 2.191217 -0.043476

C 3.154937 3.154815 -0.257493

C 2.116865 2.117829 3.715325

C 1.158254 1.159311 3.990882

C 0.515618 0.516401 2.925852

C 0.825978 0.826393 1.578833

C -0.517409 -0.516608 2.925498

C -0.826947 -0.826502 1.578267

C -0.000225 -0.000024 0.731464

C -1.160695 -1.159596 3.990088

C -2.119137 -2.118095 3.713876

C -2.465581 -2.464893 2.377555

C -1.815568 -1.815189 1.269324

C -3.452427 -3.451792 2.116699

C -3.793442 -3.793146 0.827217

C -3.154783 -3.154789 -0.259652

C -2.191240 -2.191206 -0.044976

C 0.000225 0.000028 -0.731464

C -0.825963 0.826519 -1.578775

C -0.515609 0.516615 -2.925815

C 0.517399 -0.516412 -2.925534

C 0.826932 -0.826406 -1.578326

C 1.160673 -1.159338 -3.990170

C 2.119097 -2.117874 -3.714025

C 2.465536 -2.464771 -2.377728

C 1.815535 -1.815133 -1.269451

C -1.814756 1.815225 -1.270438

C -2.464081 2.464936 -2.379068

C -2.116827 2.118128 -3.715177

C -1.158234 1.159612 -3.990800

C -2.191222 2.191255 0.043631

C -3.154878 3.154855 0.257715

C -3.792864 3.793218 -0.829546

C -3.451069 3.451853 -2.118817

C 3.452364 -3.451707 -2.116941

C 3.793372 -3.793157 -0.827484

C 3.154725 -3.154865 0.259430

C 2.191201 -2.191249 0.044821

H 4.549665 4.549757 0.645844

H 3.935516 3.936242 2.962972

H 1.714887 1.714624 -0.890310

H 3.425461 3.425061 -1.273974

H 2.625921 2.627093 4.529165

H 0.907960 0.909298 5.017884

H -0.911029 -0.909658 5.017261

H -2.628689 -2.627420 4.527367

H -3.937326 -3.936455 2.960280

H -4.550060 -4.549800 0.642732

H -3.424686 -3.424961 -1.276317

H -1.714349 -1.714550 -0.891483

H 0.911011 -0.909324 -5.017325

H 2.628639 -2.627151 -4.527552

H -2.625874 2.627459 -4.528980

H -0.907945 0.909666 -5.017820

H -1.714855 1.714594 0.890431

H -3.425397 3.425036 1.274215

H -4.549581 4.549887 -0.645524

H -3.935444 3.936522 -2.962696

H 3.937253 -3.936320 -2.960556

H 4.549976 -4.549839 -0.643052

H 3.424625 -3.425112 1.276076

H 1.714318 -1.714642 0.891363

*S_0_*-**1b**

0 1

C 8.870643 21.235077 10.117529

C 9.554513 22.356627 9.626852

H 9.394437 22.670535 8.598511

C 7.890315 20.483110 9.251240

H 6.879600 20.898818 9.350295

H 7.835679 19.426780 9.531156

H 8.163076 20.541139 8.193240

C 9.103015 20.849391 11.445569

C 10.434819 23.073745 10.428767

C 9.979986 21.556330 12.259980

H 8.588294 19.979573 11.845706

C 10.663166 22.685216 11.764307

H 10.957964 23.937973 10.032467

H 10.149521 21.242360 13.284916

C 11.563981 23.411217 12.589101

C 12.336777 24.035235 13.294125

C 13.240955 24.768923 14.102047

C 13.480733 24.393322 15.473991

C 13.882907 25.886742 13.568831

C 12.782265 23.303660 16.053749

C 14.407900 25.143895 16.280959

H 13.655342 26.200812 12.555550

C 14.819595 26.577519 14.335845

H 12.101005 22.738332 15.426727

C 12.941018 22.979328 17.381846

C 14.501431 24.810011 17.657032

C 15.155520 26.194383 15.649681

C 15.593517 27.764039 13.985692

H 12.394263 22.144082 17.808979

C 13.792721 23.757981 18.193949

H 15.135079 25.400868 18.301350

C 16.157493 27.139258 16.155812

C 15.596548 28.485471 12.793053

C 16.407122 28.109053 15.083348

H 13.887156 23.533844 19.252286

C 16.765449 27.119158 17.431400

H 14.948989 28.200433 11.970506

C 16.429376 29.596424 12.658463

C 17.381571 29.151585 14.926810

C 16.848849 28.237271 18.377910

C 17.434669 25.976441 18.063344

C 16.381098 30.361027 11.466136

C 17.348600 29.933499 13.717800

C 18.395527 29.448570 15.874164

C 16.248854 29.541387 18.392822

C 17.535885 27.788281 19.523408

C 17.808498 24.679791 17.573525

C 17.897388 26.387932 19.329234

C 16.328701 31.011481 10.437625

C 18.252899 31.015680 13.567873

C 19.272516 30.494354 15.688370

H 18.489123 28.833855 16.756844

C 15.359298 30.024909 17.398514

C 16.545498 30.410707 19.502083

C 17.779092 28.609726 20.622928

C 18.473507 23.779322 18.479681

C 17.589829 24.232248 16.244741

C 18.588740 25.536115 20.189044

C 16.264421 31.769241 9.237415

H 18.202343 31.604933 12.658427

C 19.188466 31.303552 14.535388

H 20.037841 30.689800 16.433561

C 14.853494 31.305303 17.443976

H 15.063085 29.370469 16.592465

C 16.024424 31.729714 19.507126

C 17.334595 29.931918 20.610639

H 18.321683 28.236472 21.485155

C 18.789797 22.467076 18.044546

C 18.843692 24.220313 19.802270

H 17.157521 24.910195 15.524163

C 17.928259 22.957382 15.847920

H 18.921409 25.880824 21.162530

C 15.366141 31.418003 8.209562

C 17.100546 32.887342 9.043606

H 19.875392 32.133631 14.400776

H 14.172592 31.639595 16.666894

C 15.206107 32.177934 18.495353

H 16.272388 32.376648 20.341902

C 17.640119 30.778261 21.705601

C 18.513185 22.054428 16.761024

H 19.274753 21.796380 18.745869

C 19.492441 23.343182 20.706896

H 17.748818 22.651004 14.821680

H 14.719913 30.556532 8.343292

C 15.311386 32.162099 7.036695

C 17.033853 33.621537 7.865224

H 17.800881 33.166693 9.824185

H 14.813128 33.189881 18.518848

C 17.911232 31.496663 22.651152

H 18.766749 21.047083 16.444824

C 20.048414 22.598397 21.494207

H 14.614091 31.872972 6.254500

C 16.139177 33.277034 6.841831

H 17.689507 34.478549 7.733764

C 18.229244 32.332209 23.755492

C 20.697098 21.732058 22.414801

C 16.048843 34.100192 5.580400

C 17.790814 33.671105 23.798017

C 18.995253 31.843322 24.833046

C 20.992979 20.398734 22.066325

C 21.056046 22.184044 23.700764

H 15.734082 33.492762 4.726543

H 15.316698 34.909999 5.691572

H 17.009260 34.562574 5.333464

H 17.204342 34.062917 22.973188

C 18.109186 34.483015 24.880463

H 19.343757 30.815856 24.811075

C 19.305229 32.667556 25.908532

H 20.718139 20.035179 21.081387

C 21.624804 19.556581 22.973931

C 21.687659 21.329823 24.597181

H 20.829652 23.206775 23.984293

H 17.764258 35.513787 24.892781

C 18.867966 33.999112 25.955813

H 19.899819 32.272561 26.728351

H 21.841393 18.530714 22.687031

C 21.986626 20.003774 24.252786

H 21.953538 21.696609 25.585328

C 19.179266 34.876441 27.143385

C 22.704015 19.093685 25.219240

H 18.400722 34.788008 27.911643

H 19.236491 35.931382 26.858922

H 20.128891 34.597819 27.610069

H 22.432095 18.045787 25.060815

H 23.791810 19.169952 25.096543

H 22.475599 19.351277 26.257818

*T_1_*-**1b**

0 3

C 7.082906 22.528109 11.577463

C 7.820534 23.552337 10.966266

H 7.513404 23.920930 9.990846

C 5.850840 21.958975 10.918022

H 4.953589 22.516604 11.214987

H 5.691500 20.913804 11.199758

H 5.919221 22.010863 9.827260

C 7.507229 22.067640 12.832346

C 8.937695 24.104137 11.582074

C 8.622178 22.609086 13.461118

H 6.954113 21.270377 13.322277

C 9.359433 23.641134 12.845279

H 9.498721 24.894631 11.094241

H 8.937817 22.239026 14.431271

C 10.501536 24.197767 13.479136

C 11.482075 24.678268 14.021371

C 12.620974 25.245242 14.638647

C 13.068835 24.781970 15.932725

C 13.315943 26.268734 13.978161

C 12.378687 23.748662 16.613470

C 14.228251 25.368904 16.548073

H 12.967208 26.605367 13.007035

C 14.435440 26.837071 14.570235

H 11.505127 23.312833 16.140140

C 12.800118 23.302023 17.847279

C 14.632938 24.884538 17.817329

C 14.909910 26.413211 15.840023

C 15.317804 27.900535 14.094650

H 12.257709 22.508937 18.353151

C 13.937365 23.876571 18.452136

H 15.503265 25.314524 18.294363

C 16.072408 27.195269 16.162953

C 15.281955 28.636590 12.918324

C 16.317418 28.110506 15.081481

H 14.269938 23.524846 19.424249

C 16.871800 27.079352 17.386739

H 14.510581 28.462472 12.174903

C 16.252927 29.620212 12.680772

C 17.328259 29.107079 14.877357

C 16.651967 27.806640 18.607365

C 18.009351 26.219385 17.570359

C 16.201552 30.360494 11.477059

C 17.288958 29.868100 13.658051

C 18.361061 29.382828 15.808338

C 15.671205 28.784729 18.978917

C 17.642676 27.403907 19.541970

C 18.660804 25.288918 16.694877

C 18.493352 26.409065 18.892065

C 16.146903 30.992059 10.435668

C 18.277333 30.858242 13.434068

C 19.307586 30.354548 15.558043

H 18.405521 28.819897 16.730857

C 14.648631 29.243222 18.110805

C 15.731326 29.329232 20.308312

C 17.698653 27.931123 20.824826

C 19.799837 24.571368 17.199941

C 18.245266 25.037987 15.363026

C 19.593220 25.715060 19.377360

C 16.080367 31.726557 9.222365

H 18.238311 31.426590 12.510733

C 19.267738 31.100058 14.361426

H 20.087725 30.544770 16.289151

C 13.731279 30.187096 18.523573

H 14.588575 28.844289 17.107217

C 14.772857 30.297059 20.698292

C 16.757715 28.890179 21.226807

H 18.463050 27.611759 21.526056

C 20.459923 23.646721 16.353320

C 20.257965 24.796093 18.552529

H 17.390242 25.568962 14.966886

C 18.911441 24.130398 14.565967

H 19.949391 25.872626 20.390445

C 15.065528 31.469777 8.277870

C 17.030004 32.726997 8.930385

H 20.016346 31.863330 14.171428

H 12.958299 30.520205 17.837380

C 13.791630 30.720216 19.827949

H 14.827438 30.701295 21.703629

C 16.829750 29.417066 22.537068

C 20.028643 23.427409 15.062874

H 21.318313 23.110771 16.744483

C 21.376454 24.097502 19.062952

H 18.571178 23.957924 13.549211

H 14.330869 30.699048 8.487313

C 15.008778 32.190692 7.090923

C 16.959664 33.439213 7.739001

H 17.819918 32.932278 9.645690

H 13.065985 31.462915 20.145652

C 16.902269 29.864270 23.668927

H 20.547947 22.714544 14.429436

C 22.339334 23.500853 19.513755

H 14.220417 31.975491 6.374119

C 15.949588 33.188813 6.798843

H 17.703262 34.204373 7.531153

C 16.988609 30.383032 24.987811

C 23.460895 22.807659 20.040638

C 15.859972 33.990984 5.523998

C 16.069848 31.349645 25.445536

C 17.998420 29.947549 25.870141

C 24.164593 21.870621 19.256553

C 23.895123 23.036049 21.362418

H 15.405189 33.410477 4.715728

H 15.244238 34.887967 5.667134

H 16.846910 34.324734 5.189751

H 15.290174 31.698116 24.775879

C 16.163248 31.854833 26.736903

H 18.715424 29.207656 25.529445

C 18.078522 30.462483 27.158644

H 23.838842 21.680677 18.238921

C 25.259401 21.193778 19.780765

C 24.991894 22.351098 21.872122

H 23.359798 23.750883 21.978899

H 15.447168 32.601879 27.069709

C 17.164305 31.421424 27.618326

H 18.866427 30.116185 27.822661

H 25.786569 20.472836 19.161130

C 25.696150 21.420972 21.093980

H 25.309050 22.538972 22.894737

C 17.236851 31.949299 29.029999

C 26.906985 20.707520 21.643176

H 16.632524 31.335705 29.710062

H 16.858911 32.974042 29.095070

H 18.262969 31.940243 29.409609

H 27.028138 19.717857 21.192531

H 27.823800 21.273865 21.436057

H 26.840953 20.581754 22.728031

**1b**-TS_rot_ (OS, S^2^=1.073687)

0 1

C 3.801396 3.783505 0.751813

C 3.485283 3.449863 2.051082

C 2.505440 2.467541 2.338226

C 1.834460 1.812716 1.248105

C 2.185002 2.181056 -0.075133

C 3.144113 3.142065 -0.318650

C 2.179026 2.122645 3.703602

C 1.206574 1.148004 3.970189

C 0.557430 0.514471 2.919366

C 0.846741 0.822140 1.563553

C -0.476638 -0.518273 2.933579

C -0.803277 -0.825738 1.586225

C 0.010101 -0.001743 0.732944

C -1.096400 -1.152078 4.001840

C -2.075730 -2.126793 3.762059

C -2.439783 -2.471431 2.406164

C -1.799265 -1.816348 1.298022

C -3.427121 -3.453777 2.146050

C -3.779029 -3.787214 0.855964

C -3.151647 -3.145538 -0.232151

C -2.186244 -2.184493 -0.015078

C -0.010086 -0.001784 -0.732934

C -0.845632 0.823147 -1.563605

C -0.556730 0.514992 -2.919395

C 0.475962 -0.519128 -2.933531

C 0.802197 -0.826923 -1.586154

C 1.094875 -1.153843 -4.001744

C 2.072913 -2.129837 -3.761890

C 2.436522 -2.474842 -2.405969

C 1.796875 -1.818826 -1.297876

C -1.832031 1.815061 -1.248231

C -2.502138 2.470697 -2.338401

C -2.176177 2.125272 -3.703752

C -1.205023 1.149316 -3.970265

C -2.182079 2.183969 0.074979

C -3.139912 3.146270 0.318425

C -3.796344 3.788500 -0.752086

C -3.480675 3.454341 -2.051331

C 3.422566 -3.458468 -2.145783

C 3.774040 -3.792266 -0.855672

C 3.147507 -3.149680 0.232395

C 2.183371 -2.187380 0.015251

H 4.555330 4.539045 0.552205

H 3.985461 3.937768 2.881061

H 1.690032 1.698365 -0.906973

H 3.392903 3.404657 -1.342597

H 0.972724 0.900175 5.000677

H -0.834120 -0.904447 5.025510

H -3.904123 -3.941862 2.989456

H -4.538138 -4.542777 0.677147

H -3.428632 -3.407971 -1.248873

H -1.714526 -1.701645 -0.860223

H 0.832916 -0.905950 -5.025433

H -0.971499 0.901104 -5.000735

H -1.687746 1.700687 0.906855

H -3.388349 3.409272 1.342352

H -4.549274 4.545057 -0.552535

H -3.980205 3.942848 -2.881346

H 3.898921 -3.947247 -2.989152

H 4.532155 -4.548814 -0.676798

H 3.424149 -3.412399 1.249137

H 1.712293 -1.703845 0.860360

C -2.826824 2.761472 -4.786167

C -3.380956 3.302858 -5.727523

C 2.693245 -2.766578 -4.861646

C 3.220921 -3.308744 -5.817641

C 2.830533 2.758045 4.785971

C 3.385400 3.298745 5.727288

C -2.696921 -2.762615 4.861862

C -3.225335 -3.303993 5.817896

C -4.023996 3.932095 -6.825999

C -3.695887 3.581720 -8.151699

C -5.009389 4.918813 -6.617959

C -4.331940 4.199675 -9.221896

H -2.942811 2.820187 -8.326499

C -5.636347 5.527525 -7.698723

H -5.276019 5.195264 -5.602947

C -5.310019 5.183939 -9.018666

H -4.067137 3.913192 -10.236512

H -6.395825 6.283735 -7.517211

C 3.834331 -3.938041 -6.932899

C 3.467442 -3.591307 -8.249349

C 4.821512 -4.928158 -6.751091

C 4.070006 -4.214078 -9.336021

H 2.705367 -2.834383 -8.404012

C 5.414692 -5.541679 -7.848068

H 5.110574 -5.209057 -5.743467

C 5.054646 -5.196830 -9.158891

H 3.770934 -3.934972 -10.343142

H 6.171515 -6.305088 -7.686716

C 4.029292 3.927180 6.825724

C 3.700816 3.577225 8.151445

C 5.015912 4.912657 6.617625

C 4.337704 4.194385 9.221603

H 2.946794 2.816639 8.326290

C 5.643693 5.520589 7.698351

H 5.282832 5.188769 5.602596

C 5.317007 5.177420 9.018314

H 4.072598 3.908240 10.236235

H 6.404110 6.275844 7.516793

C -3.839605 -3.932377 6.933195

C -3.472389 -3.585912 8.249625

C -4.827992 -4.921302 6.751450

C -4.075803 -4.207790 9.336338

H -2.709395 -2.829905 8.404241

C -5.422009 -5.533941 7.848466

H -5.117321 -5.201985 5.743842

C -5.061638 -5.189354 9.159269

H -3.776466 -3.928912 10.343443

H -6.179758 -6.296441 7.687162

C -5.970288 5.874879 -10.186307

H -5.407799 6.769419 -10.482288

H -6.023900 5.220643 -11.061657

H -6.986325 6.197209 -9.939519

C 5.728833 -5.842742 -10.344221

H 5.063634 -5.881889 -11.212039

H 6.621563 -5.279031 -10.643326

H 6.050745 -6.863199 -10.115784

C -5.736714 -5.834272 10.344635

H -5.071664 -5.874041 11.212537

H -6.628824 -5.269471 10.643534

H -6.059787 -6.854394 10.116338

C 5.978207 5.867532 10.185917

H 5.416812 6.762732 10.481980

H 6.031116 5.213202 11.061241

H 6.994606 6.188656 9.939049

*S_0_*-**1c**

0 1

C 11.275801 7.469673 8.760920

C 11.460103 7.173411 10.191862

C 12.466309 6.100540 10.268200

C 13.192518 5.524369 11.365602

C 13.051263 5.918907 12.721205

H 12.385973 6.733886 12.964403

H 13.615194 5.627332 14.757544

C 12.860841 5.779440 8.960036

C 12.118913 6.619747 8.028117

C 10.316853 8.267206 8.048109

C 9.266868 9.001352 8.657851

H 9.135749 8.940073 9.727897

C 8.405595 9.781767 7.917320

H 7.612871 10.327260 8.421150

C 8.539466 9.870593 6.516145

H 7.863890 10.497391 5.941769

C 9.512971 9.134825 5.877617

H 9.606169 9.162266 4.798531

C 10.400613 8.308387 6.612144

C 11.354537 7.482861 5.925950

C 12.171919 6.614028 6.624569

H 12.847136 5.938905 6.113930

C 12.261327 6.815043 3.816892

H 12.104791 7.088129 2.772391

H 12.064509 5.742413 3.943586

H 13.302434 7.024147 4.095321

C 10.121088 7.118873 12.388189

C 9.603593 8.107424 13.239674

C 9.949166 9.416952 12.700465

C 9.664519 10.680704 13.243328

H 9.099811 10.756505 14.164115

C 10.114785 11.805649 12.577891

C 10.938292 11.688147 11.407269

C 11.460296 12.848957 10.782388

H 11.183801 13.818259 11.179718

C 12.316552 12.749876 9.708236

H 12.711802 13.646599 9.240419

C 12.700848 11.476745 9.238592

H 13.408388 11.392911 8.418729

C 12.193759 10.332202 9.814896

H 12.513203 9.369208 9.445434

C 11.269018 10.386854 10.889634

C 10.668573 9.237890 11.508575

C 10.805579 7.790845 11.270506

C 8.860728 7.813904 14.395020

H 8.491796 8.619653 15.017238

C 8.621810 6.489655 14.711808

C 9.039688 5.435115 13.831088

C 8.716252 4.086909 14.128039

H 8.193197 3.872622 15.052356

C 9.038899 3.072859 13.253746

C 9.676445 3.380957 12.034019

H 9.895148 2.589974 11.322446

C 10.019580 4.680258 11.728580

H 10.495512 4.891928 10.782731

C 9.753758 5.749944 12.622088

C 9.059506 13.283278 14.133325

H 8.060761 12.843757 14.013003

H 8.966915 14.363761 14.251844

O 11.352779 7.604196 4.568377

O 9.859116 13.083075 12.978562

C 14.154875 4.494116 11.077259

C 13.749307 5.294674 13.732277

C 13.836495 4.812248 8.667649

O 7.958708 6.079842 15.829886

H 8.782882 2.044708 13.491543

H 9.532046 12.860388 15.029537

C 14.443030 4.143766 9.714700

C 14.844700 3.856571 12.139396

C 14.638711 4.238029 13.446627

H 14.095623 4.602980 7.637332

C 7.504680 7.063131 16.746351

O 15.364893 3.152499 9.555689

H 15.552140 3.072041 11.898755

H 15.175635 3.743821 14.250778

H 7.031080 6.517281 17.563549

H 6.768507 7.734750 16.285594

H 8.336787 7.660044 17.141879

C 15.711454 2.755069 8.238542

H 14.837216 2.388093 7.685203

H 16.172052 3.578241 7.676791

H 16.435039 1.946143 8.348665

*T_1_*-**1c**

0 3

C 11.029604 7.149238 8.801200

C 11.476257 7.160143 10.170266

C 12.712873 6.425545 10.241771

C 13.588345 6.119641 11.334639

C 13.360014 6.533232 12.670922

H 12.476773 7.115733 12.898028

H 14.041411 6.536704 14.695225

C 13.026573 5.967423 8.940022

C 11.972745 6.420432 8.038172

C 9.879104 7.714576 8.159970

C 8.881564 8.459286 8.837639

H 8.977896 8.620810 9.903300

C 7.795817 8.978341 8.162354

H 7.046842 9.545118 8.707968

C 7.651670 8.779225 6.773802

H 6.793651 9.191669 6.251383

C 8.602274 8.059829 6.080338

H 8.502555 7.900148 5.013227

C 9.727745 7.514748 6.745762

C 10.719018 6.764386 6.020651

C 11.826764 6.224545 6.658926

H 12.571746 5.659983 6.111479

C 11.399867 5.912525 3.890440

H 11.007620 5.940791 2.872911

H 11.482228 4.868115 4.218284

H 12.395568 6.374402 3.908048

C 9.802582 7.194368 12.143768

C 9.395724 8.153747 13.101322

C 10.139574 9.383506 12.849341

C 10.094391 10.612616 13.519618

H 9.428588 10.743485 14.364027

C 10.912854 11.643819 13.080670

C 11.798306 11.472472 11.959030

C 12.627874 12.534474 11.522509

H 12.582428 13.479475 12.050785

C 13.476707 12.371791 10.447758

H 14.107386 13.194180 10.123493

C 13.524168 11.135795 9.770564

H 14.192778 11.008369 8.924030

C 12.727797 10.083111 10.172966

H 12.775501 9.140923 9.642835

C 11.843123 10.211525 11.272854

C 10.990679 9.158733 11.741215

C 10.791988 7.804457 11.293567

C 8.435758 7.870739 14.081326

H 8.152195 8.633597 14.796225

C 7.865231 6.606042 14.112618

C 8.240231 5.590757 13.163909

C 7.648843 4.304338 13.208284

H 6.907562 4.099869 13.971735

C 8.007228 3.330457 12.299881

C 8.973565 3.612124 11.312284

H 9.255414 2.844669 10.597114

C 9.566836 4.856180 11.244354

H 10.306648 5.056844 10.480550

C 9.224902 5.882700 12.159915

C 10.111934 13.139234 14.768641

H 9.051217 13.031471 14.507242

H 10.308415 14.172491 15.058115

O 10.475133 6.637134 4.686233

O 10.952117 12.878575 13.655259

C 14.767856 5.347747 11.059432

C 14.242261 6.205938 13.680264

C 14.176453 5.214589 8.669731

O 6.924084 6.220302 15.019134

H 7.546019 2.348382 12.345102

H 10.343376 12.476154 15.612338

C 15.036099 4.908532 9.715334

C 15.654700 5.027443 12.116549

C 15.398927 5.447736 13.404968

H 14.379994 4.883002 7.658757

C 6.492770 7.156733 15.994106

O 16.180924 4.184391 9.569401

H 16.540131 4.444306 11.892639

H 16.087130 5.195203 14.206113

H 5.750061 6.637689 16.601558

H 6.030193 8.037581 15.530223

H 7.322263 7.482077 16.635317

C 16.526953 3.709411 8.277836

H 15.762731 3.028914 7.880407

H 16.675118 4.535881 7.570696

H 17.465196 3.166175 8.398738

**1c**-TS_rot_ (OS, S^2^=1.048891)

0 1

C 3.942655 3.629208 0.776929

C 3.601814 3.309681 2.074494

C 2.583724 2.362456 2.344866

C 1.899143 1.728363 1.252933

C 2.277689 2.082774 -0.066225

C 3.273616 3.009172 -0.298535

C 2.229228 2.029719 3.699424

C 1.234165 1.103769 3.979483

C 0.567473 0.486565 2.913333

C 0.874559 0.775311 1.562397

C -0.501109 -0.506931 2.928906

C -0.832555 -0.811653 1.587270

C 0.013310 -0.023171 0.728053

C -1.148215 -1.111500 4.014201

C -2.147948 -2.040745 3.763191

C -2.526785 -2.389567 2.419312

C -1.862262 -1.768421 1.307723

C -3.549274 -3.340065 2.178678

C -3.913390 -3.675073 0.891382

C -3.264073 -3.067833 -0.203297

C -2.264428 -2.138608 -0.000067

C 0.000071 -0.031962 -0.736292

C -0.801119 0.816505 -1.581058

C -0.516615 0.489005 -2.928096

C 0.476917 -0.579655 -2.930618

C 0.785898 -0.890735 -1.585086

C 1.078044 -1.243164 -4.007888

C 2.008104 -2.238998 -3.744701

C 2.361188 -2.597439 -2.396348

C 1.743544 -1.916138 -1.292995

C -1.753376 1.845530 -1.284139

C -2.391051 2.513395 -2.384306

C -2.062547 2.138454 -3.734385

C -1.137319 1.139375 -4.002268

C -2.103698 2.243957 0.030247

C -3.029551 3.243153 0.250373

C -3.653107 3.895735 -0.833137

C -3.337615 3.535323 -2.126393

C 3.312452 -3.616204 -2.143258

C 3.651517 -3.960802 -0.851659

C 3.047706 -3.294978 0.234973

C 2.117834 -2.298490 0.019561

H 4.725026 4.357063 0.583599

H 4.108866 3.779477 2.908942

H 1.773599 1.615549 -0.902062

H 3.542840 3.261365 -1.320210

H 0.968764 0.854469 4.999715

H -0.864414 -0.850157 5.026437

H -4.041003 -3.799941 3.027692

H -4.698882 -4.405274 0.720898

H -3.551588 -3.332243 -1.216868

H -1.775608 -1.681416 -0.850391

H 0.813472 -0.974752 -5.023475

H -0.891228 0.858552 -5.019146

H -1.633757 1.752630 0.872139

H -3.278573 3.527780 1.268645

H -4.380500 4.680839 -0.649380

H -3.810143 4.029602 -2.966938

H 3.769662 -4.120747 -2.986173

H 4.382264 -4.743645 -0.671603

H 3.315299 -3.567137 1.251942

H 1.663284 -1.796873 0.863827

O -2.843009 -2.689793 4.739006

O 2.941847 2.690107 4.654745

O -2.726095 2.836431 -4.698289

O 2.654067 -2.948781 -4.711927

C -2.537094 -2.408658 6.095809

H -3.206183 -3.032322 6.690248

H -1.496385 -2.662699 6.335455

H -2.714405 -1.352903 6.338675

C 2.660565 2.425113 6.020098

H 3.340195 3.055754 6.594893

H 1.624318 2.682027 6.275509

H 2.842413 1.372307 6.272236

C 2.368502 -2.663575 -6.072310

H 2.621629 -1.626615 -6.328601

H 2.990323 -3.341587 -6.658512

H 1.311989 -2.844647 -6.309025

C -2.465475 2.534449 -6.060059

H -1.413504 2.712492 -6.318324

H -2.723118 1.494431 -6.298861

H -3.098028 3.205240 -6.643077

*S_0_*-*anti*-**1d**

0 1

C 13.134754 24.808046 14.149837

C 13.372251 24.414853 15.511156

C 13.767168 25.913521 13.609426

C 12.658391 23.327180 16.074236

C 14.308520 25.150329 16.318379

H 13.548385 26.258055 12.606676

C 14.717167 26.591612 14.388000

H 11.970424 22.778060 15.442616

C 12.817888 22.986531 17.399375

C 14.402281 24.796679 17.688410

C 15.059360 26.204931 15.693443

C 15.483859 27.781800 14.037503

H 12.263631 22.152614 17.819330

C 13.681850 23.745744 18.214856

H 15.047010 25.370090 18.337536

C 16.054200 27.156354 16.207089

C 15.483232 28.502808 12.843648

C 16.296719 28.132230 15.134002

H 13.779496 23.507570 19.269993

C 16.660813 27.138373 17.478875

H 14.835664 28.215449 12.021631

C 16.312652 29.614780 12.704629

C 17.270640 29.173007 14.971222

C 16.755663 28.259637 18.423675

C 17.325634 25.992456 18.117294

C 16.260932 30.377500 11.510429

C 17.233901 29.954052 13.761163

C 18.289548 29.470294 15.914350

C 16.158911 29.567267 18.444670

C 17.432878 27.811118 19.568624

C 17.705084 24.696881 17.631667

C 17.790003 26.409569 19.380765

C 16.206850 31.026635 10.481268

C 18.137863 31.036151 13.607370

C 19.165707 30.515340 15.724179

H 18.384805 28.855827 16.797060

C 15.278298 30.067459 17.452132

C 16.446723 30.425645 19.562466

C 17.674132 28.623501 20.686795

C 18.370090 23.798846 18.541039

C 17.491875 24.246514 16.302109

C 18.481229 25.559676 20.243885

C 16.142167 31.784226 9.280630

H 18.083870 31.624958 12.697790

C 19.077560 31.323954 14.570642

H 19.934430 30.711144 16.465904

C 14.772536 31.348506 17.513357

H 14.989384 29.426237 16.632874

C 15.927992 31.744670 19.590104

C 17.225527 29.932051 20.664282

H 18.206032 28.221060 21.539552

C 18.689923 22.486401 18.108670

C 18.737881 24.243371 19.862827

H 17.059379 24.923243 15.580457

C 17.834134 22.972159 15.908633

H 18.814395 25.907046 21.216491

C 15.244587 31.433099 8.252237

C 16.977696 32.902737 9.086518

H 19.764483 32.153553 14.432494

H 14.098817 31.693830 16.734620

C 15.115648 32.206664 18.578111

H 16.171407 32.378190 20.434603

C 18.418567 22.071544 16.824927

H 19.174022 21.817640 18.812489

C 19.387323 23.369376 20.771050

H 17.658759 22.663577 14.882256

H 14.598549 30.571376 8.385607

C 15.189783 32.177091 7.079141

C 16.911082 33.636907 7.908027

H 17.677716 33.182504 9.867238

H 14.722762 33.218330 18.612073

H 18.675591 21.064304 16.510939

C 19.943589 22.626946 21.560253

H 14.492752 31.887557 6.296782

C 16.017012 33.292284 6.884224

H 17.566528 34.494184 7.776910

C 20.592676 21.761824 22.482152

C 15.926889 34.115558 5.622747

C 20.889122 20.428142 22.135454

C 20.951715 22.214535 23.767741

H 15.608076 33.509142 4.769641

H 15.198248 34.928391 5.735015

H 16.888579 34.574134 5.373496

H 20.614336 20.063435 21.150921

C 21.521190 19.586967 23.043901

C 21.583639 21.361375 24.665175

H 20.725056 23.237457 24.050538

H 21.737915 18.560854 22.757823

C 21.883031 20.035151 24.322353

H 21.849383 21.729310 25.652993

C 22.600680 19.125881 25.289523

H 22.323420 18.078523 25.136548

H 23.688336 19.196492 25.162123

H 22.377686 19.388485 26.328041

O 12.235142 24.040870 13.476266

O 17.443017 30.828521 21.664608

C 11.937494 24.369066 12.126838

H 12.834817 24.329133 11.496267

H 11.222121 23.619311 11.786524

H 11.484436 25.365398 12.045765

C 18.198752 30.416247 22.794267

H 19.208929 30.097572 22.507268

H 18.265537 31.288863 23.445259

H 17.702902 29.598974 23.333459

*S_0_*-*syn*-**1d**

0 1

C 13.156876 24.824445 14.153106

C 13.423411 24.414146 15.503960

C 13.787224 25.929473 13.609229

C 12.710681 23.328985 16.073267

C 14.386824 25.131068 16.295643

H 13.547523 26.287130 12.615908

C 14.761285 26.590975 14.371877

H 12.001120 22.794460 15.453119

C 12.897896 22.973179 17.390789

C 14.512319 24.760367 17.658558

C 15.131541 26.186916 15.665048

C 15.526303 27.780828 14.016760

H 12.343708 22.141824 17.815903

C 13.792062 23.712566 18.191680

H 15.182471 25.317297 18.296128

C 16.140518 27.128549 16.166911

C 15.499892 28.518441 12.833208

C 16.366372 28.113339 15.097525

H 13.914150 23.460831 19.241155

C 16.777312 27.102632 17.423642

H 14.831494 28.245320 12.023083

C 16.328838 29.630300 12.690846

C 17.341728 29.150816 14.925683

C 16.889130 28.227281 18.365516

C 17.452462 25.959734 18.051584

C 16.249616 30.411819 11.510320

C 17.277035 29.950998 13.729262

C 18.387065 29.428403 15.845762

C 16.288664 29.529485 18.395687

C 17.603926 27.779231 19.493647

C 17.820204 24.658499 17.563224

C 17.953710 26.377261 19.295414

C 16.172054 31.077224 10.493137

C 18.178833 31.034212 13.571099

C 19.259561 30.475739 15.651979

H 18.506877 28.795905 16.712672

C 15.364438 30.008440 17.429906

C 16.622034 30.404731 19.490521

C 17.880998 28.605833 20.582488

C 18.526662 23.775268 18.451804

C 17.561165 24.188677 16.250655

C 18.686834 25.539496 20.149056

C 16.079860 31.853895 9.306545

H 18.103462 31.638132 12.673020

C 19.142146 31.305081 14.515817

H 20.048503 30.656409 16.376029

C 14.865283 31.290543 17.482608

H 15.037080 29.347258 16.641473

C 16.105609 31.725618 19.503039

C 17.442624 29.929258 20.577188

H 18.446209 28.235447 21.431608

C 18.856058 22.463517 18.026967

C 18.931670 24.237368 19.750514

H 17.086303 24.847986 15.539571

C 17.910164 22.914128 15.857474

H 19.043939 25.918503 21.098224

C 15.157298 31.520451 8.294543

C 16.912517 32.974175 9.110159

H 19.826493 32.136312 14.374784

H 14.158169 31.621317 16.727691

C 15.256722 32.169792 18.515148

H 16.382319 32.377340 20.325004

C 17.783894 30.780785 21.658448

C 18.544062 22.032254 16.756454

H 19.374895 21.811074 18.718996

H 17.697245 22.592024 14.842296

H 14.513085 30.657633 8.429680

C 15.075776 32.283032 7.134975

C 16.819017 33.727043 7.945392

H 17.631558 33.240500 9.878191

H 14.868052 33.183323 18.544470

C 18.084272 31.504785 22.590671

H 18.804457 21.026099 16.442060

H 14.359692 32.006915 6.365069

C 15.900088 33.399972 6.937946

H 17.472695 34.585356 7.812189

C 18.435129 32.349279 23.678538

C 15.781484 34.243366 5.692205

C 17.999878 33.689311 23.722147

C 19.230867 31.869555 24.738259

H 15.436338 33.652239 4.838629

H 15.061247 35.058641 5.836457

H 16.739056 34.700190 5.424484

H 17.390610 34.074713 22.910924

C 18.349915 34.510336 24.787761

H 19.577639 30.841470 24.715725

C 19.572444 32.702880 25.797398

H 18.006646 35.541720 24.800540

C 19.138477 34.035214 25.845539

H 20.189696 32.314137 26.603400

C 19.483903 34.923657 27.015408

H 18.711306 34.868214 27.792733

H 19.564458 35.972099 26.712668

H 20.431166 34.629342 27.477105

O 12.232986 24.074166 13.493824

O 19.587187 23.319237 20.511064

C 20.030027 23.700335 21.805764

H 19.191533 24.011514 22.441701

H 20.500514 22.814641 22.234539

H 20.765501 24.513400 21.756095

C 11.904291 24.421203 12.156268

H 12.784654 24.379452 11.502399

H 11.172593 23.682809 11.826037

H 11.459848 25.422999 12.098428

*T_1_*-**1d**

0 3

C 12.544474 25.279788 14.692861

C 12.986127 24.799943 15.976533

C 13.220796 26.294787 14.027888

C 12.285672 23.763344 16.640372

C 14.148483 25.375979 16.591767

H 12.888585 26.655290 13.062178

C 14.352836 26.854328 14.629037

H 11.410479 23.339126 16.162824

C 12.707326 23.301101 17.869687

C 14.550436 24.874868 17.854169

C 14.832836 26.423806 15.889452

C 15.233497 27.916962 14.152236

H 12.161642 22.505529 18.368010

C 13.848117 23.862994 18.477604

H 15.423912 25.294792 18.334911

C 16.000252 27.199385 16.211997

C 15.192257 28.660532 12.979191

C 16.241203 28.120181 15.132067

H 14.180428 23.498244 19.445170

C 16.799706 27.075958 17.434071

H 14.415221 28.493537 12.239634

C 16.163348 29.641720 12.739010

C 17.252620 29.112889 14.925041

C 16.579912 27.797115 18.658789

C 17.933170 26.208272 17.620021

C 16.105737 30.389280 11.538794

C 17.207431 29.880815 13.709636

C 18.294319 29.378709 15.850332

C 15.604874 28.780342 19.035716

C 17.559788 27.385248 19.594010

C 18.585101 25.280424 16.745130

C 18.408878 26.389773 18.945768

C 16.047240 31.027470 10.501903

C 18.198883 30.867845 13.484350

C 19.243048 30.346903 15.598117

H 18.341821 28.809369 16.769078

C 14.587452 29.257909 18.173625

C 15.665140 29.312941 20.367670

C 17.620298 27.902523 20.891976

C 19.717414 24.553402 17.253288

C 18.176728 25.040657 15.408068

C 19.502914 25.685854 19.433917

C 15.977572 31.770758 9.293647

H 18.155831 31.441604 12.564486

C 19.197181 31.099626 14.405518

H 20.030374 30.529331 16.323610

C 13.679129 30.207052 18.597757

H 14.524284 28.869808 17.165732

C 14.719321 30.285439 20.774538

C 16.684623 28.855014 21.275467

H 18.387144 27.558363 21.574911

C 20.377557 23.630636 16.404352

C 20.167784 24.768008 18.609939

H 17.326948 25.579948 15.011176

C 18.843180 24.135307 14.609761

H 19.854445 25.835284 20.450150

C 14.956246 31.526912 8.352879

C 16.930976 32.767807 9.002514

H 19.948046 31.860405 14.213989

H 12.910181 30.555101 17.914207

C 13.742267 30.726530 19.906510

H 14.778260 30.677449 21.782921

C 19.953522 23.422555 15.109846

H 21.230596 23.087368 16.797258

C 21.279824 24.059528 19.123841

H 18.509702 23.971692 13.589210

H 14.218239 30.758948 8.560964

C 14.896730 32.256341 7.171082

C 16.857986 33.488727 7.816380

H 17.726276 32.963645 9.714509

H 13.023275 31.472830 20.230726

H 20.473300 22.711214 14.474950

C 22.236432 23.453869 19.575407

H 14.102942 32.050496 6.457414

C 15.841359 33.250990 6.880165

H 17.604987 34.250967 7.609724

C 23.351438 22.748977 20.101799

C 15.749188 34.062578 5.611333

C 24.051680 21.812587 19.313931

C 23.783106 22.964196 21.426521

H 15.285135 33.490834 4.802023

H 15.141054 34.963197 5.764030

H 16.736387 34.391307 5.272775

H 23.728323 21.632189 18.293788

C 25.139975 21.124180 19.837026

C 24.873349 22.267873 21.935250

H 23.250790 23.678284 22.046552

H 25.664118 20.404080 19.213763

C 25.573971 21.338484 21.153188

H 25.188124 22.446249 22.960379

C 26.777978 20.612588 21.701420

H 26.894528 19.625868 21.243095

H 27.699635 21.174279 21.503169

H 26.706485 20.477942 22.784920

O 16.648388 29.425538 22.509170

O 11.434558 24.664574 14.204576

C 17.616122 29.034889 23.473276

H 18.634773 29.263996 23.135691

H 17.394510 29.613203 24.371027

H 17.544368 27.964426 23.703601

C 10.925734 25.070866 12.941777

H 11.659503 24.909192 12.142076

H 10.048679 24.449031 12.758433

H 10.627247 26.126774 12.949761

**1d**-TS_rot_ (OS, S^2^=1.058827)

0 1

C 3.897600 3.778541 0.791647

C 3.584004 3.425579 2.087667

C 2.602856 2.439257 2.353148

C 1.927747 1.799676 1.259246

C 2.277060 2.188808 -0.057218

C 3.237385 3.153823 -0.285701

C 2.276582 2.073440 3.706878

C 1.316059 1.109022 3.984336

C 0.659681 0.487531 2.917059

C 0.939393 0.805746 1.566489

C -0.375084 -0.542853 2.930784

C -0.712951 -0.839138 1.583734

C 0.097106 -0.008522 0.731100

C -0.989095 -1.186518 3.998204

C -1.971021 -2.156917 3.758101

C -2.346046 -2.488315 2.402063

C -1.712082 -1.823815 1.295093

C -3.337692 -3.465766 2.138938

C -3.700046 -3.785012 0.848337

C -3.079381 -3.133638 -0.238636

C -2.110155 -2.177774 -0.019522

C 0.076453 0.002304 -0.733970

C -0.753043 0.837088 -1.563524

C -0.454795 0.539103 -2.919527

C 0.574633 -0.496598 -2.934863

C 0.890639 -0.816279 -1.592671

C 1.197456 -1.121476 -4.020133

C 2.160438 -2.090828 -3.769703

C 2.522836 -2.458330 -2.425654

C 1.882103 -1.815311 -1.313251

C -1.738598 1.826846 -1.246946

C -2.400122 2.494598 -2.335678

C -2.065167 2.161320 -3.701707

C -1.095355 1.185931 -3.969281

C -2.097597 2.182822 0.078329

C -3.055355 3.143632 0.324572

C -3.703107 3.798178 -0.744545

C -3.378949 3.477097 -2.044815

C 3.505989 -3.449689 -2.187801

C 3.854132 -3.804315 -0.901090

C 3.227720 -3.176264 0.194351

C 2.266323 -2.206308 -0.007102

H 4.651471 4.536574 0.601648

H 4.084401 3.898910 2.924052

H 1.780143 1.719314 -0.895724

H 3.483992 3.432521 -1.306079

H 1.070588 0.834273 5.002755

H -0.719532 -0.949371 5.022759

H -3.809819 -3.961230 2.980846

H -4.462638 -4.536733 0.667684

H -3.365793 -3.384923 -1.255653

H -1.642665 -1.686471 -0.862484

H 0.924810 -0.845464 -5.031275

H -0.856003 0.947451 -5.001005

H -1.609011 1.689117 0.907821

H -3.311663 3.396379 1.349230

H -4.456445 4.553788 -0.542550

H -3.872107 3.974975 -2.873135

H 3.980191 -3.925544 -3.037900

H 4.609148 -4.566220 -0.732310

H 3.501507 -3.456276 1.207412

H 1.795648 -1.734324 0.845031

C -2.707050 2.810159 -4.783238

C -3.254025 3.363405 -5.721626

C -2.585477 -2.802627 4.857292

C -3.108637 -3.353270 5.810670

C -3.891113 4.006730 -6.815936

C -3.554674 3.675836 -8.144425

C -4.872553 4.995894 -6.601041

C -4.180343 4.312269 -9.210145

H -2.797346 2.919893 -8.325321

C -5.489192 5.623268 -7.677201

H -5.138798 5.265204 -5.583997

C -5.159276 5.293962 -8.999799

H -3.904067 4.044703 -10.226939

H -6.241126 6.385606 -7.489674

C -3.718069 -3.993719 6.922281

C -3.343553 -3.664393 8.240931

C -4.709499 -4.978468 6.734684

C -3.942368 -4.298072 9.323594

H -2.578141 -2.911854 8.400768

C -5.298940 -5.603136 7.827522

H -5.004961 -5.246536 5.725409

C -4.931104 -5.275378 9.140471

H -3.636908 -4.031781 10.332343

H -6.059164 -6.362104 7.660911

C -5.858975 5.954928 -10.161968

H -5.214930 5.999732 -11.045376

H -6.761812 5.399033 -10.445018

H -6.169830 6.974720 -9.915690

C -5.601328 -5.933338 10.321566

H -6.493900 -5.373863 10.629067

H -5.922804 -6.951946 10.084179

H -4.933516 -5.980516 11.187018

O 2.829364 -2.762053 -4.744914

O 2.976154 2.741057 4.662866

C 2.537244 -2.466836 -6.103641

H 3.183026 -3.115817 -6.696372

H 1.488134 -2.680045 -6.344517

H 2.756857 -1.418752 -6.343266

C 2.721022 2.447039 6.029280

H 3.386631 3.092553 6.603578

H 1.680250 2.665587 6.299735

H 2.941914 1.397784 6.262522

S_0_-*anti*-(*M*,*M*)-**2**

0 1

C 12.362019 8.501629 5.874446

C 11.896176 9.823761 5.874448

C 13.670582 8.025554 5.401153

C 10.587612 10.299841 5.401158

C 13.713439 6.626089 5.573200

C 10.544754 11.699304 5.573224

C 12.411426 6.180958 6.096883

C 11.846769 12.144431 6.096904

C 11.633170 7.312052 6.340687

C 12.625029 11.013335 6.340690

C 14.735999 8.697725 4.718630

C 9.522195 9.627679 4.718625

C 15.943117 7.949802 4.476428

C 8.315074 10.375603 4.476440

C 16.002424 6.576436 4.822057

C 8.255766 11.748964 4.822090

C 14.901214 5.911409 5.317122

C 9.356976 12.413986 5.317159

C 14.685125 10.031300 4.231831

C 9.573072 8.294114 4.231800

H 13.767757 10.594604 4.318728

H 10.490442 7.730812 4.318681

C 15.775789 10.617712 3.629560

C 8.482408 7.707710 3.629522

H 15.698374 11.637285 3.263757

H 8.559824 6.688144 3.263699

C 16.986833 9.905124 3.469628

C 7.271361 8.420297 3.469608

H 17.842576 10.384764 3.004249

H 6.415617 7.940663 3.004224

C 17.057668 8.593186 3.873778

C 7.200523 9.732227 3.873783

H 17.966160 8.016510 3.720240

H 6.292028 10.308904 3.720257

C 11.891247 4.860550 6.312226

C 12.366948 13.464838 6.312256

C 10.611595 4.728394 6.942594

C 13.646606 13.596990 6.942613

C 9.920557 5.913684 7.436580

C 14.337651 12.411696 7.436580

C 10.437809 7.215742 7.148063

C 13.820399 11.109639 7.148055

C 12.540503 3.691780 5.839313

C 11.717685 14.633611 5.839361

H 13.467231 3.783237 5.291983

H 10.790952 14.542158 5.292039

C 11.979721 2.440151 5.993735

C 12.278466 15.885240 5.993791

H 12.493784 1.564559 5.608866

H 11.764398 16.760835 5.608935

C 10.731626 2.307968 6.624779

C 13.526566 16.017419 6.624825

H 10.279723 1.327610 6.742190

H 13.978468 16.997777 6.742242

C 10.065025 3.431733 7.076023

C 14.193173 14.893651 7.076051

H 9.087827 3.303934 7.525727

H 15.170375 15.021448 7.525748

C 8.750509 5.815405 8.225537

C 15.507706 12.509969 8.225526

H 8.345445 4.840381 8.468230

H 15.912771 13.484992 8.468226

C 9.801521 8.337757 7.730034

C 14.456697 9.987620 7.730005

H 10.226637 9.320120 7.587808

H 14.031582 9.005257 7.587773

C 8.119506 6.933432 8.735099

C 16.138718 11.391938 8.735069

C 8.663084 8.206395 8.499037

C 15.595141 10.118976 8.498998

H 7.225716 6.822485 9.341642

H 17.032514 11.502881 9.341604

H 8.202793 9.088269 8.934185

H 16.055439 9.237098 8.934131

H 16.932266 6.038175 4.659389

H 14.980161 4.857825 5.548415

H 9.278030 13.467567 5.548467

H 7.325922 12.287226 4.659433

*S_0_*-*syn*-(*M,M*)-**2**

0 1

C 12.340083 8.495168 6.036351

C 11.874547 9.814946 6.122708

C 13.627459 8.036086 5.501893

C 10.542781 10.296556 5.715932

C 13.663879 6.628387 5.596411

C 10.434192 11.648684 6.037338

C 12.368763 6.163545 6.121178

C 11.734388 12.099295 6.561985

C 11.605130 7.284530 6.442986

C 12.589014 10.980177 6.656942

C 14.671505 8.735627 4.813392

C 9.529216 9.650675 4.913579

C 15.865332 7.997042 4.493826

C 8.291870 10.338977 4.707266

C 15.928548 6.608318 4.770867

C 8.093951 11.669262 5.270715

C 14.839490 5.923590 5.267055

C 9.189157 12.349265 5.895611

C 14.607296 10.090555 4.393791

C 9.727160 8.411471 4.258393

H 13.695288 10.649956 4.542525

H 10.685648 7.920284 4.332091

C 15.678152 10.702680 3.781333

C 8.733345 7.824836 3.501663

H 15.592008 11.738455 3.466626

H 8.920796 6.874573 3.010971

C 16.880433 9.996099 3.545762

C 7.495241 8.469502 3.350124

H 17.721066 10.495767 3.073996

H 6.709289 8.015023 2.754461

C 16.960911 8.666135 3.884260

C 7.293841 9.707423 3.928641

H 17.861275 8.095664 3.671183

H 6.351614 10.211202 3.749935

C 6.846065 12.330568 5.209629

H 5.994086 11.830965 4.764783

C 6.665639 13.600161 5.725343

H 5.691730 14.076214 5.662339

C 7.736474 14.260347 6.350443

H 7.593711 15.245234 6.784842

C 8.967448 13.642034 6.434964

H 9.760537 14.135281 6.977291

C 11.838786 4.836848 6.262747

C 12.207948 13.385734 6.890756

C 10.559553 4.679116 6.888061

C 13.485673 13.535733 7.386805

C 9.879130 5.839104 7.451750

C 14.307457 12.414605 7.664416

C 10.410691 7.151437 7.245386

C 13.840879 11.090443 7.345458

C 12.477055 3.691204 5.722917

H 13.403895 3.804857 5.180307

C 11.906512 2.437377 5.807415

H 12.413110 1.580970 5.372662

C 10.658736 2.279471 6.433028

H 10.199097 1.297711 6.496084

C 10.002837 3.381184 6.949184

H 9.026142 3.235547 7.394397

C 8.705874 5.704562 8.230597

C 15.580398 12.580848 8.273945

H 8.288401 4.721060 8.409385

H 15.923938 13.589950 8.486525

C 9.787076 8.240549 7.900395

C 14.640579 9.995019 7.765608

H 10.224895 9.224560 7.826489

H 14.280989 8.987279 7.617306

C 8.085889 6.794818 8.809080

C 16.351756 11.494632 8.613094

C 8.645213 8.073700 8.657191

C 15.858481 10.190140 8.378127

H 7.188853 6.655521 9.404904

H 17.319856 11.632891 9.084860

H 8.194799 8.931280 9.147701

H 16.440957 9.329523 8.693334

H 13.868351 14.529032 7.605276

H 11.603184 14.265238 6.716516

H 14.920164 4.859212 5.440904

H 16.849518 6.074765 4.551889

*T_1_*-(*M,M*)-**2**

0 3

C 12.342751 8.462869 5.721399

C 11.915447 9.862528 5.721409

C 13.399237 7.916990 4.908801

C 10.858955 10.408416 4.908825

C 13.502066 6.530722 5.202599

C 10.756127 11.794680 5.202640

C 12.447366 6.198533 6.177108

C 11.810835 12.126858 6.177144

C 11.784396 7.393535 6.512104

C 12.473807 10.931853 6.512122

C 14.283305 8.549710 3.977499

C 9.974879 9.775707 3.977523

C 15.312667 7.735855 3.386930

C 8.945512 10.589568 3.386971

C 15.418922 6.367859 3.747125

C 8.839260 11.957560 3.747181

C 14.539094 5.772586 4.634240

C 9.719095 12.552822 4.634297

C 14.213721 9.915385 3.597811

C 10.044461 8.410036 3.597818

H 13.444613 10.548845 4.017800

H 10.813571 7.776570 4.017795

C 15.107861 10.448287 2.693305

C 9.150313 7.877144 2.693313

H 15.029856 11.496424 2.419886

H 9.228316 6.829009 2.419882

C 16.121580 9.648486 2.117522

C 8.136590 8.676951 2.117547

H 16.818295 10.083990 1.407694

H 7.439870 8.241455 1.407719

C 16.217099 8.319411 2.460198

C 8.041074 10.006022 2.460238

H 16.990113 7.691659 2.024404

H 7.268057 10.633779 2.024457

C 12.038699 4.947619 6.737688

C 12.219506 13.377766 6.737736

C 11.023065 4.950894 7.752896

C 13.235148 13.374480 7.752935

C 10.392940 6.204579 8.162158

C 13.865276 12.120790 8.162178

C 10.748183 7.428959 7.515630

C 13.510028 10.896418 7.515639

C 12.568016 3.702458 6.308347

C 11.690186 14.622932 6.308412

H 13.271573 3.680982 5.489126

H 10.986622 14.644417 5.489198

C 12.168157 2.510525 6.875409

C 12.090048 15.814858 6.875485

H 12.587075 1.573111 6.522194

H 11.671127 16.752277 6.522285

C 11.207378 2.516004 7.900700

C 13.050835 15.809368 7.900769

H 10.888810 1.583212 8.356178

H 13.369407 16.742155 8.356255

C 10.646717 3.710360 8.315053

C 13.611500 14.615007 8.315104

H 9.885879 3.676767 9.085063

H 14.372344 14.648591 9.085108

C 9.412424 6.249691 9.180393

C 14.845800 12.075666 9.180404

H 9.130048 5.342115 9.700005

H 15.128181 12.983237 9.700025

C 10.097297 8.622614 7.905275

C 14.160918 9.702758 7.905265

H 10.360007 9.553384 7.422934

H 13.898204 8.771993 7.422916

C 8.794025 7.430587 9.545764

C 15.464202 10.894766 9.545758

C 9.137586 8.627893 8.898576

C 15.120636 9.697468 8.898559

H 8.045502 7.428369 10.332405

H 16.212732 10.896975 10.332393

H 8.656512 9.560108 9.178838

H 15.601713 8.765249 9.178806

H 16.221776 5.778930 3.311864

H 14.690638 4.735029 4.900421

H 9.567553 13.590376 4.900491

H 8.036403 12.546494 3.311933

(*M,M*)-**2**-TS_rot_ (OS, S^2^=1.051288)

0 1

C 12.339948 8.462195 5.707688

C 11.918249 9.863202 5.707698

C 13.385110 7.908966 4.884520

C 10.873081 10.416440 4.884544

C 13.486305 6.523620 5.182235

C 10.771888 11.801783 5.182275

C 12.442311 6.198869 6.170996

C 11.815890 12.126522 6.171032

C 11.787319 7.397052 6.509134

C 12.470884 10.928335 6.509152

C 14.262310 8.535633 3.942752

C 9.995874 9.789784 3.942775

C 15.279811 7.715163 3.340926

C 8.978369 10.610260 3.340966

C 15.383919 6.347583 3.703315

C 8.874263 11.977837 3.703372

C 14.513191 5.759222 4.603880

C 9.744998 12.566187 4.603937

C 14.197455 9.901812 3.563857

C 10.060726 8.423608 3.563864

H 13.439089 10.540951 3.994618

H 10.819095 7.784465 3.994613

C 15.083206 10.428237 2.647413

C 9.174968 7.897194 2.647421

H 15.009094 11.476901 2.374929

H 9.249078 6.848533 2.374925

C 16.083741 9.621281 2.058656

C 8.174429 8.704157 2.058681

H 16.773836 10.051687 1.339316

H 7.484328 8.273759 1.339341

C 16.175634 8.292069 2.401748

C 8.082538 10.033365 2.401788

H 16.939393 7.659193 1.957133

H 7.318776 10.666246 1.957186

C 12.037911 4.951648 6.742983

C 12.220294 13.373737 6.743030

C 11.037052 4.962689 7.772646

C 13.221161 13.362684 7.772685

C 10.413554 6.219627 8.182037

C 13.844662 12.105742 8.182057

C 10.761478 7.439383 7.522921

C 13.496734 10.885993 7.522930

C 12.558239 3.703016 6.312853

C 11.699962 14.622373 6.312919

H 13.249414 3.675408 5.483339

H 11.008781 14.649991 5.483411

C 12.164710 2.515153 6.892782

C 12.093495 15.810230 6.892859

H 12.576507 1.574902 6.538730

H 11.681696 16.750485 6.538821

C 11.219678 2.528450 7.932528

C 13.038535 15.796921 7.932597

H 10.906519 1.598961 8.398388

H 13.351698 16.726405 8.398465

C 10.667252 3.726231 8.348024

C 13.590965 14.599136 8.348074

H 9.918042 3.698629 9.129589

H 14.340182 14.626729 9.129634

C 9.445486 6.272244 9.211760

C 14.812739 12.053113 9.211772

H 9.169262 5.368462 9.741205

H 15.088967 12.956889 9.741225

C 10.113320 8.635449 7.909768

C 14.144895 9.689923 7.909758

H 10.368126 9.562159 7.415534

H 13.890085 8.763218 7.415516

C 8.831204 7.455753 9.575619

C 15.427024 10.869600 9.575613

C 9.165505 8.647993 8.914351

C 15.092718 9.677368 8.914333

H 8.092265 7.459313 10.371267

H 16.165969 10.866031 10.371254

H 8.686320 9.581898 9.192202

H 15.571905 8.743460 9.192170

H 16.178332 5.753605 3.259506

H 14.663941 4.722065 4.871999

H 9.594250 13.603341 4.872068

H 8.079846 12.571820 3.259575

*S_0_*-*anti*-(*M,P*)-**2**

0 1

C 12.396113 8.505236 5.860807

C 11.938944 9.832098 5.872506

C 13.673835 8.010755 5.326003

C 10.637820 10.310034 5.387719

C 13.674834 6.602912 5.451524

C 10.571407 11.701172 5.608192

C 12.433673 6.189827 6.128530

C 11.857306 12.142152 6.174189

C 11.659175 7.329914 6.345573

C 12.651185 11.012989 6.382008

C 14.697885 8.671733 4.568682

C 9.597453 9.643433 4.664067

C 15.803813 7.872792 4.106206

C 8.384327 10.380198 4.420745

C 15.781752 6.468327 4.286983

C 8.297629 11.740707 4.809295

C 14.720959 5.837398 4.899140

C 9.377499 12.405446 5.350507

C 14.698104 10.045867 4.207367

C 9.679786 8.324790 4.142359

H 13.850999 10.664349 4.460190

H 10.604460 7.773674 4.233195

C 15.751462 10.611669 3.523844

C 8.609870 7.739461 3.503054

H 15.711987 11.665590 3.264735

H 8.709088 6.731675 3.110830

C 16.872642 9.837887 3.145622

C 7.391690 8.439090 3.338411

H 17.700883 10.300251 2.617292

H 6.552870 7.960155 2.842465

C 16.884409 8.491623 3.420937

C 7.292234 9.738045 3.777209

H 17.715863 7.869315 3.100158

H 6.377930 10.304899 3.621587

C 11.974845 4.889495 6.530044

C 12.348736 13.459951 6.460778

C 10.633066 4.766746 7.019256

C 13.613490 13.586552 7.121369

C 9.865436 5.965010 7.338522

C 14.328590 12.392279 7.556483

C 10.431349 7.257155 7.104662

C 13.845964 11.095664 7.192464

C 12.772116 3.719299 6.448118

C 11.682308 14.637935 6.036804

H 13.817915 3.805720 6.194801

H 10.768798 14.555580 5.466613

C 12.256455 2.469452 6.724014

C 12.210170 15.891686 6.269215

H 12.892050 1.592188 6.650515

H 11.683277 16.774874 5.920793

C 10.909314 2.339344 7.100827

C 13.441526 16.017687 6.933440

H 10.488177 1.357823 7.296574

H 13.867322 17.000156 7.113826

C 10.127089 3.468018 7.256666

C 14.125660 14.886376 7.336769

H 9.102816 3.341204 7.585357

H 15.090163 15.012304 7.813417

C 8.597956 5.892304 7.963590

C 15.493690 12.475450 8.354438

H 8.134764 4.928164 8.134634

H 15.871297 13.444922 8.656199

C 9.773609 8.387499 7.644161

C 14.520664 9.959066 7.697560

H 10.241944 9.357500 7.570437

H 14.129918 8.974843 7.487576

C 7.938483 7.022878 8.404562

C 16.156707 11.347251 8.796530

C 8.550480 8.280076 8.274137

C 15.654660 10.076022 8.475776

H 6.969167 6.931969 8.885423

H 17.045698 11.446979 9.412007

H 8.070970 9.167947 8.674797

H 16.144068 9.182536 8.851460

H 16.604880 5.880077 3.890285

H 14.697712 4.757355 4.918886

H 9.275036 13.449099 5.614851

H 7.363420 12.270654 4.644327

*S_0_*-*syn*-(*M,P*)-**2**

0 1

C 12.358113 8.498298 6.036075

C 11.901394 9.820647 6.146441

C 13.619845 8.035824 5.443838

C 10.580299 10.315402 5.728856

C 13.622535 6.622829 5.484695

C 10.456720 11.651917 6.104563

C 12.390919 6.169896 6.152688

C 11.741729 12.082374 6.681535

C 11.619259 7.293597 6.448289

C 12.604631 10.965551 6.735445

C 14.617503 8.739990 4.689927

C 9.590522 9.693634 4.880749

C 15.711907 7.972794 4.154144

C 8.355336 10.383228 4.669130

C 15.699314 6.560460 4.260845

C 8.137098 11.690994 5.276503

C 14.655586 5.892759 4.863974

C 9.211522 12.351420 5.957174

C 14.596842 10.129964 4.398624

C 9.811088 8.476510 4.192286

H 13.753318 10.726924 4.709104

H 10.770138 7.987098 4.274977

C 15.625225 10.737223 3.712456

C 8.838704 7.911632 3.392464

H 15.571344 11.802158 3.506520

H 9.041629 6.978494 2.875753

C 16.739558 9.991835 3.263294

C 7.602159 8.557225 3.230896

H 17.548577 10.486443 2.734409

H 6.833571 8.119903 2.600713

C 16.768098 8.633422 3.469708

C 7.380040 9.774347 3.844984

H 17.592939 8.034434 3.092436

H 6.439699 10.279400 3.660021

C 6.887961 12.349032 5.205952

H 6.051501 11.863289 4.718363

C 6.686222 13.597151 5.764321

H 5.711922 14.071093 5.692161

C 7.735954 14.237617 6.443283

H 7.576331 15.204942 6.910085

C 8.967471 13.621934 6.538169

H 9.742601 14.098830 7.119429

C 11.936304 4.846133 6.473617

C 12.195971 13.351832 7.093213

C 10.594364 4.689827 6.951365

C 13.461084 13.485416 7.624319

C 9.821502 5.864630 7.338471

C 14.294962 12.360206 7.843519

C 10.388027 7.170361 7.194940

C 13.852901 11.052588 7.434210

C 12.739390 3.686714 6.324547

H 13.785848 3.793402 6.080880

C 12.228787 2.420557 6.525456

H 12.868423 1.551765 6.402723

C 10.881606 2.263016 6.891689

H 10.464712 1.269922 7.029645

C 10.094048 3.377143 7.112159

H 9.070599 3.226471 7.433184

C 8.547695 5.750277 7.943914

C 15.558728 12.506593 8.476376

H 8.082626 4.777013 8.042976

H 15.881493 13.504606 8.761507

C 9.724814 8.260723 7.806351

C 14.676224 9.946427 7.772575

H 10.193427 9.233004 7.806756

H 14.342719 8.945419 7.541721

C 7.882622 6.848379 8.453377

C 16.348667 11.413911 8.745126

C 8.495591 8.111151 8.415615

C 15.886038 10.121036 8.407562

H 6.907998 6.725216 8.915993

H 17.309681 11.536745 9.235298

H 8.012120 8.970205 8.870667

H 16.486420 9.251862 8.659479

H 13.826232 14.467801 7.911391

H 11.585709 14.234460 6.959581

H 14.633568 4.813206 4.823121

H 16.513106 5.997837 3.811207

*T_1_*-(*M,P*)-**2**

0 3

C 12.311022 8.453188 5.734140

C 11.897466 9.857025 5.728237

C 13.338900 7.887235 4.898624

C 10.878992 10.417842 4.878215

C 13.437923 6.504088 5.207559

C 10.822906 11.815060 5.129994

C 12.411806 6.194692 6.219519

C 11.790597 12.118149 6.199846

C 11.767117 7.399681 6.555281

C 12.459808 10.922845 6.521025

C 14.204642 8.500856 3.937684

C 10.047524 9.804171 3.887495

C 15.204314 7.668009 3.322957

C 9.181258 10.665351 3.126871

C 15.304817 6.302394 3.693273

C 9.190333 12.062703 3.371111

C 14.447984 5.727784 4.615648

C 9.991437 12.631701 4.345709

C 14.146657 9.865675 3.552678

C 10.018010 8.413352 3.606704

H 13.403681 10.515363 3.993814

H 10.651383 7.740587 4.168332

C 15.020685 10.378534 2.617505

C 9.185729 7.902063 2.633276

H 14.951732 11.426503 2.341024

H 9.181993 6.833046 2.441875

C 16.002205 9.558853 2.014609

C 8.337241 8.749902 1.884928

H 16.682828 9.978597 1.280098

H 7.688297 8.331149 1.121744

C 16.087960 8.230918 2.363929

C 8.338509 10.103546 2.131123

H 16.837673 7.588289 1.909499

H 7.690936 10.767829 1.564624

C 12.016196 4.956692 6.817256

C 12.086947 13.336400 6.888569

C 11.036011 4.984603 7.866437

C 13.175084 13.346368 7.825565

C 10.419432 6.247874 8.266981

C 13.915318 12.118529 8.109797

C 10.756438 7.457255 7.583676

C 13.531989 10.890728 7.486237

C 12.526773 3.700862 6.396411

C 11.333980 14.525994 6.707472

H 13.201217 3.659445 5.553880

H 10.462713 14.508643 6.069449

C 12.144546 2.522616 7.002878

C 11.654974 15.694593 7.365863

H 12.548665 1.576595 6.655423

H 11.057053 16.587585 7.210779

C 11.221346 2.553120 8.061664

C 12.749966 15.718528 8.246053

H 10.917804 1.631455 8.548932

H 13.014180 16.635008 8.764993

C 10.677997 3.757783 8.469178

C 13.479249 14.565481 8.472056

H 9.945558 3.743197 9.266815

H 14.297486 14.610052 9.180151

C 9.466982 6.316298 9.310250

C 15.001390 12.094356 9.015435

H 9.199267 5.420859 9.857914

H 15.326698 13.009773 9.494589

C 10.111120 8.658375 7.959283

C 14.229823 9.706695 7.820048

H 10.355680 9.576598 7.444180

H 13.939637 8.771534 7.362738

C 8.856619 7.504915 9.664012

C 15.674603 10.925266 9.316084

C 9.178288 8.686335 8.977557

C 15.279932 9.718648 8.717170

H 8.129809 7.520630 10.470600

H 16.504165 10.943575 10.016505

H 8.700999 9.623845 9.246396

H 15.798592 8.794268 8.952769

H 16.085900 5.698985 3.238654

H 14.596851 4.691747 4.888881

H 9.985701 13.707280 4.460923

H 8.550025 12.698188 2.765276

(*M,P*)-**2**-TS_rot_ (OS, S^2^=1.051869)

0 1

C 12.358981 8.467966 5.720095

C 11.948068 9.872228 5.725665

C 13.375430 7.904096 4.868805

C 10.920937 10.440519 4.889979

C 13.429481 6.507214 5.121739

C 10.824620 11.823724 5.198664

C 12.462989 6.206650 6.193678

C 11.851478 12.131468 6.210693

C 11.796175 7.403000 6.514809

C 12.493842 10.925658 6.546797

C 14.206354 8.515466 3.876340

C 10.053872 9.828332 3.929431

C 15.069918 7.652200 3.115030

C 9.056065 10.663012 3.314195

C 15.058743 6.255078 3.360447

C 8.958352 12.028933 3.684096

C 14.258228 5.688352 4.336851

C 9.816176 12.601983 4.606520

C 14.237787 9.906071 3.594498

C 10.108841 8.463092 3.545347

H 13.606349 10.580263 4.156620

H 10.850106 7.811995 3.987306

C 15.069457 10.415197 2.619453

C 9.233962 7.951682 2.610212

H 15.074793 11.484060 2.427239

H 9.300558 6.903389 2.334381

C 15.915287 9.565286 1.870398

C 8.254453 8.773261 2.006576

H 16.563760 9.982358 1.105894

H 7.573106 8.354623 1.272103

C 15.912068 8.211820 2.117537

C 8.171436 10.101545 2.355221

H 16.557605 7.546024 1.550490

H 7.423176 10.745536 1.900327

C 12.166141 4.989449 6.884168

C 12.249211 13.368988 6.808177

C 11.079708 4.982003 7.823132

C 13.229153 13.339565 7.857514

C 10.341574 6.211107 8.107179

C 13.843514 12.075332 8.258285

C 10.725516 7.437644 7.481498

C 13.504424 10.866467 7.575077

C 12.917153 3.798654 6.703029

C 11.740937 14.625613 6.386998

H 13.787271 3.814090 6.063394

H 11.066728 14.668079 5.544334

C 12.595812 2.631244 7.363374

C 12.125134 15.803307 6.993313

H 13.192254 1.737275 7.208215

H 11.722749 16.749968 6.645589

C 11.502433 2.609782 8.245630

C 13.048052 15.771395 8.052302

H 11.237977 1.694254 8.766126

H 13.353096 16.692623 8.539462

C 10.775104 3.764044 8.471634

C 13.589202 14.565873 8.460142

H 9.958127 3.721417 9.181304

H 14.321503 14.579294 9.257929

C 9.256971 6.237840 9.014514

C 14.795839 12.005326 9.301574

H 8.931224 5.323460 9.495348

H 15.065112 12.900333 9.849183

C 10.029597 8.623008 7.814693

C 14.147763 9.664240 7.950649

H 10.320118 9.557140 7.355480

H 13.901694 8.746450 7.435482

C 8.585747 7.408196 9.314678

C 15.404152 10.815670 9.655355

C 8.980904 8.613540 8.713477

C 15.080543 9.634752 8.968903

H 7.757275 7.391867 10.016438

H 16.130927 10.798738 10.461952

H 8.463667 9.538839 8.948599

H 15.556313 8.696475 9.237756

H 15.696944 5.617938 2.754125

H 14.262261 4.612874 4.453039

H 9.669375 13.638374 4.879507

H 8.178608 12.633847 3.229175

*anti*-**2**-TS_inv_

0 1

C -0.920326 -9.239734 -2.154679

C -2.113636 -9.345613 -2.828189

C -3.152163 -8.395399 -2.632720

C -2.977373 -7.333429 -1.677369

C -1.706597 -7.224327 -1.051187

C -0.712335 -8.149689 -1.278021

C -4.341705 -8.458958 -3.398397

C -5.331203 -7.507341 -3.277054

C -5.218409 -6.493890 -2.302986

C -4.086974 -6.452849 -1.457577

C -6.098368 -5.358483 -1.960059

C -5.495786 -4.647376 -0.919110

C -4.224942 -5.293567 -0.570192

C -7.345030 -4.921907 -2.529163

C -7.937221 -3.715005 -2.028350

C -7.387827 -3.070487 -0.841569

C -6.197495 -3.582481 -0.239205

C -8.036103 -1.976216 -0.221071

C -7.573027 -1.429675 0.959669

C -6.450912 -1.989087 1.591613

C -5.786652 -3.046664 1.004469

C -3.313800 -4.874265 0.412776

C -2.746125 -5.698822 1.488158

C -1.884386 -4.909407 2.251987

C -1.828263 -3.570024 1.642449

C -2.724908 -3.534724 0.551408

C -1.999024 -1.237902 0.065741

C -2.766270 -2.394926 -0.318350

C -3.080859 -7.043207 1.901128

C -2.285297 -7.647146 2.924831

C -1.324733 -6.841860 3.670643

C -1.216611 -5.437100 3.408224

C -4.156435 -7.785372 1.359380

C -4.377324 -9.101529 1.711141

C -3.530295 -9.729929 2.638095

C -2.521264 -9.005810 3.242297

H -0.130734 -9.967657 -2.314713

H -2.277024 -10.150598 -3.540077

H -1.505811 -6.392424 -0.393116

H 0.247691 -8.031473 -0.784322

H -4.449831 -9.259023 -4.125832

H -6.180785 -7.566620 -3.940843

H -8.938613 -1.565426 -0.656741

H -8.097924 -0.593901 1.412487

H -6.110626 -1.603236 2.547750

H -4.942820 -3.483511 1.516688

H -4.827595 -7.310864 0.659650

H -5.211212 -9.643018 1.274968

H -3.688152 -10.769594 2.908724

H -1.920079 -9.495795 3.998477

C -9.085459 -3.211147 -2.681432

C -9.673586 -3.871461 -3.742877

C -7.996274 -5.593385 -3.595438

C -9.132833 -5.087085 -4.191752

H -9.600313 -5.626109 -5.010267

H -9.527789 -2.280441 -2.348201

H -10.556046 -3.454741 -4.218915

H -7.601929 -6.528210 -3.959482

C -0.541592 -7.385848 4.714677

H -0.570695 -8.450607 4.911129

C -0.444635 -4.642265 4.293787

H -0.446686 -3.568456 4.181917

C 0.279412 -5.202188 5.326340

H 0.861907 -4.567407 5.987186

C 0.255948 -6.593701 5.518763

H 0.840353 -7.045919 6.314394

C -1.138244 -1.305132 1.188567

C -1.013456 -2.457773 1.933213

H -0.266759 -2.497015 2.713388

H -0.533772 -0.435707 1.432822

C -3.476471 -2.322138 -1.546914

H -3.997096 -3.192694 -1.914968

C -2.067826 -0.053797 -0.717492

H -1.494909 0.811963 -0.395448

C -3.507007 -1.165387 -2.293664

H -4.060192 -1.150275 -3.228108

C -2.818500 -0.006217 -1.867528

H -2.860877 0.901811 -2.461332

*syn*-**2**-TS_inv_

0 1

C -1.056441 -9.443999 -2.385712

C -2.252082 -9.449606 -3.063922

C -3.243140 -8.463234 -2.810588

C -3.015980 -7.466993 -1.797707

C -1.749501 -7.467857 -1.155314

C -0.800833 -8.425730 -1.438764

C -4.439108 -8.431076 -3.568492

C -5.382883 -7.443329 -3.387704

C -5.212853 -6.482080 -2.370255

C -4.076042 -6.538448 -1.529107

C -6.029864 -5.316107 -1.978840

C -5.389247 -4.688122 -0.910712

C -4.143194 -5.403236 -0.604891

C -7.241627 -4.774349 -2.533398

C -7.759165 -3.557060 -1.976753

C -7.180560 -3.007753 -0.756371

C -6.028311 -3.622290 -0.176680

C -7.763609 -1.908770 -0.082062

C -7.276809 -1.456733 1.128828

C -6.201321 -2.122188 1.739466

C -5.600438 -3.187628 1.100992

C -3.187963 -5.001019 0.342718

C -2.622354 -5.792255 1.442578

C -1.794454 -4.935605 2.204984

C -1.762787 -3.613538 1.558597

C -2.626499 -3.646653 0.464689

C -2.200044 -1.264634 0.011787

C -2.739931 -2.514938 -0.425745

C -2.960709 -7.097828 1.933320

C -2.297784 -7.553925 3.127371

C -1.447161 -6.673528 3.840232

C -1.234464 -5.378686 3.419614

C -3.918186 -7.968923 1.349344

C -4.148673 -9.228962 1.856396

C -3.440467 -9.698975 2.986034

C -2.543598 -8.867010 3.612596

H -0.303011 -10.198460 -2.590681

H -2.453580 -10.201625 -3.822445

H -1.515512 -6.696095 -0.437776

H 0.159153 -8.390500 -0.932195

H -4.590399 -9.187461 -4.333975

H -6.244030 -7.433548 -4.039076

H -8.634292 -1.418357 -0.499913

H -7.750961 -0.613687 1.622363

H -5.849738 -1.812885 2.719027

H -4.797849 -3.711725 1.598271

H -4.488570 -7.638251 0.495193

H -4.891641 -9.864692 1.383858

H -3.624991 -10.698534 3.367738

H -2.018097 -9.193918 4.506168

C -8.866796 -2.948541 -2.610451

C -9.484841 -3.514595 -3.708710

C -7.920642 -5.344918 -3.640092

C -9.016007 -4.736292 -4.217876

H -9.505964 -5.199608 -5.068991

H -9.251646 -2.009235 -2.232812

H -10.334215 -3.018560 -4.168636

H -7.578030 -6.279100 -4.055091

C -1.360420 -1.212233 1.203507

C -1.042894 -2.420768 1.905348

C -3.336096 -2.578875 -1.707682

H -3.645904 -3.535337 -2.100962

C -2.429622 -0.120309 -0.788111

H -2.054994 0.844944 -0.469785

C -3.520101 -1.447670 -2.476500

H -3.985489 -1.530541 -3.453912

C -3.094442 -0.198527 -1.996324

H -3.250242 0.698485 -2.588092

C -0.049158 -2.374491 2.916069

C -0.769362 -0.008460 1.651591

C 0.534238 -1.185778 3.304546

H 1.292005 -1.181771 4.082223

C 0.145753 0.014988 2.687124

H 0.582836 0.957981 3.001450

H -0.991454 -7.024115 4.762365

H 0.287026 -3.293749 3.372300

H -1.019638 0.927316 1.166957

H -0.664576 -4.711070 4.049985

*S_0_*-(*M,M*)-**3**

0 1

C 12.361408 8.503869 6.080093

C 11.896806 9.821544 6.080105

C 13.695833 8.034395 5.672872

C 10.562376 10.291014 5.672899

C 13.770377 6.660901 5.900164

C 10.487827 11.664506 5.900201

C 12.420459 6.184784 6.260321

C 11.837747 12.140625 6.260344

C 11.616650 7.301086 6.487168

C 12.641553 11.024325 6.487203

C 14.752807 8.703277 4.951421

C 9.505401 9.622129 4.951454

C 16.001711 8.021269 4.803135

C 8.256492 10.304130 4.803181

C 16.182531 6.707258 5.407487

C 8.075667 11.618133 5.407551

C 15.047825 6.002672 5.924007

C 9.210371 12.322721 5.924072

C 14.584188 9.951598 4.307018

C 9.674022 8.373812 4.307044

H 13.622534 10.442813 4.347783

H 10.635678 7.882601 4.347802

C 15.611436 10.542809 3.599270

C 8.646773 7.782600 3.599300

H 15.447772 11.499079 3.111752

H 8.810438 6.826333 3.111776

C 16.852618 9.894746 3.489155

C 7.405586 8.430657 3.489195

H 17.660592 10.351209 2.925386

H 6.597610 7.974193 2.925430

C 17.030855 8.652855 4.068606

C 7.227347 9.672544 4.068656

H 17.976742 8.143710 3.924481

H 6.281455 10.181683 3.924542

C 17.453255 6.100814 5.523206

C 6.804938 12.224561 5.523292

H 18.330735 6.621366 5.157969

H 5.927461 11.704007 5.158052

C 17.620276 4.871044 6.133601

C 6.637909 13.454317 6.133714

H 18.612775 4.437662 6.213072

H 5.645405 13.887686 6.213203

C 16.512562 4.204706 6.684904

C 7.745619 14.120651 6.685027

H 16.645947 3.263599 7.209818

H 7.612228 15.061743 7.209968

C 15.254799 4.765277 6.586172

C 9.003387 13.560095 6.586273

H 14.415541 4.277127 7.064141

H 9.842641 14.048240 7.064254

C 11.838812 4.870694 6.236362

C 12.419401 13.454713 6.236370

C 10.512706 4.707441 6.751680

C 13.745498 13.617967 6.751708

C 9.828450 5.844140 7.355213

C 14.429740 12.481274 7.355268

C 10.373260 7.158776 7.207469

C 13.884931 11.166638 7.207526

C 12.454636 3.777196 5.574926

C 11.803600 14.548201 5.574897

H 13.414826 3.923568 5.097754

H 10.843423 14.401825 5.097701

C 11.827057 2.551535 5.475755

C 12.431187 15.773856 5.475717

H 12.314156 1.734978 4.951366

H 11.944105 16.590406 4.951300

C 10.545926 2.375438 6.025883

C 13.712306 15.949958 6.025871

H 10.045078 1.415245 5.946063

H 14.213161 16.910146 5.946045

C 9.903834 3.437905 6.635553

C 14.354379 14.887498 6.635572

H 8.893617 3.292753 6.999879

H 15.364590 15.032650 6.999916

C 8.629923 5.690333 8.088349

C 15.628253 12.635088 8.088424

H 8.212671 4.700417 8.232103

H 16.045505 13.625005 8.232174

C 9.720509 8.236686 7.850931

C 14.537667 10.088734 7.851015

H 10.161033 9.222617 7.810532

H 14.097142 9.102804 7.810622

C 7.988741 6.769275 8.666781

C 16.269423 11.556152 8.666881

C 8.548725 8.052639 8.557136

C 15.709438 10.272787 8.557241

H 7.072475 6.617845 9.229399

H 17.185679 11.707586 9.229515

H 8.075503 8.900050 9.043826

H 16.182648 9.425382 9.043951

*T_1_*-(*M,M*)-**3**

0 3

C 12.370642 8.472709 6.079703

C 11.884654 9.851714 6.080249

C 13.158131 7.853807 5.043295

C 10.539368 10.274989 5.782710

C 13.483141 6.551367 5.464333

C 10.470944 11.667606 5.970020

C 12.712026 6.278636 6.694277

C 11.848811 12.152808 6.188850

C 12.146517 7.495887 7.115676

C 12.667864 11.024617 6.377373

C 13.615052 8.373082 3.778880

C 9.384915 9.510831 5.382211

C 14.523408 7.576138 3.014765

C 8.126623 10.185390 5.306582

C 15.062701 6.344807 3.586012

C 8.018852 11.569169 5.761402

C 14.553819 5.843180 4.831913

C 9.198884 12.306245 6.117941

C 13.155169 9.596924 3.239715

C 9.458249 8.150540 5.002493

H 12.453104 10.196011 3.803619

H 10.410514 7.639089 5.039083

C 13.561342 10.027409 1.991324

C 8.341959 7.470396 4.555262

H 13.188628 10.968306 1.597850

H 8.426578 6.427772 4.264065

C 14.437988 9.239051 1.228310

C 7.107719 8.133620 4.460344

H 14.746666 9.565232 0.239694

H 6.231347 7.607847 4.093501

C 14.902253 8.038847 1.734049

C 7.010314 9.463358 4.826577

H 15.563173 7.440183 1.118207

H 6.050933 9.957439 4.726775

C 16.126981 5.643643 2.977120

C 6.768532 12.206324 5.922071

H 16.534541 5.996700 2.037442

H 5.859171 11.677232 5.663490

C 16.706221 4.534814 3.568186

C 6.658802 13.481264 6.448243

H 17.529497 4.028211 3.073511

H 5.678717 13.933851 6.565420

C 16.260725 4.095730 4.827240

C 7.809502 14.167990 6.873418

H 16.752015 3.263477 5.322165

H 7.722982 15.143509 7.342288

C 15.211972 4.743968 5.445785

C 9.050174 13.585754 6.717112

H 14.904603 4.437117 6.436951

H 9.929314 14.093477 7.091769

C 12.324917 5.054848 7.326972

C 12.440387 13.447282 6.039666

C 11.614538 5.125049 8.573284

C 13.821788 13.612301 6.396588

C 11.259737 6.421668 9.144419

C 14.604444 12.466669 6.853008

C 11.464946 7.612441 8.380183

C 14.046280 11.152532 6.778278

C 12.504093 3.786204 6.713207

C 11.755143 14.537110 5.438824

H 12.935455 3.740804 5.721789

H 10.751913 14.381905 5.064000

C 12.096867 2.622747 7.332246

C 12.357714 15.767854 5.281106

H 12.238063 1.666613 6.837441

H 11.814354 16.581878 4.810908

C 11.475157 2.684184 8.591689

C 13.684678 15.953821 5.706604

H 11.154033 1.772639 9.086762

H 14.165286 16.920340 5.588319

C 11.228429 3.910675 9.182627

C 14.397444 14.891923 6.234529

H 10.690080 3.929044 10.122616

H 15.437644 15.049317 6.493272

C 10.674247 6.543504 10.425144

C 15.926605 12.603713 7.333641

H 10.536510 5.662522 11.041090

H 16.364721 13.589968 7.432809

C 11.053172 8.853351 8.919210

C 14.841155 10.046694 7.159405

H 11.222354 9.760400 8.354944

H 14.419502 9.051376 7.123577

C 10.280809 7.768800 10.930775

C 16.683555 11.506590 7.701280

C 10.466833 8.932959 10.167644

C 16.137093 10.216336 7.607182

H 9.835735 7.828489 11.919403

H 17.695808 11.646146 8.068575

H 10.165157 9.899008 10.561041

H 16.724174 9.350924 7.899447

(*M,M*)-**3**-TS_rot_ (OS, S^2^=1.032362)

0 1

C 0.042856 -0.335704 -0.270723

C 1.068198 -1.226077 -0.756982

C 0.703304 -2.538801 -0.406981

C -0.672710 -2.489478 0.126572

C -1.003906 -1.135579 0.316693

C -3.152663 -1.770197 1.335563

C -2.227875 -0.742954 0.969225

C 2.300676 -0.945213 -1.449934

C 3.198500 -2.031924 -1.690053

C 2.926916 -3.339388 -1.098558

C 1.683012 -3.581906 -0.423469

C 2.626659 0.336453 -1.951000

C 3.778328 0.547700 -2.684371

C 4.646550 -0.523240 -2.951475

C 4.356309 -1.783244 -2.461815

H 1.952071 1.163219 -1.776191

H 4.001339 1.540433 -3.063558

H 5.543425 -0.365805 -3.542969

H 5.036267 -2.595459 -2.690752

C 3.885651 -4.376792 -1.109290

H 4.834317 -4.226236 -1.610353

C 1.522331 -4.795103 0.297887

H 0.635212 -4.930813 0.902558

C 2.490552 -5.777373 0.279537

H 2.346371 -6.692307 0.846155

C 3.673253 -5.576393 -0.453531

H 4.438782 -6.346197 -0.475976

C -2.915000 -3.145330 0.904753

C -1.678419 -3.498133 0.265804

C -3.899725 -4.149419 1.037477

H -4.843740 -3.916099 1.515070

C -1.549304 -4.792566 -0.304989

H -0.666556 -5.022024 -0.887324

C -2.542060 -5.740819 -0.171185

C -3.718509 -5.423795 0.530252

H -4.503286 -6.165777 0.643055

H -2.421697 -6.720302 -0.624036

C -2.520637 0.597164 1.313357

H -1.825605 1.379940 1.042393

C -4.302893 -1.402043 2.070028

H -5.002996 -2.163669 2.392897

C -3.665684 0.923400 2.014261

H -3.862969 1.959578 2.272044

C -4.560491 -0.085718 2.405482

H -5.452371 0.163773 2.972351

C 0.061641 1.123433 -0.357891

C -0.538303 1.917351 -1.402153

C -0.360235 3.273074 -1.071829

C 0.544559 3.330528 0.093718

C 0.684828 2.019756 0.584793

C 2.026545 2.837923 2.479092

C 1.386056 1.745983 1.814188

C -1.249677 1.517772 -2.590548

C -1.859874 2.539599 -3.383165

C -1.885596 3.915844 -2.894930

C -1.156517 4.275618 -1.711357

C -1.316166 0.176110 -3.032800

C -1.935495 -0.156665 -4.222117

C -2.507053 0.847230 -5.020615

C -2.466121 2.164888 -4.603849

H -0.855413 -0.602578 -2.440711

H -1.966310 -1.193868 -4.541957

H -2.978730 0.592660 -5.964940

H -2.904555 2.922419 -5.242894

C -2.667044 4.914110 -3.518088

H -3.229037 4.675604 -4.413054

C -1.347111 5.570243 -1.158516

H -0.891582 5.804427 -0.205278

C -2.134607 6.512595 -1.786571

H -2.270160 7.492365 -1.338662

C -2.780082 6.189089 -2.992913

H -3.398907 6.926420 -3.495442

C 2.091363 4.143902 1.828484

C 1.370487 4.379494 0.609184

C 2.902892 5.186708 2.327925

H 3.459830 5.041240 3.245733

C 1.597140 5.592187 -0.095112

H 1.146456 5.723168 -1.070173

C 2.413004 6.580011 0.415997

C 3.051659 6.385401 1.653249

H 3.692803 7.159530 2.064096

H 2.575849 7.494430 -0.146378

C 1.413862 0.466235 2.415440

H 0.929498 -0.364437 1.920859

C 2.623011 2.596011 3.737523

H 3.083813 3.412227 4.281502

C 2.024629 0.261731 3.637732

H 2.025423 -0.729650 4.080676

C 2.626079 1.337703 4.310482

H 3.091149 1.185505 5.279802

*S_0_*-(*M,P*)-**3**

0 1

C 12.363432 8.488490 6.078929

C 11.897404 9.810647 6.078983

C 13.628860 7.999890 5.519043

C 10.576213 10.275706 5.619600

C 13.739940 6.636104 5.791879

C 10.563274 11.670020 5.717866

C 12.459515 6.183249 6.370000

C 11.775288 12.096619 6.436145

C 11.686804 7.313337 6.639919

C 12.636843 11.000660 6.537365

C 14.578970 8.651845 4.650667

C 9.501546 9.574222 4.949793

C 15.787182 7.955446 4.332599

C 8.334033 10.324065 4.591704

C 16.051038 6.655401 4.938033

C 8.387106 11.780129 4.579867

C 15.010329 5.978100 5.652584

C 9.565132 12.447836 5.040581

C 14.337626 9.906527 4.043179

C 9.520491 8.194412 4.636340

H 13.402492 10.414881 4.231073

H 10.427866 7.629934 4.780342

C 15.256856 10.480025 3.187320

C 8.404979 7.543213 4.149695

H 15.039714 11.439845 2.728429

H 8.456406 6.480478 3.933417

C 16.454482 9.808978 2.890621

C 7.211963 8.254173 3.942024

H 17.174038 10.249973 2.207386

H 6.320006 7.742342 3.593578

C 16.701711 8.567694 3.445991

C 7.192778 9.620754 4.139557

H 17.610159 8.046455 3.167261

H 6.278906 10.162017 3.925181

C 17.322949 6.043260 4.874154

C 7.361654 12.566193 4.003715

H 18.131871 6.541481 4.352954

H 6.447378 12.095090 3.663822

C 17.584382 4.838183 5.500243

C 7.513520 13.925282 3.803035

H 18.576234 4.400611 5.437645

H 6.711320 14.495912 3.344660

C 16.578922 4.205402 6.250841

C 8.725904 14.554306 4.138126

H 16.794018 3.287554 6.789660

H 8.877555 15.605941 3.914698

C 15.321652 4.770155 6.328399

C 9.730757 13.824777 4.738705

H 14.569720 4.309635 6.955574

H 10.673844 14.302712 4.968181

C 11.887365 4.872344 6.512644

C 12.067633 13.329318 7.110669

C 10.652682 4.744508 7.227830

C 13.404236 13.545663 7.571700

C 10.038708 5.922006 7.830191

C 14.355794 12.442228 7.563474

C 10.537959 7.223178 7.508282

C 13.913389 11.126268 7.208365

C 12.406638 3.736405 5.839766

C 11.077473 14.301375 7.409323

H 13.280584 3.851004 5.212120

H 10.042747 14.084338 7.179643

C 11.786196 2.505888 5.920765

C 11.405433 15.500507 8.006952

H 12.197382 1.656157 5.384185

H 10.629583 16.227196 8.227937

C 10.606782 2.365811 6.671872

C 12.744746 15.768197 8.342231

H 10.112247 1.401268 6.737054

H 13.013939 16.716249 8.798284

C 10.050596 3.467985 7.295120

C 13.712939 14.801726 8.144757

H 9.108548 3.346635 7.816703

H 14.720839 15.006390 8.484775

C 8.942963 5.823606 8.717289

C 15.685871 12.607648 8.016344

H 8.566349 4.847168 8.999058

H 16.060809 13.601875 8.228520

C 9.934013 8.351219 8.111784

C 14.760478 10.038057 7.525893

H 10.338847 9.334799 7.919872

H 14.404979 9.029921 7.384489

C 8.352535 6.944776 9.269292

C 16.524818 11.529358 8.217312

C 8.859000 8.219709 8.968190

C 16.037929 10.228176 8.012937

H 7.516093 6.835689 9.953039

H 17.540745 11.688170 8.566088

H 8.422324 9.103205 9.423969

H 16.661738 9.366991 8.232131

*T_1_*-(*M,P*)-**3**

0 3

C 12.375168 8.473719 6.080177

C 11.886630 9.853041 6.079388

C 13.379063 7.930891 5.198440

C 10.773276 10.355905 5.312897

C 13.611120 6.595687 5.576558

C 10.683520 11.741849 5.538070

C 12.591529 6.235616 6.582141

C 11.630611 12.077970 6.619930

C 11.934975 7.420681 6.961792

C 12.434343 10.945379 6.845326

C 14.115232 8.548840 4.123416

C 9.872957 9.678544 4.412864

C 15.156528 7.791838 3.500542

C 8.932205 10.470095 3.682280

C 15.553148 6.504739 4.065291

C 9.015529 11.927152 3.736124

C 14.793989 5.921564 5.135419

C 9.924744 12.564741 4.646263

C 13.816508 9.838821 3.626484

C 9.843597 8.272235 4.265775

H 13.032238 10.419767 4.091183

H 10.551314 7.665056 4.812568

C 14.489755 10.362715 2.539694

C 8.909469 7.654803 3.456425

H 14.233977 11.352512 2.173722

H 8.904340 6.572528 3.368612

C 15.486696 9.607708 1.901085

C 7.959438 8.425420 2.766998

H 16.004413 10.006912 1.034112

H 7.210458 7.943476 2.145871

C 15.808159 8.350209 2.377313

C 7.976952 9.803072 2.881899

H 16.574693 7.783368 1.862027

H 7.227795 10.375258 2.347324

C 16.717193 5.828838 3.636496

C 8.269812 12.749834 2.862724

H 17.311594 6.241506 2.830258

H 7.573807 12.300312 2.164773

C 17.157621 4.669088 4.248894

C 8.433540 14.123275 2.832831

H 18.063094 4.183799 3.897119

H 7.849079 14.721171 2.139968

C 16.461243 4.149619 5.354255

C 9.389219 14.735608 3.662487

H 16.837688 3.275689 5.877208

H 9.565670 15.805019 3.597885

C 15.308972 4.770331 5.789581

C 10.122322 13.967872 4.543220

H 14.802425 4.398378 6.670476

H 10.891842 14.433585 5.144776

C 12.092846 4.968224 7.021810

C 11.700705 13.194911 7.512004

C 11.136404 4.945119 8.092445

C 12.807946 13.272635 8.422828

C 10.638821 6.195754 8.659163

C 13.791140 12.194061 8.476667

C 10.974951 7.439150 8.037625

C 13.560108 10.986878 7.745461

C 12.412803 3.749485 6.365505

C 10.662839 14.159639 7.614829

H 13.039942 3.778708 5.484174

H 9.772252 14.036443 7.012681

C 11.904458 2.542877 6.799329

C 10.747486 15.217358 8.496074

H 12.158585 1.626800 6.274767

H 9.936388 15.936326 8.560485

C 11.037037 2.509390 7.905255

C 11.874845 15.343955 9.326518

H 10.636016 1.563138 8.255856

H 11.951267 16.176175 10.019846

C 10.653505 3.688135 8.519674

C 12.867692 14.380932 9.296802

H 9.932155 3.635712 9.326241

H 13.690872 14.470315 9.995383

C 9.781938 6.220832 9.783060

C 14.952864 12.278019 9.277520

H 9.540447 5.298029 10.297380

H 15.173451 13.194137 9.812758

C 10.399851 8.630877 8.536863

C 14.469626 9.913784 7.891876

H 10.646887 9.575815 8.073590

H 14.303440 8.996729 7.344158

C 9.244548 7.401480 10.261320

C 15.834981 11.219619 9.392033

C 9.547761 8.615579 9.624319

C 15.583748 10.022981 8.701597

H 8.591601 7.386803 11.128778

H 16.719974 11.317323 10.013548

H 9.127142 9.546646 9.992082

H 16.270167 9.186169 8.788947

(*M,P*)-**3**-TS_rot_ (OS, S^2^=1.051182)

0 1

C 12.375298 8.473881 6.080328

C 11.886949 9.852894 6.079457

C 13.379848 7.930456 5.198765

C 10.772766 10.356163 5.313336

C 13.611645 6.595532 5.576924

C 10.683344 11.741895 5.538164

C 12.591150 6.235207 6.581866

C 11.630924 12.078278 6.619835

C 11.934711 7.419989 6.961627

C 12.434987 10.946181 6.844902

C 14.116518 8.548532 4.124271

C 9.872172 9.678686 4.413767

C 15.158096 7.791649 3.501791

C 8.930881 10.470039 3.683709

C 15.554353 6.504463 4.066567

C 9.014156 11.927092 3.737359

C 14.794698 5.921268 5.136272

C 9.924038 12.564768 4.646686

C 13.817931 9.838632 3.627501

C 9.843130 8.272347 4.266679

H 13.033459 10.419447 4.092067

H 10.551509 7.665390 4.812875

C 14.491628 10.362718 2.541107

C 8.908579 7.654699 3.458016

H 14.236006 11.352560 2.175149

H 8.903623 6.572426 3.370183

C 15.488953 9.607847 1.902883

C 7.957931 8.425131 2.769188

H 16.007100 10.007267 1.036264

H 7.208670 7.943003 2.148541

C 15.810291 8.350281 2.379010

C 7.975259 9.802793 2.883941

H 16.577164 7.783626 1.864024

H 7.225719 10.374796 2.349707

C 16.718564 5.828519 3.638215

C 8.267781 12.749666 2.864379

H 17.313306 6.241180 2.832224

H 7.571167 12.300067 2.167085

C 17.158653 4.668732 4.250750

C 8.431609 14.123069 2.834103

H 18.064252 4.183399 3.899357

H 7.846662 14.720900 2.141593

C 16.461747 4.149244 5.355768

C 9.387995 14.735469 3.662896

H 16.837914 3.275266 5.878841

H 9.564494 15.804851 3.597945

C 15.309278 4.769967 5.790580

C 10.121669 13.967837 4.543254

H 14.802282 4.397952 6.671190

H 10.891639 14.433605 5.144192

C 12.092145 4.967631 7.020842

C 11.701185 13.195511 7.511637

C 11.135221 4.944260 8.090977

C 12.808935 13.273765 8.421704

C 10.637512 6.194745 8.657879

C 13.792354 12.195410 8.475285

C 10.974162 7.438255 8.036921

C 13.561072 10.988010 7.744562

C 12.412261 3.749123 6.364238

C 10.663120 14.159944 7.614867

H 13.039865 3.778580 5.483248

H 9.772206 14.036387 7.013279

C 11.903497 2.542420 6.797337

C 10.748010 15.217888 8.495836

H 12.157733 1.626507 6.272543

H 9.936743 15.936634 8.560601

C 11.035558 2.508630 7.902844

C 11.875874 15.345034 9.325505

H 10.634245 1.562296 8.252891

H 11.952498 16.177455 10.018570

C 10.651929 3.687183 8.517541

C 12.868980 14.382311 9.295370

H 9.930231 3.634577 9.323789

H 13.692624 14.472104 9.993355

C 9.780030 6.219628 9.781329

C 14.954494 12.279734 9.275505

H 9.538013 5.296710 10.295199

H 15.175244 13.196006 9.810414

C 10.399097 8.630000 8.536224

C 14.470679 9.914950 7.890922

H 10.646548 9.575005 8.073290

H 14.304119 8.997632 7.343762

C 9.242699 7.400259 10.259688

C 15.836766 11.221449 9.389822

C 9.546522 8.614538 9.623279

C 15.585254 10.024529 8.699943

H 8.589278 7.385448 11.126788

H 16.722077 11.319411 10.010843

H 9.125930 9.545558 9.991188

H 16.271779 9.187802 8.787260

**3**-TS_inv_

0 1

C 0.055912 -0.361237 -0.089450

C 1.096907 -1.305346 -0.454016

C 0.599568 -2.617888 -0.420918

C -0.894048 -2.504412 -0.126423

C -1.145111 -1.146827 0.129816

C -3.534696 -1.474772 0.529864

C -2.379831 -0.668978 0.699591

C 2.406385 -0.972430 -0.955714

C 3.393424 -1.990789 -0.935960

C 2.982945 -3.344570 -0.606208

C 1.581767 -3.692687 -0.538953

C 2.742154 0.293474 -1.492997

C 4.035400 0.587023 -1.874805

C 5.038351 -0.390361 -1.758817

C 4.713561 -1.660560 -1.323005

H 1.969571 1.037029 -1.628685

H 4.268261 1.565311 -2.283854

H 6.057437 -0.165405 -2.058675

H 5.484176 -2.421844 -1.328516

C 3.957398 -4.353109 -0.433194

H 5.002642 -4.071444 -0.395586

C 1.306714 -5.081402 -0.513616

H 0.298510 -5.418708 -0.617236

C 2.279580 -6.054750 -0.403092

H 1.987474 -7.100228 -0.377341

C 3.627984 -5.687624 -0.311924

H 4.402804 -6.436013 -0.177338

C -3.372054 -2.807737 -0.023876

C -2.056474 -3.387740 -0.171136

C -4.510760 -3.580842 -0.343778

H -5.487086 -3.112533 -0.316645

C -2.037192 -4.779564 -0.430398

H -1.108052 -5.305682 -0.401883

C -3.168716 -5.529053 -0.683247

C -4.427009 -4.914237 -0.688970

H -5.322787 -5.477489 -0.931624

H -3.070532 -6.591149 -0.886533

C -2.488491 0.531864 1.441234

H -1.596161 1.091489 1.683434

C -4.778414 -0.979106 0.987227

H -5.674100 -1.579021 0.880961

C -3.712307 0.988007 1.886877

H -3.769757 1.912168 2.453642

C -4.873915 0.240266 1.629706

H -5.839437 0.593628 1.978926

C 0.186336 1.029044 0.028286

C -0.743357 2.050398 -0.476069

C -0.276951 3.303965 -0.082503

C 1.067125 3.126521 0.505196

C 1.282920 1.760388 0.680073

C 3.348461 2.236340 1.934892

C 2.340652 1.298440 1.546450

C -1.856257 1.944608 -1.388874

C -2.663212 3.106439 -1.601720

C -2.372833 4.331095 -0.866582

C -1.149880 4.443833 -0.129147

C -2.132450 0.780956 -2.144923

C -3.188612 0.732187 -3.032620

C -4.005049 1.860265 -3.216285

C -3.732339 3.023912 -2.522625

H -1.488566 -0.081339 -2.045250

H -3.370787 -0.174093 -3.602171

H -4.829985 1.830032 -3.921685

H -4.340879 3.899248 -2.717930

C -3.273742 5.418878 -0.832772

H -4.214124 5.352646 -1.367119

C -0.929658 5.604919 0.656133

H -0.055919 5.651688 1.292867

C -1.836165 6.645834 0.669948

H -1.650707 7.516965 1.291020

C -3.011541 6.559354 -0.095732

H -3.730584 7.373022 -0.085351

C 3.306329 3.597253 1.414626

C 2.138524 4.057965 0.724584

C 4.396937 4.485808 1.545180

H 5.299298 4.156538 2.046764

C 2.154057 5.352228 0.143324

H 1.315144 5.666637 -0.463647

C 3.241004 6.191118 0.285836

C 4.366616 5.760154 1.008689

H 5.226493 6.413871 1.120270

H 3.233314 7.171190 -0.181496

C 2.379973 -0.004346 2.097056

H 1.586827 -0.701429 1.866623

C 4.367322 1.803894 2.813935

H 5.126467 2.504734 3.141338

C 3.393187 -0.396309 2.948893

H 3.391931 -1.401584 3.359181

C 4.404311 0.512003 3.303127

H 5.196909 0.211574 3.981770

**7a** (*θ* = 75°)

0 1

C 3.731790 2.237423 3.998078

C 3.911095 2.886137 2.796457

C 2.990862 2.721738 1.728409

C 1.853631 1.858764 1.917891

C 1.693962 1.215731 3.176058

C 2.609080 1.397034 4.188795

C 3.170119 3.387048 0.482239

C 2.271095 3.234256 -0.547301

C 1.161616 2.379033 -0.365802

C 0.963627 1.695548 0.828725

C 0.066106 2.057194 -1.280582

C -0.826441 1.203432 -0.635746

C -0.229037 0.766603 0.698312

C -0.160946 2.508895 -2.598721

C -1.306023 2.111929 -3.248407

C -2.286557 1.315565 -2.594200

C -2.064909 0.869999 -1.241880

C -3.503521 0.970914 -3.240238

C -4.480760 0.258663 -2.582914

C -4.289054 -0.125286 -1.234100

C -3.115078 0.174154 -0.582649

C 0.240254 -0.770583 0.697691

C 0.836473 -1.206614 -0.637120

C -0.056605 -2.060018 -1.281696

C -1.151307 -2.382408 -0.366140

C -0.952257 -1.699678 0.828654

C -2.260941 -3.237512 -0.547222

C -3.159075 -3.390953 0.482998

C -2.978729 -2.726434 1.729427

C -1.841324 -1.863596 1.918484

C 2.074446 -0.872837 -1.244061

C 2.294983 -1.317601 -2.596822

C 1.313899 -2.113560 -3.250691

C 0.169357 -2.510923 -2.600292

C 3.125171 -0.177426 -0.585246

C 4.298625 0.122356 -1.237466

C 4.489222 -0.260777 -2.586677

C 3.511434 -0.972590 -3.243647

C -3.898046 -2.891518 2.798165

C -3.717715 -2.243579 4.000045

C -2.594839 -1.403309 4.190334

C -1.680590 -1.221365 3.176933

H 4.446523 2.372990 4.804098

H 4.767471 3.539183 2.648539

H 0.826928 0.586138 3.339894

H 2.467059 0.897309 5.142495

H 4.037524 4.030361 0.360479

H 2.417293 3.750338 -1.491134

H 0.562621 3.152109 -3.090480

H -1.494720 2.429452 -4.270512

H -3.655919 1.295260 -4.266574

H -5.407839 0.007430 -3.089935

H -5.072885 -0.660232 -0.706553

H -2.989260 -0.114091 0.452226

H -2.407939 -3.753001 -1.491255

H -4.026578 -4.034201 0.361591

H 1.501729 -2.430459 -4.273149

H -0.554612 -3.153835 -3.091855

H 3.000191 0.110194 0.449908

H 5.082920 0.656940 -0.710242

H 5.415901 -0.009251 -3.094286

H 3.662981 -1.296318 -4.270304

H -4.754545 -3.544474 2.650557

H -4.431751 -2.379669 4.806593

H -2.452002 -0.904190 5.144229

H -0.813422 -0.591875 3.340436

H 0.944800 -0.886490 1.528005

H -0.932841 0.882074 1.529308

**7a** (*θ* = 180°)

0 1

C 3.994577 0.699173 -4.350579

C 4.754190 1.055658 -3.260681

C 4.173989 1.166475 -1.969170

C 2.772593 0.876621 -1.810277

C 2.012747 0.537112 -2.964349

C 2.608387 0.448179 -4.199742

C 4.945165 1.562653 -0.842234

C 4.367320 1.740018 0.395704

C 2.989425 1.472657 0.548163

C 2.226947 0.976585 -0.508782

C 2.087771 1.795879 1.661306

C 0.782255 1.494618 1.274655

C 0.823386 0.670001 -0.004600

C 2.357129 2.460292 2.877584

C 1.299097 2.869066 3.659457

C -0.046690 2.679142 3.241810

C -0.321893 1.986163 2.010041

C -1.136050 3.166617 4.011571

C -2.433753 3.003543 3.585716

C -2.702194 2.352666 2.356250

C -1.674772 1.859850 1.587854

C 0.671598 -0.909058 0.331212

C 0.713497 -1.733761 -0.947950

C -0.591768 -2.035187 -1.335299

C -1.494073 -1.711937 -0.222696

C -0.732222 -1.215734 0.834650

C -2.872045 -1.979337 -0.071016

C -3.450633 -1.801829 1.166554

C -2.680128 -1.405523 2.293901

C -1.278625 -1.115718 2.135818

C 1.818118 -2.225251 -1.682666

C 1.543676 -2.918510 -2.914442

C 0.198143 -3.108628 -3.332823

C -0.860375 -2.699798 -2.551639

C 3.170752 -2.098609 -1.259801

C 4.198650 -2.591517 -2.027506

C 3.930959 -3.242772 -3.256936

C 2.633511 -3.406072 -3.683476

C -3.261104 -1.294518 3.585050

C -2.502128 -0.937959 4.675365

C -1.115815 -0.687141 4.525356

C -0.519439 -0.776227 3.290332

H 4.452471 0.616446 -5.331758

H 5.815360 1.262694 -3.373712

H 0.955440 0.322859 -2.855258

H 2.014308 0.174612 -5.066339

H 6.004131 1.761976 -0.984458

H 4.953761 2.103286 1.234542

H 3.380407 2.666847 3.177183

H 1.481184 3.382367 4.600060

H -0.924251 3.677583 4.947402

H -3.256755 3.379844 4.185955

H -3.728912 2.234646 2.023572

H -1.891235 1.344671 0.658647

H -3.457982 -2.342719 -0.910158

H -4.509684 -2.001138 1.308165

H 0.016640 -3.622121 -4.273434

H -1.883463 -2.906480 -2.851800

H 3.386657 -1.583117 -0.330636

H 5.225174 -2.473300 -1.694293

H 4.754331 -3.619161 -3.856612

H 2.422280 -3.917290 -4.619298

H -4.322353 -1.501492 3.697462

H -2.960608 -0.855082 5.656258

H -0.522237 -0.413565 5.392292

H 0.537965 -0.562137 3.181870

H 0.033001 0.919721 -0.717466

H 1.461652 -1.158663 1.044489

**7a**-TS_rot_ (*θ* = 0°)

0 1

C 0.893259 3.253834 4.514079

C -0.358978 3.377358 3.957153

C -0.649274 2.834085 2.677385

C 0.379856 2.100296 1.986466

C 1.666181 2.010638 2.584282

C 1.920136 2.579458 3.809711

C -1.910620 3.034633 2.053743

C -2.144125 2.611063 0.763506

C -1.131276 1.902738 0.084707

C 0.068202 1.574736 0.712309

C -1.026614 1.533132 -1.333332

C 0.235467 0.985446 -1.550940

C 0.882703 0.673051 -0.207246

C -1.910838 1.784880 -2.402493

C -1.487263 1.536206 -3.689875

C -0.159844 1.104361 -3.957513

C 0.737988 0.837046 -2.863309

C 0.322887 0.967915 -5.286230

C 1.634965 0.636032 -5.534949

C 2.530836 0.427192 -4.458280

C 2.090550 0.515890 -3.159030

C 0.697587 -0.936813 0.198009

C -0.072497 -1.695409 -0.876086

C -1.408620 -1.795376 -0.495355

C -1.513524 -1.426476 0.922836

C -0.239958 -1.106404 1.387293

C -2.616111 -1.500774 1.798725

C -2.409440 -1.309521 3.147544

C -1.102850 -1.113534 3.671514

C 0.020372 -1.026567 2.773932

C 0.379063 -2.288939 -2.076386

C -0.612243 -2.840092 -2.964339

C -1.984793 -2.803658 -2.597036

C -2.382019 -2.324199 -1.367838

C 1.751132 -2.438420 -2.415629

C 2.130016 -3.063531 -3.579853

C 1.153969 -3.556888 -4.479485

C -0.183389 -3.447304 -4.174522

C -0.866816 -1.041524 5.070187

C 0.410940 -0.940359 5.570828

C 1.518614 -0.909942 4.689076

C 1.326349 -0.942539 3.328303

H 1.102334 3.684735 5.488657

H -1.144243 3.913109 4.484385

H 2.456785 1.490665 2.053375

H 2.911182 2.503938 4.247364

H -2.678422 3.576229 2.599798

H -3.085763 2.831546 0.269419

H -2.903225 2.182945 -2.212435

H -2.153123 1.715620 -4.529796

H -0.363034 1.150724 -6.109482

H 1.991329 0.546886 -6.556833

H 3.569577 0.185494 -4.663252

H 2.781649 0.348838 -2.339416

H -3.608139 -1.720976 1.415578

H -3.244573 -1.355124 3.841447

H -2.717495 -3.209583 -3.289422

H -3.424648 -2.366132 -1.066882

H 2.504875 -2.058041 -1.734111

H 3.183889 -3.172052 -3.818572

H 1.465937 -4.034081 -5.403688

H -0.935533 -3.844077 -4.851646

H -1.718723 -1.087250 5.743840

H 0.575330 -0.897601 6.643366

H 2.524946 -0.850207 5.092971

H 2.179793 -0.912905 2.658952

H 1.954819 0.881739 -0.182252

H 1.700160 -1.333044 0.375450

**7a**-TS_roc_ (*θ* = 120°)

0 1

C 5.775862 1.964329 -0.731040

C 5.328115 2.531035 0.441124

C 3.968673 2.419544 0.836402

C 3.067964 1.642255 0.021148

C 3.557863 1.107882 -1.201406

C 4.874279 1.265623 -1.569215

C 3.472253 3.105879 1.978509

C 2.127254 3.115094 2.271983

C 1.247035 2.355492 1.470645

C 1.716038 1.548014 0.435117

C -0.218450 2.351165 1.439246

C -0.638514 1.505314 0.418970

C 0.555339 0.737059 -0.136852

C -1.138401 3.102457 2.201811

C -2.479425 2.999655 1.912949

C -2.944056 2.193416 0.836798

C -2.003656 1.437593 0.052421

C -4.322830 2.124774 0.505288

C -4.759874 1.367672 -0.557644

C -3.830011 0.649008 -1.347649

C -2.487474 0.683475 -1.050632

C 0.593971 -0.820864 0.392452

C 0.846419 -1.793370 -0.757547

C -0.353239 -2.410544 -1.110027

C -1.348155 -2.124820 -0.072175

C -0.768819 -1.296968 0.882773

C -2.661084 -2.620535 0.078602

C -3.372206 -2.282587 1.206645

C -2.792694 -1.489449 2.235754

C -1.449478 -0.995232 2.086162

C 2.053763 -2.179857 -1.390774

C 1.963553 -3.050483 -2.537066

C 0.694406 -3.535546 -2.956448

C -0.444611 -3.260753 -2.233723

C 3.344815 -1.848111 -0.897512

C 4.483185 -2.286572 -1.533917

C 4.392865 -3.079017 -2.703294

C 3.159767 -3.454252 -3.187284

C -3.506991 -1.181016 3.423307

C -2.926945 -0.440261 4.427732

C -1.594350 0.019204 4.292414

C -0.874667 -0.251592 3.152013

H 6.815248 2.071242 -1.026992

H 6.007438 3.098836 1.071797

H 2.876005 0.573314 -1.851618

H 5.223439 0.845516 -2.507492

H 4.170580 3.675109 2.586286

H 1.743093 3.710052 3.095348

H -0.791222 3.746561 3.004196

H -3.209467 3.554926 2.495652

H -5.031066 2.688271 1.107287

H -5.817769 1.325681 -0.799533

H -4.181786 0.063389 -2.191869

H -1.782597 0.123299 -1.655052

H -3.100955 -3.254178 -0.685753

H -4.390660 -2.637627 1.339039

H 0.647802 -4.179714 -3.830490

H -1.397203 -3.703218 -2.509910

H 3.425711 -1.244068 -0.001945

H 5.457318 -2.017075 -1.137436

H 5.298929 -3.408459 -3.203149

H 3.084288 -4.091716 -4.064535

H -4.525279 -1.546759 3.527457

H -3.485741 -0.213039 5.330799

H -1.139904 0.593099 5.094614

H 0.142418 0.111150 3.052248

H 0.553790 0.702507 -1.229124

H 1.356826 -0.873161 1.173227

***ω*B97XD/6-311++G**//6-31G****

*S_0_*-**1a**

0 1

C 21.447755 0.357943 12.437787

C 21.594916 0.404867 13.821838

C 21.972864 1.348777 11.591246

H 21.857861 1.271241 10.514713

C 22.640547 2.405518 12.150690

H 23.084566 3.171114 11.520831

C 22.723083 2.556651 13.560368

C 22.143489 1.579830 14.429354

C 23.345254 3.704570 14.116546

H 23.799403 4.424410 13.441165

C 23.356021 3.918052 15.468729

H 23.830173 4.802242 15.882058

C 22.712018 2.996848 16.322401

H 22.672988 3.188537 17.389866

C 22.121903 1.866503 15.818572

H 21.616157 1.192461 16.494772

C 20.677930 -0.830582 12.081642

C 20.901082 -0.771479 14.395264

C 20.313969 -1.289054 10.804379

C 20.337188 -1.515784 13.245274

C 20.800733 -1.107028 15.733450

C 19.611034 -2.459840 10.701438

H 20.578334 -0.719874 9.918769

C 19.748969 -2.817607 13.145660

C 19.575540 -1.512149 16.460717

C 21.896700 -1.134314 16.729645

H 19.288393 -2.824055 9.730238

C 19.333717 -3.251145 11.847613

C 19.581825 -3.722768 14.225150

C 18.191697 -1.475417 16.094559

C 19.924028 -1.756417 17.786756

C 21.357146 -1.530165 17.951040

C 23.318690 -1.042610 16.588009

C 18.678972 -4.502277 11.705523

C 18.965573 -4.934939 14.050274

H 19.961937 -3.463347 15.202632

C 17.687186 -0.999270 14.857100

C 17.244231 -1.918081 17.070019

C 18.980031 -2.139078 18.754503

C 22.141616 -1.680944 19.106874

C 24.008881 -0.897811 15.357050

C 24.103325 -1.144048 17.779325

H 18.351236 -4.804289 10.714580

C 18.482622 -5.324213 12.782496

H 18.858471 -5.605937 14.896476

C 16.347261 -1.041862 14.569834

H 18.366974 -0.577825 14.130681

C 15.866873 -1.970593 16.731944

C 17.668068 -2.261439 18.381229

H 19.288535 -2.341844 19.775230

H 21.685701 -1.971463 20.048098

C 23.487730 -1.443347 19.023622

H 23.453445 -0.904171 14.430310

C 25.373449 -0.771359 15.315486

C 25.511710 -0.986406 17.703401

H 17.985424 -6.280974 12.660603

C 15.423596 -1.556934 15.504550

H 15.993920 -0.664742 13.615558

H 15.164835 -2.330784 17.478929

H 16.920048 -2.585756 19.099048

H 24.115621 -1.521096 19.906637

H 25.870559 -0.667055 14.356451

C 26.137582 -0.788882 16.502020

H 26.088418 -1.044572 18.622419

H 14.367294 -1.596720 15.259394

H 27.215702 -0.674913 16.455842

*T_1_*-**1a**

0 3

C 21.205529 0.459229 12.430659

C 21.188588 0.549072 13.833460

C 21.457073 1.578077 11.627991

H 21.466988 1.492451 10.545696

C 21.691998 2.789879 12.235596

H 21.889682 3.671628 11.632664

C 21.684558 2.929690 13.651833

C 21.429648 1.798588 14.485717

C 21.929095 4.189927 14.253167

H 22.122059 5.044098 13.609899

C 21.923600 4.334943 15.616648

H 22.112023 5.305040 16.064877

C 21.671479 3.216969 16.439053

H 21.667350 3.333406 17.518055

C 21.430814 1.981180 15.890117

H 21.239474 1.136952 16.540665

C 20.928011 -0.928983 12.062862

C 20.905481 -0.761729 14.357411

C 20.833416 -1.532853 10.803566

C 20.747053 -1.664574 13.247049

C 20.797534 -1.115026 15.771666

C 20.556155 -2.878580 10.733517

H 20.975685 -0.950943 9.898058

C 20.458856 -3.064334 13.197398

C 19.592560 -1.087986 16.559370

C 21.877498 -1.551409 16.618010

H 20.477704 -3.368354 9.767049

C 20.365301 -3.664240 11.904690

C 20.262804 -3.882110 14.336897

C 18.248114 -0.735797 16.222814

C 19.919444 -1.498665 17.863398

C 21.352589 -1.788818 17.900341

C 23.268017 -1.752968 16.352091

C 20.080518 -5.049354 11.805002

C 19.988433 -5.221278 14.203528

H 20.329839 -3.447633 15.326473

C 17.829634 -0.311553 14.938068

C 17.272653 -0.822622 17.262357

C 18.950920 -1.577876 18.870921

C 22.163207 -2.226711 18.954156

C 23.881270 -1.539604 15.093539

C 24.080549 -2.199591 17.438378

H 20.011224 -5.495926 10.816848

C 19.895266 -5.814955 12.927354

H 19.841786 -5.828609 15.090931

C 16.516275 0.008947 14.695710

H 18.553054 -0.238216 14.135692

C 15.926169 -0.484019 16.976043

C 17.651083 -1.244007 18.567957

H 19.219261 -1.897052 19.873380

H 21.742736 -2.405551 19.939035

C 23.504076 -2.426520 18.719588

H 23.283538 -1.201299 14.256429

C 25.226382 -1.756512 14.920042

C 25.465323 -2.413011 17.222875

H 19.677714 -6.874286 12.837799

C 15.551735 -0.076723 15.721428

H 16.218357 0.331690 13.703281

H 15.191784 -0.553665 17.773848

H 16.882698 -1.298390 19.333920

H 24.152243 -2.765973 19.522541

H 25.674824 -1.586483 13.946491

C 26.030128 -2.197370 15.992154

H 26.074711 -2.752641 18.055911

H 14.517774 0.179696 15.514820

H 27.091462 -2.364363 15.839888

**1a**-TS_rot_ (OS, S^2^ = 1.110305)

0 1

C 21.203407 0.459918 12.430087

C 21.185390 0.550223 13.832602

C 21.452147 1.579215 11.627165

H 21.462936 1.493240 10.544906

C 21.683081 2.791977 12.234476

H 21.878412 3.674117 11.631348

C 21.674570 2.932160 13.650635

C 21.422712 1.800424 14.484605

C 21.915077 4.193188 14.251977

H 22.105684 5.047906 13.608729

C 21.908704 4.338234 15.615456

H 22.094027 5.308936 16.063670

C 21.659881 3.219540 16.437949

H 21.655247 3.336057 17.516940

C 21.423120 1.983008 15.889032

H 21.234371 1.138050 16.539433

C 20.930068 -0.929411 12.062366

C 20.905492 -0.761757 14.357650

C 20.838173 -1.533668 10.803053

C 20.750360 -1.665301 13.246276

C 20.797585 -1.114897 15.771461

C 20.564945 -2.880253 10.732906

H 20.979460 -0.951434 9.897599

C 20.465955 -3.065704 13.196653

C 19.592888 -1.084882 16.561035

C 21.877086 -1.554825 16.618020

H 20.488758 -3.370374 9.766432

C 20.375324 -3.666242 11.904013

C 20.270823 -3.883553 14.336297

C 18.249268 -0.729105 16.225534

C 19.919673 -1.496737 17.864459

C 21.352255 -1.791052 17.900337

C 23.266773 -1.760021 16.351262

C 20.094590 -5.052218 11.804552

C 20.000392 -5.223525 14.203131

H 20.335408 -3.448299 15.325718

C 17.831127 -0.304009 14.940927

C 17.274327 -0.813096 17.265813

C 18.951773 -1.573435 18.872782

C 22.162160 -2.231694 18.953559

C 23.879785 -1.547283 15.092452

C 24.078715 -2.209660 17.436765

H 20.027538 -5.499355 10.816497

C 19.910380 -5.817904 12.927019

H 19.854396 -5.830962 15.090569

C 16.518556 0.020304 14.699464

H 18.554296 -0.233189 14.138066

C 15.928633 -0.470544 16.980400

C 17.652615 -1.235693 18.571024

H 19.220049 -1.893607 19.874941

H 21.741704 -2.409634 19.938608

C 23.502322 -2.435496 18.718165

H 23.282348 -1.206372 14.256159

C 25.224126 -1.767967 14.917875

C 25.462722 -2.426978 17.220125

H 19.695955 -6.877886 12.837620

C 15.554554 -0.062292 15.725986

H 16.220801 0.343652 13.707184

H 15.194586 -0.537962 17.778710

H 16.884738 -1.287950 19.337647

H 24.149909 -2.777259 19.520606

H 25.672469 -1.598393 13.944200

C 26.027257 -2.212097 15.989143

H 26.071723 -2.768943 18.052489

H 14.521204 0.197154 15.520090

H 27.087993 -2.382088 15.836023

CS-**1b**

0 1

C 9.139898 21.055184 10.059635

C 9.800857 22.184326 9.569300

H 9.650683 22.483837 8.535667

C 8.196569 20.270148 9.185575

H 7.177206 20.664847 9.261200

H 8.163813 19.217621 9.479178

H 8.491498 20.321428 8.134160

C 9.357497 20.689348 11.390466

C 10.647527 22.929368 10.377436

C 10.201308 21.425608 12.209437

H 8.858104 19.811564 11.791370

C 10.859130 22.559129 11.712537

H 11.155398 23.802412 9.981692

H 10.361181 21.127593 13.240317

C 11.731952 23.319423 12.549762

C 12.471965 23.964087 13.260473

C 13.349486 24.732188 14.081491

C 13.580725 24.362000 15.450106

C 13.948852 25.863809 13.561349

C 12.902930 23.254472 16.018431

C 14.465812 25.134098 16.264870

H 13.719853 26.176051 12.548079

C 14.858599 26.578877 14.344416

H 12.253527 22.666089 15.379052

C 13.041550 22.945076 17.345330

C 14.531262 24.820827 17.646541

C 15.198780 26.192270 15.638620

C 15.588902 27.798452 14.008499

H 12.512209 22.096582 17.766337

C 13.844635 23.757415 18.171764

H 15.119940 25.443329 18.304595

C 16.173069 27.169062 16.167104

C 15.561141 28.529765 12.818434

C 16.381585 28.163664 15.094511

H 13.913574 23.547933 19.234248

C 16.773866 27.149144 17.417827

H 14.917346 28.233206 11.997321

C 16.351978 29.656634 12.696324

C 17.336333 29.218657 14.935756

C 16.905063 28.279053 18.360574

C 17.405986 25.987888 18.077043

C 16.274977 30.440257 11.507170

C 17.269606 30.006354 13.744680

C 18.365901 29.515459 15.864817

C 16.327026 29.588730 18.375038

C 17.565847 27.817625 19.497040

C 17.761140 24.687611 17.595130

C 17.874463 26.400608 19.322274

C 16.194021 31.096407 10.491682

C 18.146158 31.109681 13.592085

C 19.214108 30.575594 15.677791

H 18.497469 28.883192 16.730830

C 15.422123 30.076457 17.398067

C 16.656563 30.452696 19.465109

C 17.841045 28.640314 20.592200

C 18.377747 23.781107 18.512326

C 17.574039 24.248218 16.259756

C 18.531502 25.536307 20.201806

C 16.095292 31.869047 9.294143

H 18.065629 31.709254 12.691638

C 19.088380 31.401546 14.542142

H 19.993733 30.772061 16.406559

C 14.947017 31.361206 17.441410

H 15.084634 29.417417 16.611431

C 16.167417 31.783047 19.468525

C 17.440207 29.962784 20.564907

H 18.376259 28.258539 21.455016

C 18.665969 22.458326 18.092529

C 18.738912 24.220935 19.831366

H 17.194472 24.939860 15.521783

C 17.885233 22.969445 15.877598

H 18.865669 25.879872 21.174910

C 15.203563 31.499043 8.277971

C 16.890236 33.008616 9.110627

H 19.755207 32.247001 14.408251

H 14.252503 31.700997 16.680033

C 15.343826 32.236759 18.472747

H 16.446510 32.431289 20.292296

C 17.776448 30.818915 21.654877

C 18.411983 22.051944 16.809564

H 19.112184 21.777210 18.809198

C 19.348704 23.320304 20.753942

H 17.733282 22.666994 14.846609

H 14.586870 30.616193 8.408426

C 15.113891 32.251552 7.115925

C 16.790355 33.752500 7.943667

H 17.587141 33.301548 9.888820

H 14.974118 33.256716 18.492405

C 18.071713 31.534033 22.587495

H 18.642678 21.037093 16.502579

C 19.863672 22.563418 21.547795

H 14.419631 31.948472 6.336943

C 15.901275 33.390595 6.928447

H 17.416525 34.631379 7.816795

C 18.419074 32.376660 23.687545

C 20.470173 21.668561 22.481743

C 15.774345 34.221219 5.677808

C 17.998546 33.713059 23.724242

C 19.190265 31.883811 24.749200

C 20.736346 20.339206 22.126255

C 20.806041 22.100823 23.772003

H 15.496451 33.606388 4.817614

H 15.001115 34.988295 5.797434

H 16.711194 34.732903 5.442258

H 17.404688 34.106112 22.905783

C 18.341376 34.526737 24.794574

H 19.524666 30.852067 24.728138

C 19.525845 32.708114 25.813413

H 20.476608 19.992900 21.131419

C 21.322129 19.473321 23.038392

C 21.391576 21.224610 24.674364

H 20.599939 23.126522 24.058722

H 18.009300 35.561250 24.805266

C 19.106820 34.040458 25.857374

H 20.126918 32.310512 26.626596

H 21.518515 18.445185 22.746875

C 21.662170 19.899039 24.324948

H 21.642545 21.575562 25.671621

C 19.447649 34.919580 27.032582

C 22.327989 18.962672 25.299646

H 18.690469 34.827645 27.819231

H 19.494344 35.972524 26.742652

H 20.410749 34.643783 27.470346

H 22.039566 17.924440 25.115506

H 23.418627 19.021905 25.212474

H 22.067233 19.210636 26.331959

OS-**1b** (≡ *S_0_*-**1b**, S^2^ = 0.580542)

0 1

C 9.015853 20.676149 10.454381

C 9.184230 22.041810 10.211725

H 8.627016 22.513694 9.407062

C 8.047348 19.858894 9.639418

H 7.051773 19.865947 10.097003

H 8.368042 18.816316 9.566911

H 7.945749 20.255346 8.625719

C 9.750737 20.094602 11.490819

C 10.051716 22.806543 10.978225

C 10.621488 20.847696 12.265024

H 9.639541 19.032945 11.693649

C 10.783639 22.218029 12.018728

H 10.173405 23.865426 10.776481

H 11.186204 20.380994 13.065242

C 11.680226 22.999286 12.809667

C 12.440021 23.663993 13.480682

C 13.329040 24.462951 14.255990

C 14.096421 23.879194 15.322785

C 13.426740 25.819016 13.990691

C 13.954798 22.506019 15.640526

C 14.987515 24.686993 16.094322

H 12.808646 26.259398 13.215500

C 14.330028 26.595115 14.714842

H 13.286539 21.903745 15.034318

C 14.628845 21.950474 16.696538

C 15.629752 24.087741 17.206302

C 15.154267 26.056771 15.708765

C 14.596758 28.028291 14.615271

H 14.506969 20.897477 16.928389

C 15.459286 22.758375 17.498289

H 16.256020 24.691575 17.847277

C 15.964329 27.142360 16.258391

C 14.012882 28.973706 13.773665

C 15.587827 28.361097 15.543988

H 15.963601 22.330499 18.358596

C 16.926786 27.041174 17.297466

H 13.234427 28.692068 13.072524

C 14.421469 30.295208 13.848229

C 16.125266 29.688952 15.553865

C 16.952034 27.824360 18.531696

C 18.083872 26.148554 17.333140

C 13.785565 31.268999 13.025256

C 15.495768 30.670121 14.727525

C 17.256635 30.086485 16.309185

C 16.019404 28.747264 19.106591

C 18.071082 27.428632 19.270968

C 18.646862 25.259246 16.360983

C 18.772711 26.384402 18.527654

C 13.232204 32.086411 12.321699

C 15.962572 32.007173 14.761360

C 17.695047 31.386118 16.307870

H 17.795668 29.349590 16.887223

C 14.761640 29.068961 18.538205

C 16.368476 29.363165 20.347728

C 18.389863 27.993379 20.504922

C 19.811049 24.516992 16.729229

C 18.138771 25.086372 15.049460

C 19.934108 25.694222 18.870432

C 12.579509 33.050758 11.494731

H 15.462373 32.743005 14.140789

C 17.030071 32.364761 15.542773

H 18.565975 31.657704 16.895468

C 13.932259 29.995012 19.117688

H 14.440537 28.565306 17.637697

C 15.497363 30.327393 20.911836

C 17.575520 28.982954 21.030728

H 19.272981 27.679538 21.051291

C 20.357442 23.585199 15.812795

C 20.437959 24.737728 18.004381

H 17.300055 25.683518 14.721308

C 18.700373 24.187370 14.178858

H 20.441354 25.887158 19.809786

C 11.527360 32.672315 10.649610

C 12.981363 34.393636 11.510148

H 17.376995 33.392819 15.555086

H 12.974537 30.217294 18.658445

C 14.310500 30.649142 20.306939

H 15.785699 30.797685 21.845887

C 17.932124 29.597001 22.265926

C 19.810249 23.411760 14.568368

H 21.231910 23.018675 16.114741

C 21.597333 23.999374 18.379214

H 18.288567 24.080463 13.180584

H 11.211489 31.634786 10.626993

C 10.899293 33.614181 9.847394

C 12.344697 35.325150 10.703043

H 13.797896 34.696599 12.157109

H 13.653165 31.387859 20.753725

C 18.249243 30.114451 23.315216

H 20.242694 22.695847 13.877109

C 22.583160 23.378342 18.713603

H 10.087317 33.302836 9.195945

C 11.293012 34.954663 9.860796

H 12.671198 36.361270 10.725266

C 18.618463 30.727615 24.551176

C 23.745017 22.646992 19.107381

C 10.585005 35.978340 9.011748

C 17.820331 31.733213 25.114312

C 19.786567 30.340278 25.221752

C 24.296917 21.673803 18.262850

C 24.352941 22.885738 20.347570

H 10.173375 35.528124 8.104625

H 9.752453 36.430952 9.561856

H 11.259897 36.785667 8.715240

H 16.916259 32.043677 24.601180

C 18.184967 32.328728 26.312874

H 20.413535 29.566169 24.792334

C 20.139502 30.944034 26.420241

H 23.831748 21.477955 17.302471

C 25.424042 20.965072 18.652656

C 25.479959 22.169945 20.724933

H 23.931009 23.633587 21.010466

H 17.556366 33.108878 26.733424

C 19.346757 31.944664 26.987882

H 21.050096 30.633705 26.925299

H 25.836785 20.212107 17.986838

C 26.036619 21.200915 19.886214

H 25.936761 22.365613 21.691197

C 19.718279 32.573439 28.305731

C 27.279840 20.450827 20.288503

H 19.248949 32.037378 29.138175

H 19.387602 33.614207 28.357774

H 20.799093 32.550984 28.467754

H 27.301643 19.450323 19.848491

H 28.178626 20.978620 19.950537

H 27.348791 20.346882 21.374550

*T_1_*-**1b**

0 3

C 7.132409 22.472840 11.657511

C 7.833039 23.512692 11.040826

H 7.500790 23.880205 10.073798

C 5.904569 21.880059 11.016397

H 5.003254 22.416633 11.333148

H 5.776838 20.830623 11.294669

H 5.956095 21.939511 9.926039

C 7.586260 22.015424 12.897435

C 8.946738 24.083574 11.639651

C 8.698259 22.577290 13.507403

H 7.059915 21.203902 13.392394

C 9.395282 23.622473 12.885131

H 9.481525 24.888964 11.147626

H 9.039671 22.209655 14.469320

C 10.542355 24.201581 13.507401

C 11.516067 24.690619 14.039990

C 12.660409 25.272867 14.652417

C 13.109710 24.813979 15.941941

C 13.339336 26.289802 13.989431

C 12.423871 23.779292 16.622050

C 14.258846 25.401772 16.551023

H 12.985992 26.621815 13.018714

C 14.458715 26.862941 14.580426

H 11.551687 23.339926 16.149670

C 12.847535 23.338141 17.850162

C 14.668969 24.923584 17.818784

C 14.931614 26.445215 15.838738

C 15.338664 27.928919 14.100594

H 12.310313 22.543787 18.357939

C 13.981280 23.917655 18.452134

H 15.537818 25.356728 18.296965

C 16.091536 27.229147 16.157559

C 15.298483 28.662599 12.921339

C 16.331617 28.141615 15.075448

H 14.315892 23.568352 19.423539

C 16.892845 27.114306 17.377233

H 14.527342 28.487826 12.178260

C 16.262955 29.637490 12.688621

C 17.340192 29.136447 14.871446

C 16.674550 27.837773 18.597928

C 18.024823 26.250335 17.560233

C 16.213322 30.382835 11.478011

C 17.297143 29.888766 13.659278

C 18.371742 29.413116 15.800656

C 15.696737 28.816044 18.965948

C 17.652319 27.429566 19.524638

C 18.668734 25.321395 16.682130

C 18.500678 26.431385 18.872631

C 16.154872 31.007902 10.440300

C 18.281507 30.879322 13.428774

C 19.311764 30.381535 15.546554

H 18.420572 28.853114 16.725632

C 14.682632 29.281108 18.094538

C 15.753598 29.348795 20.288782

C 17.706040 27.949719 20.812035

C 19.791106 24.597843 17.185929

C 18.255660 25.082825 15.349261

C 19.593803 25.726190 19.361015

C 16.082554 31.741780 9.217803

H 18.238741 31.444556 12.503730

C 19.268625 31.123101 14.349893

H 20.092763 30.575022 16.274737

C 13.771817 30.222366 18.507358

H 14.623808 28.889513 17.087268

C 14.799448 30.317539 20.681967

C 16.771002 28.901474 21.205277

H 18.464698 27.625030 21.516592

C 20.447863 23.671167 16.341326

C 20.243091 24.813175 18.536650

H 17.407477 25.622809 14.948806

C 18.915181 24.176798 14.555477

H 19.948983 25.874657 20.375508

C 15.076428 31.468679 8.280570

C 17.017620 32.745518 8.928994

H 20.015765 31.886197 14.157760

H 13.003778 30.562966 17.820518

C 13.828449 30.747165 19.813135

H 14.852297 30.715352 21.689880

C 16.831631 29.426826 22.525842

C 20.022029 23.462524 15.053911

H 21.299446 23.126560 16.735215

C 21.359299 24.093321 19.046028

H 18.579256 24.011725 13.536875

H 14.350618 30.690833 8.492469

C 15.012500 32.182559 7.092762

C 16.942009 33.451775 7.737343

H 17.803114 32.962143 9.645315

H 13.104577 31.490540 20.130671

C 16.893517 29.865523 23.654859

H 20.536875 22.748407 14.419540

C 22.308159 23.485364 19.494380

H 14.227946 31.955637 6.376095

C 15.939636 33.186286 6.800615

H 17.676923 34.224324 7.528348

C 16.962720 30.382609 24.983823

C 23.424922 22.767986 20.020227

C 15.844532 33.979944 5.523496

C 16.038102 31.338989 25.426433

C 17.957738 29.947192 25.870035

C 24.121782 21.848376 19.223889

C 23.842718 22.965188 21.343710

H 15.399019 33.388811 4.719050

H 15.219399 34.868820 5.663961

H 16.828961 34.320018 5.191323

H 15.266549 31.686256 24.747347

C 16.111279 31.839536 26.718106

H 18.680799 29.210743 25.536060

C 18.019436 30.456176 27.159206

H 23.804299 21.684808 18.199438

C 25.203536 21.151166 19.741622

C 24.926228 22.261112 21.848828

H 23.307557 23.671185 21.969745

H 15.388323 32.582506 27.043531

C 17.098737 31.406979 27.607192

H 18.799139 30.109829 27.832067

H 25.730148 20.439843 19.111297

C 25.626541 21.345484 21.059202

H 25.234425 22.423793 22.877993

C 17.151624 31.930583 29.018935

C 26.822260 20.608538 21.604320

H 16.540357 31.312379 29.685853

H 16.771091 32.953657 29.079090

H 18.172928 31.923554 29.408715

H 26.934945 19.628355 21.133516

H 27.743819 21.171121 21.417673

H 26.740809 20.460306 22.684338

**1b**-TS_rot_ (OS, S^2^ = 1.171715)

0 1

C 7.120139 22.485706 11.665066

C 7.822520 23.523657 11.047078

H 7.490403 23.891043 10.079951

C 5.890719 21.895111 11.024960

H 4.991591 22.439082 11.335250

H 5.756342 20.848465 11.310445

H 5.945529 21.946387 9.934328

C 7.573825 22.028393 12.905040

C 8.937720 24.092747 11.644725

C 8.687376 22.588513 13.513871

H 7.046153 21.218354 13.401007

C 9.386115 23.631743 12.890331

H 9.473843 24.896649 11.151721

H 9.028628 22.220956 14.475874

C 10.534756 24.209051 13.511407

C 11.509765 24.696607 14.042967

C 12.655626 25.276943 14.654425

C 13.104393 24.818242 15.944143

C 13.336586 26.291789 13.990279

C 12.416751 23.785580 16.625550

C 14.255038 25.404163 16.552190

H 12.983687 26.623648 13.019350

C 14.457309 26.863159 14.580492

H 11.543386 23.347617 16.154044

C 12.840226 23.344617 17.853792

C 14.665073 24.926055 17.820047

C 14.929475 26.445667 15.838916

C 15.339299 27.927165 14.099354

H 12.301612 22.551840 18.362560

C 13.975644 23.922120 18.454624

H 15.535299 25.357709 18.297118

C 16.091547 27.227954 16.157553

C 15.300215 28.659566 12.919240

C 16.332707 28.139184 15.073574

H 14.310125 23.572781 19.426061

C 16.892391 27.113143 17.376834

H 14.528610 28.485339 12.176517

C 16.266473 29.632393 12.685169

C 17.342853 29.131994 14.868458

C 16.672984 27.835122 18.599152

C 18.026589 26.250766 17.559916

C 16.218119 30.376413 11.473662

C 17.301182 29.882946 13.655387

C 18.374723 29.407988 15.797567

C 15.693571 28.811312 18.967919

C 17.651224 27.427649 19.525354

C 18.672288 25.323889 16.681165

C 18.501675 26.431552 18.872397

C 16.160795 31.000434 10.435265

C 18.287424 30.871405 13.423771

C 19.316530 30.374356 15.542379

H 18.422222 28.849088 16.723302

C 14.679127 29.275728 18.096502

C 15.749053 29.342635 20.291395

C 17.703839 27.946448 20.813365

C 19.796255 24.602319 17.184273

C 18.259338 25.085427 15.348203

C 19.596201 25.728182 19.360333

C 16.089897 31.733207 9.212009

H 18.245791 31.435616 12.498053

C 19.274888 31.114447 14.344711

H 20.097762 30.567417 16.270423

C 13.766514 30.214903 18.510028

H 14.621650 28.885261 17.088694

C 14.793013 30.309250 20.685305

C 16.766991 28.896053 21.207576

H 18.462976 27.622326 21.517662

C 20.454940 23.677834 16.338737

C 20.247658 24.817401 18.535172

H 17.409696 25.623762 14.948561

C 18.920725 24.181555 14.553547

H 19.950897 25.876456 20.375021

C 15.083681 31.460783 8.274790

C 17.026636 32.735283 8.922509

H 20.023463 31.875920 14.151715

H 12.998227 30.555089 17.823263

C 13.821652 30.738161 19.816526

H 14.844724 30.706009 21.693692

C 16.826384 29.419997 22.528785

C 20.029339 23.469416 15.051210

H 21.307757 23.134716 16.732013

C 21.365481 24.099587 19.043959

H 18.584947 24.016462 13.534902

H 14.356595 30.684266 8.487185

C 15.021185 32.173683 7.086223

C 16.952460 33.440490 7.730236

H 17.812235 32.951367 9.638879

H 13.096336 31.479887 20.134624

C 16.887219 29.857551 23.658295

H 20.545675 22.757007 14.416128

C 22.315726 23.493439 19.491812

H 14.236465 31.947286 6.369584

C 15.949891 33.175642 6.793411

H 17.688613 34.211722 7.520692

C 16.955189 30.373246 24.987875

C 23.434162 22.778252 20.017116

C 15.856946 33.968544 5.515661

C 16.028773 31.327479 25.431359

C 17.950784 29.938608 25.873812

C 24.134927 21.862915 19.219182

C 23.849764 22.973350 21.341535

H 15.395548 33.383339 4.715899

H 15.247496 34.867680 5.659692

H 16.844004 34.292284 5.174935

H 15.256749 31.674136 24.752495

C 16.100782 31.826710 26.723605

H 18.675227 29.203823 25.539169

C 18.011302 30.446259 27.163571

H 23.819163 21.701023 18.193933

C 25.218273 21.167886 19.736353

C 24.934997 22.271421 21.846127

H 23.311620 23.675988 21.968788

H 15.376438 32.568032 27.049704

C 17.088822 31.394919 27.612421

H 18.791478 30.100545 27.836207

H 25.747935 20.459894 19.104820

C 25.639138 21.360149 21.054993

H 25.241456 22.432452 22.876062

C 17.140458 31.917069 29.024750

C 26.836613 20.625340 21.599143

H 16.529295 31.297674 29.690656

H 16.759052 32.939770 29.085759

H 18.161537 31.910467 29.415122

H 26.944397 19.640989 21.135863

H 27.758202 21.184280 21.402017

H 26.761430 20.486308 22.680804

*S_0_*-**1c**

0 1

C 11.301557 7.494313 8.763930

C 11.455016 7.176188 10.200459

C 12.439614 6.074073 10.267113

C 13.155358 5.483531 11.360811

C 13.033018 5.885694 12.714262

H 12.398072 6.724318 12.960829

H 13.599447 5.582404 14.741840

C 12.837894 5.770412 8.971721

C 12.128078 6.643696 8.041332

C 10.355667 8.301778 8.049486

C 9.290964 9.017704 8.651395

H 9.129854 8.928651 9.715966

C 8.449370 9.807966 7.910167

H 7.643150 10.339855 8.405368

C 8.615814 9.925618 6.516705

H 7.953444 10.563525 5.940578

C 9.597779 9.203755 5.888466

H 9.715715 9.249159 4.812635

C 10.466036 8.364888 6.628027

C 11.422692 7.543132 5.944688

C 12.208588 6.654226 6.638191

H 12.883958 5.976208 6.130914

C 12.349754 6.902885 3.859684

H 12.224100 7.193820 2.816898

H 12.129649 5.833336 3.965490

H 13.386325 7.089272 4.166688

C 10.152539 7.117604 12.408004

C 9.630446 8.099551 13.239873

C 9.937679 9.410592 12.675306

C 9.623671 10.672618 13.208296

H 9.060944 10.744269 14.131000

C 10.039357 11.791824 12.527711

C 10.864486 11.676288 11.360097

C 11.363347 12.841095 10.728549

H 11.059807 13.807241 11.113015

C 12.228047 12.748998 9.668432

H 12.607173 13.647800 9.193026

C 12.648663 11.481331 9.221915

H 13.370109 11.404221 8.414643

C 12.163651 10.337628 9.804103

H 12.517613 9.377976 9.456362

C 11.222934 10.387435 10.862913

C 10.638266 9.236885 11.488782

C 10.810575 7.784913 11.263340

C 8.910805 7.814430 14.413000

H 8.541110 8.624231 15.030004

C 8.703252 6.498197 14.749282

C 9.117402 5.442395 13.871061

C 8.809177 4.097269 14.187492

H 8.310784 3.888579 15.126347

C 9.114595 3.083630 13.316129

C 9.714398 3.387089 12.078635

H 9.914012 2.593681 11.365343

C 10.041429 4.680247 11.757662

H 10.482292 4.888993 10.793686

C 9.797391 5.750026 12.654600

C 8.963864 13.251890 14.053587

H 7.979045 12.781507 13.942585

H 8.839338 14.329252 14.161387

O 11.444886 7.690786 4.598514

O 9.757174 13.061754 12.904695

C 14.085173 4.440882 11.069098

C 13.716143 5.245040 13.716815

C 13.792899 4.784610 8.669217

O 8.073583 6.089849 15.876790

H 8.871534 2.056153 13.566653

H 9.454319 12.853276 14.950317

C 14.370202 4.093002 9.707034

C 14.762334 3.782480 12.123851

C 14.571967 4.164901 13.426744

H 14.051876 4.582143 7.637169

C 7.623705 7.074440 16.778795

O 15.261702 3.085664 9.548982

H 15.447641 2.980763 11.876871

H 15.097878 3.655822 14.227889

H 7.175478 6.537921 17.614904

H 6.868270 7.724093 16.319609

H 8.453769 7.690882 17.145488

C 15.602943 2.701825 8.236862

H 14.721556 2.368891 7.674924

H 16.088493 3.521358 7.692632

H 16.302801 1.872206 8.335734

*T_1_*-**1c**

0 3

C 11.030516 7.148266 8.807541

C 11.477272 7.159450 10.172392

C 12.710890 6.427395 10.242259

C 13.585255 6.124271 11.335040

C 13.355490 6.538602 12.668277

H 12.471795 7.120393 12.898611

H 14.037189 6.543564 14.686549

C 13.021275 5.972967 8.950952

C 11.965239 6.425920 8.049380

C 9.879128 7.713684 8.170861

C 8.885133 8.455841 8.851486

H 8.980613 8.618224 9.917737

C 7.803133 8.971914 8.179376

H 7.054345 9.537631 8.724809

C 7.658498 8.773368 6.793591

H 6.800253 9.185346 6.272670

C 8.605246 8.057668 6.102205

H 8.506112 7.897528 5.035362

C 9.728329 7.515350 6.768283

C 10.717324 6.766159 6.039638

C 11.820690 6.228434 6.669993

H 12.566007 5.664128 6.122283

C 11.386837 5.923579 3.924973

H 10.996722 5.949191 2.907669

H 11.468794 4.880818 4.255382

H 12.380779 6.387238 3.943547

C 9.806382 7.195396 12.141887

C 9.401249 8.147190 13.090782

C 10.146974 9.377690 12.839524

C 10.099216 10.606264 13.511060

H 9.432117 10.738154 14.354667

C 10.915175 11.628847 13.072353

C 11.800982 11.453734 11.952156

C 12.628461 12.515427 11.519559

H 12.581046 13.459868 12.048176

C 13.475592 12.352869 10.450660

H 14.106550 13.174220 10.126736

C 13.524172 11.118808 9.775767

H 14.194038 10.991344 8.931201

C 12.730621 10.070331 10.175485

H 12.780855 9.127894 9.644771

C 11.846548 10.202816 11.272252

C 10.992557 9.153539 11.741714

C 10.794391 7.803075 11.295052

C 8.438793 7.868070 14.069760

H 8.153909 8.631585 14.783849

C 7.872314 6.610191 14.096624

C 8.250983 5.596543 13.148314

C 7.657712 4.314004 13.194796

H 6.914636 4.112675 13.957029

C 8.016148 3.342971 12.291914

C 8.983407 3.622567 11.308543

H 9.266130 2.854474 10.595561

C 9.575632 4.860911 11.240846

H 10.317966 5.058591 10.477810

C 9.230228 5.884437 12.154705

C 10.120981 13.115256 14.742176

H 9.062344 13.005028 14.476842

H 10.313394 14.147576 15.033812

O 10.467925 6.643349 4.714795

O 10.958097 12.857942 13.637977

C 14.755484 5.359927 11.060146

C 14.236373 6.213518 13.671828

C 14.170826 5.220810 8.676321

O 6.933850 6.222056 14.991669

H 7.554775 2.361742 12.336382

H 10.352930 12.451657 15.584255

C 15.025019 4.919370 9.717202

C 15.644620 5.039864 12.111835

C 15.391375 5.458268 13.395350

H 14.372869 4.889574 7.664669

C 6.504933 7.154624 15.957400

O 16.164285 4.201696 9.577677

H 16.529832 4.457953 11.884989

H 16.080335 5.207193 14.195328

H 5.758996 6.641112 16.563728

H 6.047653 8.034902 15.489144

H 7.334012 7.477024 16.599213

C 16.504928 3.730459 8.293857

H 15.739542 3.050253 7.900461

H 16.649183 4.557976 7.588403

H 17.443232 3.188298 8.409526

**1c**-TS_rot_ (OS, S^2^ = 1.097232)

0 1

C 11.031493 7.149205 8.806742

C 11.477351 7.159476 10.172742

C 12.710892 6.425609 10.242099

C 13.584474 6.120997 11.334949

C 13.354533 6.534997 12.668289

H 12.471357 7.117722 12.898371

H 14.035335 6.538200 14.686888

C 13.021163 5.971856 8.950843

C 11.965797 6.426525 8.048895

C 9.881046 7.716037 8.169838

C 8.887364 8.458553 8.850569

H 8.982496 8.619894 9.917031

C 7.806214 8.976185 8.178289

H 7.057641 9.542160 8.723750

C 7.662197 8.778859 6.792235

H 6.804636 9.192073 6.271165

C 8.608615 8.062768 6.100761

H 8.509869 7.903539 5.033739

C 9.730828 7.518848 6.767025

C 10.719499 6.769189 6.038517

C 11.821931 6.229973 6.669292

H 12.567054 5.665251 6.121735

C 11.389496 5.927218 3.923837

H 11.000080 5.953776 2.906289

H 11.470181 4.884194 4.253742

H 12.383905 6.389850 3.943326

C 9.807173 7.194954 12.143636

C 9.401794 8.146901 13.091854

C 10.146303 9.378224 12.839397

C 10.097407 10.607152 13.510234

H 9.430544 10.738747 14.354082

C 10.911939 11.630478 13.070520

C 11.797480 11.455632 11.950143

C 12.623660 12.517883 11.516387

H 12.575411 13.462707 12.044259

C 13.470600 12.355328 10.447316

H 14.100545 13.177103 10.122495

C 13.520390 11.120735 9.773413

H 14.190183 10.993313 8.928781

C 12.728110 10.071731 10.174256

H 12.779183 9.128798 9.644452

C 11.844174 10.204244 11.271164

C 10.991496 9.154465 11.741571

C 10.794792 7.802848 11.295109

C 8.440051 7.867680 14.071524

H 8.154919 8.631471 14.785227

C 7.874637 6.609317 14.099604

C 8.253706 5.595404 13.151822

C 7.661668 4.312317 13.199327

H 6.919143 4.110725 13.962040

C 8.020612 3.341125 12.296795

C 8.987117 3.621041 11.312727

H 9.270184 2.852796 10.600043

C 9.578129 4.859907 11.244033

H 10.319906 5.058004 10.480531

C 9.232202 5.883599 12.157549

C 10.116299 13.117219 14.739299

H 9.057789 13.005232 14.474184

H 10.307287 14.150089 15.029929

O 10.470811 6.647490 4.713434

O 10.953651 12.860028 13.635272

C 14.754009 5.355541 11.060127

C 14.234576 6.208431 13.672087

C 14.170034 5.218680 8.676071

O 6.936990 6.220939 14.995412

H 7.560190 2.359484 12.342046

H 10.349266 12.454760 15.582001

C 15.023567 4.915576 9.717043

C 15.642298 5.033951 12.112094

C 15.388860 5.451986 13.395708

H 14.372062 4.887928 7.664251

C 6.507317 7.154028 15.960280

O 16.162112 4.196752 9.577447

H 16.526996 4.451191 11.885403

H 16.077158 5.199726 14.195884

H 5.761858 6.640438 16.567132

H 6.049263 8.033475 15.491215

H 7.336148 7.477758 16.601753

C 16.502723 3.726229 8.293374

H 15.736804 3.047087 7.899166

H 16.648001 4.554181 7.588634

H 17.440463 3.183062 8.408909

*S_0_*-(*M*,*M*)-**3**

0 1

C 12.357956 8.513251 6.077285

C 11.900216 9.812158 6.077295

C 13.704641 8.039124 5.697861

C 10.553538 10.286300 5.697877

C 13.767259 6.679099 5.911441

C 10.490932 11.646326 5.911459

C 12.410900 6.200869 6.243772

C 11.847295 12.124541 6.243800

C 11.606533 7.299344 6.457141

C 12.651647 11.026054 6.457163

C 14.770892 8.706666 4.986065

C 9.487280 9.618765 4.986089

C 16.009755 8.027094 4.854407

C 8.248427 10.298350 4.854418

C 16.179190 6.711207 5.459995

C 8.079002 11.614240 5.460003

C 15.044485 6.012589 5.949211

C 9.213710 12.312846 5.949231

C 14.605477 9.945600 4.331672

C 9.652680 8.379812 4.331727

H 13.642020 10.436014 4.354320

H 10.616128 7.889380 4.354400

C 15.637013 10.530041 3.633442

C 8.621140 7.795376 3.633499

H 15.478735 11.481845 3.137212

H 8.779406 6.843558 3.137290

C 16.875891 9.882935 3.542578

C 7.382273 8.442501 3.542612

H 17.689605 10.335250 2.985109

H 6.568556 7.990187 2.985147

C 17.046501 8.648779 4.129462

C 7.211676 9.676669 4.129476

H 17.993155 8.138002 3.997951

H 6.265029 10.187454 3.997956

C 17.446068 6.109007 5.598071

C 6.812127 12.216448 5.598077

H 18.330385 6.630464 5.251322

H 5.927809 11.695000 5.251321

C 17.599899 4.882642 6.207500

C 6.658302 13.442809 6.207517

H 18.589790 4.449121 6.306853

H 5.668414 13.876335 6.306869

C 16.484141 4.217720 6.733293

C 7.774061 14.107715 6.733327

H 16.605968 3.275610 7.257664

H 7.652237 15.049818 7.257710

C 15.232847 4.778448 6.611620

C 9.025351 13.546978 6.611657

H 14.383725 4.288351 7.071138

H 9.874472 14.037061 7.071192

C 11.834551 4.880501 6.207599

C 12.423669 13.444900 6.207629

C 10.513645 4.712648 6.699487

C 13.744575 13.612729 6.699526

C 9.820838 5.844056 7.305239

C 14.437362 12.481306 7.305274

C 10.358169 7.150576 7.170526

C 13.900013 11.174796 7.170547

C 12.461082 3.801182 5.544377

C 11.797168 14.524228 5.544393

H 13.428360 3.952383 5.081981

H 10.829894 14.373044 5.081984

C 11.838683 2.579182 5.424871

C 12.419591 15.746217 5.424891

H 12.334002 1.769003 4.899825

H 11.924293 16.556403 4.899835

C 10.553891 2.396868 5.953580

C 13.704380 15.928508 5.953612

H 10.055183 1.438003 5.855918

H 14.203108 16.887363 5.855951

C 9.905095 3.448598 6.563514

C 14.353151 14.876767 6.563554

H 8.889989 3.299612 6.912031

H 15.368258 15.025733 6.912076

C 8.625195 5.677946 8.033061

C 15.633010 12.647389 8.033095

H 8.209905 4.685976 8.167117

H 16.048316 13.639350 8.167163

C 9.709630 8.219810 7.823799

C 14.548542 10.105541 7.823794

H 10.150610 9.206778 7.798153

H 14.107552 9.118577 7.798124

C 7.984012 6.746488 8.619402

C 16.274179 11.578828 8.619418

C 8.541205 8.028026 8.524634

C 15.716972 10.297298 8.524628

H 7.067975 6.588281 9.179161

H 17.190221 11.737014 9.179175

H 8.067353 8.869091 9.019900

H 16.190816 9.456218 9.019876

*T_1_*-(*M*,*M*)-**3**

0 3

C 12.367440 8.471706 6.080538

C 11.882179 9.849919 6.082078

C 13.141710 7.852746 5.040569

C 10.544382 10.274027 5.771956

C 13.469917 6.564576 5.459039

C 10.477461 11.653112 5.959798

C 12.715606 6.295901 6.700926

C 11.853399 12.135539 6.198953

C 12.156481 7.501617 7.119793

C 12.661742 11.016705 6.390939

C 13.584899 8.371373 3.769566

C 9.392698 9.509964 5.359126

C 14.475646 7.577188 3.003177

C 8.147732 10.183471 5.267233

C 15.024295 6.347713 3.573508

C 8.037155 11.568346 5.723267

C 14.534605 5.852176 4.815485

C 9.203586 12.297366 6.091732

C 13.115961 9.589257 3.236126

C 9.471342 8.154058 4.980888

H 12.421162 10.192441 3.806048

H 10.421157 7.637588 5.030626

C 13.503582 10.013138 1.984347

C 8.363181 7.479520 4.518570

H 13.125643 10.951595 1.592235

H 8.448804 6.437613 4.228097

C 14.366766 9.223287 1.215069

C 7.136249 8.144981 4.406502

H 14.660626 9.544343 0.221051

H 6.264455 7.622731 4.026052

C 14.837171 8.029564 1.718190

C 7.037543 9.470100 4.772014

H 15.488219 7.427952 1.095095

H 6.081128 9.966353 4.657857

C 16.086137 5.656037 2.957515

C 6.786816 12.200140 5.875216

H 16.484039 6.009124 2.013917

H 5.879668 11.672581 5.606847

C 16.674905 4.556594 3.545468

C 6.673226 13.468317 6.404266

H 17.497344 4.054253 3.046571

H 5.692787 13.919968 6.515635

C 16.243264 4.119942 4.805308

C 7.816746 14.151066 6.840566

H 16.742471 3.292428 5.298597

H 7.726913 15.124249 7.311910

C 15.198763 4.762904 5.428211

C 9.054829 13.570080 6.692701

H 14.901420 4.456769 6.423214

H 9.931036 14.076997 7.076775

C 12.341791 5.070779 7.344735

C 12.447162 13.433132 6.062136

C 11.649382 5.145058 8.586857

C 13.813225 13.592645 6.431238

C 11.295745 6.444461 9.156460

C 14.590014 12.443480 6.893861

C 11.484357 7.623089 8.390269

C 14.037909 11.139977 6.805700

C 12.517145 3.806827 6.732533

C 11.770304 14.517708 5.455018

H 12.941623 3.757827 5.737712

H 10.769846 14.365450 5.070258

C 12.116022 2.648137 7.356076

C 12.375950 15.743226 5.301593

H 12.253542 1.691274 6.863314

H 11.839813 16.557536 4.825383

C 11.506027 2.713504 8.616172

C 13.695293 15.924837 5.738108

H 11.187891 1.804228 9.115800

H 14.180178 16.888712 5.622022

C 11.265882 3.937544 9.203597

C 14.396915 14.865316 6.273189

H 10.735004 3.958976 10.147546

H 15.435095 15.019379 6.541224

C 10.727307 6.566357 10.440451

C 15.901254 12.581357 7.391996

H 10.601625 5.688461 11.063059

H 16.337593 13.566888 7.503717

C 11.076197 8.862996 8.923009

C 14.821920 10.033096 7.190218

H 11.233110 9.769554 8.352990

H 14.402327 9.036514 7.143909

C 10.335925 7.788491 10.942955

C 16.650518 11.485933 7.763531

C 10.506135 8.946254 10.173928

C 16.107665 10.199761 7.654934

H 9.903438 7.851316 11.936186

H 17.656542 11.623577 8.146235

H 10.204458 9.912170 10.565471

H 16.690445 9.333452 7.950220

(*M*,*M*)-**3**-TS_rot_ (OS, S^2^ = 1.070994)

0 1

C 12.366930 8.472548 6.074664

C 11.883282 9.850052 6.074451

C 13.044016 7.814677 4.988432

C 10.577263 10.287308 5.656436

C 13.409146 6.541539 5.420995

C 10.491522 11.658620 5.887264

C 12.773104 6.317882 6.735061

C 11.837700 12.131062 6.269312

C 12.253553 7.537476 7.163348

C 12.629241 11.007050 6.495051

C 13.381041 8.290356 3.668283

C 9.464445 9.539104 5.123156

C 14.176280 7.453039 2.844192

C 8.240377 10.226193 4.917872

C 14.764818 6.235768 3.400240

C 8.088413 11.597535 5.401367

C 14.403910 5.797209 4.705453

C 9.211409 12.303710 5.917611

C 12.901504 9.509874 3.148130

C 9.564452 8.185775 4.740751

H 12.294996 10.156254 3.768580

H 10.493103 7.652166 4.894027

C 13.170807 9.884957 1.850514

C 8.505327 7.533068 4.149864

H 12.785022 10.825270 1.470671

H 8.608745 6.493171 3.857950

C 13.925197 9.045387 1.022004

C 7.306920 8.217015 3.912278

H 14.123476 9.327489 -0.006880

H 6.476362 7.712733 3.429201

C 14.415678 7.855572 1.514842

C 7.182988 9.536043 4.291632

H 14.987945 7.218476 0.850950

H 6.249092 10.046210 4.087492

C 15.749708 5.505570 2.705477

C 6.831254 12.233001 5.442084

H 16.048454 5.813991 1.711007

H 5.956143 11.722405 5.058885

C 16.388974 4.424894 3.274975

C 6.666497 13.481052 6.004229

H 17.150002 3.891972 2.714224

H 5.681513 13.935910 6.027511

C 16.091037 4.047976 4.591602

C 7.759109 14.137171 6.587326

H 16.634376 3.236969 5.065266

H 7.622772 15.091885 7.084547

C 15.122556 4.728583 5.292301

C 9.003844 13.552889 6.549570

H 14.929031 4.467926 6.325111

H 9.836081 14.038280 7.043467

C 12.472408 5.115322 7.455531

C 12.431611 13.435763 6.244934

C 11.917618 5.232856 8.761116

C 13.749257 13.586157 6.761954

C 11.606004 6.550667 9.312190

C 14.490196 12.419470 7.238575

C 11.692385 7.698664 8.483085

C 13.965999 11.118156 7.027443

C 12.589530 3.830439 6.873894

C 11.810759 14.542725 5.618752

H 12.902879 3.745873 5.841037

H 10.858329 14.402144 5.123528

C 12.269591 2.694016 7.579823

C 12.414508 15.778339 5.588121

H 12.360117 1.720079 7.110184

H 11.922580 16.610484 5.095334

C 11.802396 2.803550 8.896650

C 13.676861 15.948520 6.172786

H 11.549860 1.912052 9.461535

H 14.159193 16.920479 6.155506

C 11.616304 4.047483 9.461228

C 14.330539 14.869506 6.728810

H 11.191385 4.101752 10.456125

H 15.332190 15.018054 7.113418

C 11.168312 6.717305 10.641619

C 15.747788 12.540658 7.863459

H 11.128840 5.865207 11.309748

H 16.156782 13.522224 8.071542

C 11.293214 8.948926 8.998282

C 14.736580 9.998732 7.402945

H 11.352804 9.830401 8.373826

H 14.347177 9.001821 7.244998

C 10.797306 7.951493 11.129663

C 16.477984 11.432967 8.236067

C 10.849457 9.075134 10.295950

C 15.971879 10.150404 7.992740

H 10.467308 8.049321 12.158741

H 17.442163 11.557723 8.718029

H 10.554166 10.049246 10.671759

H 16.543188 9.273517 8.279076

**CAM-B3LYP/6-311++G**//6-31G****

CS-**1a**

0 1

C 21.427043 0.370131 12.436887

C 21.575549 0.418973 13.821203

C 21.906646 1.381757 11.590083

H 21.789807 1.299719 10.514479

C 22.522512 2.468799 12.146061

H 22.922917 3.257527 11.516311

C 22.607307 2.620818 13.553665

C 22.089386 1.612354 14.425464

C 23.170460 3.799727 14.103679

H 23.574409 4.542862 13.422355

C 23.185954 4.013674 15.453790

H 23.612205 4.923852 15.862374

C 22.614133 3.053117 16.312901

H 22.584408 3.238008 17.381774

C 22.084553 1.891631 15.815304

H 21.639030 1.183917 16.497827

C 20.696656 -0.839922 12.078439

C 20.901219 -0.770714 14.393808

C 20.377058 -1.317931 10.797872

C 20.356610 -1.527680 13.241254

C 20.800379 -1.107166 15.734965

C 19.727906 -2.516269 10.685456

H 20.641138 -0.744685 9.915287

C 19.805219 -2.846030 13.135358

C 19.569994 -1.490171 16.468010

C 21.901008 -1.157116 16.726993

H 19.448320 -2.902370 9.709874

C 19.451266 -3.308240 11.829084

C 19.624065 -3.745017 14.216416

C 18.182960 -1.416410 16.115856

C 19.920466 -1.734753 17.793943

C 21.358898 -1.552102 17.948015

C 23.325526 -1.105434 16.580871

C 18.858515 -4.586764 11.675725

C 19.071312 -4.984742 14.030278

H 19.943453 -3.456654 15.206372

C 17.678983 -0.957041 14.873014

C 17.229947 -1.793751 17.113180

C 18.974353 -2.069709 18.775349

C 22.144945 -1.751216 19.093862

C 24.015459 -0.944060 15.353022

C 24.114640 -1.274862 17.761490

H 18.581174 -4.908339 10.676078

C 18.660726 -5.408826 12.749927

H 18.956709 -5.650674 14.879372

C 16.334413 -0.935732 14.610748

H 18.365597 -0.599103 14.120933

C 15.847513 -1.783642 16.799723

C 17.654796 -2.135529 18.422517

H 19.287347 -2.272578 19.794262

H 21.684638 -2.040095 20.032995

C 23.497860 -1.573838 19.003196

H 23.454690 -0.882065 14.432766

C 25.383447 -0.886754 15.303882

C 25.526907 -1.185000 17.678344

H 18.213844 -6.388693 12.618738

C 15.402590 -1.374061 15.573710

H 15.983708 -0.568338 13.651797

H 15.143966 -2.092203 17.567470

H 16.901748 -2.412722 19.154013

H 24.130113 -1.699646 19.876941

H 25.878474 -0.772289 14.345026

C 26.154261 -0.985544 16.480237

H 26.103958 -1.298449 18.591452

H 14.341367 -1.362690 15.348474

H 27.236344 -0.926942 16.428125

OS-**1a** (≡ *S_0_*-**1a**, S^2^=0.417566)

0 1

C 21.398149 0.388017 12.433672

C 21.523404 0.443647 13.823815

C 21.851541 1.417097 11.596611

H 21.750579 1.335415 10.519272

C 22.423614 2.524525 12.163392

H 22.799571 3.329689 11.539403

C 22.502929 2.670655 13.572656

C 22.017044 1.641018 14.437481

C 23.035449 3.858919 14.131730

H 23.412304 4.621589 13.456472

C 23.059651 4.055775 15.484937

H 23.464012 4.972741 15.900537

C 22.531314 3.066254 16.338221

H 22.516599 3.233123 17.410367

C 22.026731 1.896973 15.830993

H 21.616382 1.163812 16.509285

C 20.725768 -0.854909 12.071354

C 20.902074 -0.767462 14.381234

C 20.431271 -1.353816 10.794790

C 20.408443 -1.552320 13.239334

C 20.799357 -1.110303 15.747553

C 19.824934 -2.576903 10.688408

H 20.679682 -0.779430 9.908283

C 19.876198 -2.879459 13.142623

C 19.566447 -1.434254 16.481405

C 21.904400 -1.212872 16.712910

H 19.569256 -2.981095 9.713600

C 19.553650 -3.364776 11.837027

C 19.679840 -3.759237 14.235753

C 18.181623 -1.338171 16.125061

C 19.915463 -1.703898 17.806819

C 21.362883 -1.585529 17.945449

C 23.328136 -1.178904 16.552354

C 18.990620 -4.657038 11.691511

C 19.151101 -5.011399 14.057890

H 19.964273 -3.445226 15.228972

C 17.692797 -0.898088 14.870121

C 17.219994 -1.677883 17.127077

C 18.961449 -2.006957 18.788275

C 22.157830 -1.813047 19.077500

C 24.004130 -0.994344 15.320909

C 24.126404 -1.383972 17.720633

H 18.739848 -4.999667 10.691824

C 18.783612 -5.465302 12.775195

H 19.020965 -5.662557 14.916153

C 16.348711 -0.848839 14.605331

H 18.390191 -0.581123 14.109093

C 15.838832 -1.634350 16.813064

C 17.637715 -2.023447 18.438483

H 19.268481 -2.224762 19.806027

H 21.703005 -2.088357 20.023470

C 23.516201 -1.680761 18.966929

H 23.432525 -0.891621 14.410575

C 25.372998 -0.960486 15.256431

C 25.538678 -1.323260 17.621171

H 18.357897 -6.455331 12.650029

C 15.405674 -1.237026 15.578148

H 16.008233 -0.499667 13.635944

H 15.126496 -1.910518 17.585052

H 16.877753 -2.271694 19.173214

H 24.155832 -1.833802 19.830899

H 25.858771 -0.824722 14.295622

C 26.155257 -1.108452 16.419422

H 26.124947 -1.467500 18.524024

H 14.345169 -1.201593 15.352027

H 27.237614 -1.070618 16.355183

*T_1_*-**1a**

0 3

C 21.203483 0.460225 12.425617

C 21.187072 0.551913 13.828401

C 21.451990 1.574329 11.618282

H 21.460441 1.482959 10.536753

C 21.685777 2.788713 12.217477

H 21.881183 3.667908 11.610952

C 21.680155 2.934802 13.630840

C 21.428100 1.807877 14.471024

C 21.924134 4.198190 14.222257

H 22.114267 5.046093 13.570600

C 21.921880 4.354561 15.582501

H 22.110268 5.327968 16.023047

C 21.673106 3.242895 16.411862

H 21.671245 3.366773 17.489914

C 21.432916 2.004287 15.872353

H 21.244301 1.165199 16.528624

C 20.928546 -0.927321 12.057974

C 20.905076 -0.761561 14.354943

C 20.837494 -1.524645 10.797143

C 20.748047 -1.665126 13.240992

C 20.796759 -1.116111 15.773822

C 20.564047 -2.869054 10.718336

H 20.980022 -0.937684 9.895316

C 20.462841 -3.066333 13.179581

C 19.590390 -1.087744 16.565007

C 21.877668 -1.554694 16.623045

H 20.488495 -3.354743 9.750120

C 20.373385 -3.659485 11.883595

C 20.265478 -3.895242 14.309304

C 18.241918 -0.735293 16.239635

C 19.919398 -1.498644 17.868596

C 21.350939 -1.790847 17.904951

C 23.270632 -1.761575 16.367354

C 20.092838 -5.043190 11.773622

C 19.995191 -5.232916 14.166195

H 20.328737 -3.467990 15.301160

C 17.809865 -0.310522 14.960862

C 17.272844 -0.821625 17.285028

C 18.957421 -1.577358 18.880123

C 22.154765 -2.229756 18.961353

C 23.896221 -1.552670 15.115447

C 24.076366 -2.209836 17.457881

H 20.027806 -5.480315 10.781432

C 19.906868 -5.817757 12.887254

H 19.847907 -5.846749 15.048841

C 16.496013 0.010252 14.728943

H 18.526242 -0.237455 14.153518

C 15.925704 -0.482731 17.009663

C 17.657301 -1.242938 18.586428

H 19.231500 -1.896970 19.880549

H 21.729031 -2.406498 19.944025

C 23.494687 -2.433965 18.734753

H 23.305824 -1.213147 14.274922

C 25.240427 -1.774076 14.951058

C 25.460337 -2.428060 17.251915

H 19.692488 -6.876782 12.789271

C 15.540130 -0.075098 15.760717

H 16.190494 0.333343 13.739087

H 15.199899 -0.553215 17.814641

H 16.892934 -1.296386 19.355796

H 24.138622 -2.774646 19.539940

H 25.695880 -1.606268 13.980518

C 26.035095 -2.216389 16.027335

H 26.060470 -2.768961 18.090589

H 14.504762 0.181708 15.562836

H 27.096683 -2.387325 15.882752

**1a**-TS_rot_ (OS, S^2^ = 1.129022)

0 1

C 21.202837 0.460589 12.425269

C 21.186160 0.552545 13.827806

C 21.450525 1.574736 11.617746

H 21.459165 1.483185 10.536242

C 21.683259 2.789458 12.216751

H 21.877961 3.668710 11.610094

C 21.677496 2.935718 13.630050

C 21.426306 1.808610 14.470266

C 21.920492 4.199297 14.221525

H 22.109973 5.047383 13.569928

C 21.918153 4.355566 15.581774

H 22.105759 5.329106 16.022346

C 21.670368 3.243665 16.411178

H 21.668546 3.367547 17.489222

C 21.431124 2.004908 15.871653

H 21.243313 1.165523 16.527791

C 20.929113 -0.927448 12.057614

C 20.905061 -0.761613 14.355306

C 20.838752 -1.524753 10.796725

C 20.749089 -1.665391 13.240355

C 20.796727 -1.115975 15.773477

C 20.566446 -2.869423 10.717780

H 20.980953 -0.937632 9.894961

C 20.464902 -3.066673 13.178914

C 19.590249 -1.086892 16.565836

C 21.877622 -1.555908 16.623443

H 20.491509 -3.355128 9.749532

C 20.376163 -3.659998 11.882948

C 20.267833 -3.895545 14.308766

C 18.242048 -0.733553 16.240818

C 19.919305 -1.498170 17.869076

C 21.350841 -1.791639 17.905137

C 23.270282 -1.763677 16.367607

C 20.096653 -5.043950 11.773105

C 19.998554 -5.233411 14.165764

H 20.330505 -3.467966 15.300526

C 17.810132 -0.308670 14.962001

C 17.273111 -0.819100 17.286416

C 18.957601 -1.576225 18.880900

C 22.154365 -2.231292 18.961468

C 23.895764 -1.554790 15.115587

C 24.075859 -2.212771 17.457918

H 20.032132 -5.481281 10.780980

C 19.911001 -5.818457 12.886825

H 19.851469 -5.847273 15.048412

C 16.496498 0.013073 14.730273

H 18.526516 -0.236380 14.154585

C 15.926174 -0.479178 17.011228

C 17.657616 -1.240711 18.587661

H 19.231727 -1.896181 19.881195

H 21.728560 -2.407758 19.944148

C 23.494128 -2.436625 18.734758

H 23.305330 -1.214508 14.275382

C 25.239751 -1.777103 14.950896

C 25.459660 -2.431952 17.251599

H 19.697415 -6.877646 12.788939

C 15.540754 -0.071389 15.762284

H 16.191003 0.336226 13.740437

H 15.200420 -0.549024 17.816297

H 16.893464 -1.293565 19.357275

H 24.137826 -2.777945 19.539855

H 25.695213 -1.609319 13.980365

C 26.034274 -2.220351 16.026955

H 26.059743 -2.773490 18.090041

H 14.505556 0.186192 15.564552

H 27.095703 -2.392009 15.882107

CS-**1b**

0 1

C 8.685027 21.407154 10.215963

C 9.173955 22.663287 9.854308

H 8.837172 23.119688 8.927996

C 7.674095 20.693794 9.358976

H 6.652714 20.958564 9.653488

H 7.768225 19.609064 9.450028

H 7.787647 20.957402 8.304753

C 9.142256 20.842048 11.407335

C 10.082591 23.338133 10.653535

C 10.050531 21.506337 12.215751

H 8.780452 19.862334 11.705692

C 10.535162 22.768353 11.850174

H 10.454306 24.312745 10.357147

H 10.396543 21.051438 13.137378

C 11.473501 23.458848 12.674948

C 12.268810 24.048545 13.370473

C 13.208308 24.769030 14.163675

C 13.683990 24.236293 15.408943

C 13.634912 26.013299 13.742550

C 13.184604 23.003670 15.894077

C 14.634503 24.964384 16.190441

H 13.226157 26.439802 12.833474

C 14.591002 26.703310 14.488345

H 12.479137 22.459787 15.276650

C 13.558311 22.519129 17.117394

C 14.960490 24.450118 17.470137

C 15.161109 26.181781 15.648736

C 15.140478 28.033188 14.247605

H 13.159579 21.576518 17.477304

C 14.440026 23.266503 17.921640

H 15.620534 25.012314 18.112838

C 16.086510 27.195008 16.197128

C 14.886589 28.894758 13.180061

C 16.029774 28.347698 15.274152

H 14.704645 22.908255 18.911082

C 16.857364 27.085330 17.351651

H 14.173764 28.627272 12.408218

C 15.546562 30.105883 13.108453

C 16.836734 29.525402 15.152626

C 16.945000 28.062978 18.456260

C 17.752066 25.967838 17.718241

C 15.245455 31.002260 12.042259

C 16.551229 30.428705 14.081700

C 17.929318 29.838679 15.999072

C 16.171884 29.225745 18.777321

C 17.822667 27.554047 19.412418

C 18.244834 24.845188 16.976844

C 18.334142 26.268798 18.948739

C 14.966875 31.744170 11.127943

C 17.297011 31.626406 13.965840

C 18.648179 30.994167 15.844850

H 18.214432 29.142658 16.773048

C 15.090134 29.708335 17.999322

C 16.481288 29.926783 19.984453

C 18.099228 28.215386 20.609315

C 19.171991 23.968171 17.621503

C 17.907612 24.564823 15.629236

C 19.268010 25.435154 19.564653

C 14.632834 32.615420 10.047730

H 17.051029 32.305182 13.157469

C 18.316853 31.913446 14.830985

H 19.484633 31.196370 16.505771

C 14.404445 30.841280 18.348562

H 14.786439 29.161167 17.120012

C 15.769987 31.108581 20.303053

C 17.474508 29.415654 20.886152

H 18.802637 27.801130 21.322770

C 19.636957 22.821674 16.933421

C 19.659505 24.268307 18.937952

H 17.265822 25.246605 15.092503

C 18.395009 23.460323 14.982547

H 19.686603 25.689591 20.531838

C 13.656702 32.249948 9.112275

C 15.275415 33.850474 9.896554

H 18.883520 32.832257 14.722772

H 13.575180 31.175856 17.733959

C 14.760301 31.566483 19.501927

H 16.033846 31.633860 21.213641

C 17.799795 30.110501 22.087110

C 19.252706 22.562391 15.646536

H 20.324817 22.160931 17.448178

C 20.575340 23.396811 19.595722

H 18.122853 23.282695 13.947298

H 13.155425 31.293984 9.215896

C 13.337688 33.097048 8.063284

C 14.946847 34.688377 8.843079

H 16.036672 34.143723 10.611079

H 14.220008 32.469648 19.765390

C 18.098760 30.679601 23.112175

H 19.625086 21.683295 15.131208

C 21.350872 22.674755 20.179845

H 12.580592 32.794703 7.345525

C 13.972346 34.330579 7.910327

H 15.458702 35.640835 8.740224

C 18.455839 31.346133 24.322853

C 22.265715 21.827257 20.874341

C 13.598276 35.259470 6.786700

C 17.855354 32.560499 24.677734

C 19.419637 30.800462 25.180014

C 22.723147 20.637160 20.295022

C 22.721755 22.167036 22.154187

H 13.256573 34.706283 5.908623

H 12.786028 35.930887 7.085891

H 14.443269 35.884002 6.487195

H 17.111074 32.996629 24.020548

C 18.210649 33.202106 25.853261

H 19.895359 29.863089 24.913731

C 19.765706 31.452215 26.352739

H 22.374149 20.359630 19.306470

C 23.608522 19.819011 20.978054

C 23.607266 21.340075 22.826342

H 22.370920 23.083579 22.615433

H 17.736615 34.145096 26.109827

C 19.167560 32.660612 26.712859

H 20.517959 31.015336 27.003146

H 23.949341 18.898042 20.513922

C 24.069070 20.154946 22.251949

H 23.947059 21.618493 23.819722

C 19.525160 33.347094 28.003653

C 25.055363 19.277353 22.974688

H 18.876795 33.010902 28.820142

H 19.412414 34.430932 27.923760

H 20.555675 33.133392 28.297129

H 24.935753 18.228009 22.694746

H 26.085240 19.563665 22.735048

H 24.939538 19.355237 24.058336

OS-**1b** (≡ *S_0_*-**1b**, S^2^ = 0.924583)

0 1

C 6.418513 23.666923 11.971009

C 7.303737 24.509803 11.297345

H 7.053016 24.857820 10.299453

C 5.109231 23.262619 11.348501

H 4.316368 23.973957 11.604305

H 4.788447 22.278276 11.697714

H 5.178642 23.230504 10.258591

C 6.771334 23.228987 13.248399

C 8.497254 24.909205 11.876635

C 7.961726 23.620724 13.839181

H 6.100847 22.567100 13.788860

C 8.844951 24.469866 13.160423

H 9.175003 25.563025 11.338997

H 8.220857 23.269244 14.831827

C 10.074592 24.875964 13.759402

C 11.118870 25.223881 14.263769

C 12.352559 25.653361 14.826716

C 12.757157 25.217493 16.135898

C 13.177028 26.488817 14.087452

C 11.943165 24.331024 16.878872

C 13.996487 25.659330 16.692130

H 12.875750 26.786281 13.089096

C 14.373020 26.935687 14.635794

H 11.002273 24.013428 16.444462

C 12.332400 23.872677 18.109227

C 14.375603 25.145912 17.955400

C 14.789044 26.577012 15.927426

C 15.395578 27.797299 14.047330

H 11.696786 23.190171 18.663521

C 13.568364 24.278121 18.645288

H 15.324880 25.436055 18.381498

C 16.080023 27.195338 16.168569

C 15.417229 28.412392 12.801574

C 16.434770 27.947437 14.978839

H 13.889785 23.899417 19.609987

C 16.869304 27.082809 17.376305

H 14.604067 28.274632 12.097477

C 16.494963 29.211403 12.448954

C 17.529997 28.821384 14.675817

C 16.510317 27.595658 18.685966

C 18.163008 26.436035 17.498531

C 16.517075 29.814198 11.160750

C 17.561449 29.436313 13.386855

C 18.571974 29.130540 15.582364

C 15.411136 28.392410 19.146401

C 17.549216 27.276714 19.574229

C 18.960706 25.682971 16.575667

C 18.576292 26.546294 18.835397

C 16.509139 30.312278 10.057207

C 18.642758 30.284214 13.051827

C 19.600673 29.966285 15.230246

H 18.552378 28.706816 16.575815

C 14.369537 28.864175 18.312305

C 15.375248 28.752126 20.528383

C 17.523356 27.645015 20.913800

C 20.201798 25.149936 17.040549

C 18.585193 25.415704 15.237500

C 19.774014 26.009504 19.291506

C 16.494116 30.896815 8.755750

H 18.653944 30.735150 12.066234

C 19.645808 30.542651 13.947634

H 20.382377 30.186380 15.949877

C 13.336907 29.614286 18.813971

H 14.392703 28.636975 17.256499

C 14.289892 29.517084 21.015469

C 16.441523 28.358200 21.409021

H 18.336327 27.380041 21.580761

C 21.020828 24.423697 16.144916

C 20.603219 25.332623 18.409168

H 17.634829 25.776616 14.872215

C 19.397352 24.697748 14.397488

H 20.073061 26.114540 20.328572

C 15.427228 30.660114 7.879542

C 17.545776 31.715017 8.324000

H 20.465772 31.198539 13.674633

H 12.555646 29.964146 18.147186

C 13.287212 29.936793 20.182503

H 14.275317 29.773129 22.068600

C 16.415002 28.706302 22.787960

C 20.634895 24.204218 14.849440

H 21.962940 24.034227 16.513154

C 21.838671 24.810670 18.883031

H 19.078640 24.506652 13.378038

H 14.609414 30.024436 8.200434

C 15.418007 31.227243 6.615477

C 17.524154 32.275833 7.057303

H 18.380389 31.902501 8.990561

H 12.464110 30.525990 20.572899

C 16.419130 28.986982 23.965734

H 21.274369 23.641778 14.177284

C 22.884347 24.379178 19.314693

H 14.583752 31.029333 5.948684

C 16.461588 32.046303 6.181928

H 18.350523 32.904702 6.738890

C 16.428795 29.314159 25.354465

C 24.115601 23.873941 19.828940

C 16.429772 32.684722 4.819356

C 15.373948 30.034016 25.929770

C 17.497840 28.925361 26.171759

C 25.004781 23.173494 19.003946

C 24.458994 24.064500 21.173467

H 15.877386 32.070945 4.103884

H 15.940197 33.664035 4.855763

H 17.437897 32.839220 4.427432

H 14.542288 30.345396 25.307341

C 15.393243 30.349917 27.278590

H 18.322780 28.371849 25.737044

C 17.504708 29.247906 27.519175

H 24.749031 23.014669 17.962120

C 26.196745 22.683851 19.512816

C 25.654165 23.569508 21.669621

H 23.776645 24.600998 21.823427

H 14.567905 30.911939 27.706023

C 16.454264 29.961605 28.098024

H 18.344203 28.941038 28.136304

H 26.871903 22.139940 18.858543

C 26.545295 22.874299 20.850832

H 25.901518 23.723930 22.715916

C 16.453109 30.280963 29.568807

C 27.856227 22.366986 21.388684

H 15.965466 29.485679 30.143085

H 15.914226 31.208744 29.775259

H 17.469709 30.385249 29.955316

H 28.181645 21.466328 20.862640

H 28.645898 23.117028 21.270674

H 27.786637 22.132166 22.453461

*T_1_*-**1b**

0 3

C 7.120024 22.615145 11.479334

C 7.872613 23.635198 10.895312

H 7.584812 24.018486 9.920620

C 5.898546 22.064597 10.793738

H 5.001148 22.622871 11.081978

H 5.729886 21.018095 11.058634

H 5.987503 22.129916 9.706733

C 7.518681 22.136808 12.728477

C 8.981058 24.166539 11.534440

C 8.625004 22.659224 13.378495

H 6.951850 21.338713 13.199098

C 9.374779 23.685602 12.789831

H 9.556243 24.957603 11.066191

H 8.921658 22.274288 14.347950

C 10.517225 24.227650 13.450016

C 11.487574 24.691708 14.006690

C 12.630972 25.258760 14.632062

C 13.072301 24.793962 15.920561

C 13.321875 26.273986 13.980196

C 12.376766 23.761948 16.591506

C 14.221994 25.372180 16.537986

H 12.975879 26.612385 13.009608

C 14.439719 26.838146 14.577617

H 11.505579 23.332636 16.110162

C 12.787762 23.311327 17.818500

C 14.618038 24.882788 17.804626

C 14.908133 26.416113 15.836692

C 15.322420 27.902391 14.101747

H 12.241718 22.518425 18.318716

C 13.920659 23.879625 18.428954

H 15.486564 25.307702 18.288722

C 16.071483 27.197581 16.161562

C 15.280571 28.634704 12.924169

C 16.314799 28.112516 15.078402

H 14.247021 23.523453 19.400550

C 16.872493 27.081281 17.387356

H 14.508314 28.458500 12.183337

C 16.241508 29.610708 12.685456

C 17.321328 29.109878 14.867193

C 16.654749 27.808117 18.609569

C 18.010383 26.220856 17.573056

C 16.184640 30.351987 11.474148

C 17.275397 29.861464 13.654606

C 18.355795 29.391749 15.789781

C 15.678968 28.786359 18.988146

C 17.637808 27.406398 19.534263

C 18.665859 25.289146 16.704321

C 18.488326 26.410435 18.883944

C 16.111195 30.967550 10.433715

C 18.257061 30.851869 13.421343

C 19.293263 30.360021 15.532733

H 18.407713 28.833331 16.714286

C 14.655590 29.250593 18.129102

C 15.745433 29.322127 20.309466

C 17.699623 27.929125 20.817874

C 19.795272 24.579795 17.212835

C 18.258543 25.032585 15.374269

C 19.586425 25.719309 19.375164

C 16.019085 31.689695 9.206986

H 18.209209 31.414436 12.496033

C 19.246011 31.099428 14.336866

H 20.075944 30.555293 16.258439

C 13.747828 30.190341 18.547813

H 14.588303 28.856973 17.124217

C 14.794298 30.289114 20.708535

C 16.768944 28.880618 21.219709

H 18.464392 27.607376 21.516351

C 20.460386 23.654366 16.376058

C 20.247122 24.807081 18.560136

H 17.405181 25.559842 14.970516

C 18.926286 24.127687 14.588301

H 19.940040 25.878662 20.387998

C 14.997068 31.411527 8.290230

C 16.949126 32.687960 8.889802

H 19.991052 31.863390 14.141039

H 12.973004 30.528446 17.867589

C 13.815352 30.716954 19.850570

H 14.857511 30.686759 21.714940

C 16.846281 29.406345 22.537899

C 20.039191 23.429759 15.091682

H 21.316437 23.123286 16.776176

C 21.370169 24.107012 19.078469

H 18.591901 23.950572 13.571351

H 14.273824 30.637575 8.522163

C 14.913013 32.113124 7.098533

C 16.853526 33.382010 7.694576

H 17.748267 32.910047 9.588425

H 13.093790 31.459773 20.174047

C 16.936919 29.836513 23.666418

H 20.561829 22.715831 14.463807

C 22.323499 23.524979 19.546666

H 14.115679 31.880843 6.398485

C 15.834899 33.111316 6.779583

H 17.586165 34.150151 7.464488

C 17.048428 30.338922 24.996831

C 23.445948 22.842736 20.102995

C 15.718797 33.891279 5.497662

C 16.152056 31.301641 25.478343

C 18.061771 29.882258 25.849407

C 24.164037 21.906959 19.347432

C 23.851153 23.091566 21.420623

H 15.259554 33.293307 4.706911

H 15.097443 34.782934 5.635303

H 16.696805 34.227370 5.145072

H 15.365816 31.667413 24.827145

C 16.270054 31.786125 26.770906

H 18.764841 29.140659 25.486639

C 18.168481 30.375144 27.139821

H 23.858044 21.701230 18.327596

C 25.250947 21.246736 19.897029

C 24.939969 22.424300 21.957995

H 23.300354 23.810217 22.017311

H 15.567837 32.535146 27.125314

C 17.275566 31.331546 27.625563

H 18.963030 30.011703 27.785079

H 25.793164 20.521795 19.296860

C 25.661030 21.493903 21.208097

H 25.236996 22.627789 22.982751

C 17.377945 31.839139 29.038720

C 26.862035 20.796170 21.787667

H 16.779996 31.225052 29.720947

H 17.013777 32.865960 29.121275

H 18.409527 31.814532 29.397846

H 26.995481 19.803150 21.351888

H 27.778328 21.363953 21.592450

H 26.773656 20.682901 22.870762

**1b**-TS_rot_ (OS, S^2^=1.193372)

0 1

C 7.119901 22.615000 11.478620

C 7.872210 23.635405 10.894847

H 7.584221 24.018952 9.920312

C 5.898479 22.064413 10.792950

H 5.001100 22.622891 11.080848

H 5.729638 21.018010 11.058109

H 5.987656 22.129410 9.705942

C 7.518795 22.136345 12.727558

C 8.980612 24.166772 11.534026

C 8.625084 22.658788 13.377626

H 6.952185 21.337980 13.197986

C 9.374573 23.685511 12.789218

H 9.555576 24.958112 11.065973

H 8.921925 22.273594 14.346922

C 10.516989 24.227595 13.449454

C 11.487295 24.691688 14.006156

C 12.630677 25.258753 14.631613

C 13.071899 24.793931 15.920087

C 13.321666 26.274008 13.979843

C 12.376404 23.761911 16.591108

C 14.221580 25.372181 16.537520

H 12.975766 26.612457 13.009242

C 14.439491 26.838111 14.577409

H 11.505216 23.332540 16.109817

C 12.787473 23.311391 17.818111

C 14.617714 24.882880 17.804208

C 14.907685 26.416025 15.836314

C 15.322305 27.902517 14.101459

H 12.241467 22.518495 18.318379

C 13.920384 23.879733 18.428577

H 15.486260 25.307938 18.288175

C 16.071689 27.197588 16.161878

C 15.280291 28.634773 12.923815

C 16.314619 28.112773 15.077836

H 14.246733 23.523576 19.400182

C 16.872283 27.081358 17.387009

H 14.507980 28.458481 12.183065

C 16.241155 29.610834 12.684969

C 17.321020 29.110069 14.866595

C 16.654912 27.808269 18.610135

C 18.010818 26.220698 17.573387

C 16.184197 30.352106 11.473624

C 17.275060 29.861667 13.654008

C 18.355491 29.391867 15.789266

C 15.679250 28.786433 18.988729

C 17.637891 27.406463 19.534543

C 18.666270 25.289069 16.704756

C 18.488537 26.410367 18.884117

C 16.110722 30.967674 10.433205

C 18.256753 30.852087 13.420799

C 19.292948 30.360129 15.532261

H 18.407284 28.833327 16.713722

C 14.655888 29.250635 18.129581

C 15.745717 29.322185 20.310066

C 17.699843 27.929092 20.818220

C 19.795675 24.579693 17.213273

C 18.258879 25.032622 15.374664

C 19.586623 25.719281 19.375493

C 16.018565 31.689816 9.206463

H 18.208937 31.414699 12.495515

C 19.245670 31.099572 14.336373

H 20.075644 30.555412 16.257947

C 13.748124 30.190367 18.548253

H 14.588747 28.856917 17.124708

C 14.794537 30.289178 20.709090

C 16.769227 28.880607 21.220195

H 18.464657 27.607265 21.516607

C 20.460761 23.654286 16.376416

C 20.247414 24.807011 18.560556

H 17.405504 25.560003 14.971064

C 18.926586 24.127761 14.588655

H 19.940140 25.878684 20.388350

C 14.996506 31.411669 8.289765

C 16.948610 32.688066 8.889228

H 19.990722 31.863536 14.140592

H 12.973303 30.528498 17.868039

C 13.815640 30.716971 19.851052

H 14.857687 30.686837 21.715494

C 16.846630 29.406297 22.538431

C 20.039504 23.429789 15.092043

H 21.316815 23.123162 16.776470

C 21.370452 24.106930 19.078970

H 18.592226 23.950673 13.571694

H 14.273254 30.637734 8.521733

C 14.912403 32.113270 7.098062

C 16.852967 33.382114 7.694009

H 17.747789 32.910142 9.587812

H 13.094053 31.459784 20.174493

C 16.937289 29.836450 23.666945

H 20.562115 22.715886 14.464114

C 22.323761 23.524891 19.547185

H 14.115026 31.881003 6.398058

C 15.834286 33.111436 6.779062

H 17.585610 34.150242 7.463886

C 17.048864 30.338833 24.997376

C 23.446197 22.842657 20.103585

C 15.718188 33.891409 5.497145

C 16.153300 31.302471 25.478541

C 18.061473 29.881211 25.850305

C 24.163864 21.906313 19.348341

C 23.851800 23.092090 21.420977

H 15.257408 33.294000 4.706870

H 15.098259 34.783986 5.635218

H 16.696376 34.226048 5.143666

H 15.367638 31.668995 24.827069

C 16.271364 31.786916 26.771116

H 18.763914 29.138884 25.487811

C 18.168257 30.374065 27.140726

H 23.857570 21.700112 18.328691

C 25.250756 21.246109 19.898016

C 24.940587 22.424845 21.958428

H 23.301332 23.811202 22.017416

H 15.569779 32.536659 27.125251

C 17.276146 31.331388 27.626124

H 18.962230 30.009859 27.786264

H 25.792643 20.520724 19.298084

C 25.661227 21.493863 21.208844

H 25.237936 22.628816 22.982995

C 17.378598 31.838995 29.039273

C 26.862177 20.796106 21.788508

H 16.779878 31.225534 29.721379

H 17.015385 32.866169 29.121636

H 18.410056 31.813446 29.398690

H 26.995148 19.802755 21.353336

H 27.778604 21.363443 21.592654

H 26.774071 20.683561 22.871700

CS-**1c**

0 1

C 11.284709 7.477980 8.759632

C 11.456534 7.176826 10.198869

C 12.459885 6.090462 10.267507

C 13.206050 5.529701 11.357984

C 13.072180 5.921801 12.712271

H 12.386090 6.715865 12.964972

H 13.680917 5.663202 14.732786

C 12.855924 5.786094 8.970724

C 12.112535 6.625902 8.038613

C 10.312503 8.252925 8.042219

C 9.259568 8.983278 8.645240

H 9.142006 8.948923 9.717671

C 8.375247 9.718116 7.898504

H 7.576657 10.258424 8.396764

C 8.487581 9.770989 6.497247

H 7.789880 10.364857 5.916106

C 9.466037 9.045986 5.871108

H 9.550940 9.044320 4.791815

C 10.376486 8.261402 6.616436

C 11.331501 7.439046 5.935505

C 12.159378 6.598050 6.635161

H 12.836083 5.921372 6.130006

C 12.211291 6.741558 3.842316

H 12.044518 6.988100 2.794064

H 12.023540 5.672599 3.995913

H 13.251522 6.964280 4.106203

C 10.130216 7.117952 12.396775

C 9.609045 8.101273 13.228997

C 9.956161 9.411202 12.690184

C 9.682732 10.669026 13.252106

H 9.118036 10.738923 14.172569

C 10.149349 11.788300 12.610487

C 10.973566 11.674881 11.444396

C 11.525125 12.837332 10.857439

H 11.263304 13.799200 11.279751

C 12.386419 12.747285 9.796725

H 12.809199 13.644740 9.357381

C 12.741886 11.480760 9.298220

H 13.455320 11.401869 8.484140

C 12.203309 10.339939 9.834952

H 12.503977 9.380402 9.442547

C 11.276043 10.388875 10.904298

C 10.658453 9.239896 11.503320

C 10.810014 7.786741 11.264453

C 8.847525 7.820218 14.375192

H 8.480589 8.629673 14.992579

C 8.584255 6.509009 14.681142

C 8.997660 5.454307 13.804392

C 8.629258 4.118789 14.088451

H 8.082062 3.920415 15.001310

C 8.936556 3.105763 13.219890

C 9.609729 3.401482 12.020587

H 9.819757 2.608613 11.309820

C 9.997865 4.684499 11.732765

H 10.500510 4.885741 10.798977

C 9.738698 5.755546 12.622388

C 9.129739 13.248134 14.182500

H 8.126114 12.826390 14.055268

H 9.052622 14.325731 14.324849

O 11.310604 7.535507 4.582445

O 9.918630 13.058046 13.028858

C 14.186018 4.534938 11.062825

C 13.806080 5.329828 13.707538

C 13.844501 4.835686 8.667229

O 7.896298 6.106213 15.778866

H 8.643948 2.085268 13.443851

H 9.597453 12.799863 15.066700

C 14.469052 4.187111 9.702351

C 14.913314 3.924116 12.110666

C 14.722773 4.304430 13.412244

H 14.099927 4.631527 7.635676

C 7.440307 7.090995 16.679775

O 15.409094 3.222436 9.540370

H 15.637737 3.161026 11.855732

H 15.290273 3.834136 14.208459

H 6.941360 6.557046 17.488093

H 6.726228 7.771834 16.202215

H 8.272484 7.674528 17.090004

C 15.753985 2.839761 8.227363

H 14.885077 2.455276 7.681024

H 16.188643 3.675549 7.666962

H 16.496996 2.048751 8.325661

OS-**1c** (≡ *S_0_*-**1c**, S^2^ = 0.524198)

0 1

C 11.233809 7.422532 8.761625

C 11.464681 7.169935 10.186397

C 12.510922 6.147220 10.267012

C 13.267449 5.614017 11.364327

C 13.099070 5.997675 12.716223

H 12.370671 6.756070 12.962039

H 13.695710 5.759879 14.743895

C 12.890544 5.814195 8.966659

C 12.084848 6.591963 8.032316

C 10.239435 8.180936 8.056756

C 9.211521 8.929302 8.678426

H 9.137861 8.929867 9.755800

C 8.299602 9.643140 7.943007

H 7.521805 10.201509 8.454087

C 8.359296 9.652649 6.538164

H 7.638527 10.227105 5.965720

C 9.320366 8.915904 5.897144

H 9.369131 8.888874 4.815923

C 10.261873 8.158558 6.630573

C 11.215961 7.340941 5.939737

C 12.094642 6.542465 6.630770

H 12.782807 5.883904 6.116862

C 12.048091 6.618565 3.834672

H 11.836549 6.832078 2.787266

H 11.899309 5.548956 4.022322

H 13.089562 6.879776 4.054598

C 10.068348 7.124892 12.354073

C 9.568844 8.105923 13.210824

C 9.990262 9.412670 12.719034

C 9.757409 10.670644 13.293637

H 9.175634 10.751564 14.202685

C 10.284198 11.780560 12.679005

C 11.110555 11.656479 11.513777

C 11.700064 12.806832 10.941847

H 11.478234 13.770886 11.381781

C 12.544501 12.703050 9.867830

H 12.995233 13.591927 9.438951

C 12.840213 11.434761 9.337520

H 13.531644 11.345228 8.505740

C 12.268634 10.304278 9.864125

H 12.520123 9.341080 9.445613

C 11.366387 10.370171 10.952582

C 10.721714 9.232503 11.544925

C 10.802799 7.794171 11.277160

C 8.774871 7.816520 14.330092

H 8.420946 8.618358 14.965056

C 8.462043 6.504603 14.591235

C 8.878348 5.456334 13.705891

C 8.482589 4.122821 13.957023

H 7.898975 3.918498 14.845726

C 8.814805 3.116306 13.088748

C 9.545633 3.416278 11.925496

H 9.785342 2.627109 11.220015

C 9.955570 4.699641 11.666785

H 10.506564 4.905804 10.761215

C 9.661346 5.762794 12.553508

C 9.313333 13.254542 14.269738

H 8.291798 12.885144 14.123864

H 9.286990 14.331404 14.434277

O 11.149759 7.404384 4.586457

O 10.104360 13.050653 13.119792

C 14.283357 4.654396 11.077906

C 13.848945 5.437247 13.719016

C 13.907176 4.893059 8.675319

O 7.728746 6.095712 15.656543

H 8.502349 2.096673 13.288550

H 9.749473 12.765578 15.148430

C 14.571680 4.297031 9.719536

C 15.030749 4.080789 12.131631

C 14.815250 4.457061 13.431372

H 14.154603 4.666916 7.646229

C 7.270304 7.070292 16.567420

O 15.550532 3.370462 9.567571

H 15.787029 3.346373 11.885014

H 15.397428 4.013784 14.232481

H 6.726712 6.530878 17.342622

H 6.594826 7.785201 16.083640

H 8.104197 7.616579 17.022896

C 15.901122 2.975270 8.259678

H 15.046813 2.537059 7.731100

H 16.289700 3.818119 7.676565

H 16.682199 2.223193 8.367835

*T_1_*-**1c**

0 3

C 11.031778 7.146863 8.800105

C 11.476636 7.158734 10.169126

C 12.712850 6.424459 10.238571

C 13.593226 6.115003 11.326533

C 13.374233 6.522234 12.662612

H 12.493603 7.104147 12.899383

H 14.064496 6.518253 14.676656

C 13.022887 5.971084 8.946542

C 11.968507 6.424077 8.044398

C 9.883489 7.709125 8.152226

C 8.882904 8.452639 8.819280

H 8.970733 8.618865 9.884577

C 7.806064 8.964824 8.138647

H 7.053159 9.531778 8.676843

C 7.670627 8.761728 6.754054

H 6.815765 9.170758 6.225730

C 8.622443 8.045222 6.074310

H 8.534243 7.879092 5.008009

C 9.740284 7.507125 6.749484

C 10.731819 6.758262 6.028932

C 11.830286 6.224061 6.665913

H 12.576466 5.659937 6.120951

C 11.415103 5.909289 3.915725

H 11.031268 5.932026 2.896167

H 11.496705 4.868178 4.248322

H 12.408172 6.372413 3.938735

C 9.801508 7.196884 12.145757

C 9.399009 8.149901 13.094814

C 10.143528 9.379913 12.842724

C 10.092630 10.604960 13.517991

H 9.426284 10.733462 14.361487

C 10.904045 11.630678 13.085254

C 11.789056 11.462969 11.966429

C 12.611560 12.529555 11.541181

H 12.557170 13.468803 12.077117

C 13.459669 12.378694 10.473777

H 14.087284 13.204475 10.155336

C 13.513392 11.149825 9.792839

H 14.184252 11.030122 8.948104

C 12.724602 10.096828 10.185531

H 12.779035 9.158652 9.649739

C 11.838726 10.215260 11.281032

C 10.988498 9.158123 11.743700

C 10.790878 7.804722 11.294898

C 8.440280 7.875650 14.077145

H 8.158655 8.639945 14.790273

C 7.871441 6.621460 14.111466

C 8.243221 5.605525 13.166586

C 7.645859 4.326820 13.221743

H 6.906419 4.135127 13.989112

C 7.994636 3.350471 12.323694

C 8.957361 3.622109 11.335738

H 9.233605 2.849790 10.624984

C 9.553450 4.856649 11.259839

H 10.292158 5.047837 10.493057

C 9.219236 5.887333 12.168099

C 10.103780 13.110875 14.765075

H 9.046091 12.999537 14.500325

H 10.293549 14.142020 15.061843

O 10.491123 6.630681 4.700577

O 10.941850 12.858906 13.658603

C 14.761461 5.349922 11.045684

C 14.258304 6.191905 13.659850

C 14.170132 5.219136 8.667736

O 6.934516 6.240014 15.014464

H 7.529597 2.371524 12.374907

H 10.336002 12.444559 15.603701

C 15.026119 4.913711 9.702994

C 15.654062 5.024231 12.090742

C 15.409546 5.436449 13.375924

H 14.371453 4.889311 7.656449

C 6.510701 7.176643 15.980517

O 16.165534 4.193565 9.555292

H 16.535577 4.441758 11.854025

H 16.101764 5.180782 14.171342

H 5.767406 6.666884 16.592890

H 6.052689 8.055599 15.512696

H 7.342441 7.500842 16.616362

C 16.503097 3.724992 8.268191

H 15.736308 3.048270 7.874138

H 16.646581 4.553317 7.564998

H 17.440562 3.180687 8.378799

**1c**-TS_rot_ (OS, S^2^ = 1.111349)

0 1

C 11.031893 7.146712 8.799429

C 11.476448 7.158943 10.169353

C 12.713281 6.424113 10.238209

C 13.593577 6.114563 11.326039

C 13.374577 6.521965 12.662107

H 12.494013 7.104077 12.898693

H 14.064827 6.518006 14.676152

C 13.023110 5.970911 8.946364

C 11.968591 6.424055 8.044032

C 9.883761 7.708921 8.151471

C 8.883116 8.452388 8.818567

H 8.970945 8.618437 9.883903

C 7.806355 8.964631 8.137903

H 7.053384 9.531541 8.676049

C 7.671066 8.761644 6.753240

H 6.816250 9.170746 6.224895

C 8.622894 8.045199 6.073472

H 8.534763 7.879174 5.007149

C 9.740700 7.507012 6.748690

C 10.732301 6.758206 6.028308

C 11.830669 6.224009 6.665525

H 12.576934 5.659848 6.120718

C 11.416073 5.909349 3.915224

H 11.032510 5.932139 2.895559

H 11.497672 4.868206 4.247751

H 12.409110 6.372539 3.938529

C 9.801130 7.196945 12.146299

C 9.398885 8.149893 13.095161

C 10.143468 9.380158 12.842977

C 10.092277 10.605164 13.518306

H 9.425881 10.733502 14.361786

C 10.903502 11.631084 13.085605

C 11.788527 11.463512 11.966862

C 12.610972 12.530147 11.541509

H 12.556488 13.469453 12.077336

C 13.459108 12.379203 10.474161

H 14.086662 13.205001 10.155639

C 13.513019 11.150239 9.793315

H 14.183970 11.030546 8.948653

C 12.724324 10.097233 10.186067

H 12.778806 9.158933 9.650471

C 11.838360 10.215758 11.281542

C 10.988316 9.158620 11.744157

C 10.791033 7.804569 11.294621

C 8.440328 7.875811 14.077699

H 8.158841 8.640195 14.790785

C 7.871435 6.621619 14.112256

C 8.243027 5.605674 13.167415

C 7.645735 4.326908 13.222624

H 6.906425 4.135141 13.990100

C 7.994450 3.350640 12.324481

C 8.957013 3.622328 11.336325

H 9.233140 2.850019 10.625517

C 9.553022 4.856875 11.260365

H 10.291590 5.048241 10.493478

C 9.218880 5.887510 12.168764

C 10.102860 13.111097 14.765408

H 9.045200 12.999542 14.500598

H 10.292393 14.142273 15.062236

O 10.491835 6.630604 4.699887

O 10.941046 12.859344 13.658982

C 14.761708 5.349299 11.045204

C 14.258554 6.191565 13.659362

C 14.170196 5.218785 8.667365

O 6.934638 6.240280 15.015471

H 7.529483 2.371660 12.375734

H 10.335164 12.444805 15.604039

C 15.026225 4.913114 9.702551

C 15.654240 5.023531 12.090341

C 15.409704 5.435874 13.375463

H 14.371373 4.889009 7.656035

C 6.511051 7.177050 15.981478

O 16.165527 4.192752 9.554719

H 16.535684 4.440922 11.853696

H 16.101846 5.180148 14.170930

H 5.767834 6.667412 16.594055

H 6.053005 8.055984 15.513634

H 7.342920 7.501287 16.617145

C 16.502878 3.724232 8.267550

H 15.735929 3.047674 7.873504

H 16.646443 4.552587 7.564397

H 17.440266 3.179753 8.378007

*S_0_*-(*M*,*M*)-**3**

0 1

C 12.358688 8.511605 6.079749

C 11.899435 9.813740 6.079715

C 13.698483 8.033729 5.676204

C 10.559661 10.291648 5.676118

C 13.764075 6.672699 5.892307

C 10.494090 11.652672 5.892266

C 12.417878 6.197895 6.266860

C 11.840287 12.127452 6.266838

C 11.615134 7.298890 6.483178

C 12.643040 11.026448 6.483064

C 14.741489 8.690615 4.918810

C 9.516664 9.634822 4.918652

C 15.960276 7.992152 4.715829

C 8.297891 10.333318 4.715685

C 16.149669 6.680142 5.319163

C 8.108515 11.645310 5.319062

C 15.037622 5.998295 5.876117

C 9.220568 12.327116 5.876048

C 14.566162 9.933688 4.276520

C 9.691988 8.391797 4.276267

H 13.619369 10.446311 4.361354

H 10.638775 7.879161 4.361070

C 15.560151 10.492268 3.508029

C 8.698005 7.833281 3.507722

H 15.389049 11.444755 3.017558

H 8.869108 6.880828 3.017183

C 16.775504 9.820525 3.338158

C 7.482662 8.505046 3.337879

H 17.559034 10.252424 2.724450

H 6.699139 8.073200 2.724125

C 16.959685 8.590076 3.924447

C 7.298489 9.735460 3.924245

H 17.885411 8.059057 3.740665

H 6.372778 10.266507 3.740472

C 17.415396 6.066963 5.388998

C 6.842807 12.258530 5.388872

H 18.280885 6.578160 4.986482

H 5.977310 11.747367 4.986329

C 17.593626 4.846885 6.000141

C 6.664606 13.478614 6.000014

H 18.583465 4.404975 6.047086

H 5.674781 13.920558 6.046936

C 16.504281 4.198612 6.594086

C 7.753963 14.126858 6.593970

H 16.646938 3.260275 7.119732

H 7.611329 15.065206 7.119601

C 15.254290 4.768522 6.536268

C 9.003936 13.556907 6.536176

H 14.428594 4.288553 7.043537

H 9.829646 14.036858 7.043441

C 11.849202 4.873755 6.282802

C 12.408949 13.451595 6.282837

C 10.555466 4.707004 6.839726

C 13.702725 13.618324 6.839674

C 9.879816 5.847415 7.443211

C 14.378438 12.477879 7.443025

C 10.390713 7.155987 7.240392

C 13.867527 11.169316 7.240174

C 12.451923 3.780305 5.622367

C 11.806146 14.545087 5.622549

H 13.395985 3.924684 5.115059

H 10.862032 14.400734 5.115329

C 11.836028 2.552330 5.564302

C 12.422017 15.773077 5.564526

H 12.313560 1.732240 5.038423

H 11.944421 16.593202 5.038760

C 10.581071 2.373633 6.158302

C 13.677025 15.951745 6.158426

H 10.087472 1.408537 6.111192

H 14.170605 16.916853 6.111348

C 9.954480 3.435451 6.769676

C 14.303686 14.889891 6.769666

H 8.959797 3.290500 7.172178

H 15.298400 15.034828 7.172097

C 8.726420 5.685894 8.234546

C 15.531901 12.639354 8.234270

H 8.338779 4.691559 8.418273

H 15.919552 13.633680 8.418029

C 9.747179 8.233907 7.882630

C 14.511127 10.091356 7.882279

H 10.162710 9.227170 7.797900

H 14.095596 9.098095 7.797513

C 8.097745 6.759541 8.820814

C 16.160633 11.565672 8.820414

C 8.622608 8.045166 8.650950

C 15.635766 10.280054 8.650509

H 7.216613 6.604210 9.434468

H 17.041819 11.720969 9.433999

H 8.158123 8.894200 9.141323

H 16.100303 9.430991 9.140783

*T_1_*-(*M*,*M*)-**3**

0 3

C 12.372111 8.470878 6.079817

C 11.884435 9.854007 6.080321

C 13.128667 7.840186 5.029332

C 10.549698 10.281700 5.748876

C 13.460504 6.552762 5.449993

C 10.477313 11.660291 5.944994

C 12.729221 6.294213 6.708746

C 11.848475 12.143384 6.214571

C 12.179171 7.504257 7.129949

C 12.656118 11.023827 6.411525

C 13.543599 8.338338 3.739194

C 9.405001 9.526921 5.296538

C 14.404511 7.527422 2.956923

C 8.165931 10.205184 5.172556

C 14.958313 6.301235 3.523660

C 8.044936 11.584191 5.636315

C 14.500782 5.824983 4.784283

C 9.201329 12.305999 6.045033

C 13.077276 9.554303 3.201573

C 9.483410 8.174013 4.911048

H 12.410129 10.173822 3.784670

H 10.426553 7.651351 4.989580

C 13.431245 9.956906 1.934432

C 8.388159 7.510477 4.407961

H 13.052524 10.894495 1.541261

H 8.477094 6.470567 4.111715

C 14.261926 9.148874 1.151152

C 7.170059 8.182557 4.262858

H 14.530316 9.453374 0.145005

H 6.307768 7.668988 3.850806

C 14.733913 7.959583 1.657843

C 7.068717 9.502695 4.638164

H 15.362374 7.342520 1.028083

H 6.120538 10.007446 4.502104

C 15.992679 5.593160 2.883083

C 6.792751 12.216609 5.753640

H 16.361942 5.933458 1.924231

H 5.895913 11.690971 5.452085

C 16.589607 4.496160 3.462986

C 6.663606 13.478872 6.288408

H 17.391546 3.981098 2.944495

H 5.681002 13.931148 6.373053

C 16.192014 4.076958 4.738359

C 7.793708 14.155569 6.763136

H 16.697575 3.249401 5.224776

H 7.691392 15.125630 7.238064

C 15.173052 4.734505 5.384330

C 9.033569 13.574770 6.647708

H 14.901593 4.438310 6.388226

H 9.897174 14.079213 7.058743

C 12.377122 5.074317 7.374284

C 12.438246 13.446052 6.113251

C 11.722262 5.157182 8.635080

C 13.791799 13.608573 6.521730

C 11.382801 6.458980 9.202046

C 14.561545 12.458603 6.987034

C 11.543401 7.630920 8.420061

C 14.021029 11.153442 6.864242

C 12.539053 3.803657 6.773998

C 11.773970 14.539327 5.509394

H 12.936051 3.743948 5.769977

H 10.784759 14.390990 5.098685

C 12.159706 2.651797 7.419915

C 12.376425 15.768662 5.392350

H 12.286234 1.690407 6.933332

H 11.848317 16.588240 4.916494

C 11.587402 2.727922 8.695491

C 13.681275 15.949873 5.866604

H 11.287342 1.823279 9.213962

H 14.163769 16.917877 5.780604

C 11.362658 3.956306 9.275596

C 14.371519 14.885825 6.402680

H 10.861893 3.988902 10.234615

H 15.399914 15.038339 6.703834

C 10.854730 6.588105 10.501053

C 15.856542 12.599359 7.521885

H 10.753074 5.713088 11.130613

H 16.279001 13.587070 7.657118

C 11.142421 8.869896 8.957897

C 14.807505 10.050432 7.251391

H 11.272515 9.771082 8.374954

H 14.400067 9.051988 7.173964

C 10.474884 7.809864 11.007911

C 16.604668 11.507490 7.898783

C 10.613900 8.960583 10.224936

C 16.076618 10.220266 7.754897

H 10.074536 7.877993 12.013976

H 17.598265 11.648087 8.311193

H 10.319604 9.927926 10.618282

H 16.658799 9.354467 8.052459
